# Design and enantioselective synthesis of 3-(α-acrylic acid) benzoxaboroles to combat carbapenemase resistance†

**DOI:** 10.1039/d1cc03026d

**Published:** 2021-07-14

**Authors:** You-Cai Xiao, Xiao-Pan Chen, Ji Deng, Yu-Hang Yan, Kai-Rong Zhu, Gen Li, Jun-Lin Yu, Jürgen Brem, Fener Chen, Christopher J. Schofield, Guo-Bo Li

**Affiliations:** Key Laboratory of Drug-Targeting and Drug Delivery System of the Ministry of Education and Sichuan Research Center for Drug Precision Industrial Technology, West China School of Pharmacy, Sichuan University Chengdu 610041 China liguobo@scu.edu.cn; Department of Chemistry and the Ineos Oxford Institute for Antimicrobial Research, University of Oxford, 12 Mansfield Road Oxford OX1 3TA UK christopher.schofield@chem.ox.ac.uk

## Abstract

Chiral 3-substituted benzoxaboroles were designed as carbapenemase inhibitors and efficiently synthesised *via* asymmetric Morita–Baylis–Hillman reaction. Some of the benzoxaboroles were potent inhibitors of clinically relevant carbapenemases and restored the activity of meropenem in bacteria harbouring these enzymes. Crystallographic analyses validate the proposed mechanism of binding to carbapenemases, *i.e.* in a manner relating to their antibiotic substrates. The results illustrate how combining a structure-based design approach with asymmetric catalysis can efficiently lead to potent β-lactamase inhibitors and provide a starting point to develop drugs combatting carbapenemases.

## Abstract 2

An organocatalytic MBH cascade reaction was developed to construct new 3-(α-acrylic acid) benzoxaboroles, designed to mimic ‘anchoring’ pharmacophore features of carbapenems, with the aim of helping overcome carbapenemase resistance.

## Graphical abstract

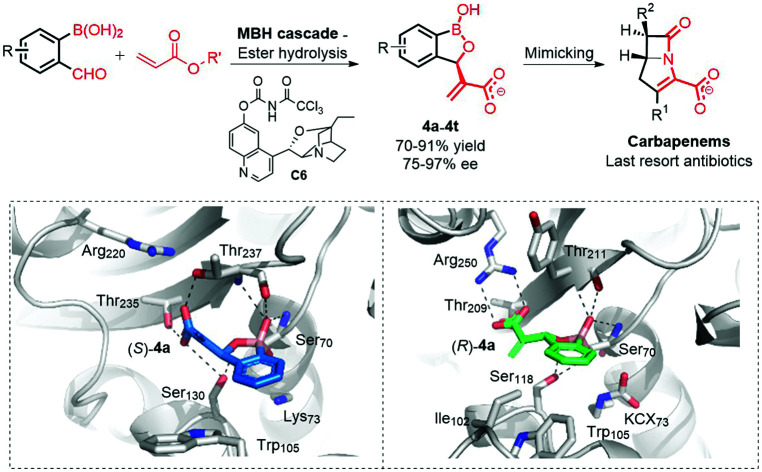

The benzoxaborole ring system has emerged as a useful drug scaffold, due to its intrinsic reactivity, metabolic stability and low toxicity.^1^ Benzoxaborole derivatives have been developed to treat infectious, inflammatory and neoplastic diseases;^2^ Tavaborole and Crisaborole are approved for treatment of onychomicosis and mild-to-moderate atopic dermatis, respectively and other benzoxaboroles are in development (Fig. 1a).^3^ However, most benzoxaborole containing drugs/drug candidates have achiral substituents, hampering the development of selectivity. The development of efficient new methods for the asymmetric construction of chiral benzoxaboroles, including with C3 substituents is a current challenge in the development of boron containing drugs.

**Fig. 1:**
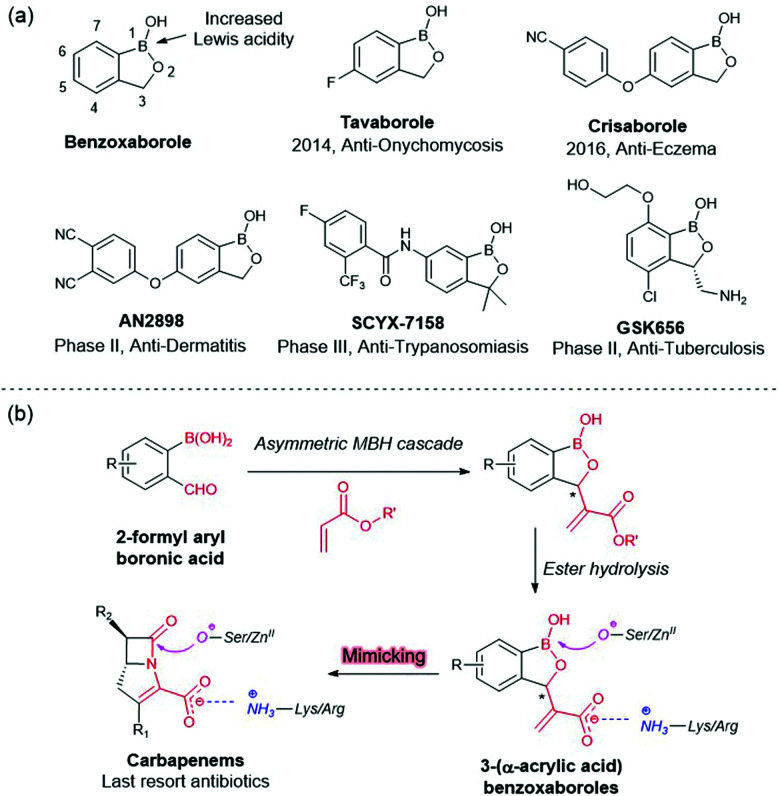
Design of 3-substitued benzoxaboroles as β-lactamase Inhibitors.

Procedures have been recently described for synthesis of C3-substituted benzoxaboroles *via* reaction of nucleophiles with 2-formyl aryl boronic acid.^1*a*^ Most, however, suffer from low yields and limited substrate scope.^1*b*^ Indeed, there has been little progress towards the asymmetric synthesis of 3-substituted benzoxaboroles suitable for biological testing, with only a Wittig/oxa-Michael reaction cascade from 2-formyl aryl boronic acid having been reported.^4^

A major mechanism of carbapenem resistance involves production of carbapenemases, *i.e.* evolved β-lactamases that inactivate carbapenem antibiotics, which can be divided into two categories: serine-carbapenemases (*e.g.* Ambler class A KPC-2 and class D OXA-48 types) and metallo-carbapenemases (*e.g.* NDM and VIM types).^5^ With the aim of helping enable agents to overcome carbapenem resistance in Gram-negative pathogens,^6^ we analysed carbapenem-substrate derived complexes and identified benzoxaboroles with a C3 acrylic acid group as potential inhibitors (Fig. 1b). Here we report use of an asymmetric Morita–Baylis–Hillman (MBH) cascade reaction^7^ to construct the desired chiral C3-substituted benzoxaboroles. Some of the compounds manifested inhibition of clinically relevant carbapenemases, with the proposed mode of action being validated by crystallography and potentiation of carbapenem activity in cells.

Initially, we utilized 2-formyl-phenylboronic acids as the electrophiles and hexafluoroisopropyl acrylate (HFIPA) as the activated alkene in model reactions. We envisaged challenges relating to the limited substrate scope of the MBH reaction, the desire for asymmetric induction,^7*c*^ and the presence of a boronic acid group at the benzaldehyde *ortho*-position. After optimization of the model reaction, we identified reaction conditions using cinchona alkaloid derived chiral tertiary amines **C6** and **C7** as catalysts (see ESI† for optimization details).

We explored the scope of the asymmetric reaction with various substituted 2-formylphenylboronic acids using **C6** (Table 1). The products **3** were hydrolyzed to the corresponding carboxylic acids **4** for biological evaluation. The chiral MBH adducts made using **C6** were assigned the *R*-configuration, based on X-ray analysis of a single crystal of **4a**.^8^

**Table 1:** Substrate scope with small substituents on the phenyl ring of 2-formylphenylboronic acids^a^

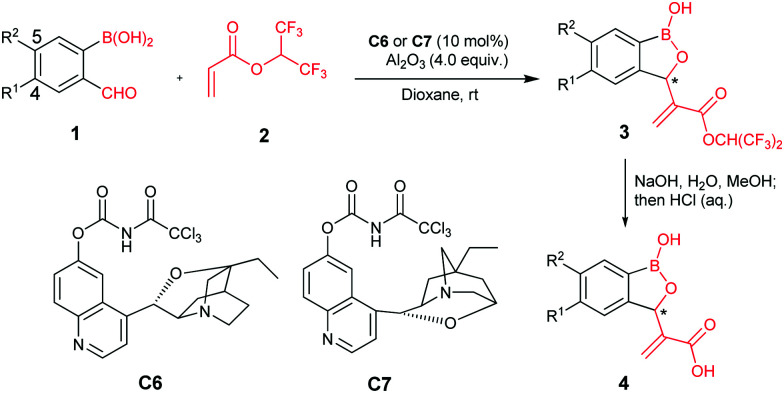					
Entry	R^1^	R^2^	Time^b^ (h)	ID, Yield^c^ (%)	ee^d^^e^ (%)
1	H	H	29	(*R*)-**4a**, 80	90(96)
2	Me	H	29	(*R*)-**4b**, 80	89
3	OMe	H	43	(*R*)-**4c**, 85	90(99)
4	OEt	H	10	(*R*)-**4d**, 82	86
5	OiPr	H	24	(*R*)-**4e**, 71	82
6	F	H	46	(*R*)-**4f**, 86	82(90)
7	Cl	H	22	(*R*)-**4g**, 70	75
8	H	Me	7	(*R*)-**4h**, 84	88
9	H	OMe	17	(*R*)-**4i**, 72	84
10	H	F	36	(*R*)-**4j**, 91	81(96)
11	H	Cl	36	(*R*)-**4k**, 81	78
12	OMe	OMe	48	(*R*)-**4l**, 84	82
13^f^	H	H	96	(*S*)-**4a**, 88	60(99.5)
14^f^	OMe	H	90	(*S*)-**4c**, 80	52(96)
15^f^	F	H	76	(*S*)-**4f**, 79	48(90)
16^f^	H	F	48	(*S*)-**4j**, 65	57(95)

a Standard reaction conditions: **1** (0.1 mmol), **2** (0.5 mmol), **C6** or **C7** (0.01 mmol), Al_2_O_3_ (0.4 mmol), 1,4-dioxane (4.0 mL), r.t.

b Reaction time for the first step.

c Isolated yields over two steps.

d ee's were determined by chiral HPLC.

e ee after recrystallization from ethyl acetate.

f Reaction at 0 °C.

The asymmetric MBH reaction with aromatic aldehydes bearing electron-donating groups is reported to be challenging, owing to their low reactivity.^7^ However, electron-donating groups, *e.g.* methyl and alkoxy groups at C4 of the 2-formylphenylboronic ring are well tolerated in our conditions (Table 1, entries 1–5); it is possible that the aldehyde is activated by an intramolecular hydrogen bond between its carbonyl oxygen and the boronic acid, so promoting MBH reaction.^9^ By contrast, electron-withdrawing substituents at the same position in the phenyl ring delivered relatively low enantioselectivities (entries 6 and 7). Similarly, substituents at C5 of the 2-formylphenylboronic ring were amenable to reaction regardless of the electronic properties of the tested substituents (entries 8–12). However, substituents at either C3- or C6- of the 2-formylphenylboronic ring were not amenable to reaction, with only traces of the desired products being observed, revealing a limitation of the methodology. We applied catalyst **C7** for the asymmetric MBH synthesis of (*S*)-enantiomers; the desired products were observed in good yield, albeit with moderate ee (entries 13–16); pleasingly, excellent ee's were obtained after recrystallization.

We next examined the reaction scope with larger substituents, *e.g.* aryloxy and benzyloxy groups, at C4 and C5- of 2-formylphenylboronic acids, since structural analyses suggested these derivatives are of interest as β-lactamase inhibitors (representatives are given in Scheme 1).^10^ Aryloxy substituents, (*p*-tolyloxy, *m*-tolyloxy, *p*-trifluoromethyl-phenoxy) at C4 of 2-formylphenylboronic acids gave products (**4m–4o**) in high yields and good enantioselectivities. A benzyloxy group at C4 was also amenable to reaction, affording **4p** in 81% yield and 87% ee. By contrast, aryloxyl- and benzyloxy substituents at C5 of 2-formylphenylboronic acids gave relatively lower ee‘s compared to the C4 derivatives (**4q–4t**). Density functional theory (DFT) calculations inform on the mechanism of reaction and rationalise its stereoselectivity (see E. 1 Chemistry in ESI†).

**Scheme 1:**
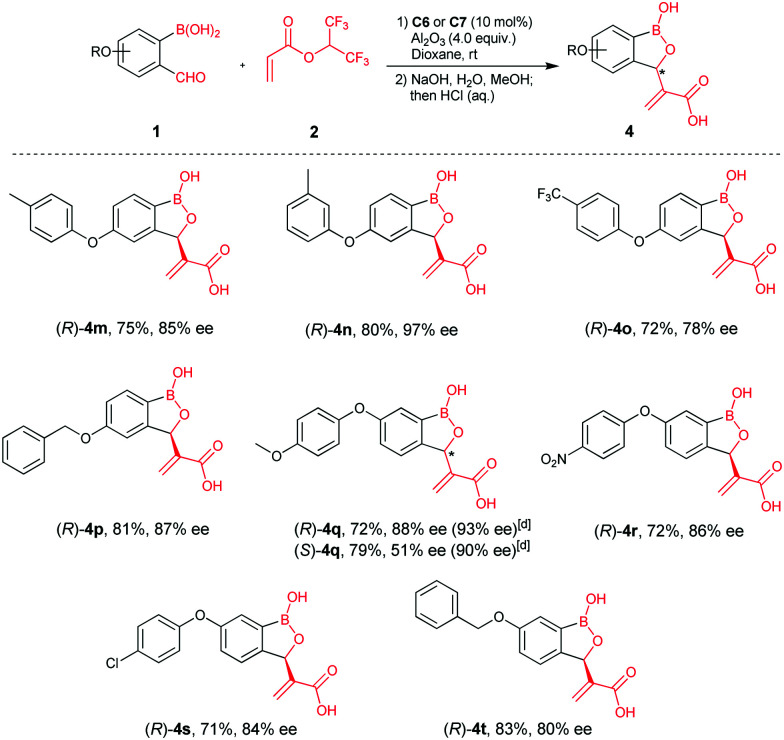
Scope of C3 substituted benzoxaborole synthesis employing aryloxy and benzyloxy substituted 2-formylphenylboronic acids.^*a*,*b*,*c a*^Unless otherwise stated, reactions were performed with **1** (0.1 mmol), **2** (0.5 mmol), **C6** or **C7** (0.01 mmol), Al_2_O_3_ (0.4 mmol), 1,4-dioxane (4.0 mL), r.t. 12–48 h. ^*b*^Isolated yields over two steps. ^*c*^ee's determined by chiral HPLC. ^*d*^ee after recrystallization.

We then tested the activity of the benzoxaborole derivatives against purified recombinant forms of 7 types of clinically relevant β-lactamases, *i.e.* class A SBL KPC-2, class C SBL AmpC, class D SBL OXA-48, class B1 MBLs NDM-1, IMP-1, VIM-1, and VIM-2; the SBL inhibitor avibactam was used as a positive control (Table 2 and Table S1, ESI†).

**Table 2:** Comparison of the inhibitory activities (IC_50_, μM) of (*R*)- and (*S*)-configuration of selected benzoxaboroles^a^

Cpd ID	Class A KPC-2	Class C AmpC	Class D OXA-48	Class B1 NDM-1	Class B1 IMP-1	Class B1 VIM-1	Class B1 VIM-2
(*R*)-**4a**	3.08	134.10	15.84	549.80	>600	>600	225.40
(*S*)-**4a**	0.48	185.7	14.06	>600	142.5	308.6	12.05
(*R*)-**4c**	7.62	178.20	42.52	>600	>600	>600	232.60
(*S*)-**4c**	0.54	74.14	34.15	169.7	>600	466.3	22.40
(*R*)-**4f**	3.87	140.90	10.13	>600	65.65	513.8	82.80
(*S*)-**4f**	0.34	149.8	14.68	325.3	46.5	>600	120.5
(*R*)-**4j**	0.97	140.50	5.97	>600	>600	>600	55.87
(*S*)-**4j**	0.49	108.7	12.03	719.1	142.5	308.6	105.7
(*R*)-**4q**	0.07	18.38	0.43	260.00	497.36	388.3	57.37
(*S*)-**4q**	0.01	22.52	0.74	34.06	>600	>600	58.49
Avibactam	0.004	0.24	0.17	>600	425	>600	19.08

a All tested compounds were recrystallised. IC_50_ curves are shown in ESI Fig. S1.

The results (Table 2) show both enantiomers of some of the chiral benzoxaborole derivatives manifest moderate, or potent, inhibition of the carbapenemase KPC-2, demonstrating their potential for inhibition of clinically relevant SBLs. In all cases, the (*S*)-configured compounds (**4a**, **4c**, **4f**, **4j**, **4q**) were more potent than the corresponding (*R*)-enantiomers, illustrating how chirality can confer selectivity in boron based SBL inhibitors. This selectivity was mostly, but not exclusively observed for the other β-lactamases, substantial inhibition of which was more sporadic than for KPC-2 (Table 2). The KPC-2 stereoselectivity for the benzoxaborole derivatives is interesting since the penicillin substrates of some of the β-lactamases have a (2*S*) C-3 carbon adjacent to their C-2 carboxylate. The difference in results for the (*R*)- and (*S*)-benzoxaboroles may reflect different distances between their carboxylate and electrophilic boron; the analogous carboxylate to β-lactam distance is proposed to be important with respect to antibacterial activity.^11^

Overall, these results reveal that when combined with an appropriate C-5 or C-6 substituent, the C-3 acrylate substituted benzoxaborole derivatives are potent KPC-2 inhibitors. They were in general much less potent inhibitors for the other tested SBLs/MBLs, but in some cases potent inhibition was observed, suggesting that with optimisation the scaffold could be developed into potent broad spectrum β-lactamase inhibitors.

The abilities of selected Inhibitors to potentiate the antibacterial activity of meropenem (MEM), a clinically important carbapenem, was then investigated for AmpC, TEM-1, or KPC-2 producing clinically derived strains, including *Klebsiella Pneumoniae* C692, *Escherichia coli* BAA-2340, and *Escherichia coli* 11119 (for details, see ESI†). None of these isolates were susceptible to MEM alone (MIC ≥ 16 μg ml^−1^); for the control strain ATCC25922, the MEM MIC is 0.06 μg ml^−1^.

Addition of **4a**, **4c** or **4q** at 100 μM substantially reduced the MEM MICs, *i.e.* by 16-128 fold for the KPC-2 producing *E. coli* strains; notably, the presence of 10 μM (*R*)-**4a** and (*S*)-**4a** decreased the MEM MICs to below 2 μg ml^−1^ for *E. coli* BAA-2340 and *E. coli* 11119 (Table 3). Consistent with the biochemical results, reduced (but still clear) activity was observed for AmpC and TEM-1 (both class A SBLs) producing *K. pneumoniae* C692, *i.e.* the addition of 100 μM (*S*)-**4a** resulted in a 16-fold decrease in the MEM MICs. Overall, consistent with the biochemical results, the (*S*)-compounds show better cellular activity than the corresponding (*R*)-enantiomers, illustrating the importance of stereochemistry. Interestingly, although **4q** was a better inhibitor than **4a***versus* against the isolated β-lactmases (Table 2), it was less active in restoring MEM activity in cells (Table 3), possibly reflecting different permeation or efflux properties.

**Table 3:** *In vitro* cell-based screening of **4a**, **4c**, and **4q**^a^

Compound	Conc. (μM)	MEM (μg ml^−1^) in presence or absence of inhibitor		
		*K. pneumoniae* C692 (blaAmpC, blaTEM-1)	*E. coli* BAA-2340 (blaKPC-2)	*E. coli* 11119 (blaKPC-2)
—	—	>128	16	64
(*R*)-**4a**	100	16	0.25	0.5
	10	128	0.5	4
(*S*)-**4a**	100	8	0.25	0.5
	10	64	0.5	2
(*R*)-**4c**	100	128	0.5	2
	10	>128	2	16
(*S*)-**4c**	100	32	0.25	1
	10	128	4	4
(*R*)-**4q**	100	>128	1	2
	10	>128	2	16
(*S*)-**4q**	100	64	0.25	2
	10	>128	1	16

a Clinically isolated strains producing KPC-2, AmpC, and/or TEM-1.

To investigate the structural basis of inhibition by the chiral benzoxaboroles, we obtained KPC-2:(*S*)-**4a** and OXA-48:(*R*)-**4a** complex structures by co-crystallisation (Table S2, ESI†). The KPC-2: (*S*)-**4a** structure reveals a covalent bond between boron and Ser70, with hydrogen bonds between the inhibitor and β3 Thr237, α8 Ser130, β3 Thr235, and β3 Thr237 (Fig. 2a and Fig. S4, ESI†). The OXA-48:(*R*)-**4a** structure similarly reveals a tetrahedral boron with hydrogen bonds between the inhibitor α3 Ser70, β5 Tyr211, α5 Ser118, and α10 Arg250; Lys73 of OXA-48 was refined in its catalytically active carbamylated form (Fig. 2b and Fig. S5, ESI†). Superimposition of structures of KPC-2:(*S*)-**4a** with KPC-2:reacted imipenem (PDB: 6XJ8),^12^ and OXA-48:(*R*)-**4a** with OXA-48:reacted meropenem (PDB: 6P98)^13^ reveals that (*S*)-**4a** and (*R*)-**4a** have binding modes closely related with those of ring-opened products of carbapenems (for more details, see Fig. S7–S9, ESI†). Based on these structures and computational results, it is proposed that the C-6 4-methoxyphenoxyl group of (*S*)-**4q**, which occupies a hydrophobic subpocket adjacent to OXA-48 active site, forms an additional hydrogen bond with Asn170 of KPC-2 (Fig. S6, ESI†). This putative interaction may explain why **4q** is more potent than **4a** and suggests that further optimization of substituents particularly at C-6 should improve the potency of the series for carbapenemase inhibition.

**Fig. 2:**
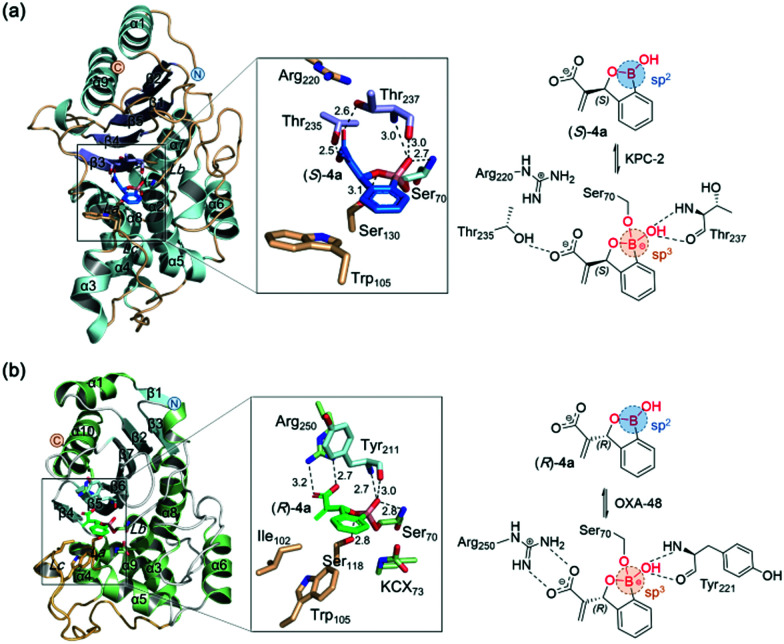
Crystallographic analyses of the KPC-2:(*S*)-**4a** (PDB: 7E9A) and OXA-48:(*R*)-**4a** (PDB: 7DML) complexes.

In summary, an enantioselective MBH cascade reaction, employing a bifunctional tertiary amine-carbamate catalyst, was developed for efficient synthesis of 3-substituted benzoxaboroles. The reaction enabled synthesis of C3 acrylate substituted benzoxaboroles in high yield and enantiomeric excess. Screening against clinically relevant SBLs and MBLs, revealed that some of the benzoxaborole derivatives are potent inhibitors of SBLs/MBLs. Crystallographic analyses revealed that active site of (*S*)-**4a** to KPC-2 and of (*R*)-**4a** to OXA-48 involves reaction with the nucleophilic serine. (*R*)-**4a** and (*S*)-**4a** potentiate carbapenem activity in cells. The results thus provide an excellent starting point for optimisation to develop selective benzoxaborole derivatives to combat carbapenemase resistance.

The authors thank the staff of BL19U1 beamline of the National Center for Protein Science Shanghai at Shanghai Synchrotron Radiation Facility for assistance during data collection. The authors are grateful to the National Natural Science Foundation (Grant numbers: 81874291, 21907072, and 82073698). CJS thanks the Medical Research Council and the Wellcome Trust for funding. This work was funded in whole, or in part, by the Wellcome Trust (Grant number 106244/Z/14/Z).

## Conflicts of interest

There are no conflicts to declare.

## Supplementary Material

CC-057-D1CC03026D-s001
