# Supplementary material for: Design and enantioselective synthesis of 3-(α-acrylic acid) benzoxaboroles to combat carbapenemase resistance
Source: Chem Commun (Camb). 2021 Jul 14;57(62):7709–12. doi: 10.1039/d1cc03026d (PMC8330636; doi:10.1039/d1cc03026d)
Supplement: CC-057-D1CC03026D-s001 [file CC-057-D1CC03026D-s001.pdf]

Electronic Supplementary Material (ESI) for ChemComm.
This journal is © The Royal Society of Chemistry 2021

Supporting Information

Design and Enantioselective Synthesis of 3-(α-Acrylic Acid) Benzoxaboroles to Combat Carbapenemase Antibiotic Resistance

You-Cai Xiao,[a] Xiao-Pan Chen,[a] Ji Deng,[a] Yu-Hang Yan,[a] Kai-Rong Zhu,[a] Gen Li,[a] Jun-Lin Yu,[a] Jürgen Brem,[b] Fener Chen,[a] Christopher J. Schofield,*[b] and Guo-Bo Li*[a]

[a]Key Laboratory of Drug-Targeting and Drug Delivery System of the Ministry of Education and Sichuan Research Center for Drug Precision Industrial Technology, West China School of Pharmacy, Sichuan University, Chengdu 610041, China.

[b]Department of Chemistry and the Ineos Oxford Institute for Antimicroibial Research, University of Oxford, 12 Mansfield Road, Oxford, OX1 3TA, United Kingdom.

Correspondance: E-mail: liguobo@scu.edu.cn (G.-B. Li);

christopher.schofield@chem.ox.ac.uk (C.J. Schofield)

Contents

Supplementary Experiment (SE) Section 2

SE. 1 Chemistry 2

(1) General Experimental Methods 2

(2) General Experimental Procedure for the Synthesis of Substituted 2-Formylphenylboronic Acids **1a-1t** 2

(3) Optimization of Reaction Conditions 7

(4) General Procedure for the Catalytic Asymmetric MBH Cascade Reaction 9

(5) The proposed mechanism for the enantioselective synthesis of (*R*)-enantiomers in the presence of catalyst **C6** 18

(6) Density Functional Theory Calculations for Proposed Intermediates 19

SE. 2 Biology 20

(1) Recombinant Protein Production and Purification 20

(2) Carbapenemase Inhibition Assays 20

(3) Minimal Inhibitory Concentrations (MICs) 21

(4) Crystallization and Data Collection 21

Copies of ^{1}H and ^{13}C NMR Spectra 22

Copies of HPLC spectra 42

X-ray Crystallographic Data for 4a. 72

Supplementary Figures 73

Figure S1. IC_{50} curves of **4a-4t** and avibactam with KPC-2, AmpC, OXA-48, NDM-1, IMP-1, VIM-1, and VIM-2 100

Figure S2. The mode of (*S*)-**4a** binding to KPC-2 as defined by the electron density map 101

Figure S3. The mode of (*R*)-**4a** binding to OXA-48 as defined by the electron density map......102

Figure S4. Protein-ligand interactions between (*S*)-**4a** and KPC-2 as defined using the LigPlot+ program 103

Figure S5. Protein-ligand interactions between (*R*)-**4a** and OXA-48 as defined using the LigPlot^{+} program 104

Figure S6. The proposed binding modes of (S)-4q/(R)-4q with KPC-2 and OXA-48................105

Figure S7. Comparison of KPC-2:(*S*)-**4a**/OXA-48:(*R*)-**4a** structures with those of KPC-2/OXA-48 reacted with β-lactam substrates..106

Figure S8. Comparison of KPC-2:(*S*)-**4a**/OXA-48:(*R*)-**4a** structures with those of KPC-2/OXA-48 in complex with other boron-containing inhibitors 108

Figure S9. Binding modes of the new C-3 substituted benzoxaboroles mimics those of proposed intermediates in carbapenemase catalysis 109

Supplementary Tables 111

Table S1. The inhibitory activities of synthesized benzoxaborole derivatives against representative serine- and metallo-carbapenemases..111

Table S2. Co-crystallization conditions for the OXA-48:(*R*)-**4a** and KPC-2:(*S*)-**4a** complexes. 112

Table S3. Data collection and refinement statistics for KPC-2:(*S*)-**4a** and OXA-48:(*R*)-**4a** crystal structures. 113

References 114

Supplementary Experiment (SE) Section

SE. 1 Chemistry

(1) General Experimental Methods

1,4-Dioxane was freshly distilled from CaH_2 under N_2. Unless otherwise specified, all reagents and solvents were from commercial sources and used as received. ^1H (400 MHz) and ^{13}C (100 MHz) NMR spectra were recorded using a Bruker Avance 400 spectrometer in $CDCl_3$, CD_3OD or DMSO-d_6. Coupling constant (*J*) values are given in Hz. Multiplicities are designated by the following abbreviations: s, singlet; d, doublet; t, triplet; q, quartet; br, broad; m, multiplet. Products were purified by flash column chromatography on silica gel from Qingdao Haiyang Chemical Co., Ltd. Optical rotations were measured using a Rudolph AUTOPOL I Automatic Polarimeter. HRMS were recorded using a Bruker microTOF spectrometer. HPLC analyses were performed with Daicel Chiralpak AD column (25 cm×4.6 mm×5 μm). Catalysts **C1-C13** were synthesized according to the reported procedures[1-3] or obtained from commercial sources.

(2) General Experimental Procedure for the Synthesis of Substituted 2-Formylphenylboronic Acids 1a-1t[4]

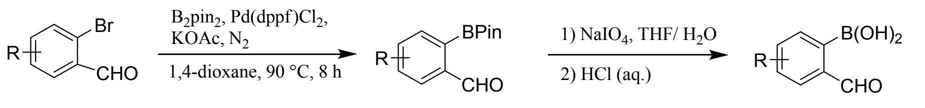

6b, **6d**, **6e**, **6h**, **6i**, **6l**

7b, **7d**, **7e**, **7h**, **7i**, **7l**

1b, R = 4-Me
1d, R = 4-OEt
1e, R = 4-O*i*Pr
1h, R = 5-Me
1i, R = 5-OMe
1l, R = 4,5-OMe$_2$

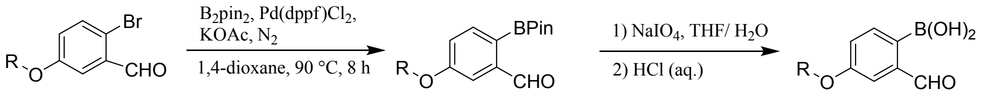

6m, **6n**, **6o**, **6p**

7m, **7n**, **7o**, **7p**

1m, R = *p*-Tolyl
1n, R = *m*-Tolyl
1o, R = *p*-CF$_3$Ph
1p, R = Bn

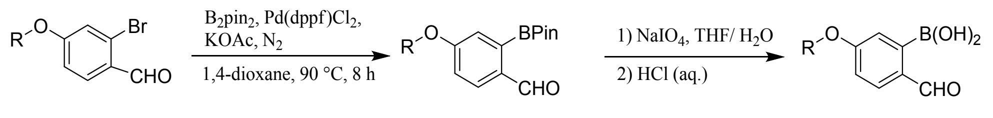

6q, **6r**, **6s**, **6t**

7q, **7r**, **7s**, **7t**

1q, R = *p*-OMePh
1r, R = *p*-NO_2Ph
1s, R = *p*-ClPh
1t, R = Bn

Syntheses of **7a-7t**: To a round bottom flask (25 mL) were added bis(pinacolato)diboron (1.22 g, 4.8 mmol, 1.2 equiv.), substrate **6a-6t** (4.0 mmol, 1.0 equiv.), $PdCl_2$(dppf) (88 mg, 0.12 mmol, 3 mol %) and KOAc (1.18 g, 12 mmol, 3.0 equiv.). The mixture was closed by a septum, then purged several times with argon gas; dried 1,4-dioxane (15 mL) was then added to the reaction mixture, which was stirred at 80 °C for 8 h. The crude reaction mixture was diluted with ethyl acetate, then extracted with saturated NaCl solution. The combined organic extracts were dried over Na_2SO_4, then concentrated in vacuo. The residue was purified by flash column chromatography using 40-60 petroleum ether and ethyl acetate as eluent to give **7a-7t** in 60%-85% yield.

Syntheses of 1a-1t：To a solution of **7a-7t** (4.0 mol, 1.0 equiv.) in THF:H_2O = 10:1 (32 mL) was added $NaIO_4$ (6.0 mmol, 1.5 equiv.). The mixture was stirred at room temperature for 20 minutes, until homogeneous; HCl (1.0 M in water, 10 mL) was then added and the mixture was stirred for 2 h. The resulting mixture was extracted with DCM (CH_2Cl_2) (3 × 50 mL), dried over with anhydrous Na_2SO_4, then concentrated in vacuo. The residue was purified by flash column chromatography by using 40-60 petroleum ether and ethyl acetate as eluent to give **1a-1t** in 41-68% yield. (**1a**, **1c, 1f, 1g, 1j, 1k** were obtained from commercial sources).

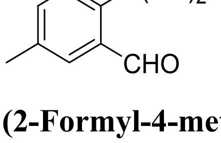

(2-Formyl-4-methylphenyl) boronic acid (1b): 45% yield over two steps. ^{1}H NMR (400 MHz, Chloroform-*d*) δ 9.88 (s, 1H), 8.20 (d, *J* = 7.6 Hz, 1H), 7.72 (s, 1H), 7.53 – 7.47 (m, 3H), 2.49 (s, 3H).

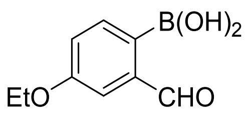

(4-Ethoxy-2-formylphenyl) boronic acid (1d): 47% yield over two steps. ^{1}H NMR (400 MHz, Chloroform-*d*) δ 9.86 (s, 1H), 8.22 (d, *J* = 8.3 Hz, 1H), 7.42 (d, *J* = 2.7 Hz, 1H), 7.17 (d, *J* = 2.7 Hz, 2H), 4.15 (q, *J* = 7.0 Hz, 2H), 1.48 (t, *J* = 7.0 Hz, 3H).

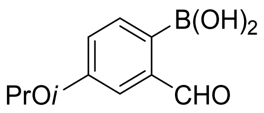

(2-Formyl-4-isopropoxyphenyl)boronic acid (1e): 38% yield over two steps. ^{1}H NMR (400 MHz, Chloroform-*d*) δ 9.84 (s, 1H), 8.23 (d, *J* = 8.3 Hz, 1H), 7.41 (d, *J* = 2.6 Hz, 1H), 7.17 (dd, *J* = 8.3, 2.6 Hz, 1H), 4.75 – 4.66 (m, 1H), 1.40 (d, *J* = 6.0 Hz, 6H).

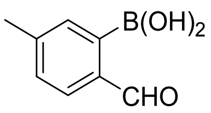

(2-Formyl-5-methylphenyl) boronic acid (1h): 56% yield over two steps. ^{1}H NMR (400 MHz, Chloroform-*d*) δ 9.78 (s, 1H), 8.05 (s, 1H), 7.74 (d, *J* = 7.7 Hz, 1H), 7.51 – 7.42 (m, 2H), 2.42 (s, 3H).

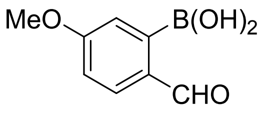

(2-Formyl-5-methoxyphenyl) boronic acid (1i): 50% yield over two steps. ^{1}H NMR (400 MHz, Chloroform-*d*) δ 9.61 (s, 1H), 7.96 (d, *J* = 8.8 Hz, 1H), 7.01 (dd, *J* = 8.8, 2.5 Hz, 1H), 6.75 (d, *J* = 2.5 Hz, 1H), 3.83 (s, 3H).

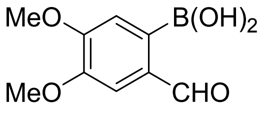

(2-Formyl-4,5-dimethoxyphenyl) boronic acid (1l): 45% yield over two steps ^{1}H NMR (400 MHz, DMSO-d_6) δ 10.02 (s, 1H), 7.14 (s, 1H), 6.56 (s, 1H), 3.83 (s, 3H), 3.73 (s, 3H).

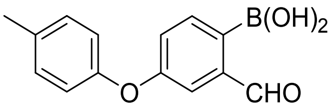

(2-Formyl-4-(p-tolyloxy) phenyl) boronic acid (1m): 35% yield over two steps. ^{1}H NMR (400 MHz, Chloroform-*d*) δ 9.73 (s, 1H), 8.16 (d, *J* = 8.3 Hz, 1H), 7.40 (d, *J* = 2.5 Hz, 1H), 7.22 (s, 1H), 7.20 – 7.16 (m, 1H), 7.16 (s, 1H), 7.14 – 7.13 (m, 1H), 6.92 (s, 1H), 2.31 (s, 3H).

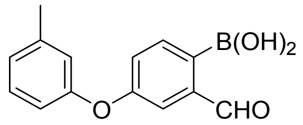

(2-Formyl-4-(m-tolyloxy) phenyl) boronic acid (1n): 28% yield over two steps. ^{1}H NMR (400 MHz, Chloroform-*d*) δ 9.82 (s, 1H), 8.24 (d, *J* = 8.3 Hz, 1H), 7.50 (d, *J* = 2.6 Hz, 1H), 7.34 – 7.23 (m, 3H), 7.04 (d, *J* = 7.5 Hz, 1H), 6.90 (s, 1H), 2.38 (s, 3H).

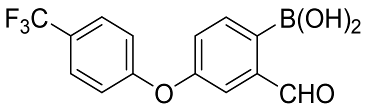

(2-Formyl-4-(4-(trifluoromethyl) phenoxy) phenyl) boronic acid (1o): 37% yield over two steps ^{1}H NMR (400 MHz, Chloroform-*d*) δ 9.86 (s, 1H), 8.31 (d, *J* = 8.3 Hz, 1H), 7.67 (d, *J* = 8.4 Hz, 2H), 7.57 (d, *J* = 2.6 Hz, 1H), 7.33 (dd, *J* = 8.3, 2.5 Hz, 1H), 7.15 (d, *J* = 8.5 Hz, 2H).

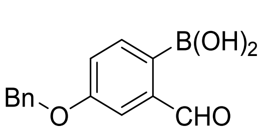

(4-(Benzyloxy)-2-formylphenyl) boronic acid (1p): 52% yield over two steps. ^{1}H NMR (400 MHz, Chloroform-*d*) δ 9.88 (s, 1H), 8.26 (d, *J* = 8.4 Hz, 1H), 7.53 (d, *J* = 2.7 Hz, 1H), 7.50 – 7.36 (m, 5H), 7.29 (d, *J* = 5.6 Hz, 1H), 5.21 (s, 2H).

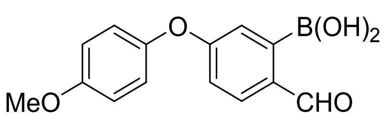

(2-Formyl-5-(4-methoxyphenoxy) phenyl) boronic acid (1q): 34% yield over two steps. ^{1}H NMR (400 MHz, Chloroform-*d*) δ 9.77 (s, 1H), 7.85 (d, *J* = 2.7 Hz, 1H), 7.82 (d, *J* = 8.5 Hz, 1H), 7.08 (dd, *J* = 8.5, 2.7 Hz, 1H), 7.06 – 7.02 (m, 2H), 6.96 (d, *J* = 2.3 Hz, 1H), 6.94 (d, *J* = 2.3 Hz, 1H), 3.84 (s, 3H).

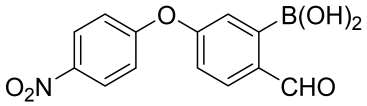

(2-Formyl-5-(3-nitrophenoxy) phenyl) boronic acid (1r): 27% yield over two steps. ^{1}H NMR (400 MHz, DMSO-d_6) δ 10.11 (s, 1H), 8.36 (s, 2H), 8.34 – 8.28 (m, 2H), 7.99 (d, *J* = 8.3 Hz, 1H), 7.27 (d, *J* = 2.4 Hz, 2H).

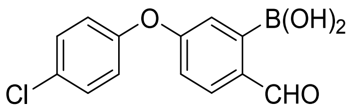

(5-(4-Chlorophenoxy)-2-formylphenyl) boronic acid (1s): 45% yield over two steps. ^{1}H NMR (400 MHz, Chloroform-*d*) δ 9.81 (s, 1H), 7.89 – 7.81 (m, 2H), 7.41 – 7.38 (m, 2H), 7.15 (dd, *J* = 8.4, 2.7 Hz, 1H), 7.08 – 7.02 (m, 2H).

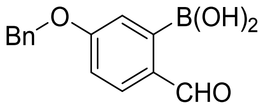

(5-(Benzyloxy)-2-formylphenyl) boronic acid (1t): 47% yield over two steps. ^{1}H NMR (400 MHz, Chloroform-*d*) δ 9.68 (s, 1H), 7.86 (s, 1H), 7.77 (d, *J* = 8.7 Hz, 1H), 7.56 (s, 1H), 7.36 (dd, *J* = 13.2, 7.6 Hz, 2H), 7.33 – 7.25 (m, 1H), 7.11 (d, *J* = 8.6 Hz, 1H), 5.15 (s, 2H).

(3) Optimization of Reaction Conditions.[a]

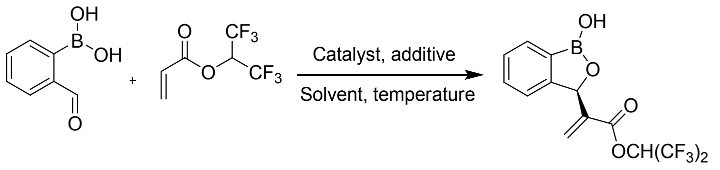

1a **2** **3a**

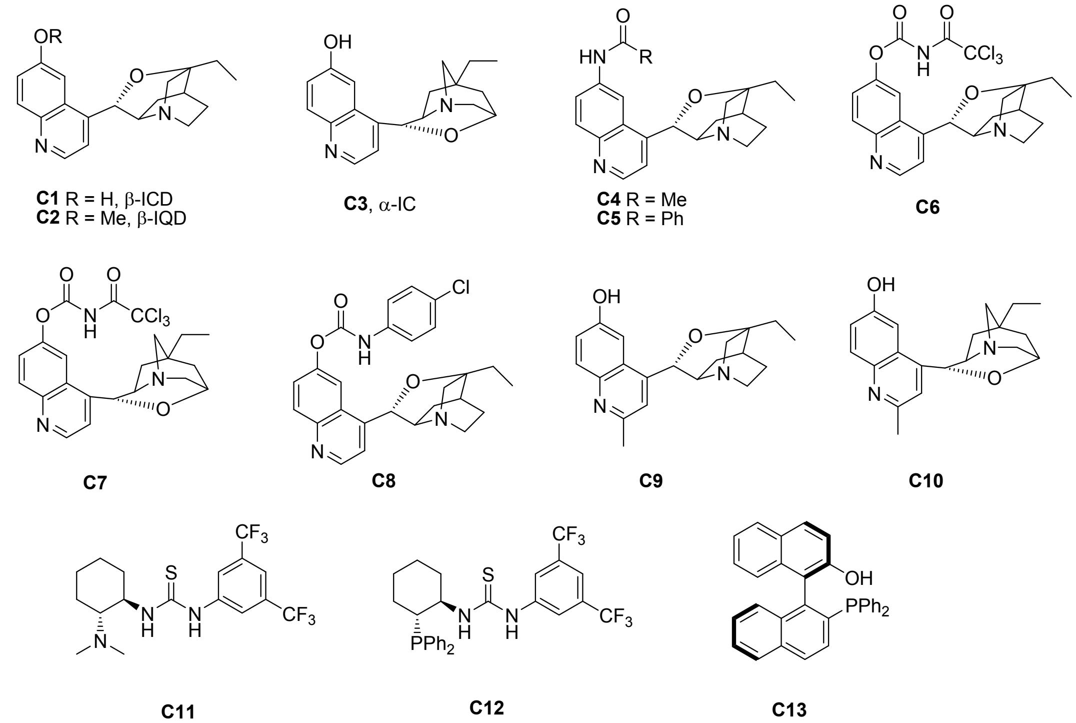

Entry	Cat.	**1a**:**2**	[M]: **1a**	Additive (equiv.)	Tem. (ºC)	Solvent	Time (h)	Yield (%)[b]	Ee (%)[c]
1	**C1**	1:3	0.1	-	rt	THF	29	45	27
2	**C1**	1:3	0.1	4Å MS (1.0)	rt	THF	43	13	6
3	**C1**	1:3	0.1	2-Naphthol (1.0)	rt	THF	36	nr	/
4	**C1**	1:3	0.1	Et_3N (1.0)	rt	THF	10	84	<5
5	**C1**	1:3	0.1	DIPEA (1.0)	rt	THF	24	77	21
6	**C1**	1:3	0.1	Na_2CO_3 (1.0)	rt	THF	44	20	25
7	**C1**	1:3	0.1	KF (1.0)	rt	THF	61	12	23
8	**C1**	1:3	0.1	Na_3PO_4 (1.0)	rt	THF	37	33	17
9	**C1**	1:3	0.1	$NaHCO_3$ (1.0)	rt	THF	46	50	41
10	**C1**	1:3	0.1	Cs_2CO_3 (1.0)	rt	THF	22	43	13
11	**C1**	1:3	0.1	MgO (1.0)	rt	THF	19	88	19
12	**C1**	1:3	0.1	CaO (1.0)	rt	THF	19	57	45
13	**C1**	1:3	0.1	$Al(OH)_3$ (1.0)	rt	THF	7	88	64
14	**C1**	1:3	0.1	Al_2O_3 (1.0)	rt	THF	17	82	70
15	**C1**	1:3	0.1	Al_2O_3 (1.0)	0	THF	54	46	78
16	**C1**	1:3	0.1	Al_2O_3 (1.0)	-10	THF	72	38	76
17	**C1**	1:3	0.1	Al_2O_3 (1.0)	-30	THF	72	nr	/
18	**C2**	1:3	0.1	Al_2O_3 (1.0)	rt	THF	36	28	9

19	**C3**	1:3	0.1	Al_2O_3 (1.0)	rt	THF	48	49	-37
20	**C3**	1:3	0.1	Na_2CO_3 (1.0)	rt	THF	68	68	-8
21	**C3**	1:3	0.1	KF (1.0)	rt	THF	28	48	-15
22	**C3**	1:3	0.1	$Al(OH)_3$ (1.0)	rt	THF	48	81	-13
23	**C4**	1:3	0.1	Al_2O_3 (1.0)	rt	THF	50	70	48
24	**C5**	1:3	0.1	Al_2O_3 (1.0)	rt	THF	48	46	37
25	**C6**	1:3	0.1	Al_2O_3 (1.0)	rt	THF	28	83	74
26	**C7**	1:3	0.1	Al_2O_3 (1.0)	rt	THF	29	90	-50
27	**C8**	1:3	0.1	Al_2O_3 (1.0)	rt	THF	41	87	24
28	**C9**	1:3	0.1	Al_2O_3 (1.0)	rt	THF	37	84	64
29	**C10**	1:3	0.1	Al_2O_3 (1.0)	rt	THF	90	64	-40
30	**C11**	1:3	0.1	Al_2O_3 (1.0)	rt	THF	72	nr	/
31	**C12**	1:3	0.1	Al_2O_3 (1.0)	rt	THF	72	<5	nd
32	**C13**	1:3	0.1	Al_2O_3 (1.0)	rt	THF	90	<5	nd
33	**C6**	1:3	0.1	Al_2O_3 (2.0)	rt	THF	28	85	73
34	**C6**	1:3	0.1	Al_2O_3 (4.0)	rt	THF	28	87	76
35	**C6**	1:3	0.1	Al_2O_3 (6.0)	rt	THF	20	75	69
36	**C6**	1:3	0.1	Al_2O_3 (4.0)	rt	DCM	42	30	6
37	**C6**	1:3	0.1	Al_2O_3 (4.0)	rt	CH_3CN	42	30	26
38	**C6**	1:3	0.1	Al_2O_3 (4.0)	rt	DMF	120	68	65
39	**C6**	1:3	0.1	Al_2O_3 (4.0)	rt	Et_2O	120	nr	/
40	**C6**	1:3	0.1	Al_2O_3 (4.0)	rt	EA	47	25	55
41	**C6**	1:3	0.1	Al_2O_3 (4.0)	rt	Toluene	60	28	27
42	**C6**	1:3	0.1	Al_2O_3 (4.0)	rt	Dioxane	16	90	85
43	**C6**	1:5	0.05	Al_2O_3 (4.0)	rt	Dioxane	18	86	88
**44**	**C6**	**1:5**	**0.025**	**Al_2O_3 (4.0)**	**rt**	**Dioxane**	**24**	**85**	**90**
45[d]	**C6**	1:5	0.025	Al_2O_3 (4.0)	rt	Dioxane	36	63	70
46	**C7**	1:5	0.025	Al_2O_3 (4.0)	rt	Dioxane	72	80	-47
**47**	**C7**	**1:5**	**0.1**	**Al_2O_3 (4.0)**	**0**	**Dioxane**	**96**	**90**	**-60**

[a]Standard reaction conditions: **1a** (0.1 mmol), **2** (0.3 or 0.5 mmol), catalyst (0.01 mmol), additive, solvent, rt. [b]Isolated yields of **3a**. [c]ee was determined by chiral HPLC analysis. [d]5 mol% of **C6** was used.

(4) General Procedure for the Catalytic Asymmetric MBH Cascade Reaction.

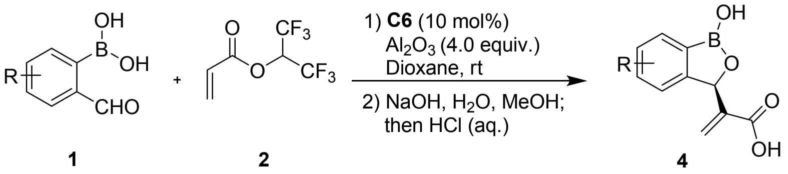

To a mixture of substituted 2-formylphenylboronic acids **1** (0.1 mmol), catalyst **C6** (0.01 mmol), Al_2O_3 (0.4 mmol) in dioxane (4.0 mL) was added hexafluoroisopropyl acrylate **2** (HFIPA, 0.5 mmol, 53 μL) at room temperature. The reaction was stirred for the specified time until no remaining starting material **1** was observed by TLC. The reaction mixture was then filtered and concentrated in vacuo; the resulting residue was subsequently dissolved in MeOH (2.0 mL) and an NaOH solution (12 mg, 0.3 mmol dissolved in 0.5 mL water) was added dropwise. The reaction mixture was stirred at room temperature for 3 hours, then acidified with hydrochloric acid. The mixture was extracted with ethyl acetate; the combined extracts were was dried over sodium sulfate, filtered and evaporated under vacuum. The crude product was purified by chromatography to give **4**.

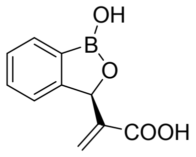

(*R*)-2-(1-Hydroxy-1,3-dihydrobenzo[*c*][1,2]oxaborol-3-yl) acrylic acid (4a): white solid, 80% yield; mp = 89.8-91.9 °C. $[\alpha]_D^{20}$: +94.50 (*c* = 0.20 in MeOH); 90% ee (96% ee after recrystallization from EA) determined by chiral HPLC (Chiralpak AD), *n*-hexane/*i*-PrOH /CH_3COOH = 980/19/1, 1.0 mL/ min, λ = 254 nm; *t* (minor) = 6.249 min, *t* (major) = 7.381 min; ^1H NMR (400 MHz, Methanol-d_4): δ = 7.64 (d, *J* = 7.4 Hz, 1H), 7.42 (t, *J* = 7.4 Hz, 1H), 7.38 (d, *J* = 7.4 Hz, 1H), 7.32 (t, *J* = 7.4 Hz, 1H), 6.25 (s, 1H), 6.05 (s, 1H), 5.77 (s, 1H); ^{13}C NMR (100 MHz, Methanol-d_4): δ = 167.5, 155.6, 140.9, 130.7, 130.6, 129.9, 127.2, 124.4, 121.6, 79.7. ESI HR-MS: *m/z* = 203.9880, calcd. for $[C_{10}H_8BO_4\text{-}H]^-$:203.0521, found:203.0522. (*S*)-**4a**: white solid, 88% yield; $[\alpha]_D^{20}$: -63.50 (*c* = 0.20 in MeOH); 60% ee (99.5% ee after recrystallization from EA) determined by chiral HPLC (Chiralpak AD), *n*-hexane/*i*-PrOH/CH_3COOH = 980/19/1, 1.0 mL/ min, λ = 254 nm; *t* (minor) =7.593 min, *t* (major) = 6.461 min

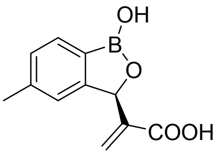

(*R*)-2-(1-Hydroxy-5-methyl-1,3-dihydrobenzo[*c*][1,2]oxaborol-3-yl)acrylic acid (4b): semi-solid, 80% yield; $[\alpha]_D^{20}$: +66.03 (c = 0.12 in MeOH); 89% ee, determined by chiral HPLC (Chiralpak AD), *n*-hexane/ *i*-PrOH/CH_3COOH = 980/19/1, 1.0 mL/ min, λ = 254 nm; *t* (minor) = 6.122 min, *t* (major) = 7.447 min; ^1H NMR (400 MHz, DMSO-d_6): δ =12.78 (s, 1H), 9.23 (s, 1H), 7.60 (d, J = 7.4 Hz, 1H), 7.23 – 7.09 (m, 2H), 6.12 (s, 1H), 5.90 (s, 1H), 5.71 (s, 1H), 2.33 (s, 3H); ^{13}C NMR (100 MHz, DMSO-d_6): δ =167.4, 156.4, 141.7, 145.0, 130.9, 128.8, 125.1, 122.5, 78.8, 49.1, 22.0; ESI HR-MS: *m/z* = 218.0150, calcd. for $[C_{11}H_{10}BO_4\text{-}H]^-$: 217.0678, found: 217.0679.

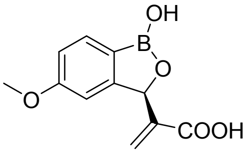

(*R*)-2-(1-Hydroxy-5-methoxy-1,3-dihydrobenzo[*c*][1,2]oxaborol-3-yl) acrylic acid (4c): white solid, 85% yield; mp = 116.2-119.6 ℃. $[\alpha]_D^{20}$: +28.00 (c = 0.10 in MeOH); 90% ee (99% ee after recrystallization from EA), determined by chiral HPLC (Chiralpak AD), *n*-hexane/ *i*-PrOH/CH_3COOH = 980/19/1, 1.0 mL/ min, λ = 254 nm; *t* (minor) = 8.687 min, *t* (major) = 10.625 min; ^1H NMR (400 MHz, Methanol-d_4): δ = 7.45 (d, J = 8.1 Hz, 1H), 6.85 – 6.78 (m, 2H), 6.16 (s, 1H), 5.90 (s, 1H), 5.67 (s, 1H), 3.69 (s, 3H); ^{13}C NMR (100 MHz, Methanol-d_4): δ = 167.7, 162.6, 158.1, 141.1, 131.2, 124.2, 114.4, 106.4, 79.2, 54.3; ESI HR-MS: *m/z* = 234.0140, calcd. for $[C_{11}H_{10}BO_5\text{-}H]^-$: 233.0627, found: 233.0617. (*S*)-**4c**: white solid, 80% yield; $[\alpha]_D^{20}$: -17.80 (c = 0.20 in MeOH); 52% ee (96% ee after recrystallization from EA) determined by chiral HPLC (Chiralpak AD), *n*-hexane/*i*-PrOH/CH_3COOH = 980/19/1, 1.0 mL/ min, λ = 254 nm; *t* (minor) = 10.391 min, *t* (major) = 8.427 min.

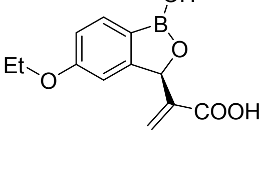

(*R*)-2-(5-Ethoxy-1-hydroxy-1,3-dihydrobenzo[*c*][1,2]oxaborol-3-yl) acrylic acid (4d): semi-solid, 82% yield; $[\alpha]_D^{20}$: +37.23 (c = 0.13 in MeOH); 86% ee, determined by chiral HPLC (Chiralpak AD), *n*-hexane/*i*-PrOH/CH_3COOH = 980/19/1, 1.0 mL/min, λ = 254 nm; *t* (minor) = 7.646 min, *t* (major) = 11.231 min; ^1H NMR (400 MHz, Methanol-d_4) δ =7.54 (d, J = 8.1 Hz, 1H),

6.97 – 6.80 (m, 2H), 6.25 (s, 1H), 5.99 (s, 1H), 5.77 (s, 1H), 4.02 (q, J = 7.0 Hz, 2H), 1.37 (t, J = 7.0 Hz, 3H); ^{13}C NMR (100 MHz, Methanol-d_4): δ = 167.7, 161.9, 158.1, 141.1, 131.2, 124.2, 121.2, 114.8, 107.0, 79.2, 63.2, 13.6; ESI HR-MS: m/z = 248.0410, calcd. for $[C_{12}H_{13}BO_5-H]^-$: 247.0783, found: 247.0780.

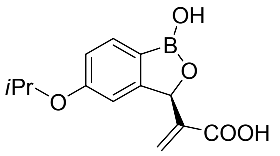

(*R*)-2-(1-Hydroxy-5-isopropoxy-1,3-dihydrobenzo[*c*][1,2]oxaborol-3-yl) acrylic acid (4e): semi-solid, 71% yield; $[\alpha]_D^{20}$: +31.30 (c = 0.09 in MeOH); 82% ee, determined by chiral HPLC (Chiralpak AD), n-hexane/i-PrOH/CH_3COOH = 980/19/1, 1.0 mL/ min, λ = 254 nm; t (minor) = 6.481 min, t (major) = 9.261 min; ^{1}H NMR (400 MHz, Methanol-d_4) δ =7.54 (d, J = 8.1 Hz, 1H), 6.91 (d, J = 2.2 Hz, 1H), 6.87 (dd, J = 8.1, 2.2 Hz, 1H), 6.25 (s, 1H), 5.99 (s, 1H), 5.77 (s, 1H), 1.30 (t, J = 6.0 Hz, 9H); ^{13}C NMR (100 MHz, Methanol-d_4): δ =167.7, 160.8, 158.1, 141.1, 131.3, 124.0, 115.9, 108.2, 79.1, 69.6, 29.3, 20.9, 20.8; ESI HR-MS: m/z = 262.0680, calcd. for $[C_{13}H_{15}BO_5-H]^-$: 261.0959, found: 261.0962.

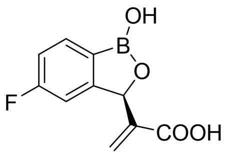

(*R*)-2-(5-Fluoro-1-hydroxy-1,3-dihydrobenzo[*c*][1,2]oxaborol-3-yl) acrylic acid (4f): white solid, 86% yield; mp = 91.9-93.4 ℃. $[\alpha]_D^{20}$: +50.01 (c= 0.09 in MeOH); 82% ee (90% ee after recrystallization from EA), determined by chiral HPLC (Chiralpak AD), n-hexane/ i-PrOH/CH_3COOH = 980/19/1, 1.0 mL/ min, λ = 254 nm; t (minor) = 6.004 min, t (major) = 7.41 min; ^{1}H NMR (400 MHz, Methanol-d_4): δ =7.55 (dd, J = 8.1, 5.6 Hz, 1H), 7.06 – 6.93 (m, 2H), 6.19 (s, 1H), 5.93 (s, 1H), 5.73 (s, 1H); ^{13}C NMR (100 MHz, Methanol-d_4): δ =167.3, 166.3, 163.8, 158.5, 158.4, 140.4, 132.0, 131.9, 124.7, 114.9, 114.7, 108.9, 108.7, 79.3, 79.3; ESI HR-MS: m/z = 221.9784, calcd. for $[C_{10}H_7BFO_4-H]^-$: 221.0427, found: 221.0434. (*S*)-**4f**: white solid, 79% yield; $[\alpha]_D^{20}$: -28.65 (c = 0.20 in MeOH); 48% ee (90% ee after recrystallization from EA) determined by chiral HPLC (Chiralpak AD), n-hexane/i-PrOH/CH_3COOH = 980/19/1, 1.0 mL/ min, λ = 254 nm; t (minor) = 7.475 min, t (major) = 5.981 min.

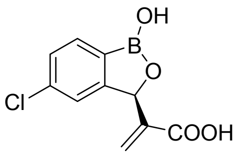

(*R*)-2-(5-Chloro-1-hydroxy-1,3-dihydrobenzo[*c*][1,2]oxaborol-3-yl) acrylic acid (4g): semi-solid, 70% yield; $[\alpha]_D^{20}$: +45.00 (*c* = 0.07 in MeOH), 75% ee, determined by chiral HPLC (Chiralpak AD), *n*-hexane/ *i*-PrOH/CH_3COOH = 980/19/1, 1.0 mL/ min, λ = 254 nm; *t* (minor) = 6.422 min, *t* (major) = 7.594 min; ^1H NMR (400 MHz, Methanol-d_4): δ = 7.50 (d, *J* = 7.8 Hz, 1H), 7.32 (d, *J* = 1.8 Hz, 1H), 7.24 (dd, *J* = 7.8, 1.8 Hz, 1H), 6.20 (s, 1H), 5.93 (s, 1H), 5.74 (s, 1H); ^{13}C NMR (100 MHz, Methanol-d_4): δ = 167.3, 157.5, 140.2, 136.9, 131.2, 127.6, 124.9, 122.0, 79.4; ESI HR-MS: *m/z* = 238.4300, calcd. for $[C_{10}H_7BClO_4\text{-}H]^-$: 237.0131, found: 237.0132.

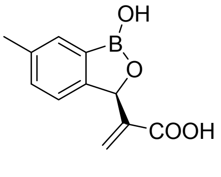

(*R*)-2-(1-Hydroxy-6-methyl-1,3-dihydrobenzo[*c*][1,2]oxaborol-3-yl) acrylic acid (4h): semi-solid, 84% yield; $[\alpha]_D^{20}$: +19.24 (*c* = 0.11 in MeOH); 88% ee), determined by chiral HPLC (Chiralpak AD), *n*-hexane/ *i*-PrOH/CH_3COOH = 980/19/1, 1.0 mL/ min, λ = 254 nm; *t* (minor) = 6.534 min, *t* (major) = 6.954 min; ^1H NMR (400 MHz, DMSO-d_6): δ =12.81 (s, 1H), 9.29 (s, 1H), 7.52 (s, 1H), 7.25 (q, *J* = 8.1 Hz, 2H), 6.11 (s, 1H), 5.91 (s, 1H), 5.70 (s, 1H), 2.34 (s, 3H); ^{13}C NMR (100 MHz, DMSO-d_6): δ =167.4, 153.2, 152.3, 141.8, 136.8, 132.2, 131.3, 124.8, 122.0, 78.8, 21.3; ESI HR-MS: *m/z* = 218.0150, calcd. for $[C_{11}H_{10}BO_4\text{-}H]^-$: 217.0678, found: 217.0669.

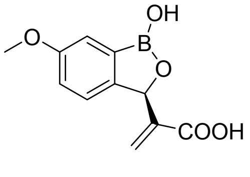

(*R*)-2-(1-Hydroxy-6-methoxy-1,3-dihydrobenzo[*c*][1,2]oxaborol-3-yl) acrylic acid (4i): semi-solid, 72% yield; $[\alpha]_D^{20}$: +35.45 (*c* = 0.11 in MeOH); 84% ee, determined by chiral HPLC (Chiralpak AD), *n*-hexane/ *i*-PrOH/CH_3COOH = 980/19/1, 1.0 mL/ min, λ = 254 nm; *t* (minor) = 8.659 min, *t* (major) = 9.343 min; ^1H NMR (400 MHz, DMSO-d_6): δ =12.82 (s, 1H), 9.30 (s, 1H), 7.29 – 7.12 (m, 2H), 7.03 (dd, *J* = 8.4, 2.6 Hz, 1H), 6.09 (s, 1H), 5.89 (s, 1H), 5.69 (s, 1H), 3.77 (s, 3H); ^{13}C NMR (100 MHz, DMSO-d_6): δ =167.8, 159.6, 148.3, 142.2,

125.0, 123.6, 118.8, 114.6, 78.9, 56.0; ESI HR-MS: *m/z* = 234.0140, calcd. for $[C_{11}H_{10}BO_5\text{-}H]^-$: 233.0627, found: 233.0634.

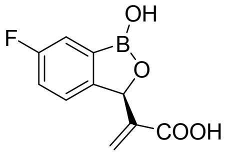

(*R*)-2-(6-Fluoro-1-hydroxy-1,3-dihydrobenzo[*c*][1,2]oxaborol-3-yl) acrylic acid (4j): white solid, 91% yield; mp = 118.7-122.4 °C. $[\alpha]_D^{20}$: +68.54 (*c* = 0.15 in MeOH), 81% ee (96% ee after recrystallization from EA), determined by chiral HPLC (Chiralpak AD), *n*-hexane/ *i*-PrOH/CH_3COOH = 980/19/1, 1.0 mL/ min, λ = 254 nm; *t* (minor) = 6.385 min, *t* (major) = 6.941 min; ^1H NMR (400 MHz, Methanol-d_4): δ = 7.30 (dd, *J* = 8.4, 4.5 Hz, 1H), 7.18 (dd, *J* = 8.4, 2.5 Hz, 1H), 7.06 (ddd, *J* = 9.3, 8.4, 2.5 Hz, 1H), 6.17 (s, 1H), 5.93 (s, 1H), 5.71 (s, 1H); ^{13}C NMR (100 MHz, Methanol-d_4): δ = 167.7, 163.8, 162.2, 151.4, 141.0, 125.0, 123.91, 123.85, 118.3, 118.1, 115.7, 115.5, 79.9; ESI HR-MS: *m/z* = 221.9784, calcd. for $[C_{10}H_7BFO_4\text{-}H]^-$: 221.0427, found: 221.0421. (*S*)-**4j**: white solid, 65% yield; $[\alpha]_D^{20}$: -47.60 (*c* = 0.20 in MeOH); 57% ee (95% ee after recrystallization from EA) determined by chiral HPLC (Chiralpak AD), *n*-hexane/*i*-PrOH/CH_3COOH = 980/19/1, 1.0 mL/ min, λ = 254 nm; *t* (minor) = 7.053 min, *t* (major) = 6.373 min.

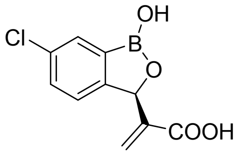

(*R*)-2-(6-Chloro-1-hydroxy-1,3-dihydrobenzo[*c*][1,2]oxaborol-3-yl) acrylic acid (4k): semi-solid, 81% yield; $[\alpha]_D^{20}$: +51.11 (*c* = 0.09 in MeOH); 78% ee, determined by chiral HPLC (Chiralpak AD), *n*-hexane/ *i*-PrOH/CH_3COOH = 980/19/1, 1.0 mL/ min, λ = 254 nm; *t* (major) = 4.750 min, *t* (minor) = 5.220 min; ^1H NMR (400 MHz, Methanol-d_4): δ = 7.48 (d, *J* = 2.0 Hz, 1H), 7.38 – 7.24 (m, 2H), 6.19 (s, 1H), 5.93 (s, 1H), 5.73 (s, 1H); ^{13}C NMR (100 MHz, Methanol-d_4): δ = 167.2, 153.9, 140.3, 133.5, 130.6, 129.5, 129.4, 125.0, 123.3, 79.7; ESI HR-MS: *m/z* = 238.4300, calcd. for $[C_{10}H_7BClO_4\text{-}H]^-$: 237.0131, found: 237.0138.

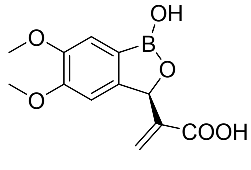

(*R*)-2-(1-Hydroxy-5,6-dimethoxy-1,3-dihydrobenzo[*c*][1,2]oxaborol-3-yl) acrylic acid (4l): semi-

solid, 84% yield; $[\alpha]_D^{20}$: +40.56 (*c* = 0.23 in MeOH); 82% ee, determined by chiral HPLC (Chiralpak AD), *n*-hexane/*i*-PrOH/CH_3COOH = 980/19/1, 1.0 mL/ min, λ = 254 nm; *t* (minor) = 12.348 min, *t* (major) = 15.173 min; ^1H NMR (400 MHz, DMSO-d_6): δ = 12.79 (s, 1H), 9.10 (s, 1H), 7.21 (s, 1H), 6.90 (s, 1H), 6.11 (s, 1H), 5.85 (s, 1H), 5.68 (s, 1H), 3.77 (s, 3H), 3.74 (s, 3H); ^{13}C NMR (100 MHz, DMSO-d_6): δ = 167.9, 152.4, 149.8, 149.5, 142.2, 125.3, 122.2, 112.8, 105.7, 78.8, 56.2, 56.1; ESI HR-MS: *m/z* = 264.0400, calcd. for $[C_{12}H_{12}BO_6\text{-}H]^-$: 263.0732, found: 263.0730.

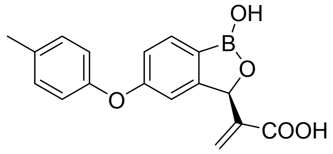

(*R*)-2-(1-Hydroxy-5-(p-tolyloxy)-1,3-dihydrobenzo[*c*][1,2]oxaborol-3-yl) acrylic acid (4m): semi-solid, 75% yield; $[\alpha]_D^{20}$: +20.24 (c = 0.25 in MeOH); 85% ee, determined by chiral HPLC (Chiralpak AD), *n*-hexane/*i*-PrOH/CH_3COOH = 980/19/1, 1.0 mL/ min, λ = 254 nm; *t* (minor) = 8.585 min, *t* (major) = 12.019 min; ^1H NMR (400 MHz, Methanol-d_4): δ = 7.59 (d, *J* = 8.0 Hz, 1H), 7.17 (d, *J* = 8.0 Hz, 2H), 6.96 – 6.86 (m, 4H), 6.24 (s, 1H), 5.98 (s, 1H), 5.77 (s, 1H), 2.32 (s, 3H); ^{13}C NMR (100 MHz, Methanol-d_4): δ =167.5, 161.0, 158.1, 153.9, 140.8, 133.5, 131.5, 130.1, 124.3, 119.3, 117.3, 110.6, 79.2, 29.3, 19.4; ESI HR-MS: *m/z* = 310.1120, calcd. for $[C_{17}H_{15}BO_5\text{-}H]^-$: 309.0940, found: 309.0936.

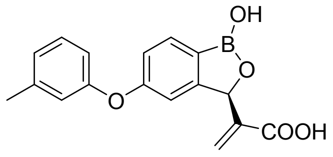

(*R*)-2-(1-Hydroxy-5-(m-tolyloxy)-1,3-dihydrobenzo[*c*][1,2]oxaborol-3-yl) acrylic acid (4n): semi-solid, 80% yield; $[\alpha]_D^{20}$: +17.82 (c = 0.09 in MeOH); 97% ee, determined by chiral HPLC (Chiralpak AD), *n*-hexane/*i*-PrOH/CH_3COOH = 980/19/1, 1.0 mL/ min, λ = 254 nm; *t* (minor) = 7.672 min, *t* (major) = 11.012 min; ^1H NMR (400 MHz, Methanol-d_4): δ = 7.60 (d, *J* = 8.0 Hz, 1H), 7.23 (t, *J* = 7.8 Hz, 1H), 6.98 – 6.91 (m, 3H), 6.83 (t, *J* = 2.4 Hz, 1H), 6.78 (dd, *J* = 8.0, 2.4 Hz, 1H), 6.24 (s, 1H), 6.00 (s, 1H), 5.79 (s, 1H), 2.31 (s, 3H); ^{13}C NMR (100 MHz, Methanol-d_4): δ =167.5, 160.6, 158.1, 156.4, 140.8, 140.0, 131.5, 129.3, 124.4, 124.2, 119.8, 117.7, 116.2, 111.1, 79.3, 20.0; ESI HR-MS: *m/z* = 310.1120, calcd. for $[C_{17}H_{15}BO_5\text{-}H]^-$: 309.0940, found: 309.0937.

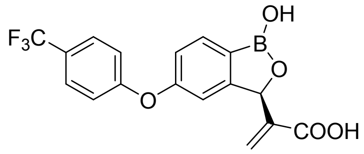

(*R*)-2-(1-Hydroxy-5-(4-(trifluoromethyl)phenoxy)-1,3-dihydrobenzo[*c*][1,2] oxaborol-3-yl) acrylic acid (4o): semi-solid, 72% yield; $[\alpha]_D^{20}$: +19.15 (*c* = 0.10 in MeOH); 78% ee, determined by chiral HPLC (Chiralpak AD), *n*-hexane/*i*-PrOH/CH_3COOH = 980/19/1, 1.0 mL/ min, λ = 254 nm; *t* (minor) = 7.822 min, *t* (major) = 11.579 min; ^1H NMR (400 MHz, Methanol-d_4): δ =7.71 – 7.65 (m, 2H), 7.64 (s, 1H), 7.13 (s, 1H), 7.12 – 7.07 (m, 2H), 7.04 (dd, *J* = 8.0, 2.1 Hz, 1H), 6.26 (s, 1H), 6.03 (s, 1H), 5.82 (s, 1H); ^{13}C NMR (100 MHz, Methanol-d_4): δ = 167.4, 160.1, 158.7, 158.3, 140.5, 131.9, 127.1, 127.01, 126.98, 126.94, 125.6, 125.2, 124.9, 124.52, 124.46, 122.9, 118.9, 118.3, 112.6, 79.4; ESI HR-MS: *m/z* = 364.0832, calcd. for $[C_{17}H_{12}BF_3O_5\text{-}H]^-$: 363.0657, found: 363.0648.

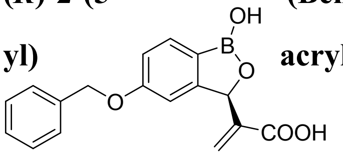

(*R*)-2-(5-(Benzyloxy)-1-hydroxy-1,3-dihydrobenzo[*c*][1,2]oxaborol-3-yl) acrylic acid (4p): semi-solid, 81% yield; $[\alpha]_D^{20}$: +50.65 (*c* = 0.45 in MeOH); 87% ee, determined by chiral HPLC (Chiralpak AD), *n*-hexane/*i*-PrOH/CH_3COOH = 980/19/1, 1.0 mL/ min, λ = 254 nm; *t* (minor) = 11.906 min, *t* (major) = 17.899 min; ^1H NMR (400 MHz, Methanol-d_4): δ =7.55 (d, *J* = 8.2 Hz, 1H), 7.41 (d, *J* = 7.5 Hz, 2H), 7.35 (t, *J* = 7.4 Hz, 2H), 7.29 (t, *J* = 7.2 Hz, 1H), 7.02 (d, *J* = 2.2 Hz, 1H), 6.97 (dd, *J* = 8.2, 2.2 Hz, 1H), 6.24 (s, 1H), 5.99 (s, 1H), 5.74 (s, 1H), 5.07 (s, 2H); ^{13}C NMR (100 MHz, Methanol-d_4): δ =167.7, 161.6, 158.0, 141.0, 136.9, 131.2, 128.1, 127.6, 127.3, 124.3, 115.2, 107.5, 79.2, 69.6; ESI HR-MS: *m/z* = 310.1120, calcd. for $[C_{17}H_{15}BO_5\text{-}H]^-$: 309.0940, found: 309.0947.

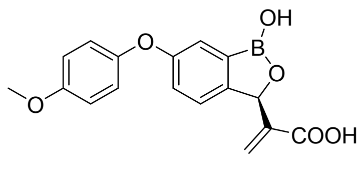

(*R*)-2-(1-Hydroxy-6-(4-methoxyphenoxy)-1,3-dihydrobenzo[c][1,2]oxaborol-3-yl) acrylic acid (4q): white solid, 72% yield; mp = 97.2-101.1 °C. $[\alpha]_D^{20}$: +47.89 (*c* = 0.25 in MeOH); 88% ee (93% ee after recrystallization from EA), determined by chiral HPLC (Chiralpak AD), *n*-hexane/ *i*-PrOH/ CH_3COOH =

980/19/1, 1.0 mL/ min, λ = 254 nm; *t* (minor) = 16.031 min, *t* (major) = 18.446 min; ^{1}H NMR (400 MHz, Methanol-d_4): δ =7.34 (d, *J* = 8.4 Hz, 1H), 7.09 (d, *J* = 2.4 Hz, 1H), 7.04 – 6.99 (m, 1H), 6.94 – 6.88 (m, 4H), 6.26 (s, 1H), 6.02 (s, 1H), 5.79 (s, 1H), 3.79 (s, 3H); ^{13}C NMR (100 MHz, Methanol-d_4): δ = 167.4, 158.4, 156.1, 150.2, 150.0, 140.9, 124.2, 123.0, 120.7, 120.2, 117.5, 114.6, 79.4, 54.6, 23.8; ESI HR-MS: *m/z* = 326.1110, calcd. for $[C_{17}H_{15}BO_6\text{-}H]^-$: 325.0889, found: 325.0896. (*S*)-**4q**: white solid, 79% yield; $[\alpha]_D^{20}$: -28.80 (*c* = 0.20 in MeOH); 51% ee (90% ee after recrystallization from EA) determined by chiral HPLC (Chiralpak AD), *n*-hexane/*i*-PrOH/CH_3COOH = 980/19/1, 1.0 mL/ min, λ = 254 nm; *t* (minor) = 15.670 min, *t* (major) = 13.418 min.

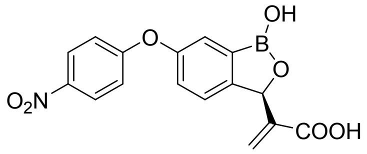

(*R*)-2-(1-Hydroxy-6-(4-nitrophenoxy)-1,3-dihydrobenzo[*c*][1,2]oxaborol-3-yl) acrylic acid (4r): semi-solid, 72% yield; $[\alpha]_D^{20}$: +31.29 (*c* = 0.12 in MeOH); 86% ee, determined by chiral HPLC (Chiralpak AD), *n*-hexane/ *i*-PrOH/ CH_3COOH = 980/19/1, 1.0 mL/ min, λ = 254 nm; *t* (minor) = 7.595 min, *t* (major) = 9.326 min; ^{1}H NMR (400 MHz, Methanol-d_4): δ =8.29 – 8.11 (m, 2H), 7.50 (d, *J* = 8.3 Hz, 1H), 7.34 (d, *J* = 2.4 Hz, 1H), 7.21 (dd, *J* = 8.3, 2.4 Hz, 1H), 7.12 – 6.96 (m, 2H), 6.30 (s, 1H), 6.08 (s, 1H), 5.86 (s, 1H); ^{13}C NMR (100 MHz, Methanol-d_4): δ = 167.3, 163.5, 154.6, 152.4, 142.7, 140.5, 125.6, 124.8, 123.8, 123.4, 121.1, 116.9, 79.7, 53.4; ESI HR-MS: *m/z* = 341.0820, calcd. for $[C_{16}H_{12}BO_6\text{-}H]^-$: 340.0634, found: 340.0634.

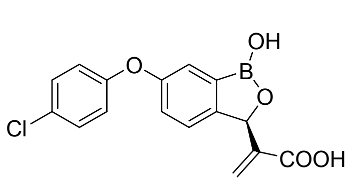

(*R*)-2-(6-(4-Chlorophenoxy)-1-hydroxy-1,3-dihydrobenzo[*c*][1,2]oxaborol-3-yl)acrylic acid (4s): semi-solid, 71% yield; $[\alpha]_D^{20}$: +12.53 (*c* = 0.12 in MeOH); 84% ee, determined by chiral HPLC (Chiralpak AD), *n*-hexane/*i*-PrOH/CH_3COOH = 980/19/1, 1.0 mL/ min, λ = 254 nm; *t* (minor) = 11.134 min, *t* (major) = 12.089 min; ^{1}H NMR (400 MHz, Methanol-d_4): δ = 7.40 (d, *J* = 8.4 Hz, 1H), 7.36 – 7.28 (m, 2H), 7.19 (d, *J* = 2.4 Hz, 1H), 7.10 (dd, *J* =

8.4, 2.4 Hz, 1H), 6.98 – 6.86 (m, 2H), 6.27 (s, 1H), 6.05 (s, 1H), 5.82 (s, 1H); ^{13}C NMR (100 MHz, Methanol-d_4): δ = 167.3, 156.5, 156.3, 150.8, 140.7, 129.4, 127.8, 124.5, 123.3, 122.00, 121.95, 119.5, 119.3, 79.6. ESI HR-MS: *m/z* = 330.5270, calcd. for $[C_{16}H_{12}BClO_5\text{-}H]^-$: 329.0394, found: 329.0392.

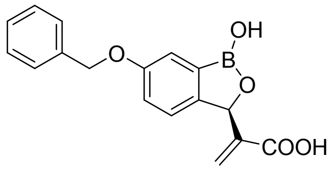

(*R*)-2-(6-(Benzyloxy)-1-hydroxy-1,3-dihydrobenzo[*c*][1,2]oxaborol-3-yl) acrylic acid (4t): semi-solid, 83% yield; $[\alpha]_D^{20}$: +29.05 (*c* = 0.20 in MeOH); 80% ee, determined by chiral HPLC (Chiralpak AD), *n*-hexane/*i*-PrOH/CH_3COOH = 980/19/1, 1.0 mL/ min, λ = 254 nm; *t* (minor) = 12.694 min, *t* (major) = 16.429 min; ^1H NMR (400 MHz, Methanol-d_4) δ =7.43 (d, *J* = 7.1 Hz, 2H), 7.39 – 7.33 (m, 2H), 7.32 – 7.26 (m, 2H), 7.20 (d, *J* = 2.5 Hz, 1H), 7.07 (dd, *J* = 8.4, 2.5 Hz, 1H), 6.18 (s, 1H), 6.00 (s, 1H), 5.75 (s, 1H), 5.08 (s, 2H); ^{13}C NMR (100 MHz, Methanol-d_4): δ = 167.6, 158.6, 148.1, 141.1, 137.3, 128.1, 127.9, 127.5, 127.2, 124.1, 122.8, 118.9, 114.2, 79.4, 69.8; ESI HR-MS: *m/z* = 310.1120, calcd. for $[C_{17}H_{15}BO_5\text{-}H]^-$: 309.0940, found: 309.0934.

(6) The proposed mechanism for the enantioselective synthesis of (*R*)-enantiomers in the presence of catalyst C6

Based on the absolute stereochemistry of the products and a literature report,[5] a mechanism for the reaction can be proposed as exemplified for catalyst **C6**. Following Michael addition of **C6** to acrylate **2** to forms enolate intermediate **I**, aldol reaction with the boronic acid activated aldehyde **1a** gives a mixture of two diastereomers **IIA** and **IIB**, stabilized by intramolecular hydrogen bonds between the oxy anion/boronic acid and N-H of **C6**. By contrast, the other two possible diastereomers **IIC** and **IID** cannot form such hydrogen bonding interactions. **IIB** may be less stable than **IIA** due to steric interactions between phenylboronic acid, the ester and quinuclidine moieties in the following E2 or E1cb elimination reactions, as apparent in Newman projection

of **IIB**. Density functional theory (DFT) calculations revealed that transformation of **IIA** into cyclic boronate intermediate **IIIA** (predicted to be more stable than **IIA**) may be aided by hydrogen-bonding interactions between the boronate hydroxyl and N-H of **C6** (see video in additional material); **IIIA** is transformed into (*R*)-**4a** by E2 or E1cb elimination then hydrolysis. In addition, (*R*)-**3a'**, which is generated after elimination of catalyst **C6**, can undergo intramolecular cyclization and hydrolysis to form (*R*)-**4a**. Note there was no evidence that (*R*)-**3a'** reacts with **1a** to form the potential dioxanone byproduct. The proposed mechanism is consistent both with the stereoselectivity and reaction substrate scope, in the lalter case with respect to C-5 or C-6 substituents.

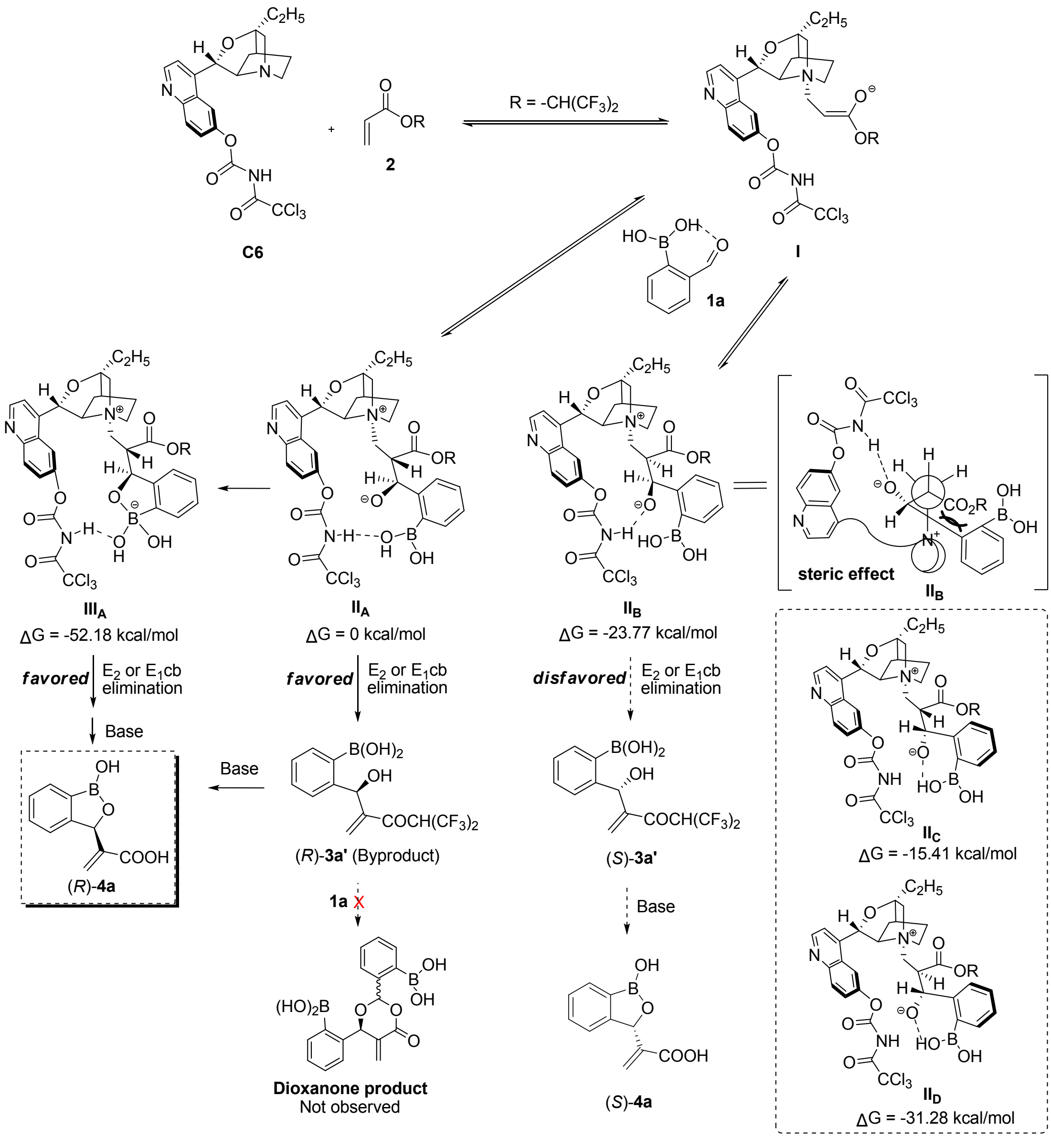

(5) Density Functional Theory (DFT) Calculations for Proposed Intermediates

To assist in the evaluation of the proposed mechanism (Scheme 2), we conducted DFT computations using the QChem program (v5.3). Using the 3-21G basis set and HF method, the four proposed key intermediates were optimized to lowest energy, and their single point energies calculated. Through the whole calculation process, implicit solvent was taken into consideration, which was implemented by the PCM solvent method with the solvent DIELECTRIC constant set as 2.2 (*i.e.*, the dielectric constant of reaction solvent dioxane at 25℃). We selected the energy of the 13rd optimization step of II_A as a reference to compare the energies of final optimized intermediate structures as shown in following.

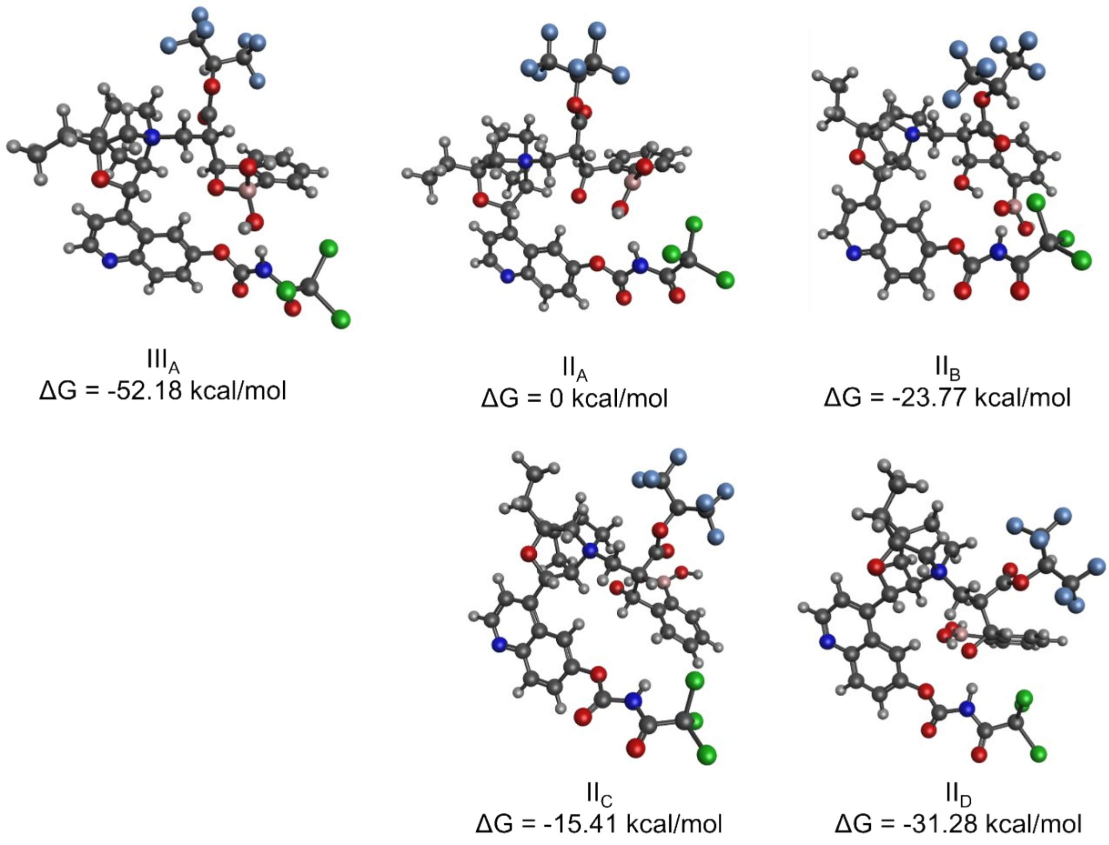

Optimized structures of the proposed intermediates in the asymmetric MBH reaction (see Scheme 2 main text).

SE. 2 Biology

(1) Recombinant Protein Production and Purification

Recombinant KPC-2, AmpC, OXA-48, NDM-1, VIM-2, and Sfh-I proteins were produced in *Escherichia coli* and purified (to >90% by SDS-PAGE) using reported procedures.[6]

(2) Carbapenemase Inhibition Assays

All assays were performed as reported.[6] The concentrations of enzymes used were: KPC-2, 2.5 nM; AmpC, 0.8 nM; OXA-48, 20 nM; NDM-1, 0.2 nM; VIM-1, 0.2 nM; IMP-1, 0.8 nM; and VIM-2, 0.2 nM; and 10 μM or 5 μM FC-5[7] were employed for Sfh-I and other tested enzymes, respectively. The assay buffer supplemented with 0.01% (v/v) Triton X-100 was used for all the tested enzymes. In general, the enzyme was preincubated with compounds for 10 min at room temperature; reactions were initiated by adding FC-5 substrate; the fluorescence intensity at λ_{ex} of 380 nm and λ_{em} of 460 nm wsa measured. All IC_{50} values were fitted by using GraphPad Prism.

(3) Minimal Inhibitory Concentrations (MICs)

The clinically isolated strains were used for the antimicrobial susceptibility test, and the ATCC25922 strain was used for quality control. The standard broth micro-dilution method was used to determine MIC values according to the Clinical and Laboratory Standards Institute (CLSI) guideline (please find the details in our previous work[6a]). Meropenem was tested alone (from 128 μg/ml to 0.25 μg/ml in 2-fold dilution) and in combination with compounds (100 μM and 10 μM). The 96-well plates were incubated for 16-20 h at 37 °C and MIC values were mainly determined by visual reading.

(4) Crystallization and Data Collection

X-ray crystal structures of KPC-2:(*S*)-**4a** and OXA-48:(*R*)-**4a** complexes were obtained by co-crystallisation procedures. In brief, the mixtures of 10 mg/mL KPC-2 and 3.5 mM (*S*)-**4a** or 12 mg/mL OXA-48 and 3.5 mM (*R*)-**4a** were incubated for 60

min on ice. The crystallization drops contained 1:1 ratios of KPC-2:(*S*)-**4a** or OXA-48:(*R*)-**4a** complex mixtures : reservoir solutions (Table S2). The crystals were cryoprotected using a solution of ~30% (v/v) glycerol, and cooled in liquid nitrogen. Data were collected and processed using HKL2000 at the Shanghai Synchrotron Radiation Facility. Molecular replacement and refinement were carried out using Phenix[8] and Wincoot[9].

Copies of ^{1}H and ^{13}C NMR Spectra

^{1}H NMR Spectra of (*R*)-**4a** (400 MHz, CD_3OD)

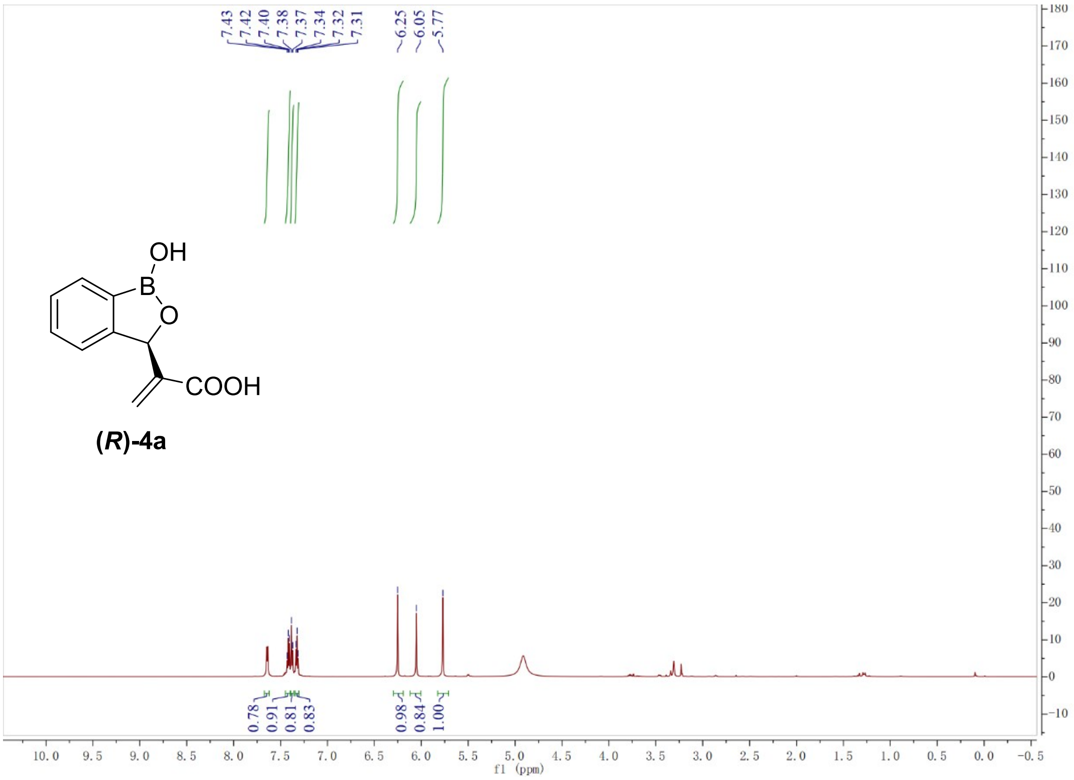

^{13}C NMR Spectra of (*R*)-**4a** (100 MHz, CD_3OD)

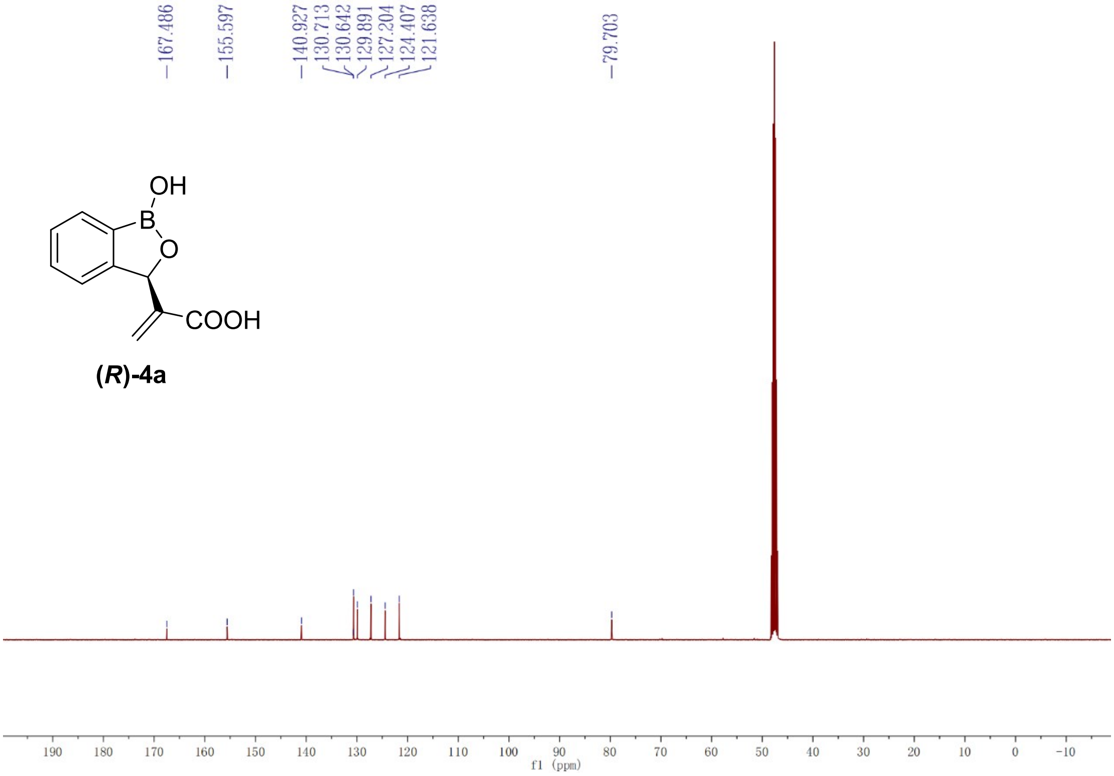

^{1}H NMR Spectra of (*R*)-**4b** (400 MHz, DMSO-d_6)

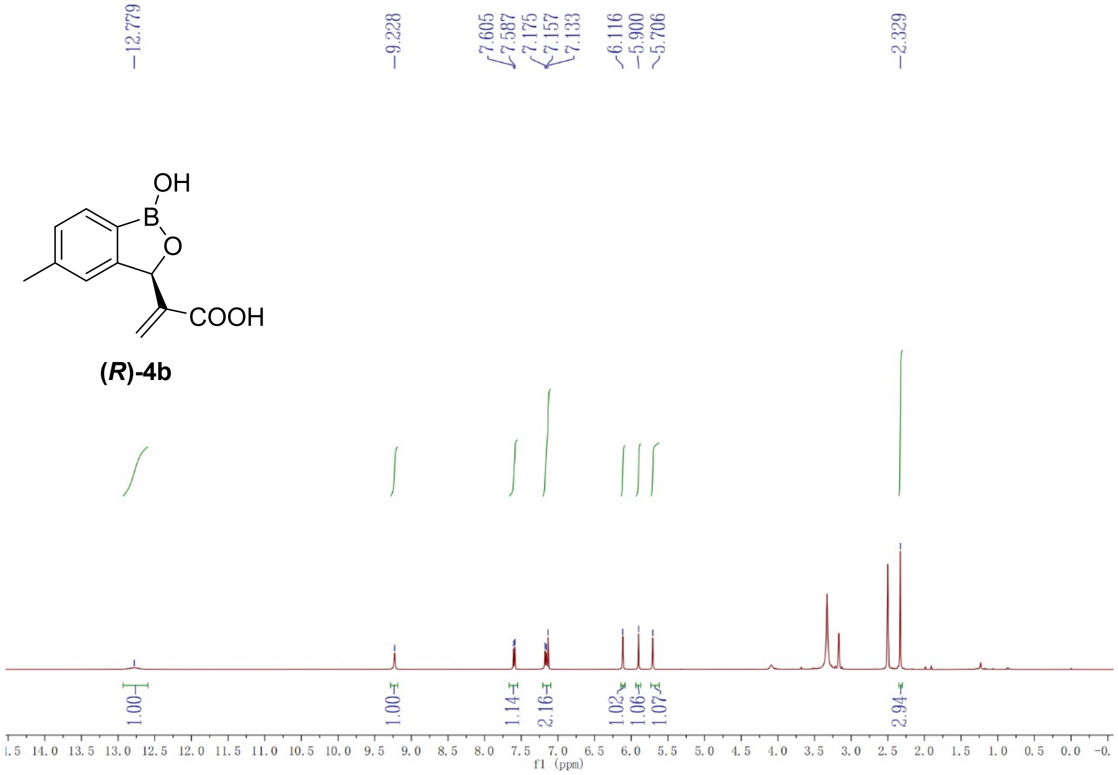

^{13}C NMR Spectra of (*R*)-**4b** (100 MHz, DMSO-d_6)

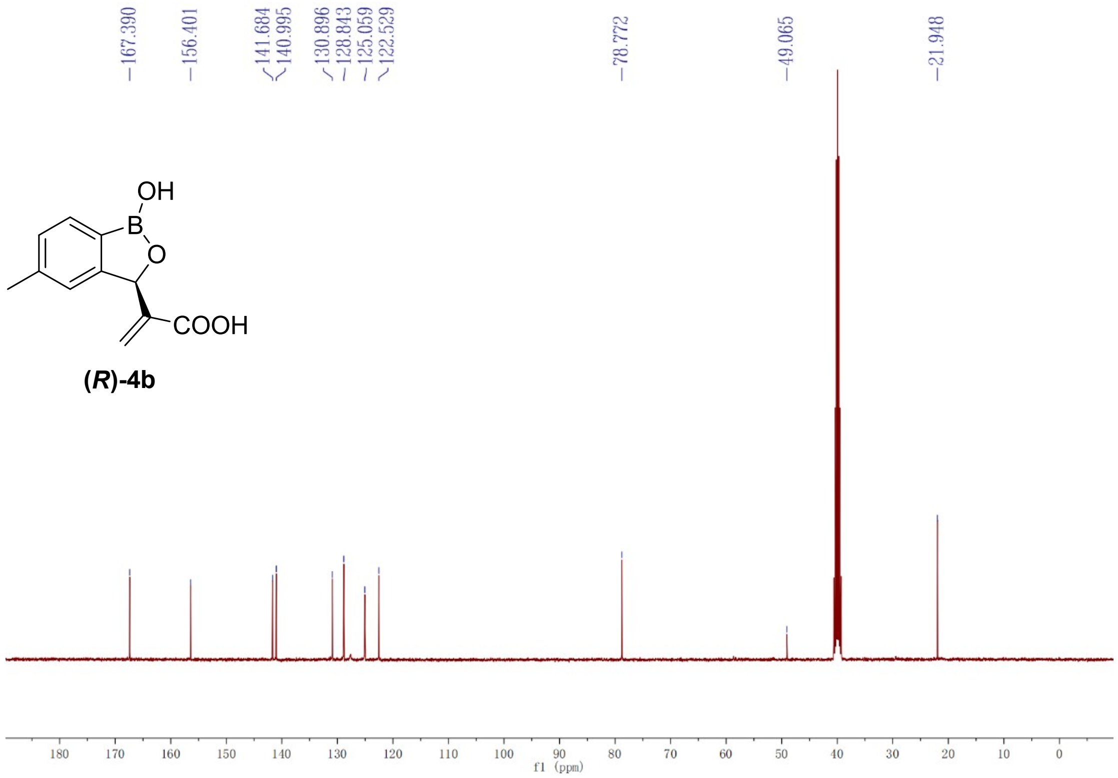

^{1}H NMR Spectra of (*R*)-**4c** (400 MHz, CD_3OD)

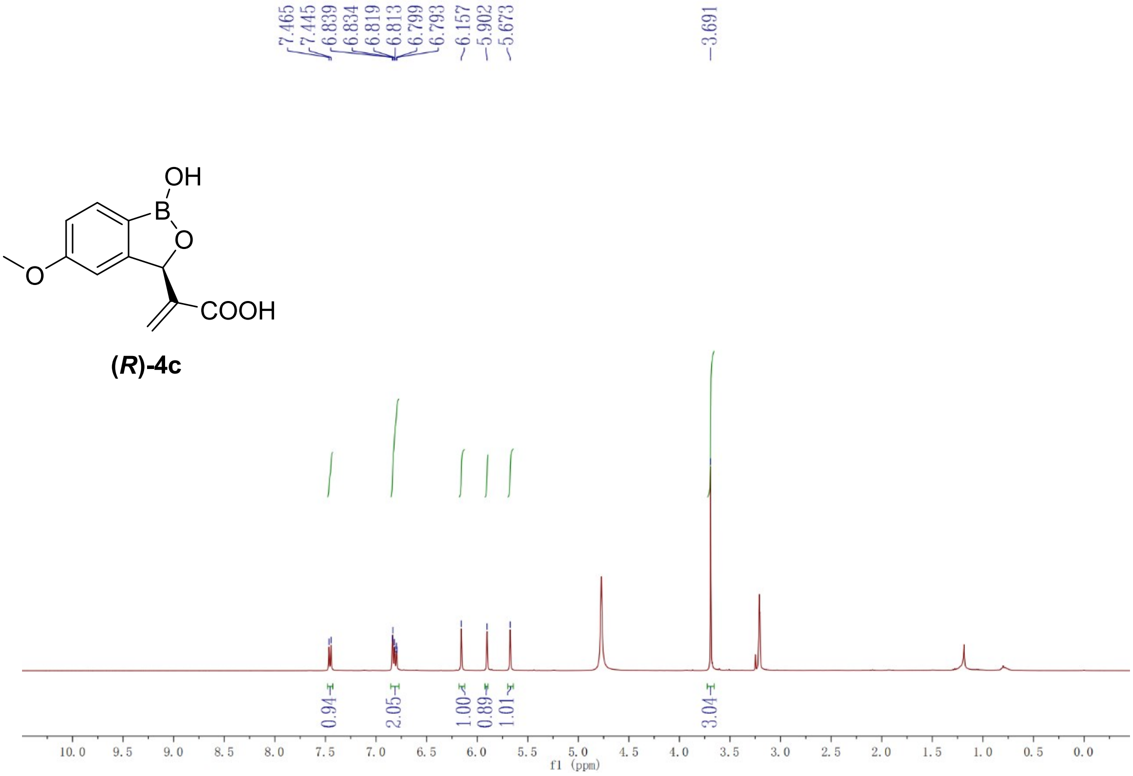

^{13}C NMR Spectra of (*R*)-**4c** (100 MHz, CD_3OD)

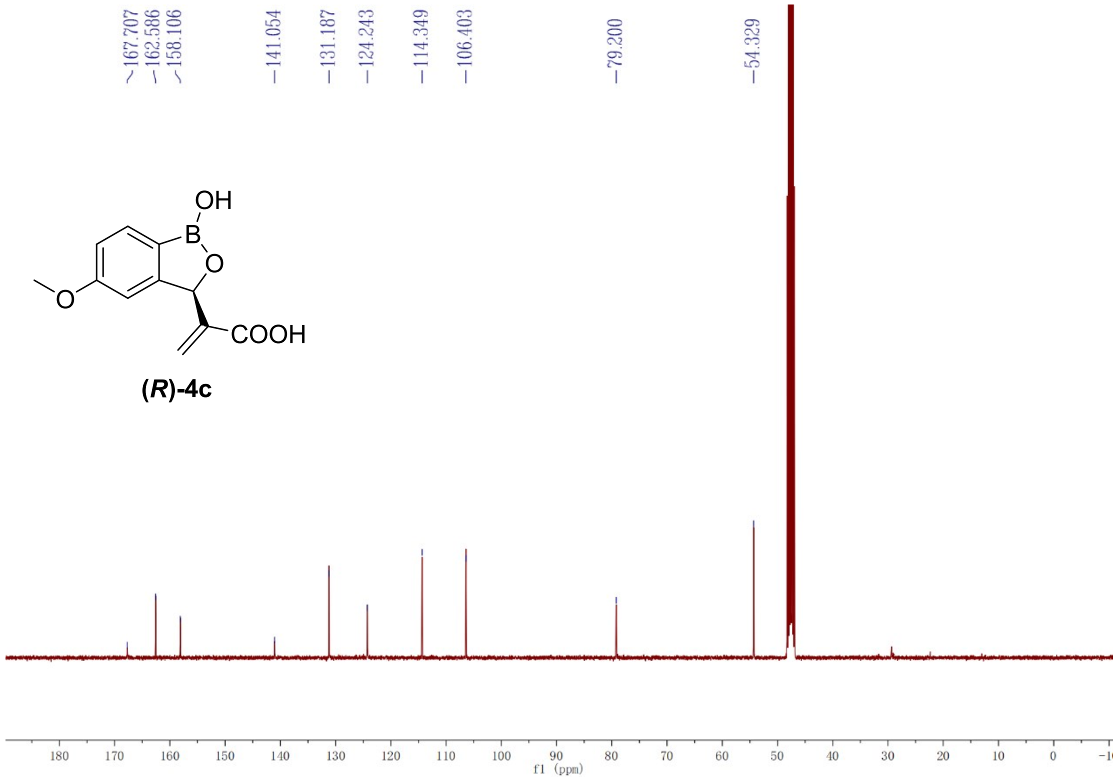

^{1}H NMR Spectra of (*R*)-**4d** (400 MHz, CD_3OD)

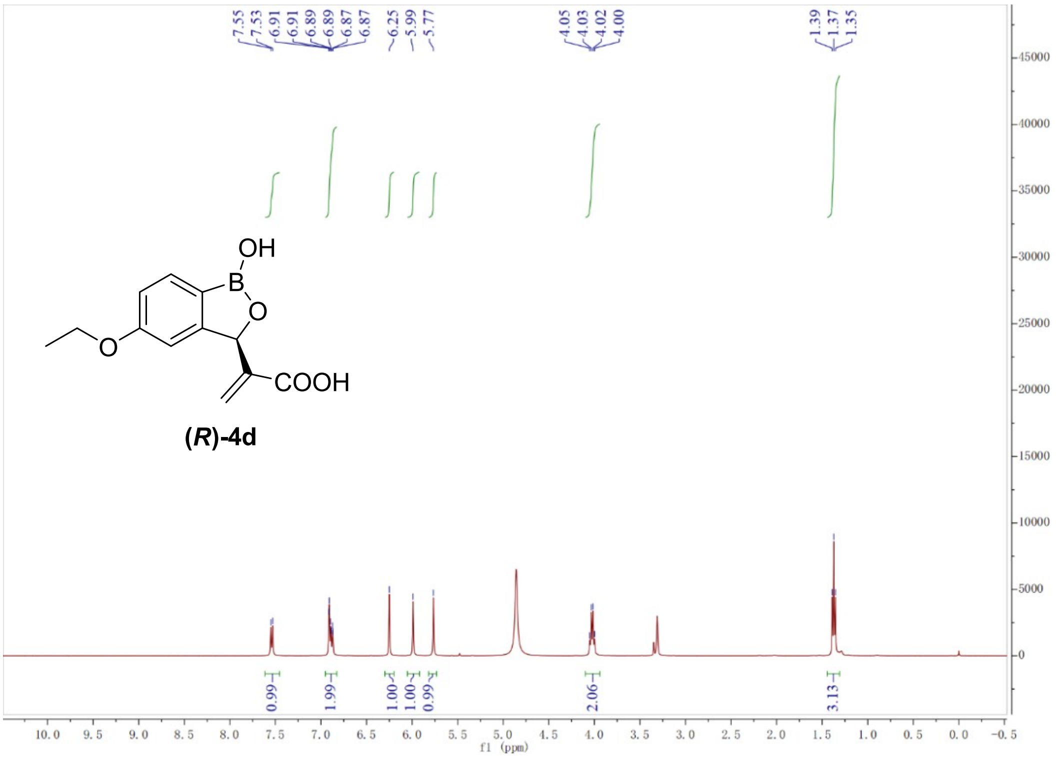

^{13}C NMR Spectra of (*R*)-**4d** (100 MHz, CD_3OD)

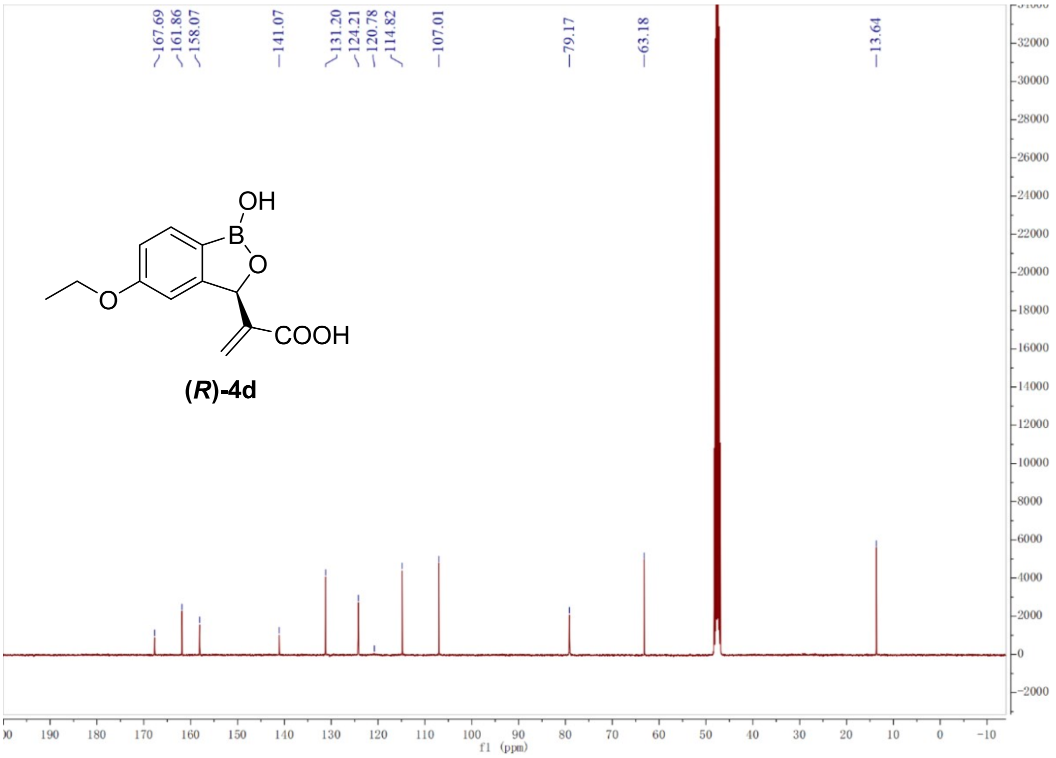

^{1}H NMR Spectra of (*R*)-**4e** (400 MHz, CD_3OD)

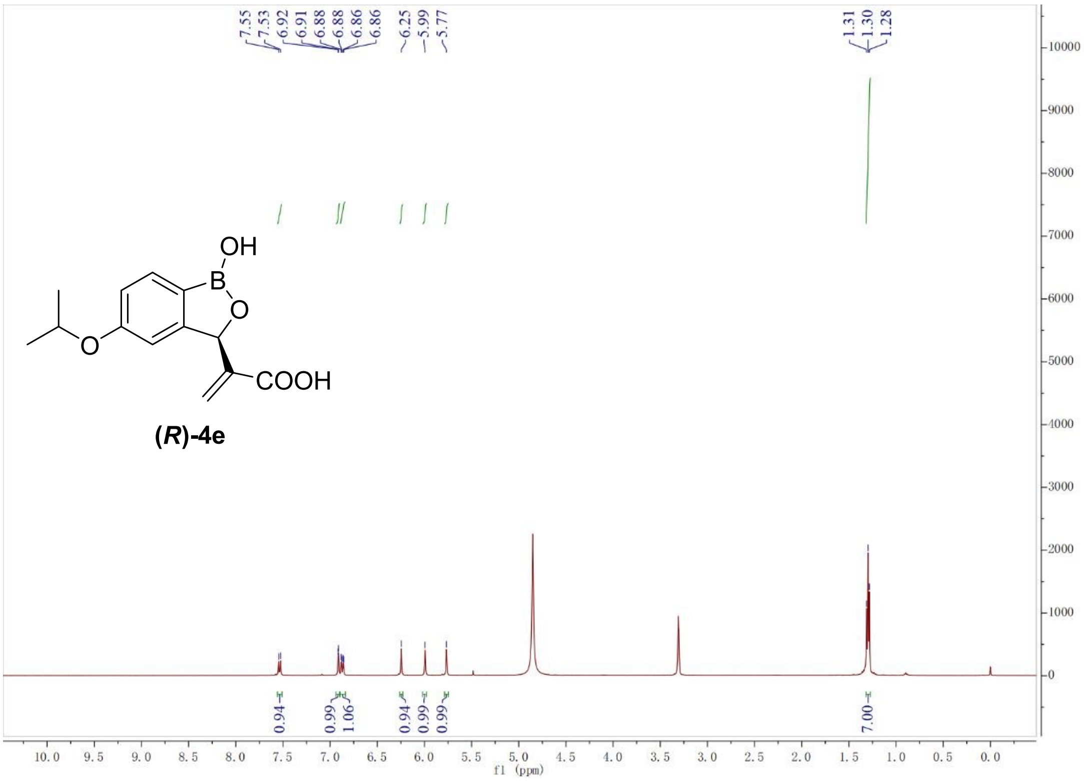

^{13}C NMR Spectra of (*R*)-**4e** (100 MHz, CD_3OD)

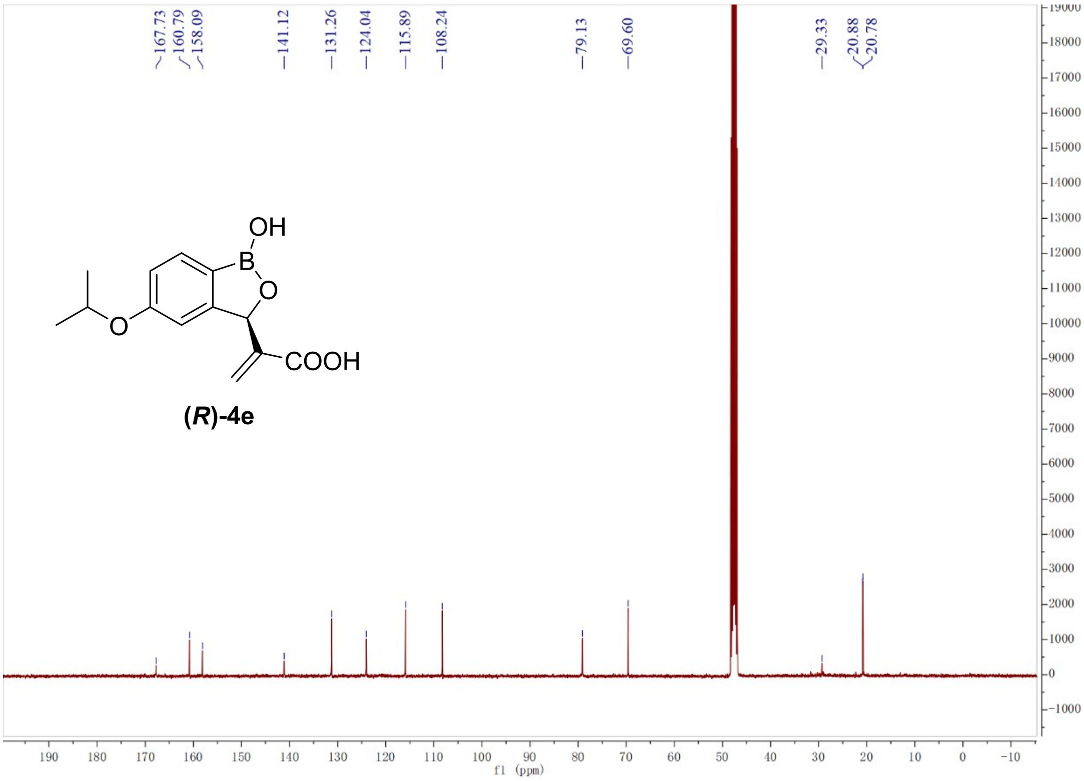

^{1}H NMR Spectra of (*R*)-**4f** (400 MHz, CD_3OD)

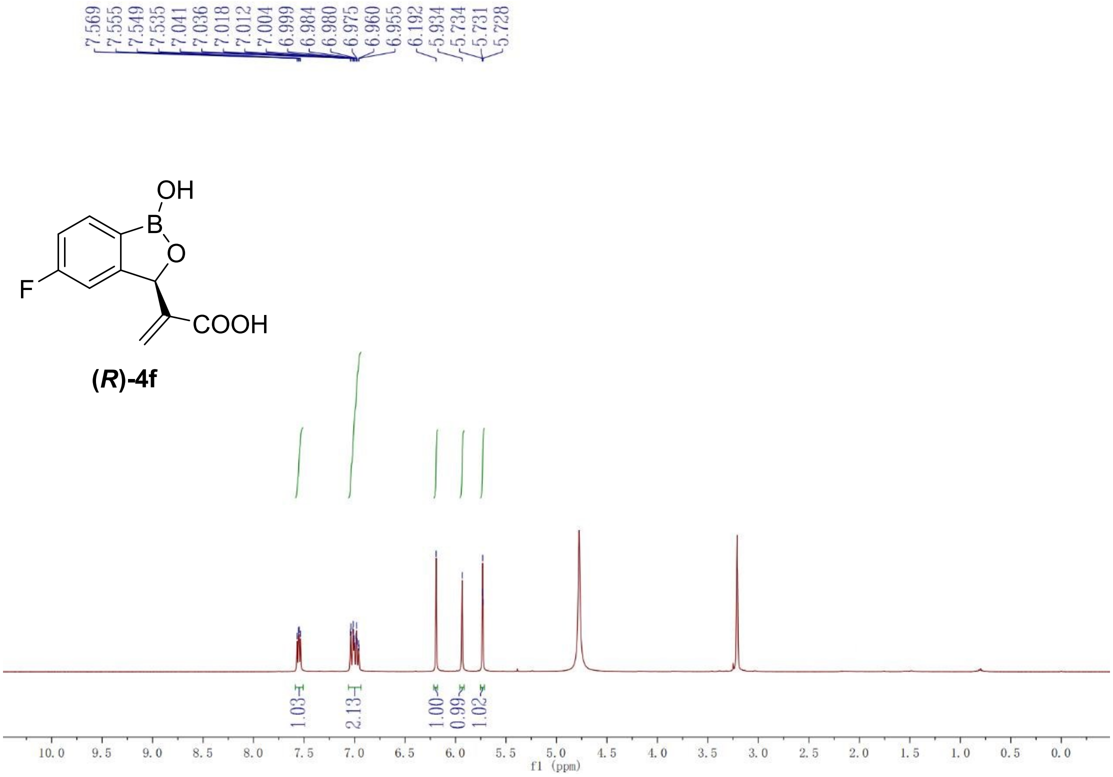

^{13}C NMR Spectra of (*R*)-**4f** (100 MHz, CD_3OD)

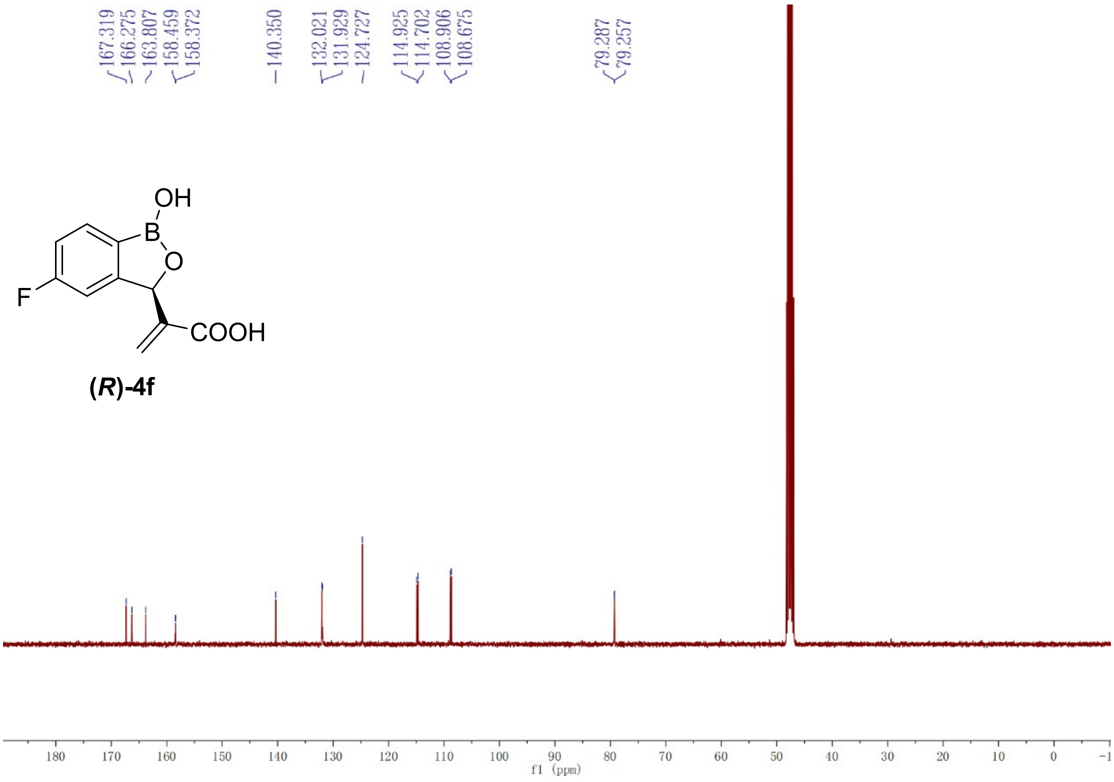

^{1}H NMR Spectra of (*R*)-**4g** (400 MHz, CD_3OD)

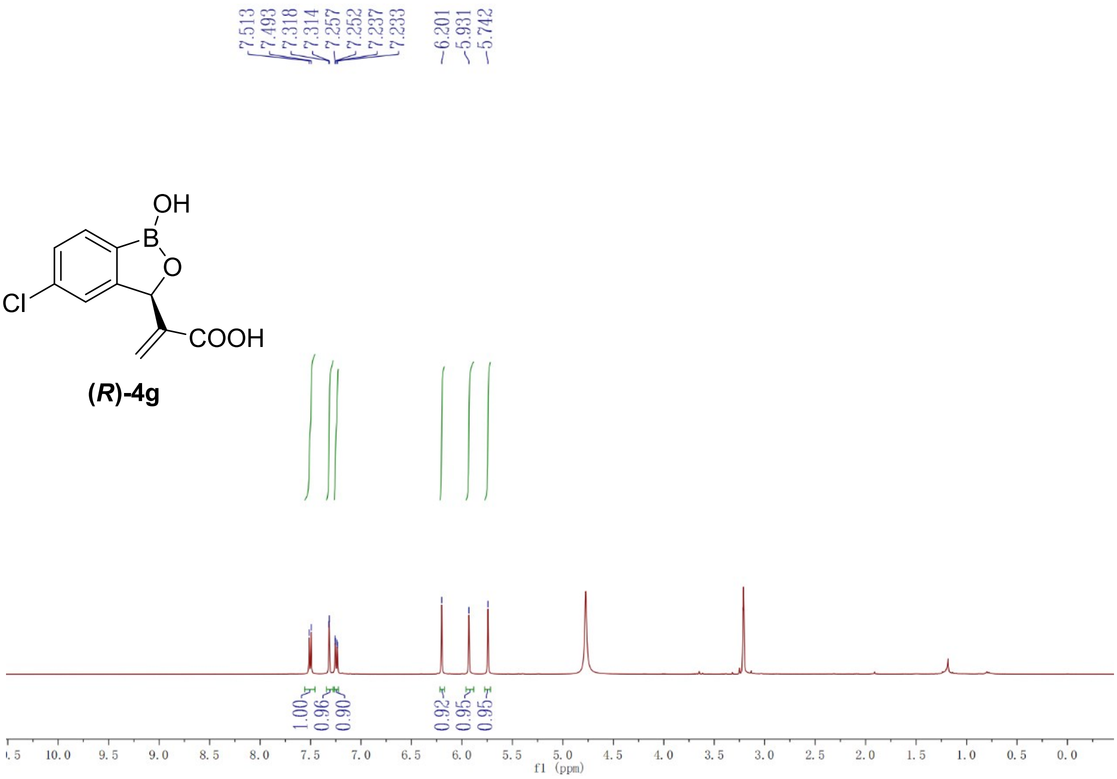

^{13}C NMR Spectra of (*R*)-**4g** (100 MHz, CD_3OD)

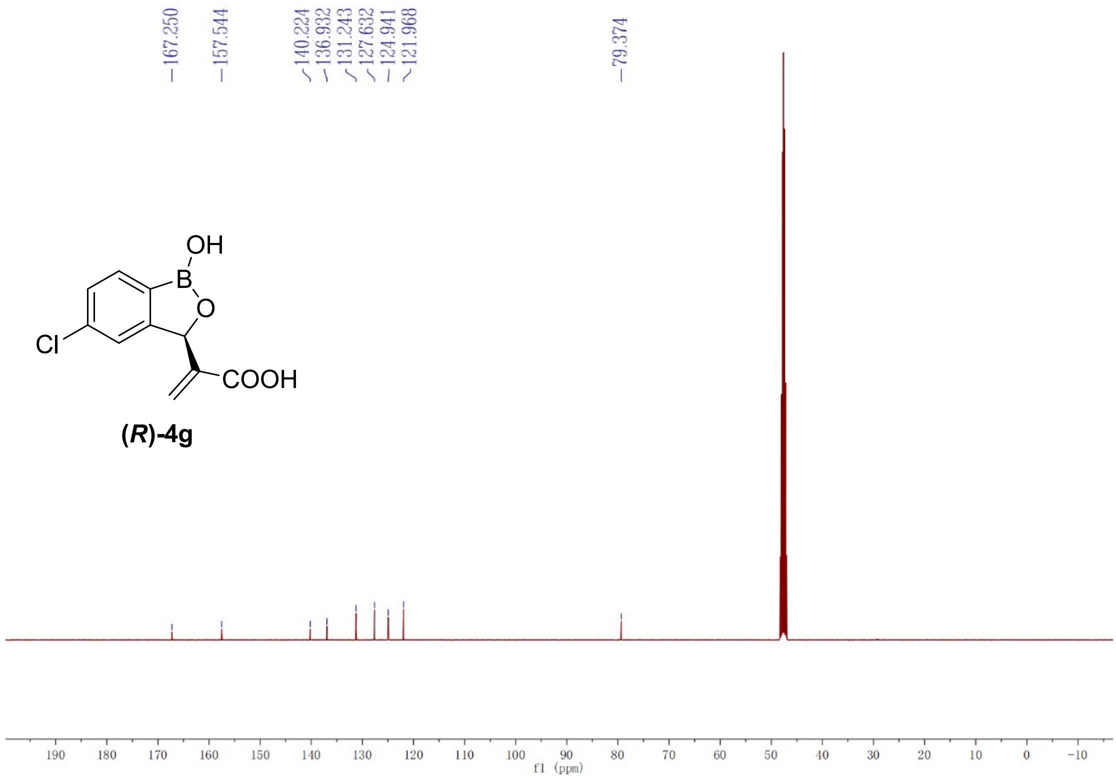

^{1}H NMR Spectra of (*R*)-**4h** (400 MHz, DMSO-d_6)

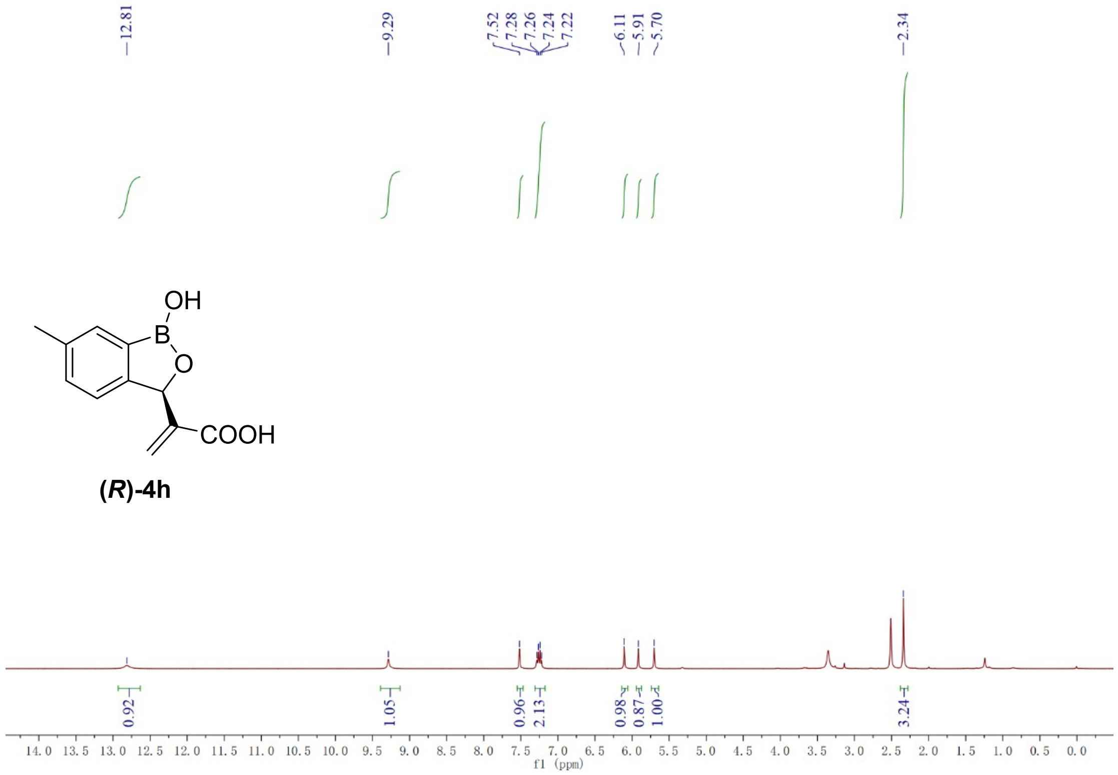

^{13}C NMR Spectra of (*R*)-**4h** (100 MHz, DMSO-d_6)

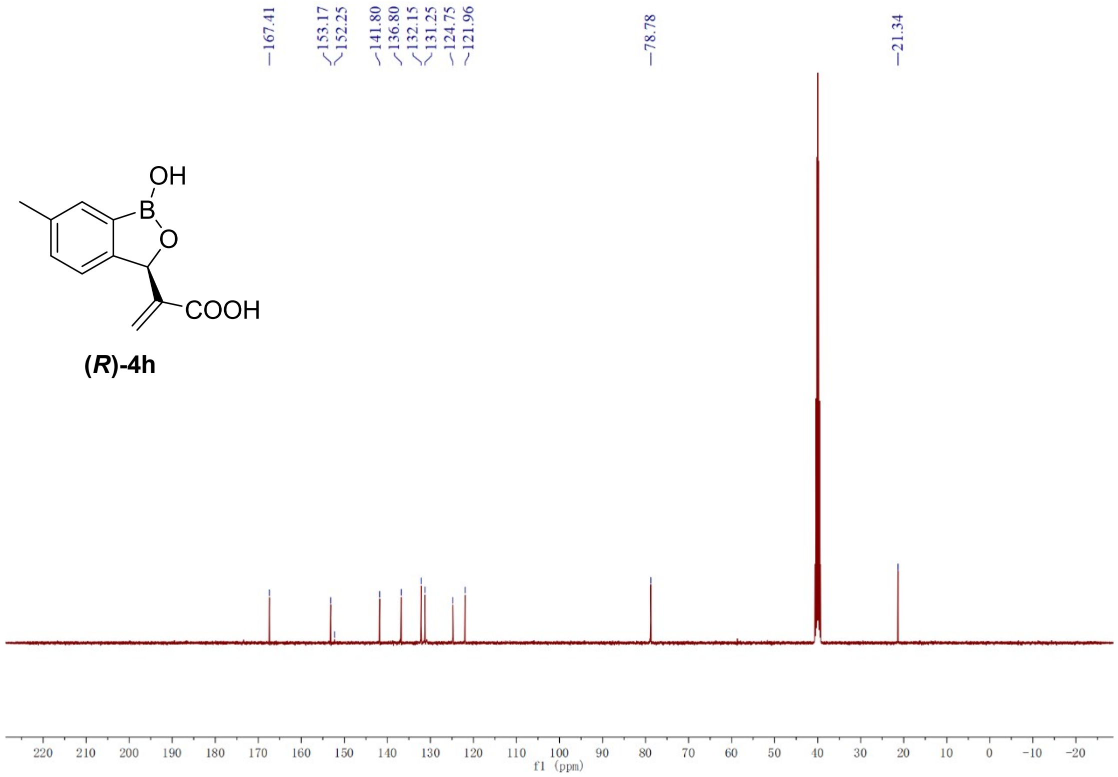

^{1}H NMR Spectra of (R)-**4i** (400 MHz, DMSO-d_6)

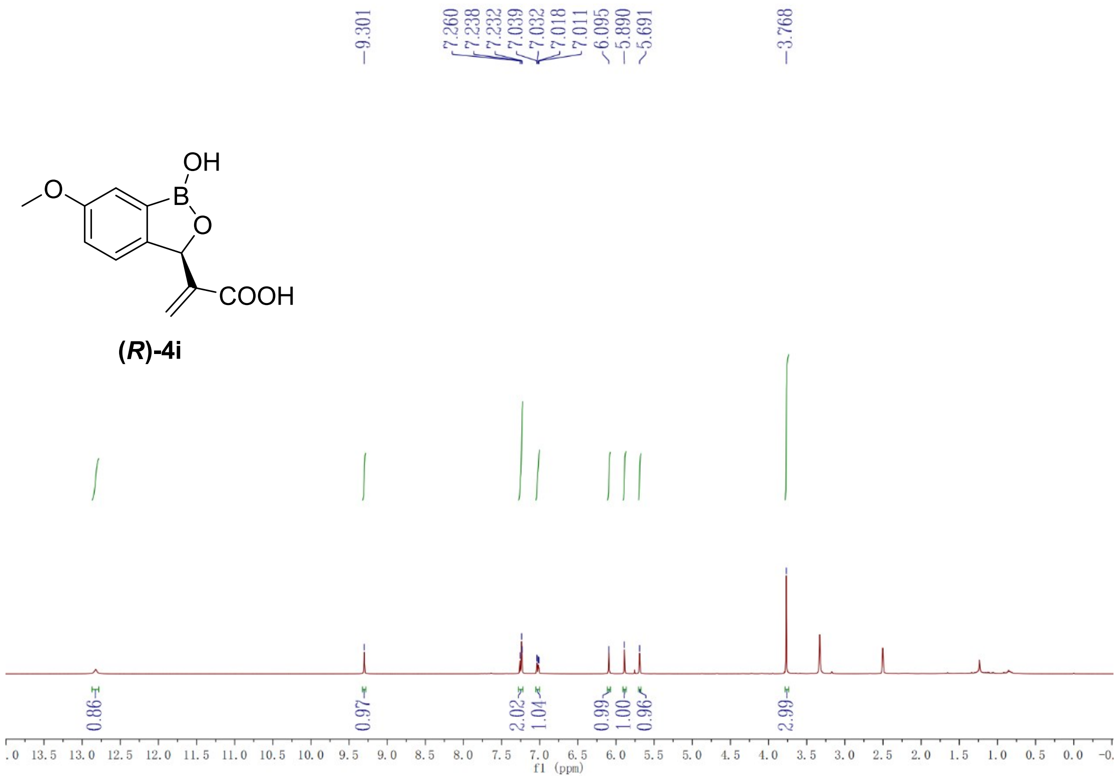

^{13}C NMR Spectra of (R)-**4i** (100 MHz, DMSO-d_6)

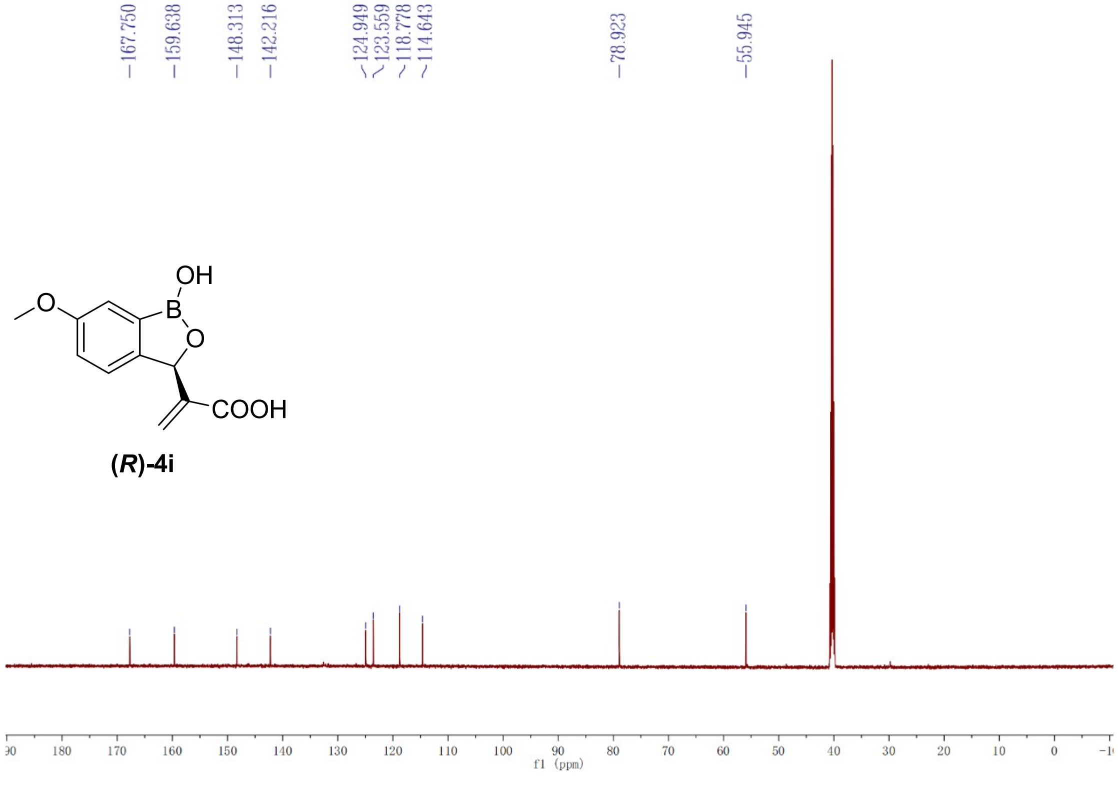

^{1}H NMR Spectra of (*R*)-**4j** (400 MHz, CD_3OD)

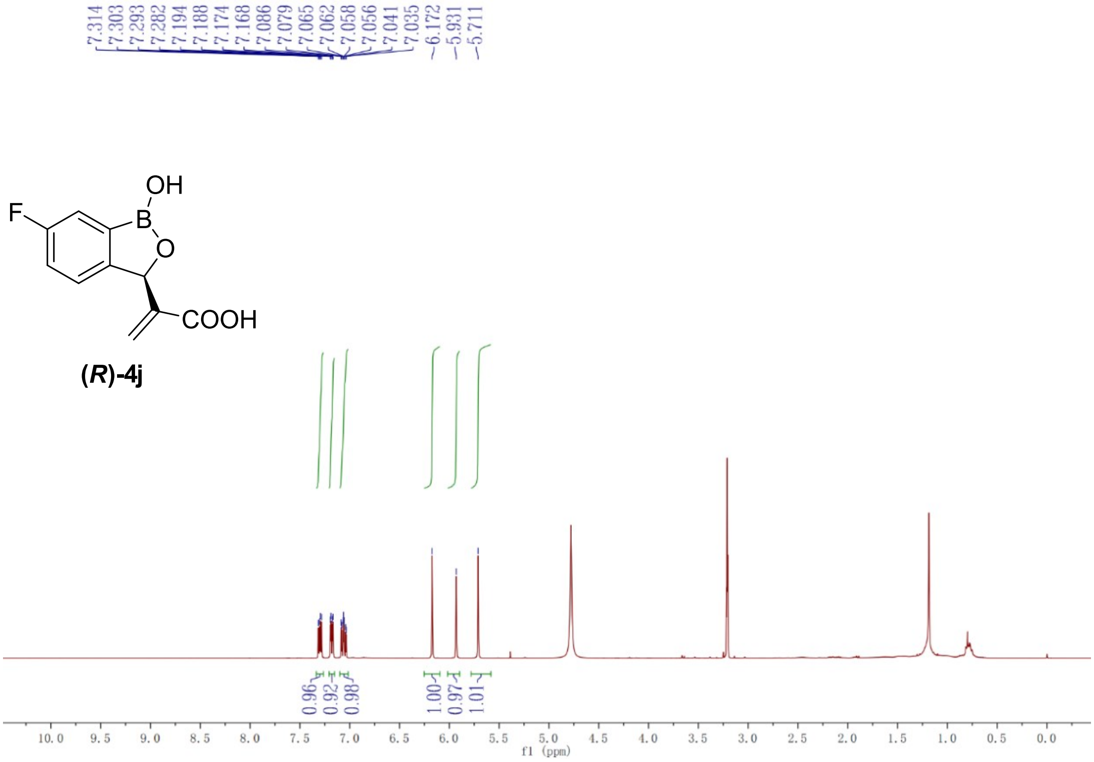

^{13}C NMR Spectra of (*R*)-**4j** (100 MHz, CD_3OD)

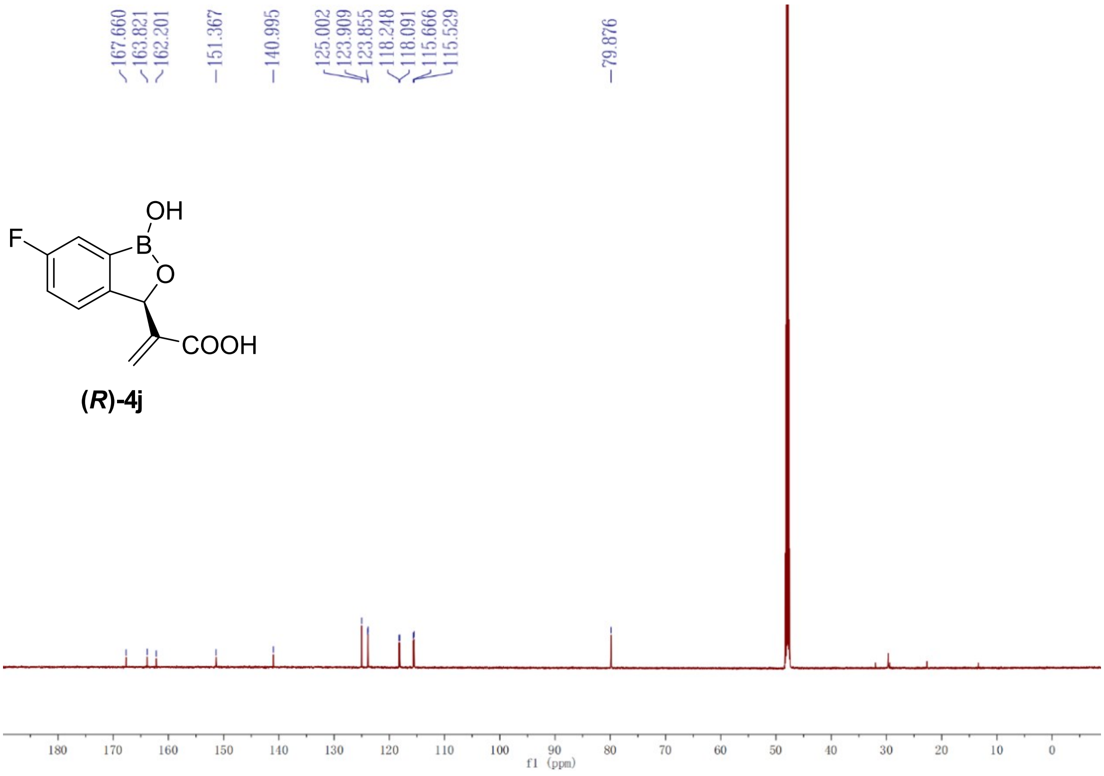

^{1}H NMR Spectra of (*R*)-**4k** (400 MHz, CD_3OD)

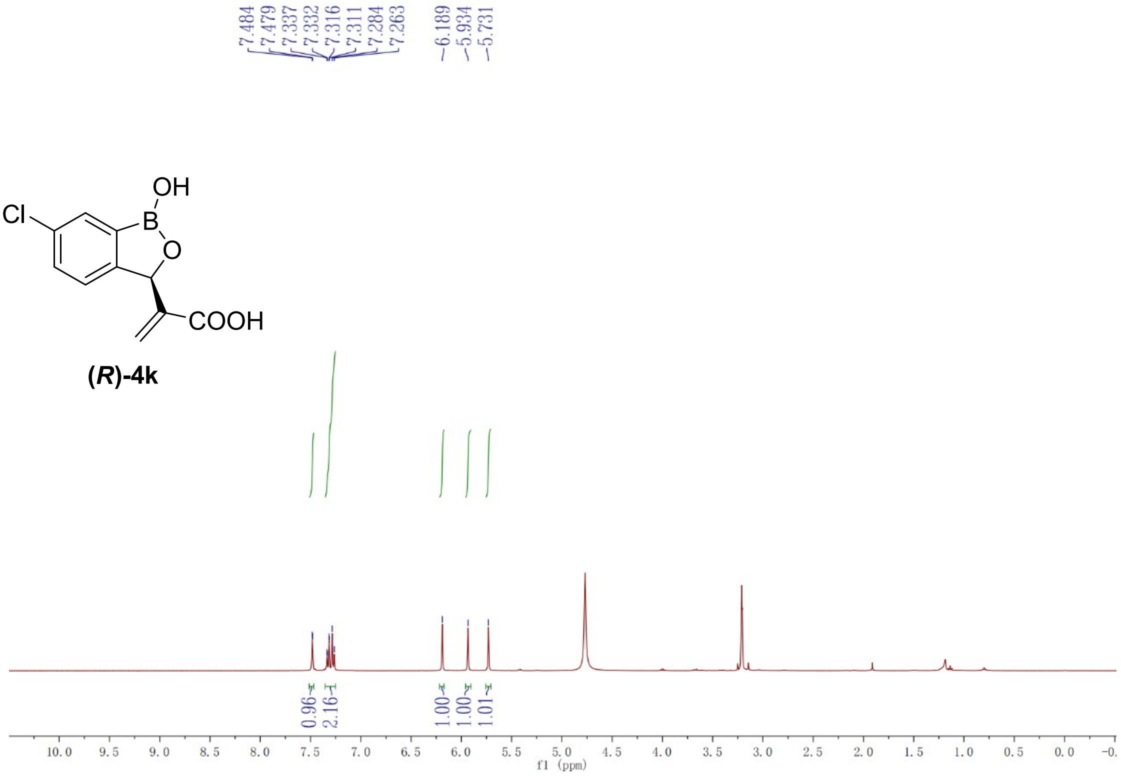

^{13}C NMR Spectra of (*R*)-**4k** (100 MHz, CD_3OD)

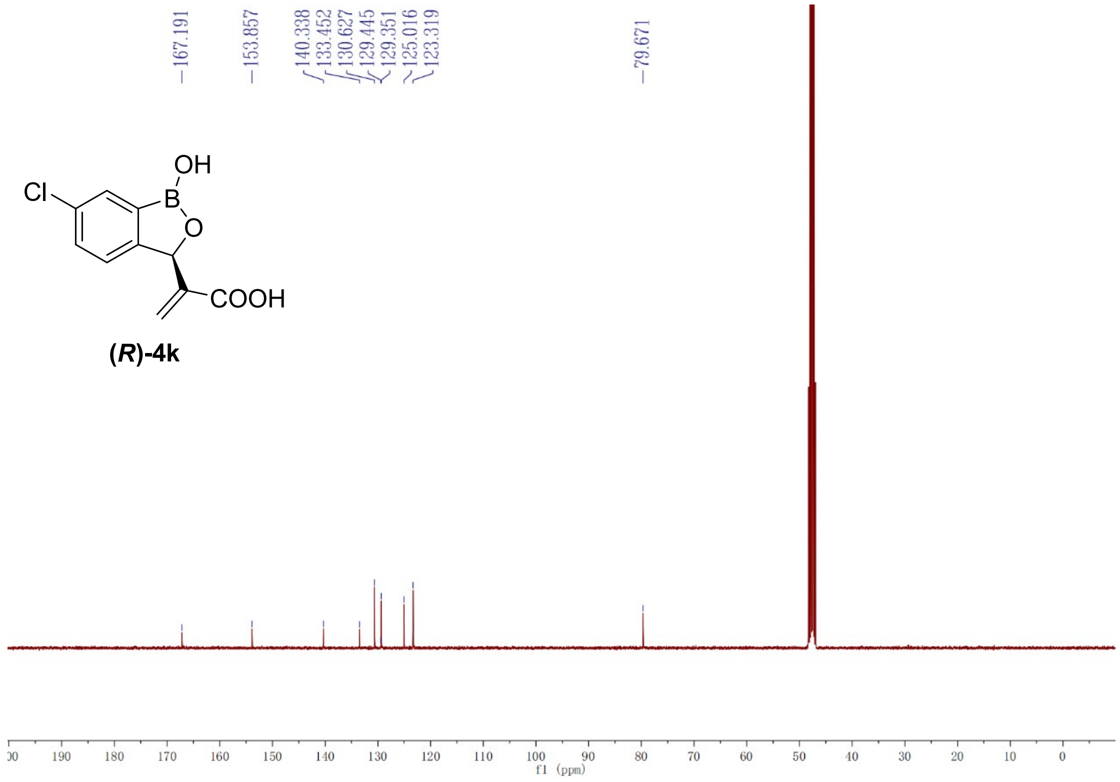

^{1}H NMR Spectra of (*R*)-**4l** (400 MHz, DMSO-d_6)

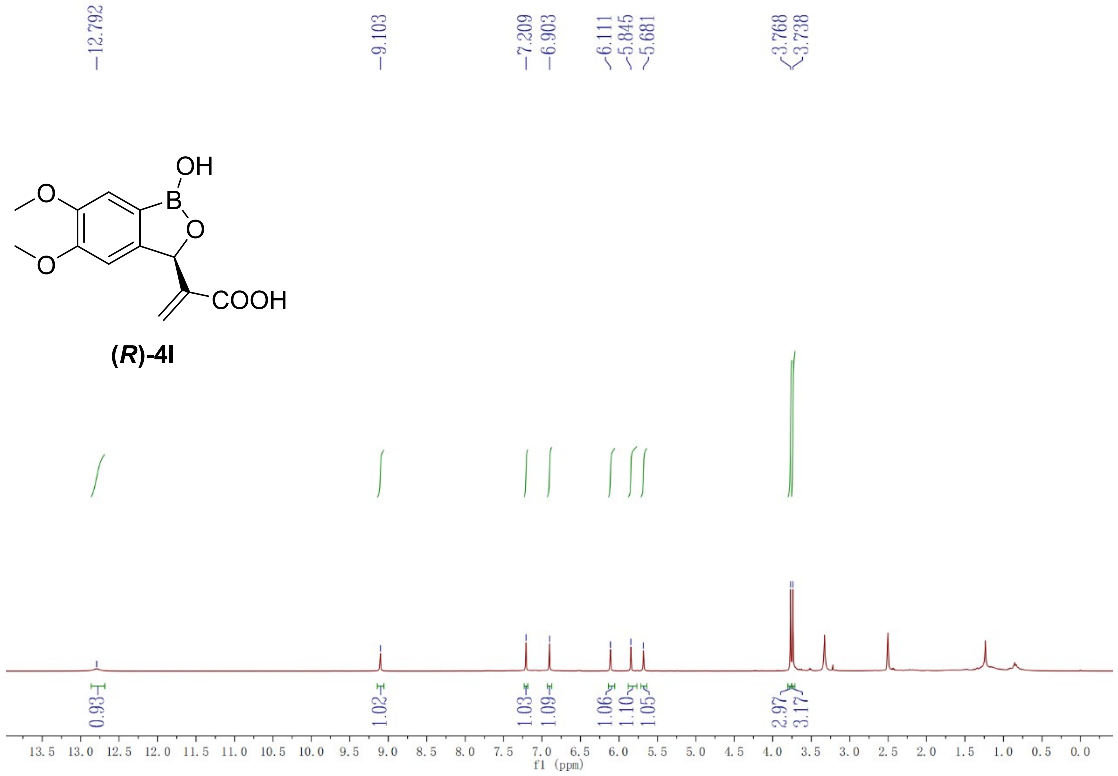

^{13}C NMR Spectra of (*R*)-**4l** (100 MHz, DMSO-d_6)

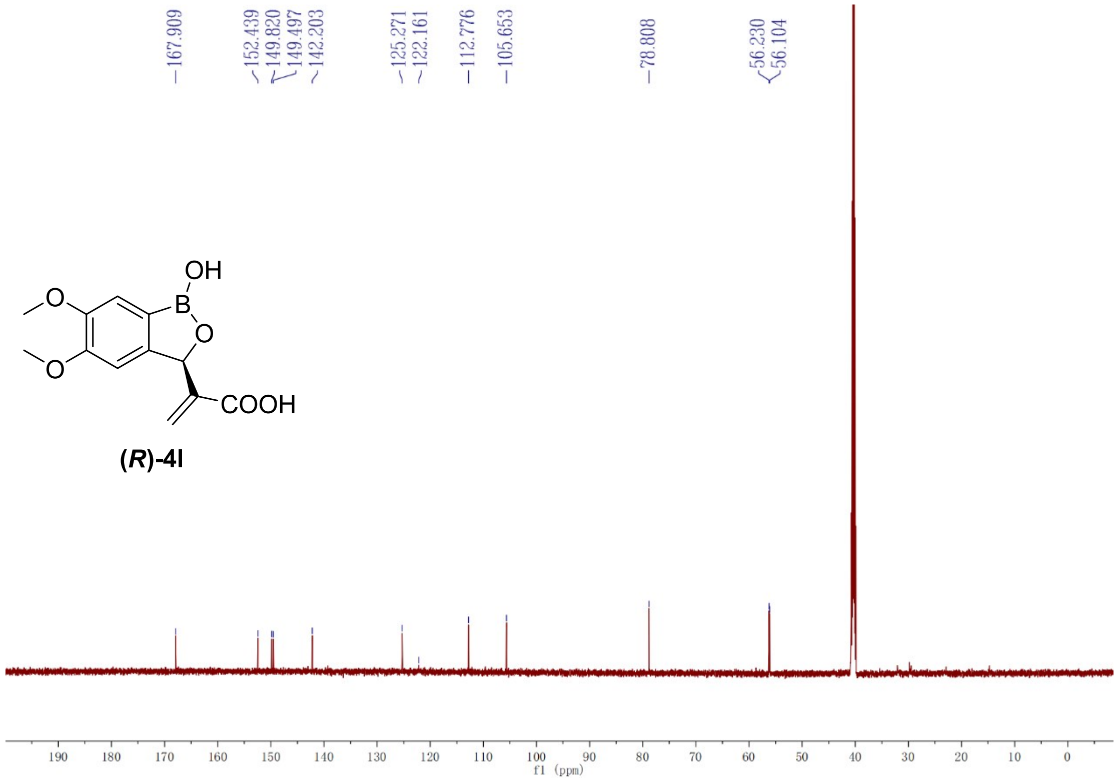

^{1}H NMR Spectra of (*R*)-**4m** (400 MHz, CD_3OD)

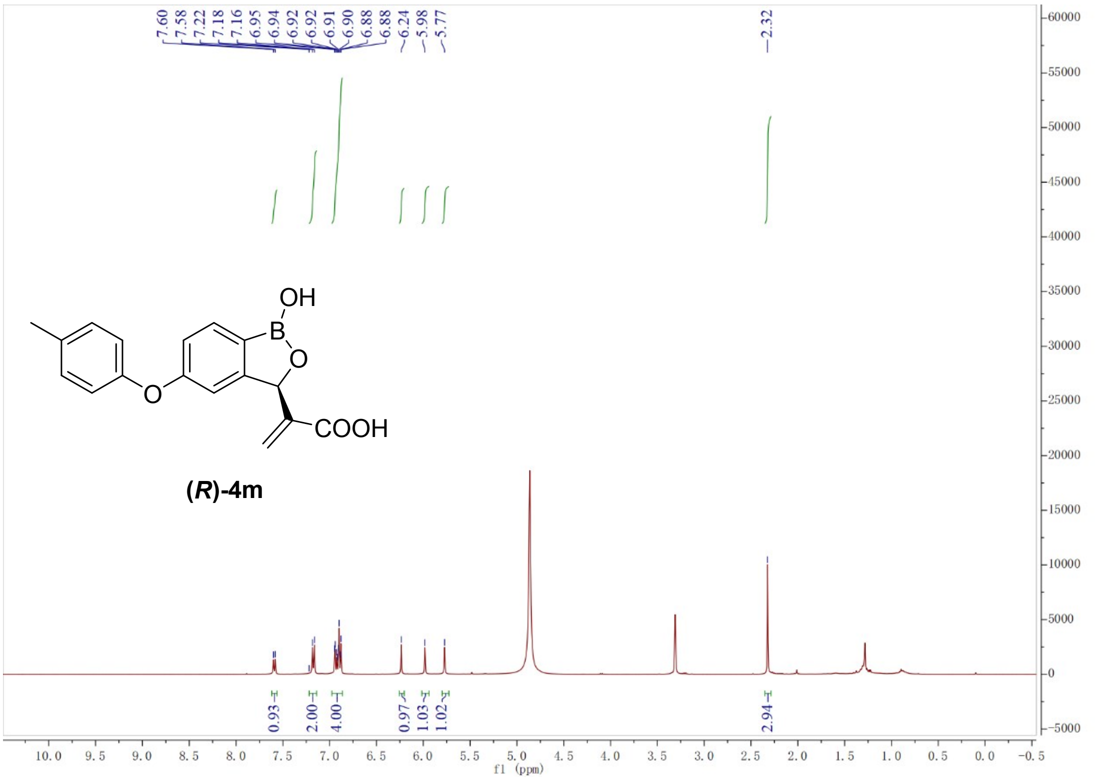

^{13}C NMR Spectra of (*R*)-**4m** (100 MHz, CD_3OD)

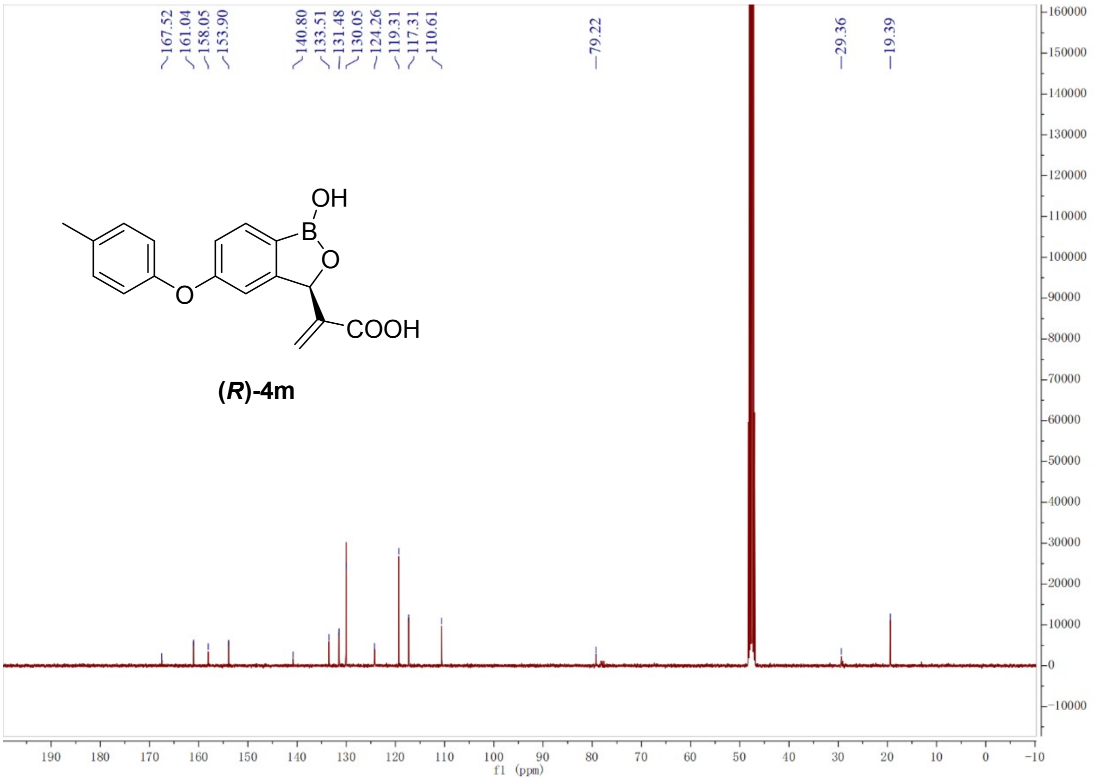

^{1}H NMR Spectra of (*R*)-**4n** (400 MHz, CD_3OD)

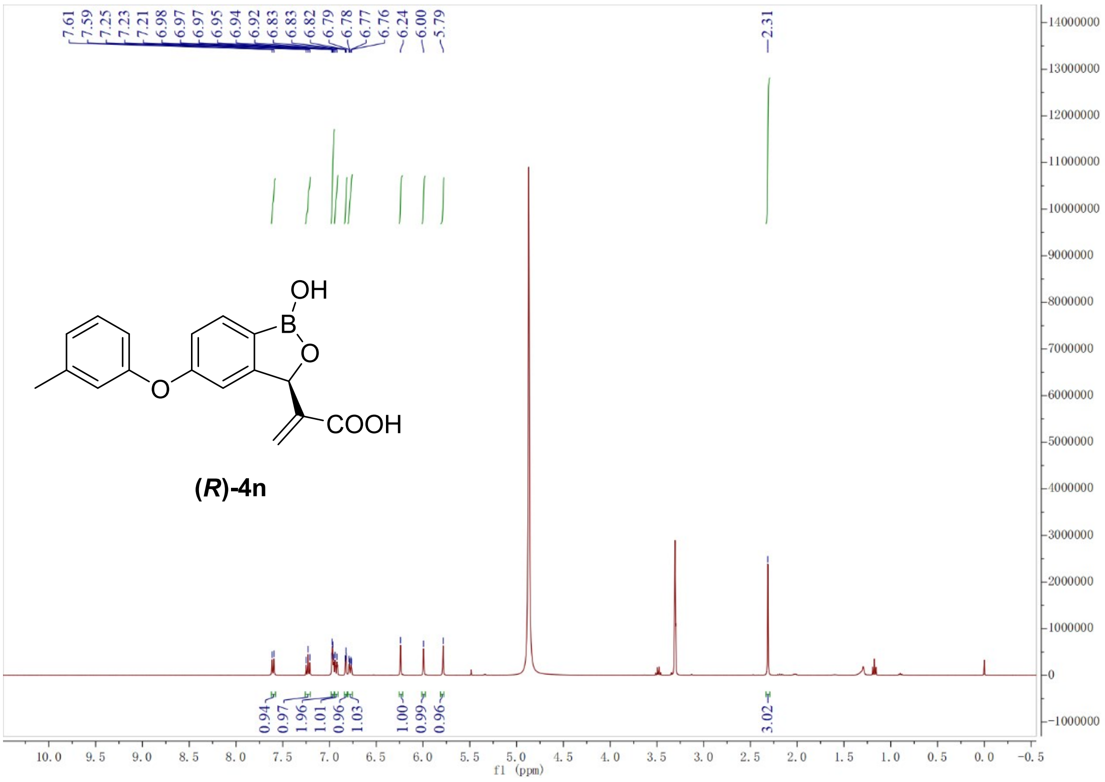

^{13}C NMR Spectra of (*R*)-**4n** (100 MHz, CD_3OD)

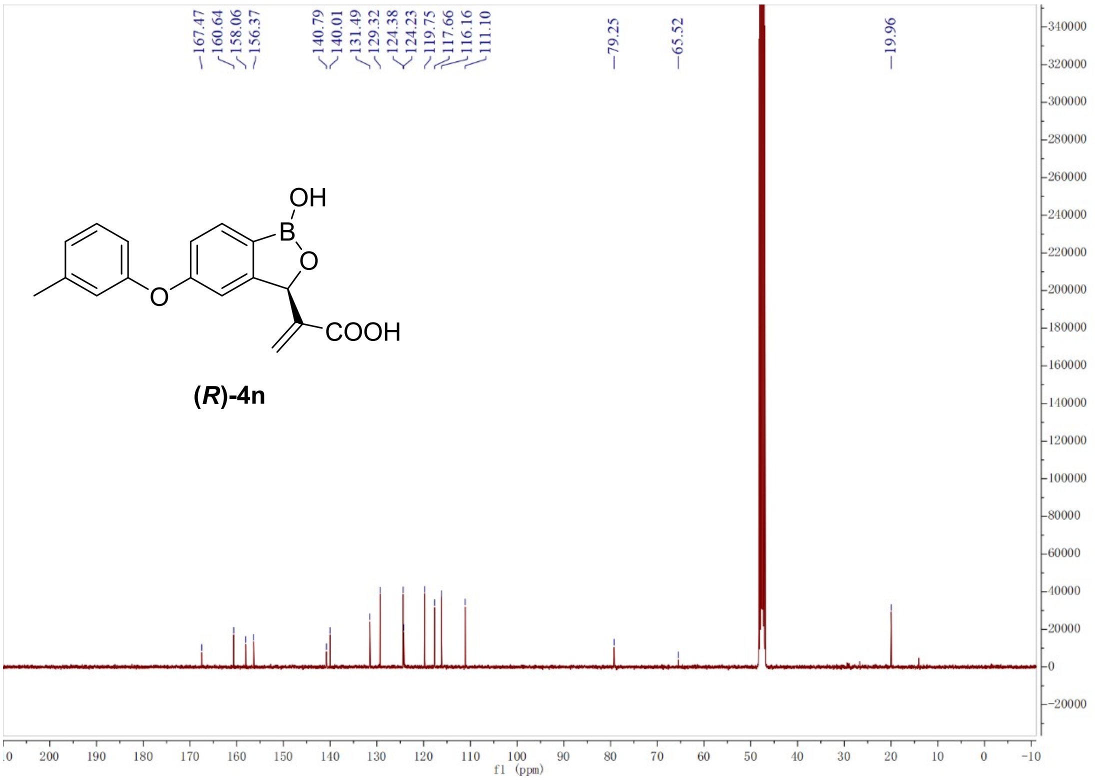

^{1}H NMR Spectra of (*R*)-**4o** (400 MHz, CD_3OD)

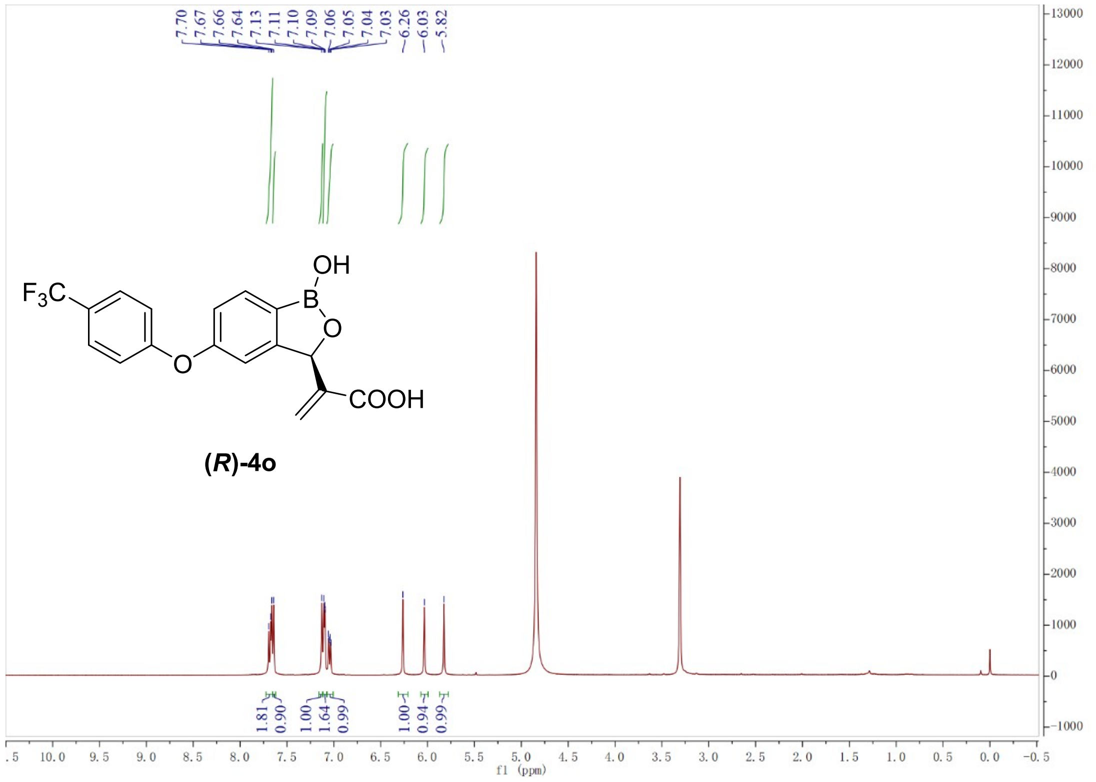

^{13}C NMR Spectra of (*R*)-**4o** (100 MHz, CD_3OD)

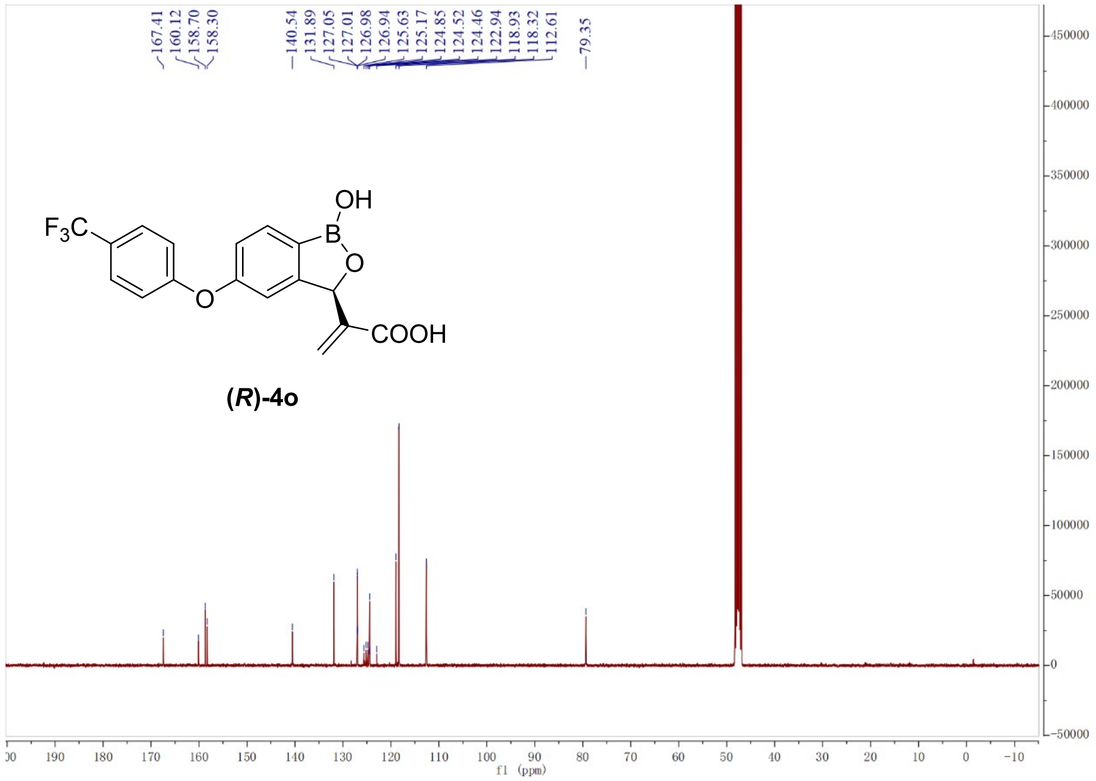

^{1}H NMR Spectra of (*R*)-**4p** (400 MHz, CD_3OD)

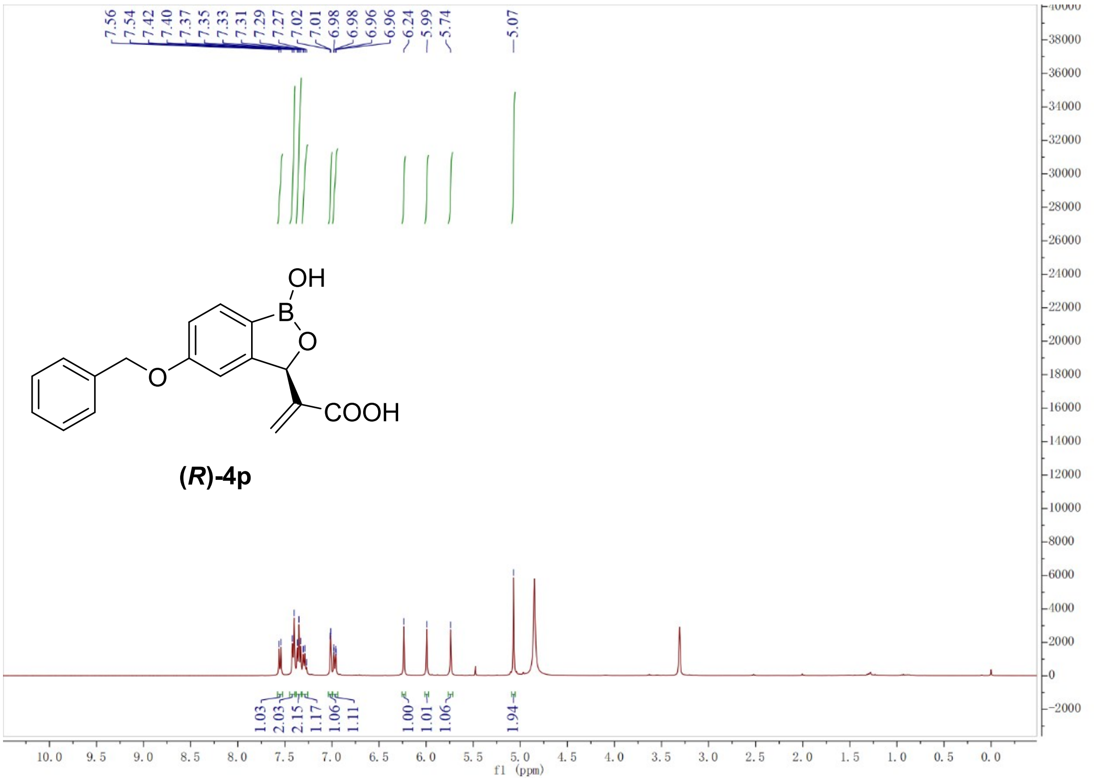

^{13}C NMR Spectra of (*R*)-**4p** (100 MHz, CD_3OD)

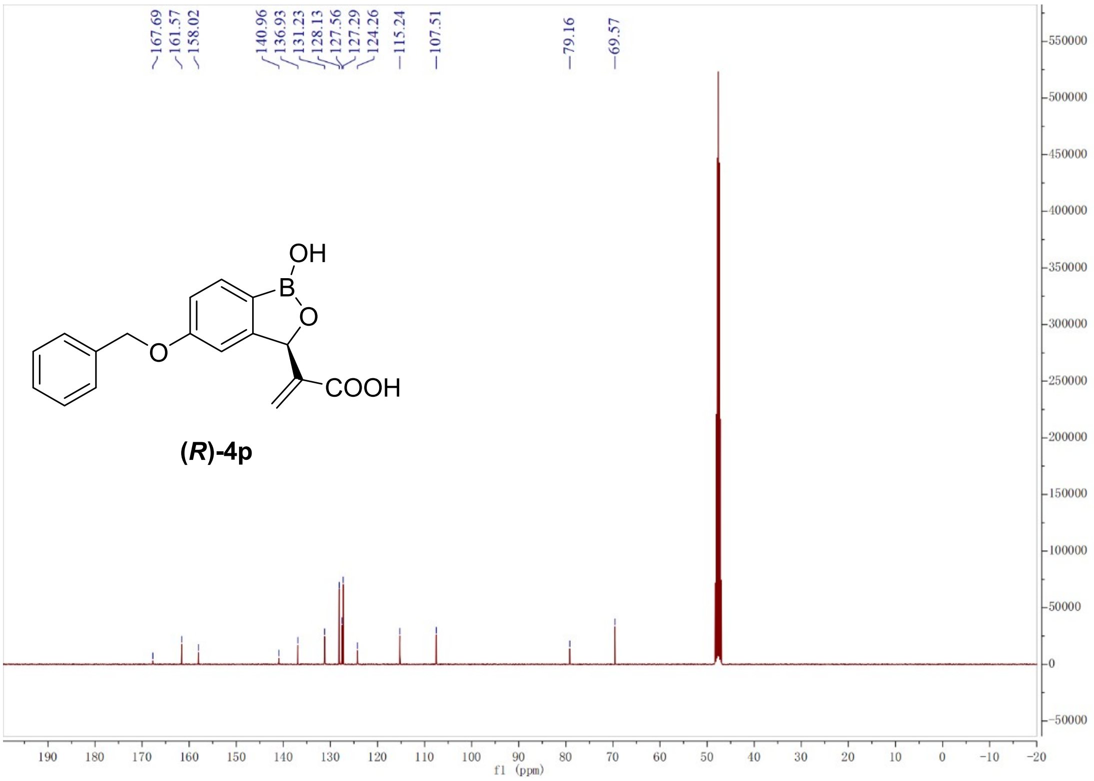

^{1}H NMR Spectra of (*R*)-**4q** (400 MHz, CD_3OD)

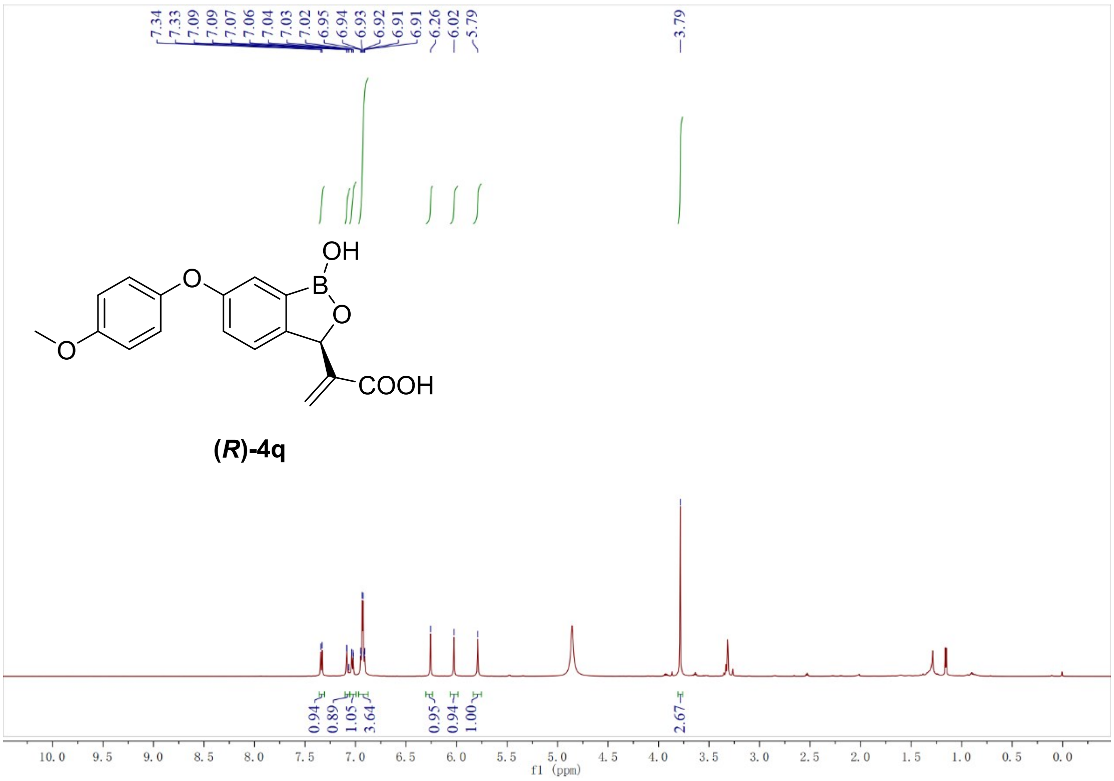

^{13}C NMR Spectra of (*R*)-**4q** (100 MHz, CD_3OD)

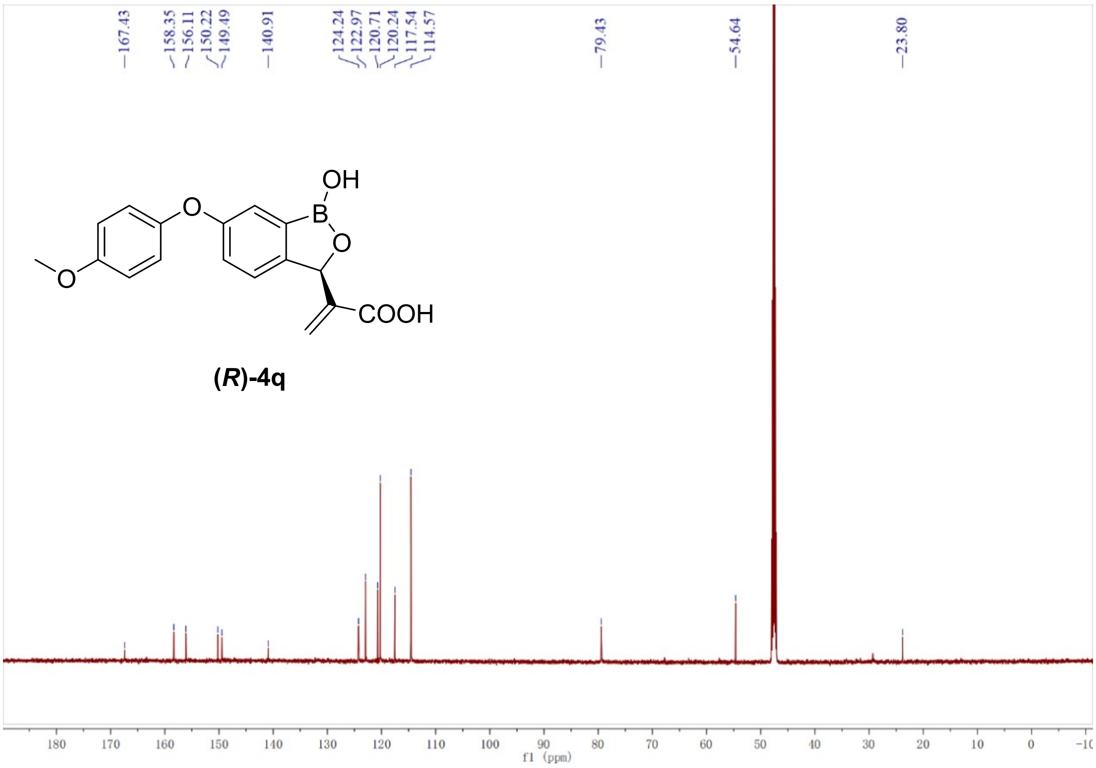

^{1}H NMR Spectra of (*R*)-**4r** (400 MHz, CD_3OD)

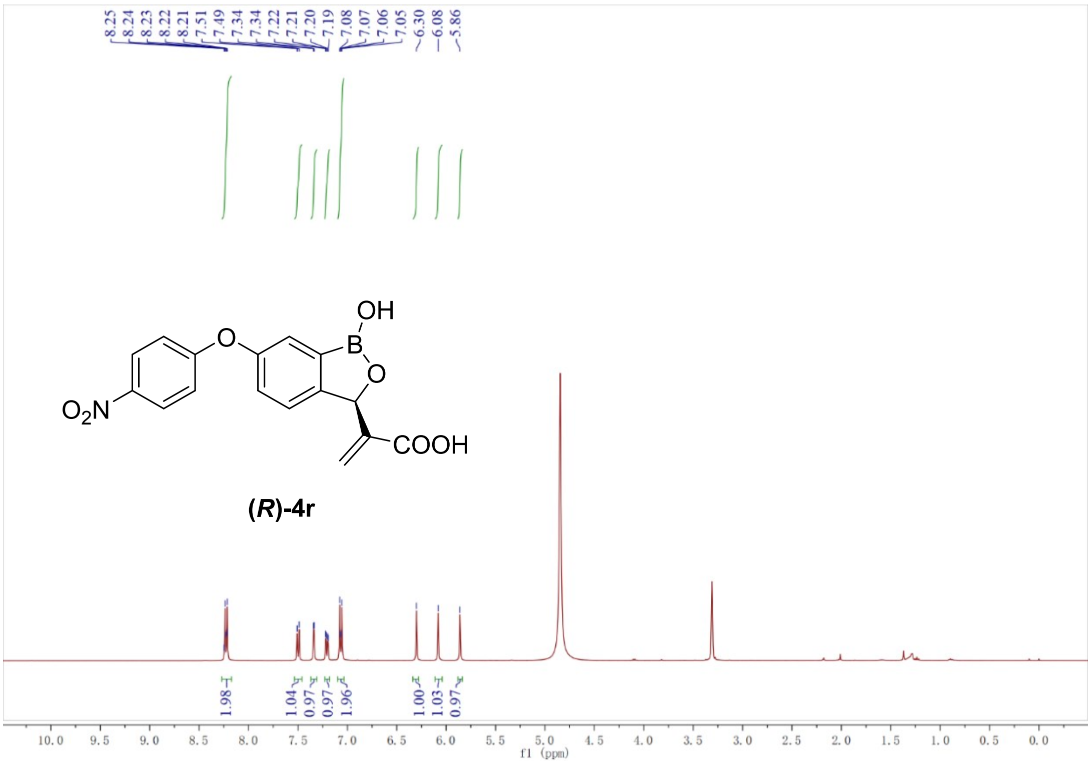

^{13}C NMR Spectra of (*R*)-**4r** (100 MHz, CD_3OD)

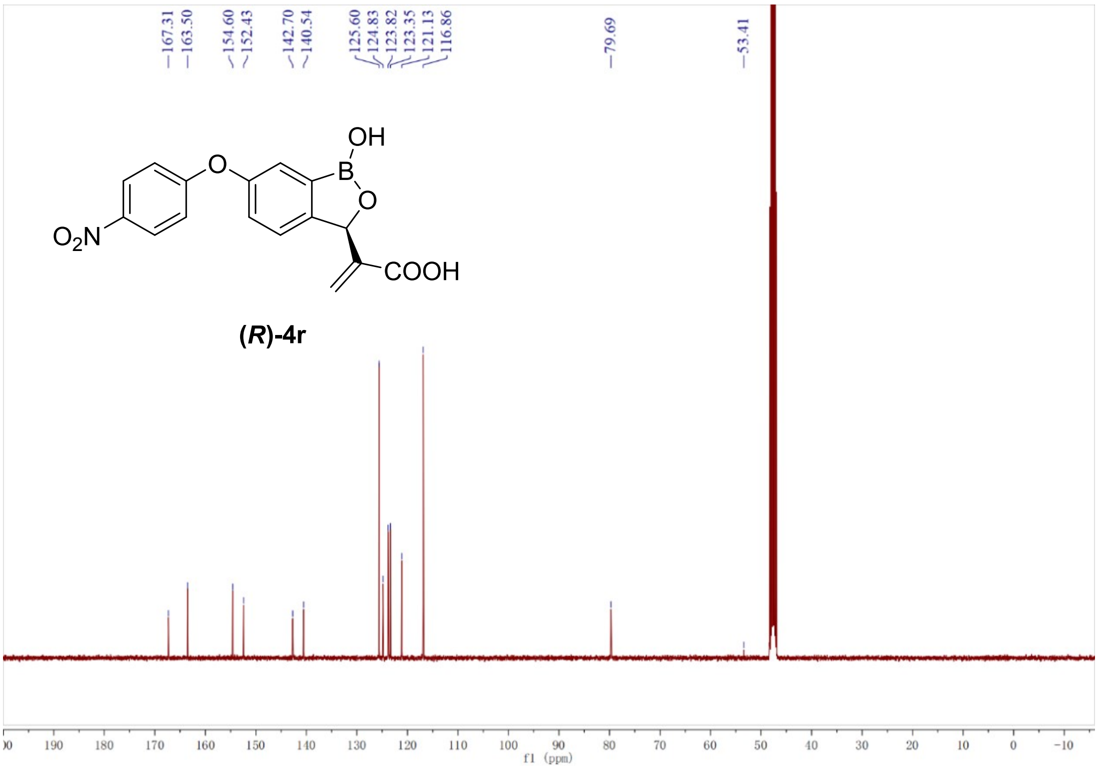

^{1}H NMR Spectra of (*R*)-**4s** (400 MHz, CD_3OD)

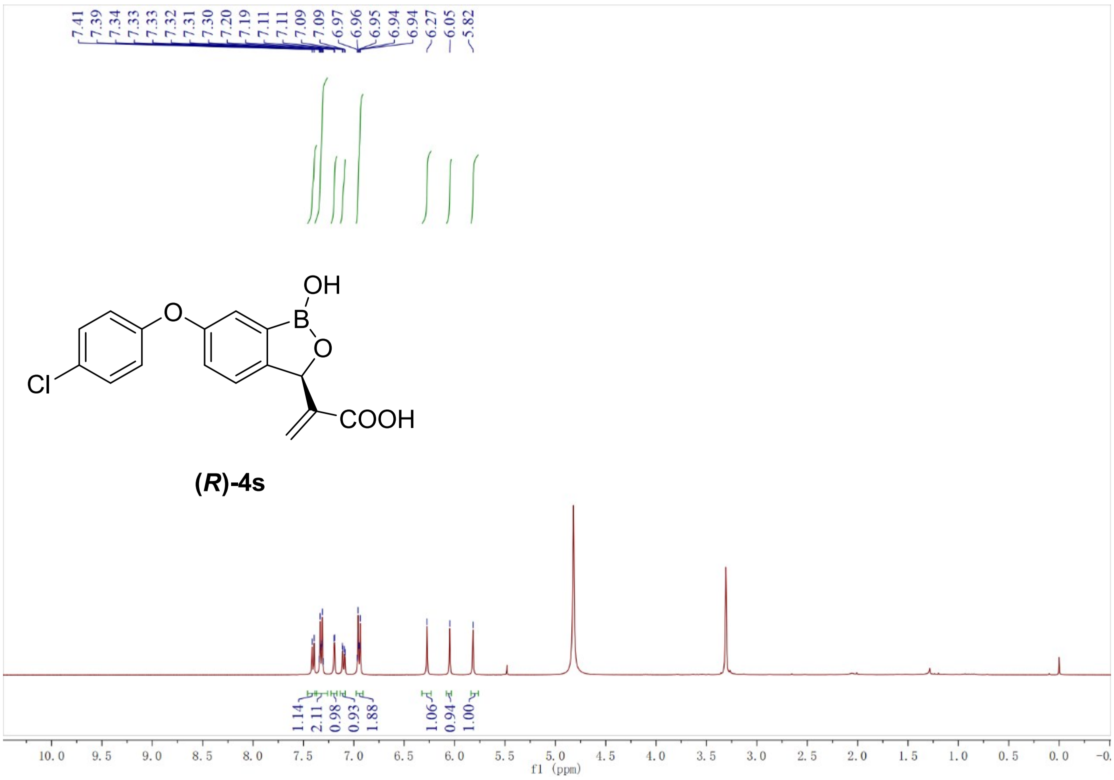

^{13}C NMR Spectra of (*R*)-**4s** (100 MHz, CD_3OD)

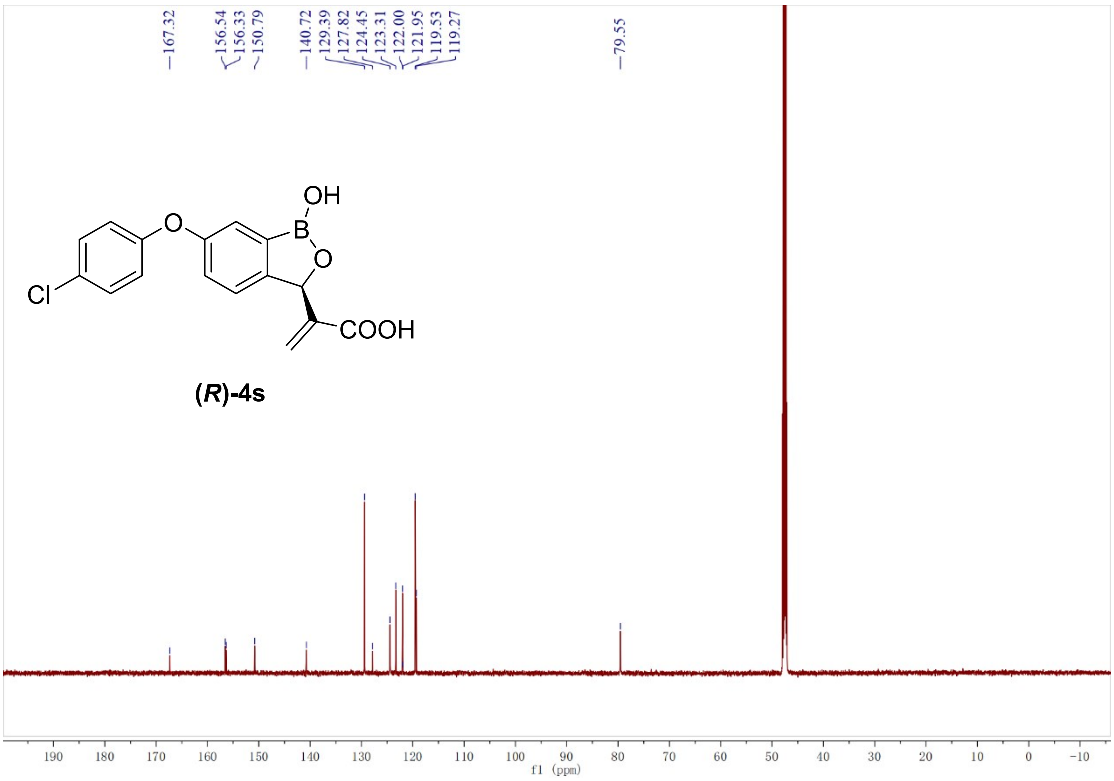

^{1}H NMR Spectra of (*R*)-**4t** (400 MHz, CD_3OD)

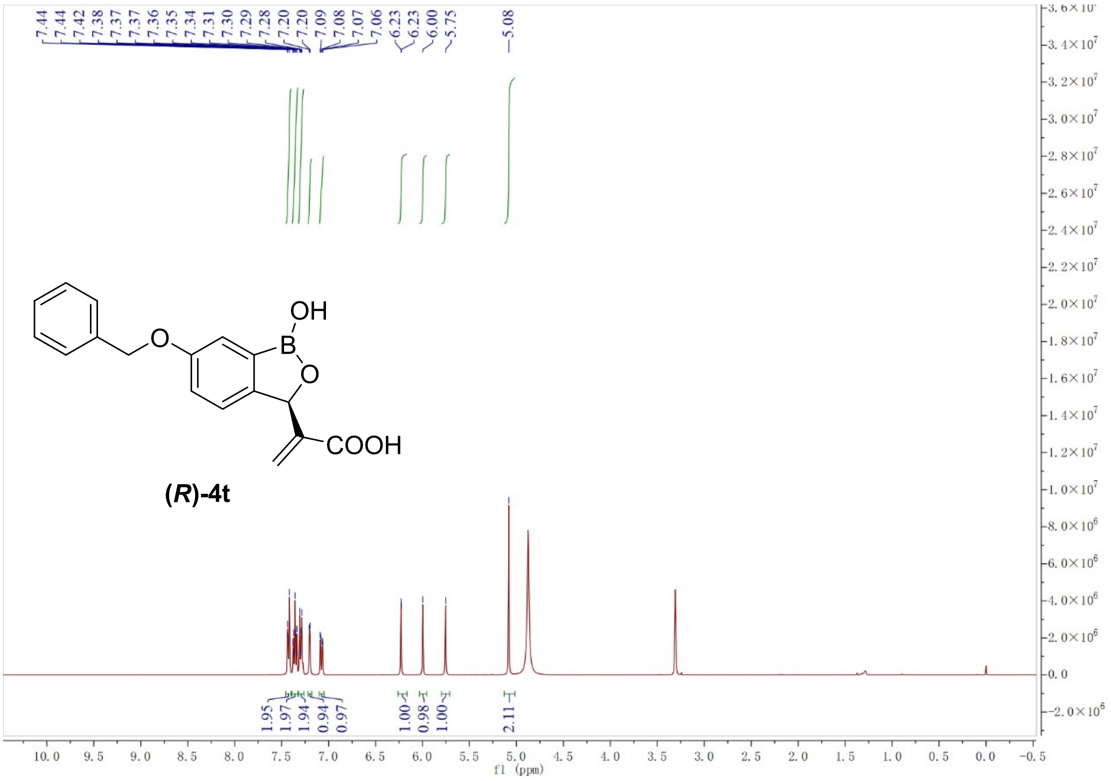

^{13}C NMR Spectra of (*R*)-**4t** (100 MHz, CD_3OD)

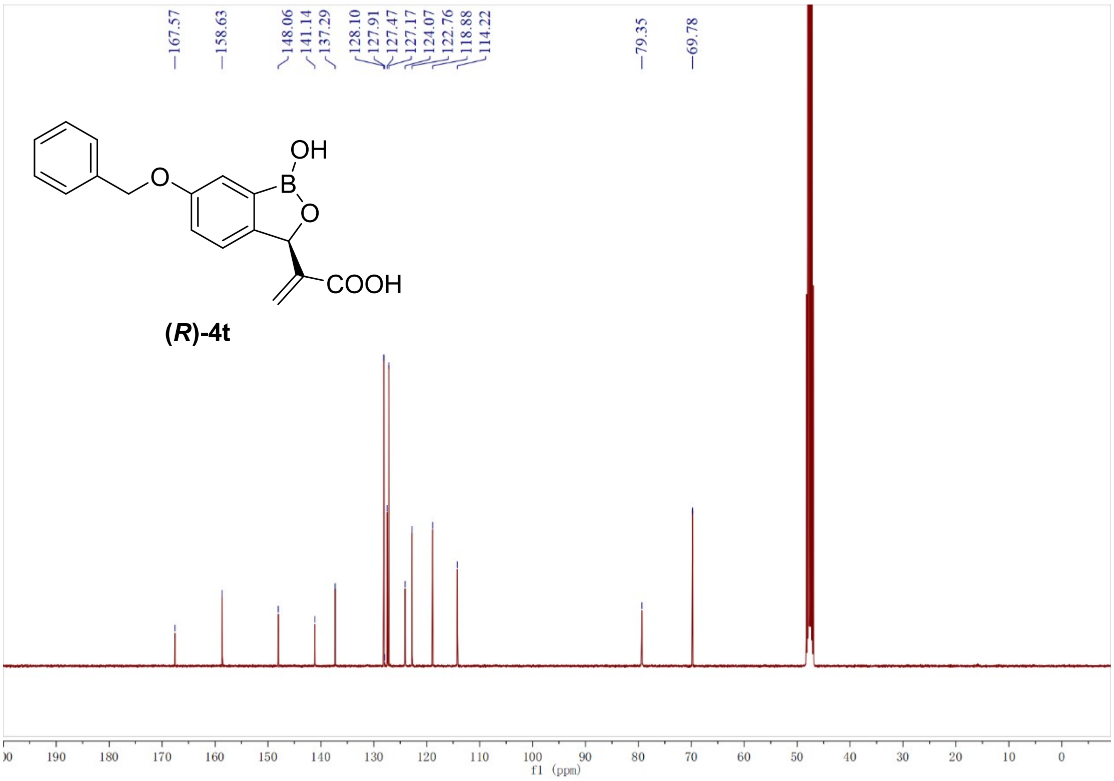

Copies of HPLC Spectra

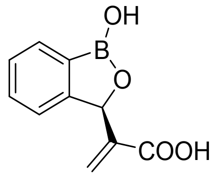

(*R*)-**4a**

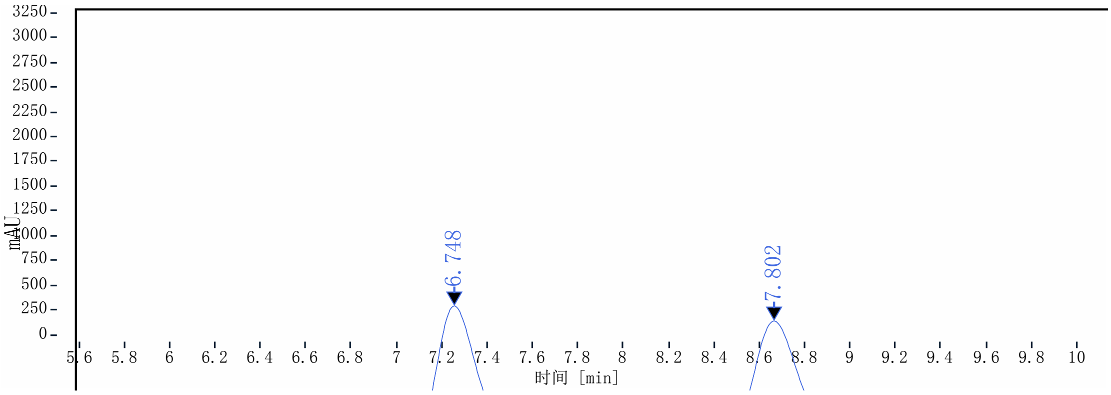

Ret Time[min]	Width [min]	Area	Height	Area %
6.748	1.03	10177.34	1050.21	49.96
7.802	0.96	10192.04	933.02	50.04
	Totals	20369.38		

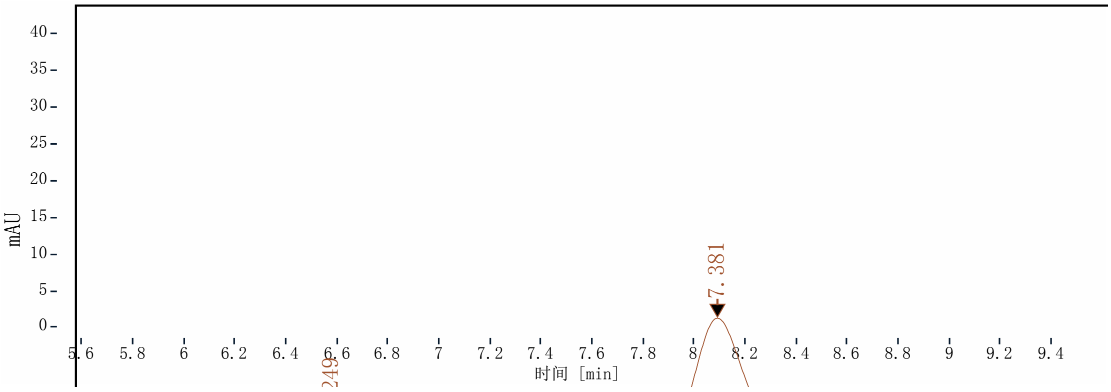

Ret Time[min]	Width [min]	Area	Height	Area %
6.249	0.12	6.78	0.82	5.00
7.381	0.16	128.78	12.53	95.00
	Totals	135.56		

ee after recrystallization

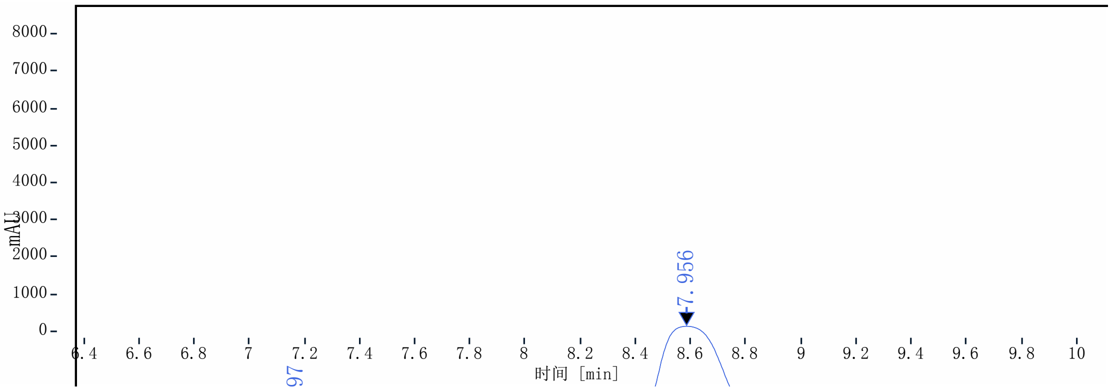

Ret Time[min]	Width [min]	Area	Height	Area %
6.897	0.14	614.85	70.79	1.93
7.956	1.33	31237.25	2373.52	98.07
	Totals	31852.10		

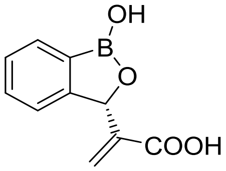

(*S*)-4a

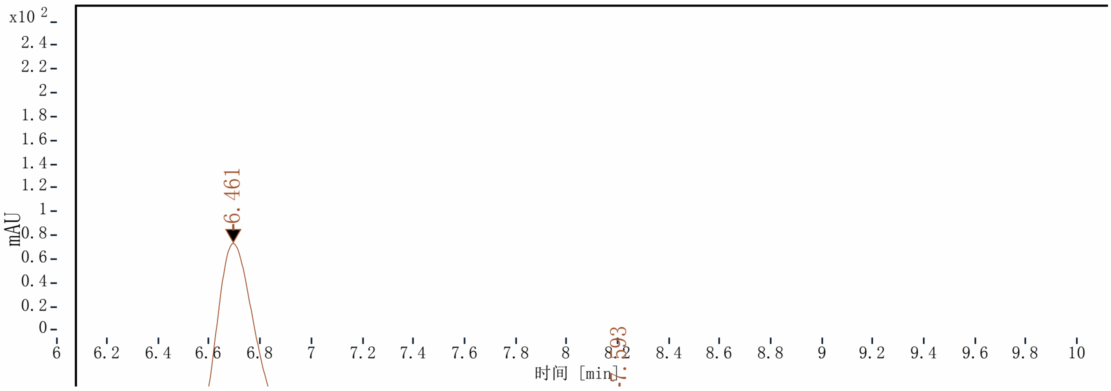

Ret Time[min]	Width [min]	Area	Height	Area %
6.461	0.13	1052.24	125.41	79.51
7.593	0.16	271.08	25.74	20.49
	Totals	1323.32		

ee after recrystallization

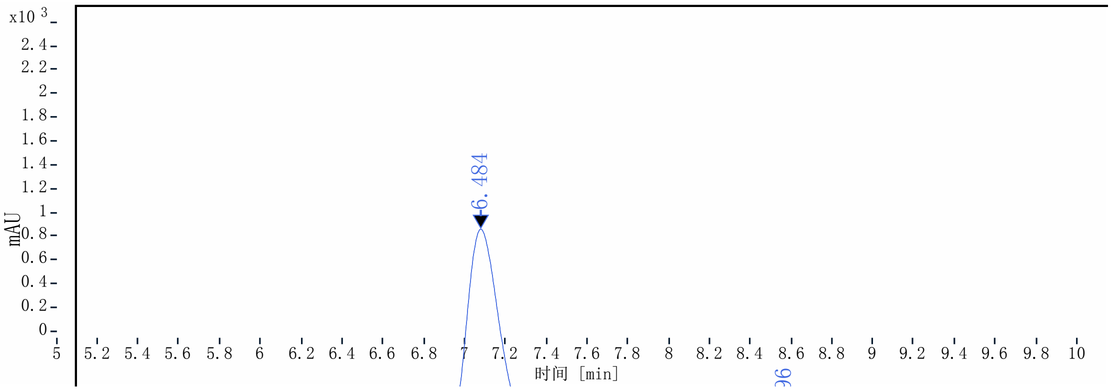

Ret Time[min]	Width [min]	Area	Height	Area %
6.484	0.14	11901.17	1351.69	99.76
7.596	0.14	28.53	3.22	0.24
	Totals	11929.70		

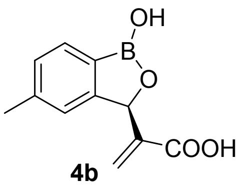

4b

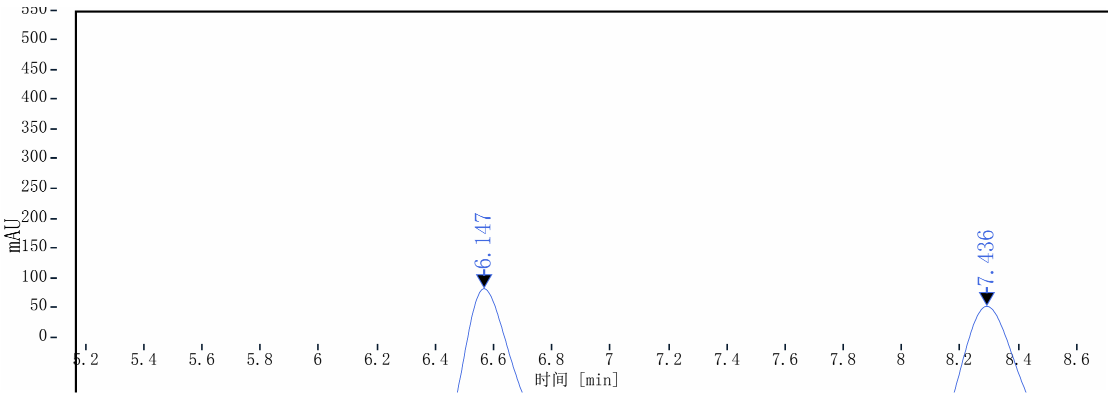

Ret Time[min]	Width [min]	Area	Height	Area %
6.147	0.14	1925.58	206.89	49.04
7.436	0.16	2000.68	186.12	50.96
	Totals	3926.26		

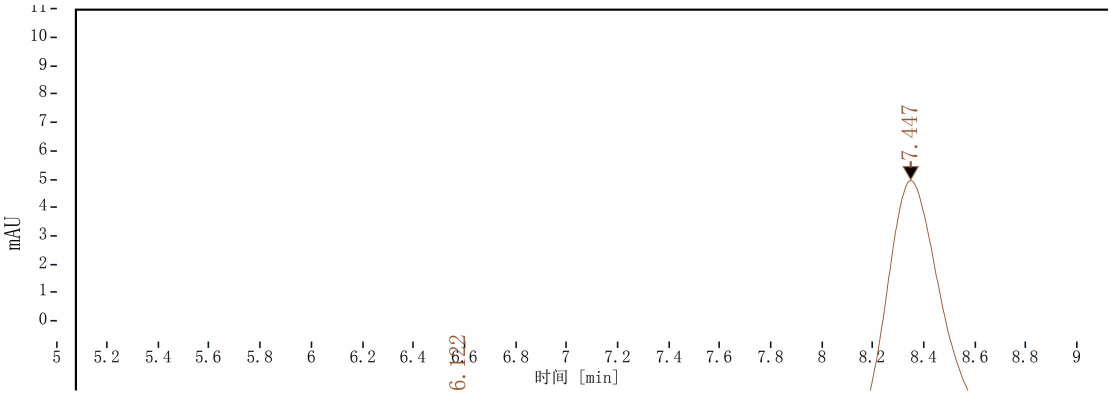

Ret Time[min]	Width [min]	Area	Height	Area %
6.122	0.10	4.26	0.57	5.57
7.447	0.17	72.30	6.56	94.43
	Totals	76.56		

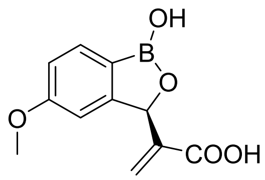

(*R*)-**4c**

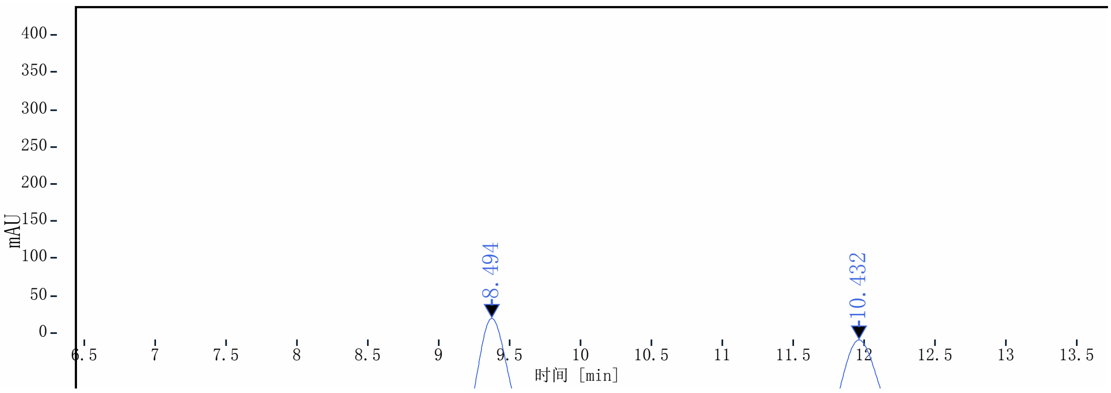

Ret Time[min]	Width [min]	Area	Height	Area %
8.494	0.19	1562.94	129.80	49.06
10.432	0.23	1622.51	107.44	50.94
	Totals	3185.45		

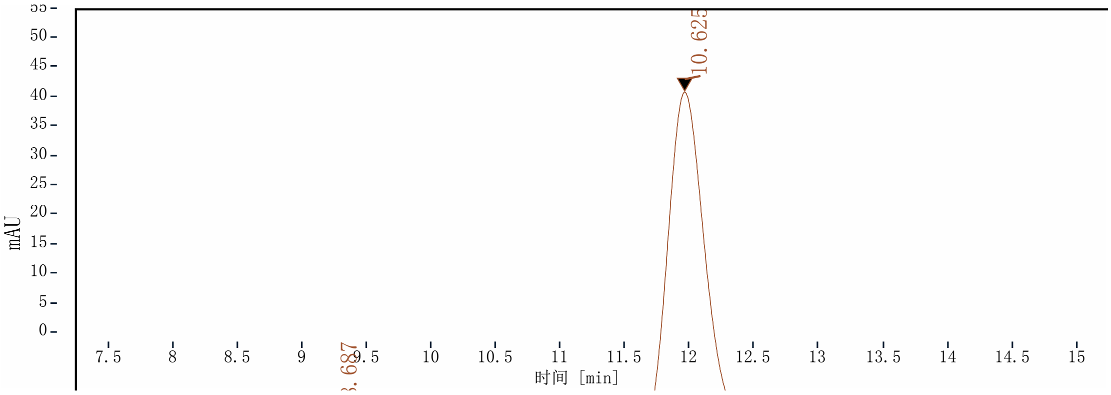

Ret Time[min]	Width [min]	Area	Height	Area %
8.687	0.17	37.15	3.16	5.00
10.625	0.24	706.04	44.56	95.00
	Totals	749.19		

ee after recrystallization

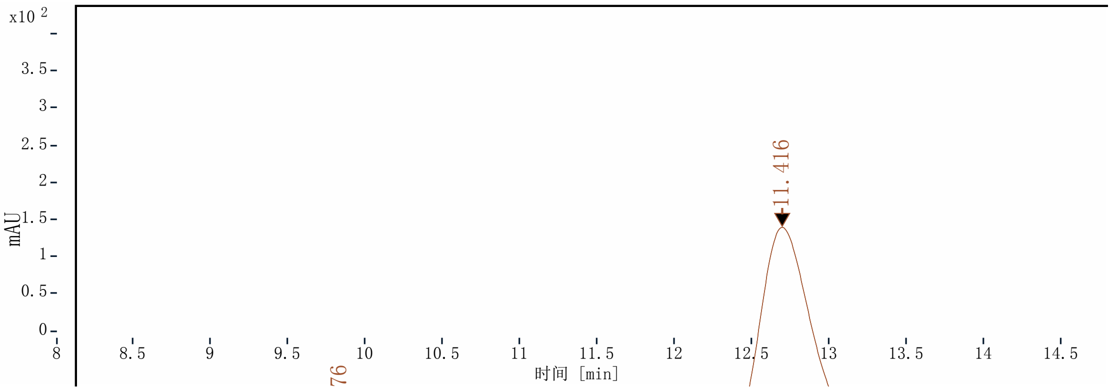

Ret Time[min]	Width [min]	Area	Height	Area %
9.276	0.20	42.28	3.29	1.05
11.416	0.28	3970.27	218.23	98.95
	Totals	4012.55		

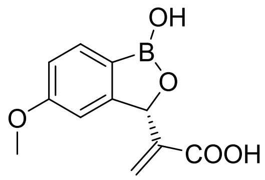

(*S*)-**4c**

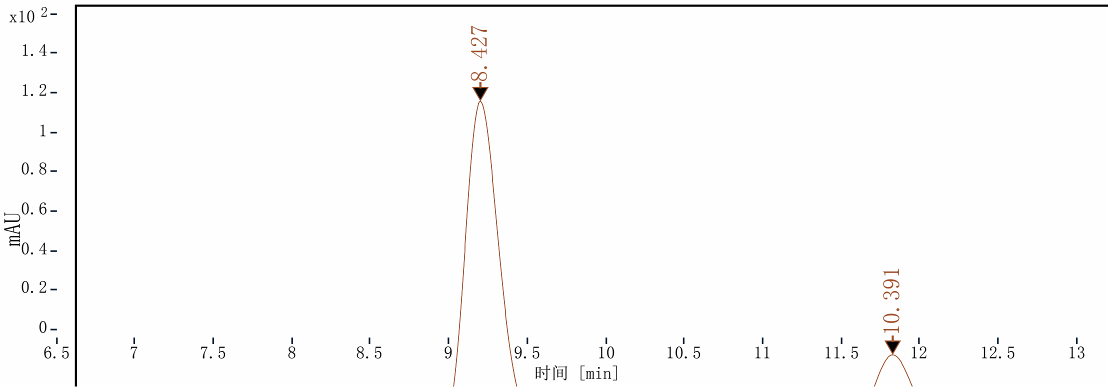

Ret Time[min]	Width [min]	Area	Height	Area %
8.427	0.18	1515.55	128.46	75.99
10.391	0.22	478.73	32.90	24.01
	Totals	1994.28		

ee after recrystallization

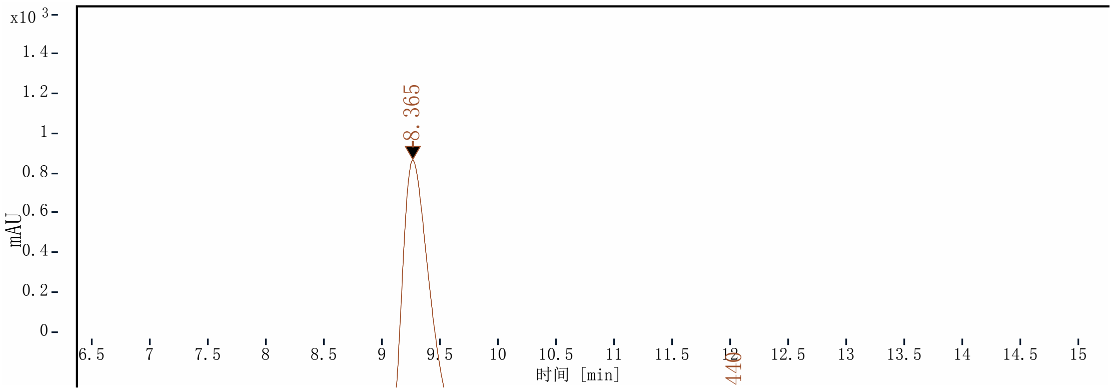

Ret Time[min]	Width [min]	Area	Height	Area %
8.365	0.19	13706.72	1069.99	97.93
10.440	0.21	289.55	21.43	2.07
	Totals	13996.27		

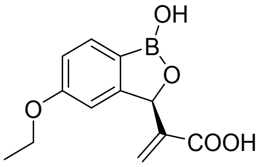

4d

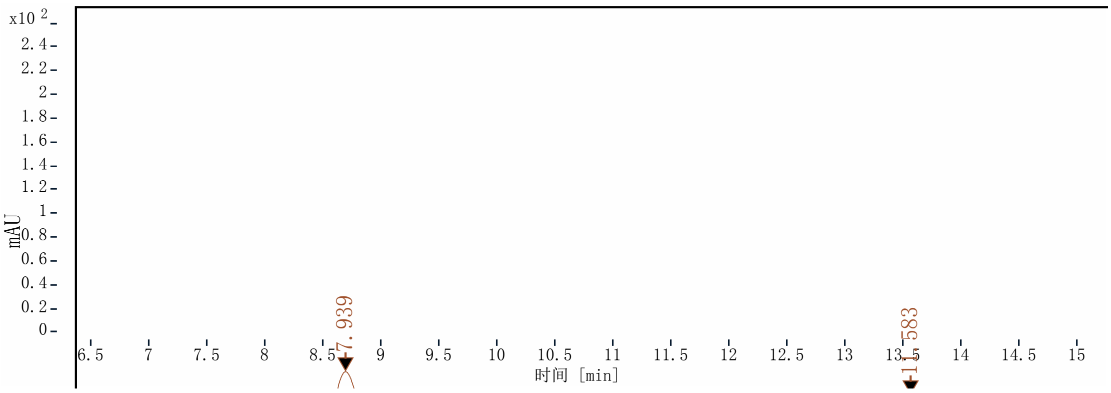

Ret Time[min]	Width [min]	Area	Height	Area %
7.939	0.18	546.71	46.79	49.86
11.583	0.26	549.86	32.53	50.14
	Totals	1096.57		

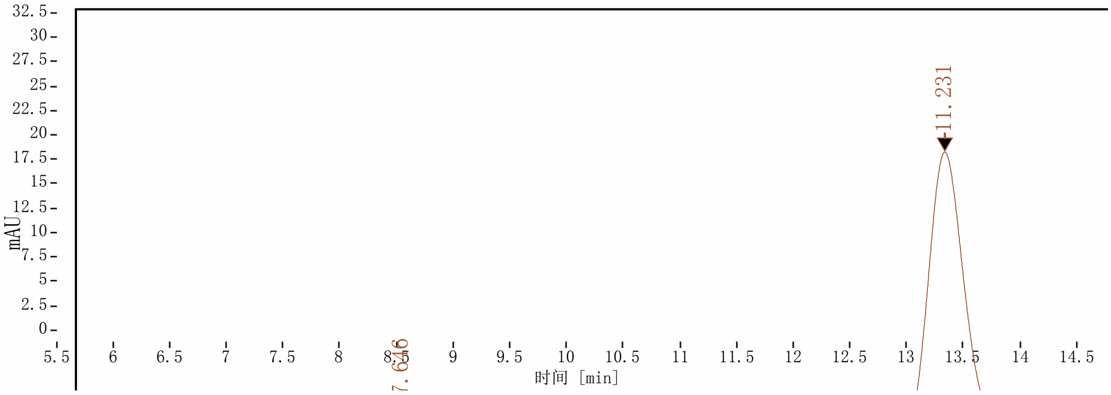

Ret Time[min]	Width [min]	Area	Height	Area %
7.646	0.16	28.78	2.44	6.93
11.231	0.26	386.35	22.46	93.07
	Totals	415.13		

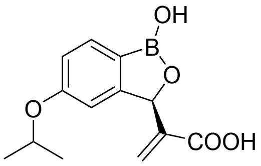

4e

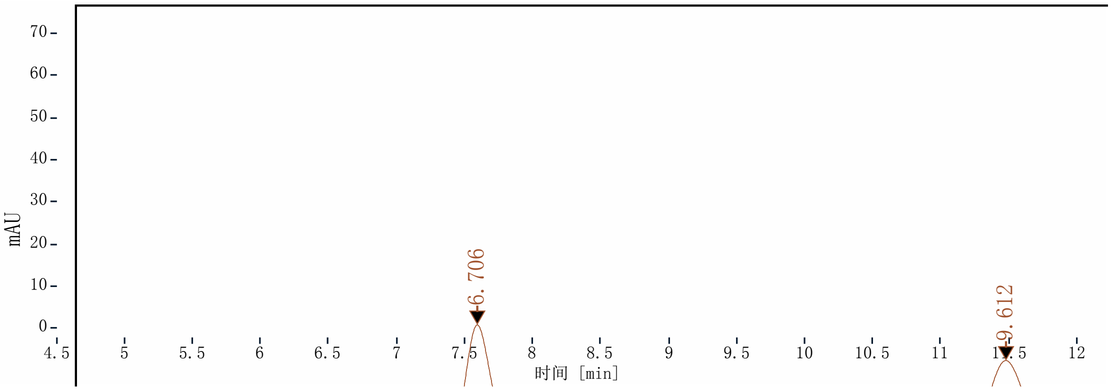

Ret Time[min]	Width [min]	Area	Height	Area %
6.706	0.15	211.99	20.84	50.15
9.612	0.22	210.71	14.70	49.85
	Totals	422.70		

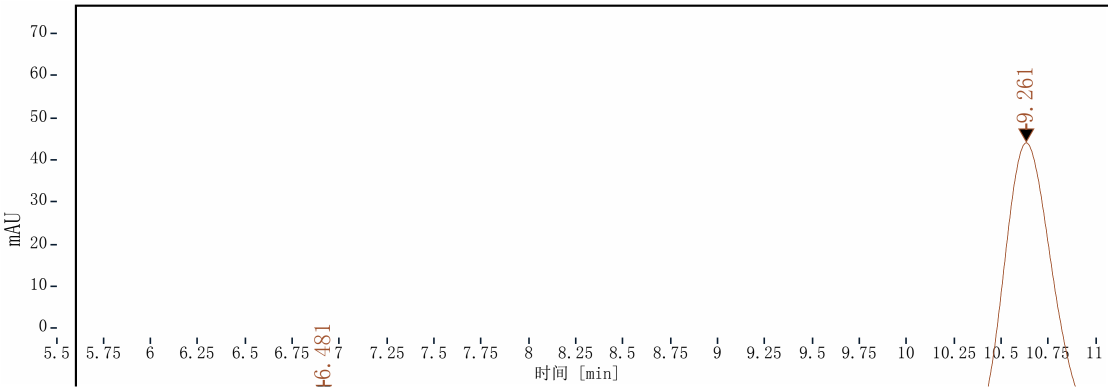

Ret Time[min]	Width [min]	Area	Height	Area %
6.481	0.15	74.05	7.48	8.90
9.261	0.22	757.63	53.09	91.10
	Totals	831.68		

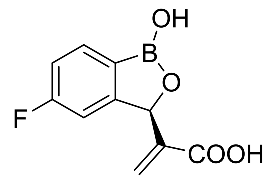

(*R*)-**4f**

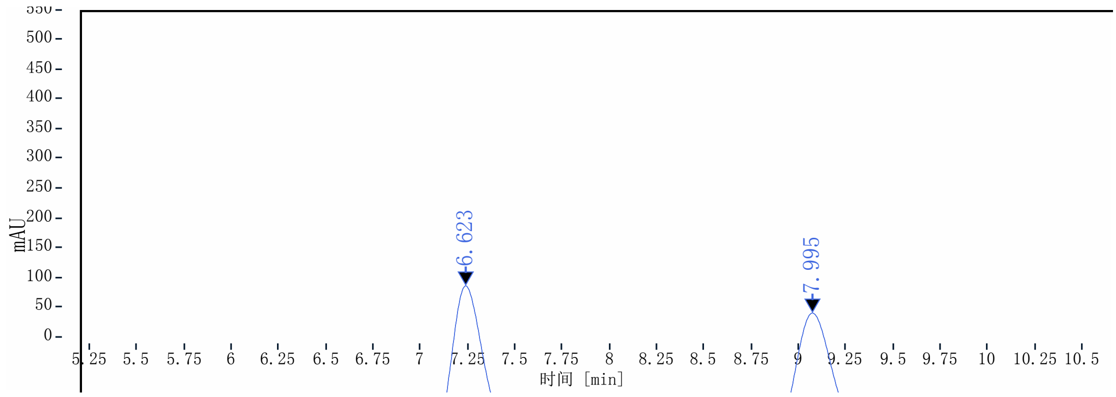

Ret Time[min]	Width [min]	Area	Height	Area %
6.623	0.14	1922.77	205.11	50.30
7.995	0.17	1899.57	171.35	49.70
	Totals	3822.35		

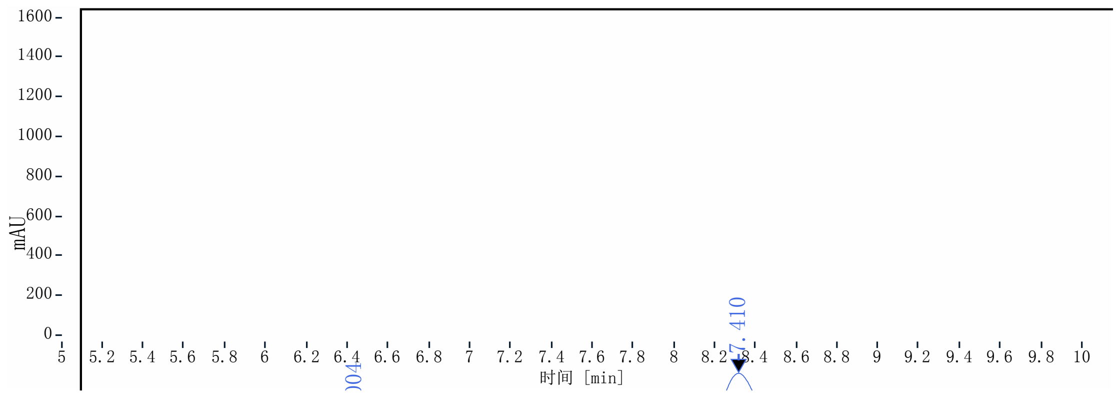

Ret Time[min]	Width [min]	Area	Height	Area %
6.004	0.10	269.72	42.28	8.35
7.410	0.69	2961.22	285.61	91.65
	Totals	3230.94		

ee after recrystallization

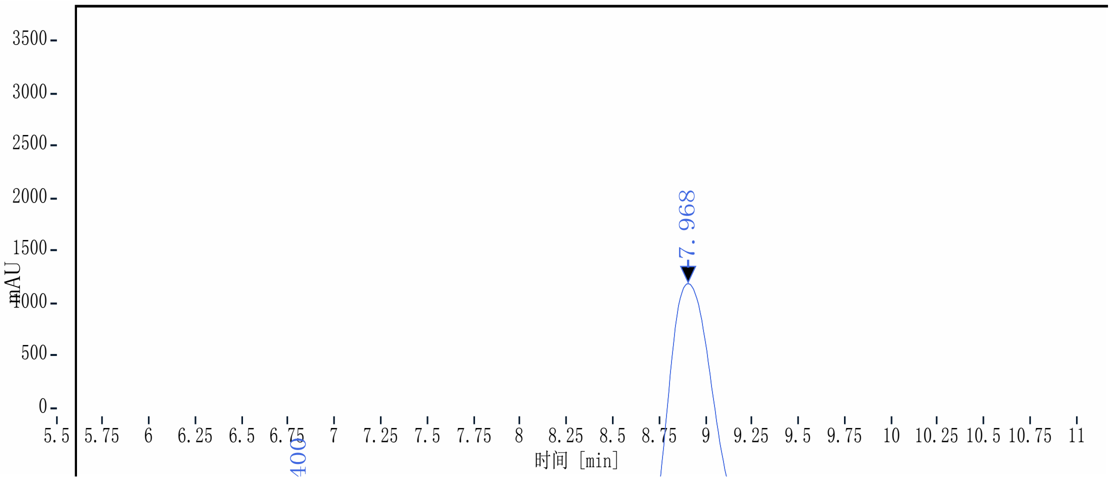

Ret Time[min]	Width [min]	Area	Height	Area %
6.400	0.16	1298.60	127.23	5.04
7.968	0.20	24447.96	1882.83	94.96
	Totals	25746.56		

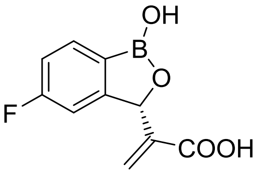

(*S*)-**4f**

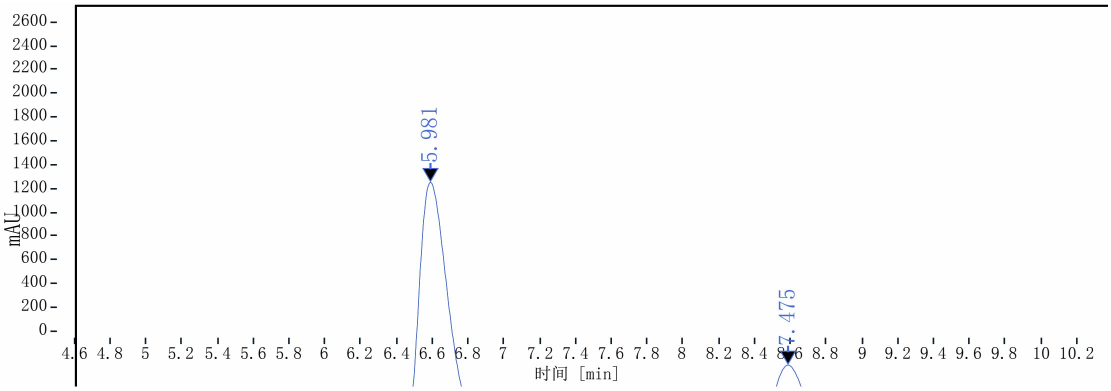

Ret Time[min]	Width [min]	Area	Height	Area %
5.981	0.13	14281.17	1636.12	73.87
7.475	0.16	5050.51	493.90	26.13
	Totals	19331.68		

ee after recrystallization

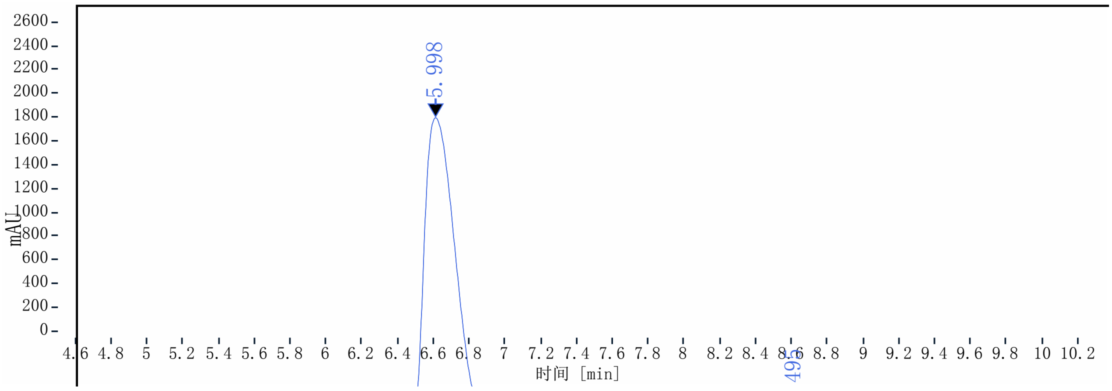

Ret Time[min]	Width [min]	Area	Height	Area %
5.998	0.15	19521.87	2040.77	94.81
7.495	0.15	1068.83	112.68	5.19
	Totals	20590.70		

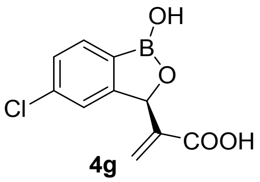

4g

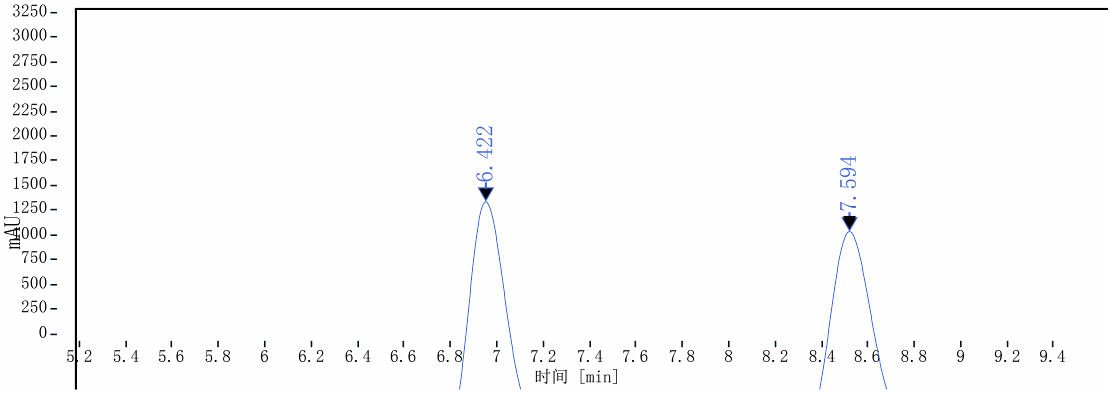

Ret Time[min]	Width [min]	Area	Height	Area %
6.422	0.14	16724.58	1852.31	50.56
7.594	0.16	16357.32	1615.85	49.44
	Totals	33081.90		

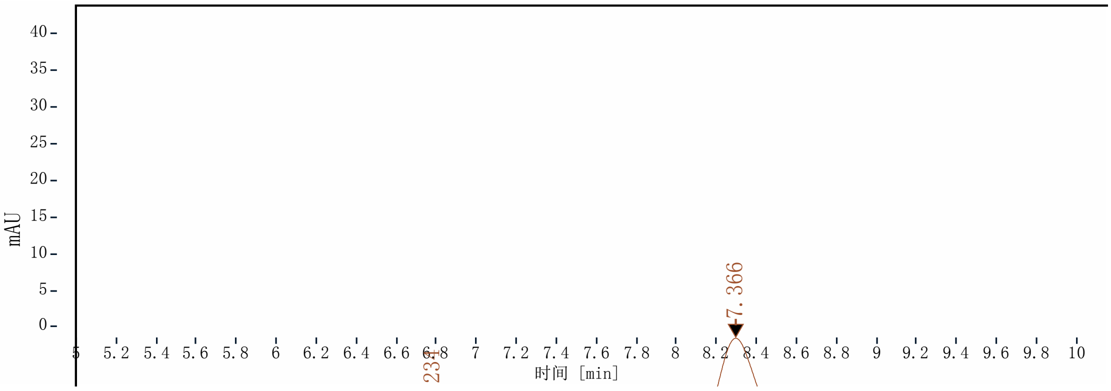

Ret Time[min]	Width [min]	Area	Height	Area %
6.234	0.13	15.48	1.71	12.42
7.366	0.16	109.20	10.53	87.58
	Totals	124.69		

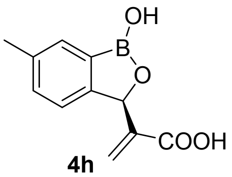

4h

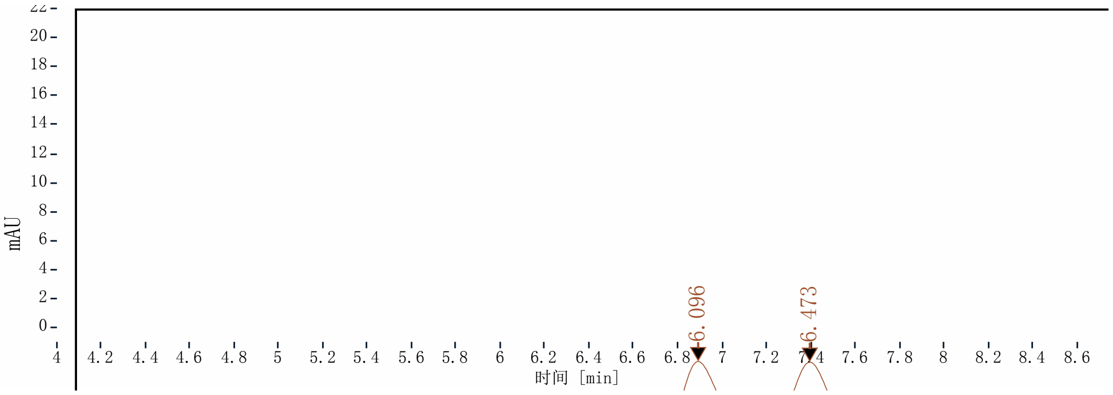

Ret Time[min]	Width [min]	Area	Height	Area %
6.096	0.13	33.33	3.89	49.11
6.473	0.14	34.53	3.85	50.89
	Totals	67.87		

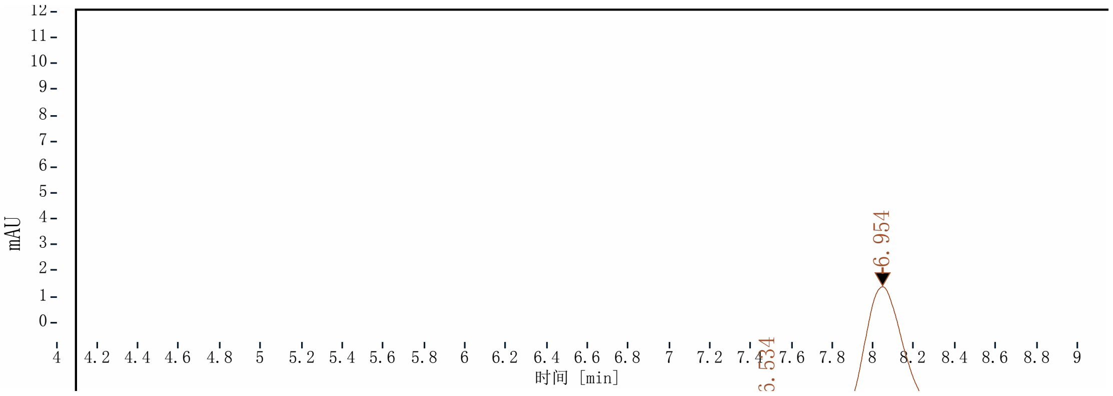

Ret Time[min]	Width [min]	Area	Height	Area %
6.534	0.10	2.66	0.31	6.03
6.954	0.16	41.48	3.94	93.97
	Totals	44.14		

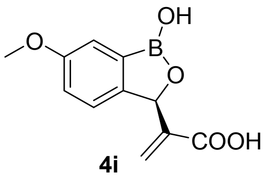

4i

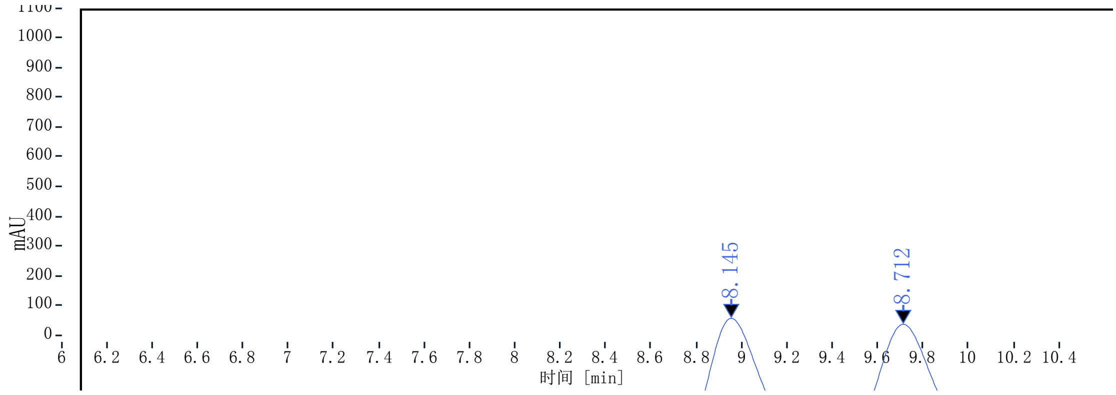

Ret Time[min]	Width [min]	Area	Height	Area %
8.145	0.19	3926.31	324.16	48.76
8.712	0.20	4125.93	308.11	51.24
	Totals	8052.24		

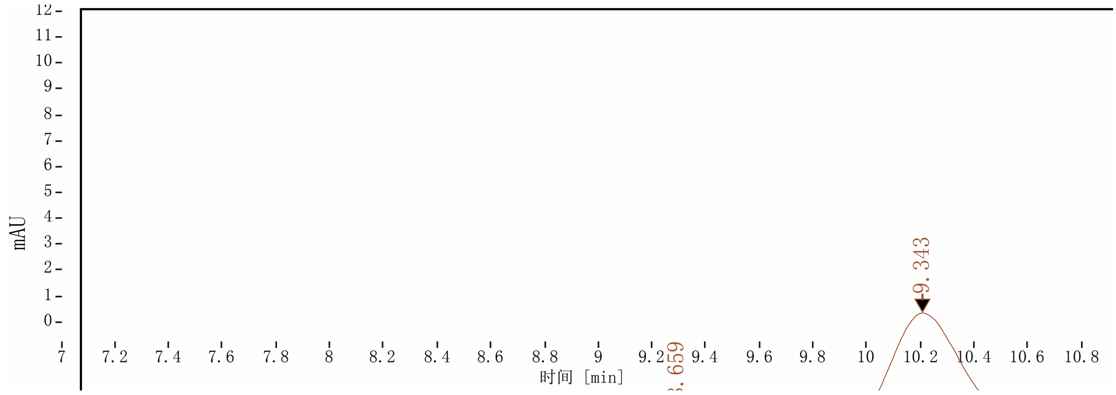

Ret Time[min]	Width [min]	Area	Height	Area %
8.659	0.14	4.19	0.36	7.92
9.343	0.20	48.67	3.36	92.08
	Totals	52.86		

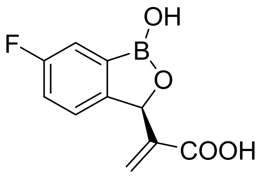

(*R*)-**4j**

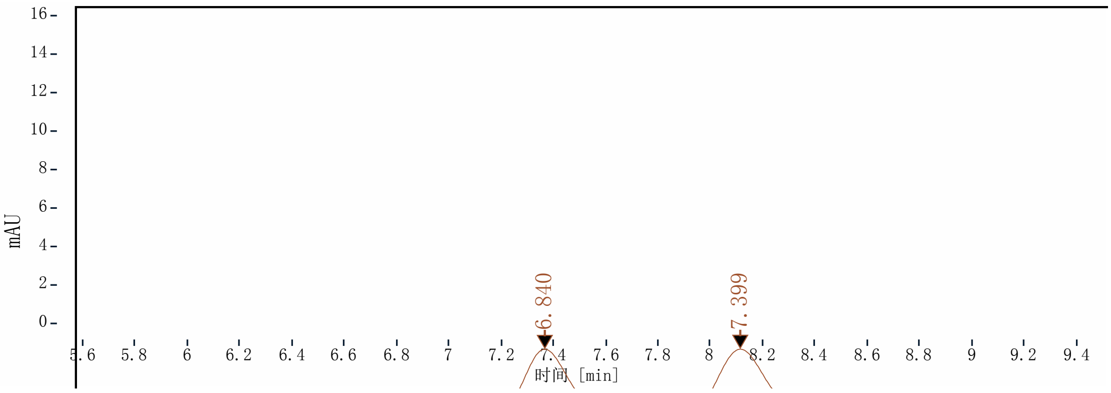

Ret Time[min]	Width [min]	Area	Height	Area %
6.840	0.15	27.11	2.75	49.16
7.399	0.16	28.04	2.72	50.84
	Totals	55.15		

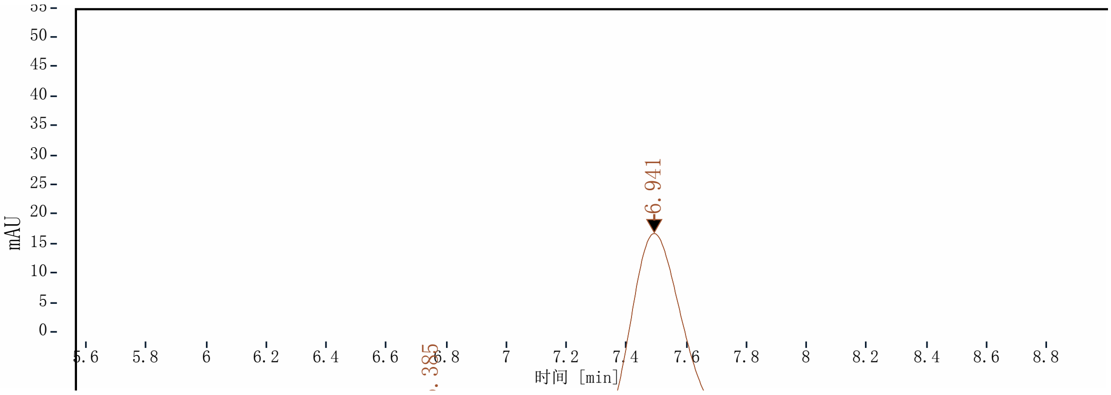

Ret Time[min]	Width [min]	Area	Height	Area %
6.385	0.14	27.03	3.01	9.47
6.941	0.15	258.38	26.41	90.53
	Totals	285.41		

ee after recrystallization

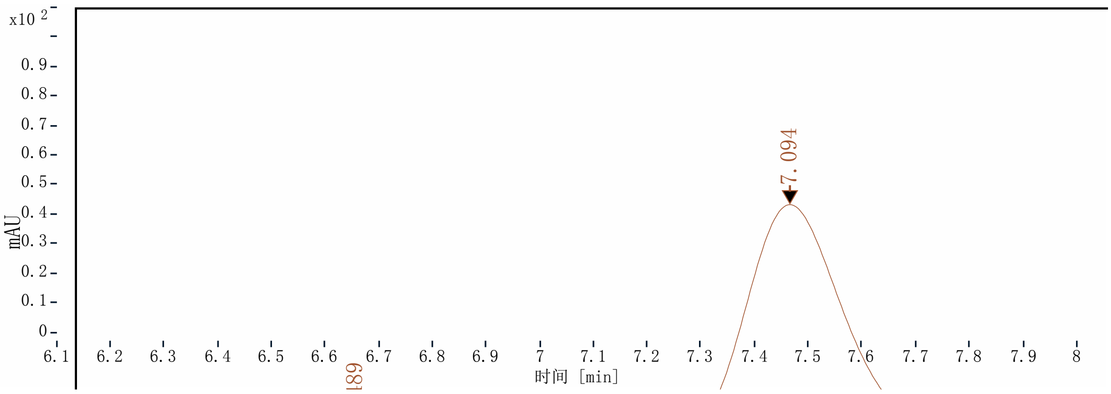

Ret Time[min]	Width [min]	Area	Height	Area %
6.489	0.13	11.74	1.34	1.94
7.094	0.15	594.49	60.10	98.06
	Totals	606.23		

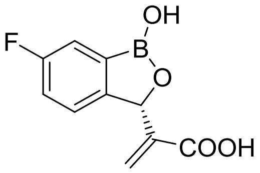

(*S*)-**4j**

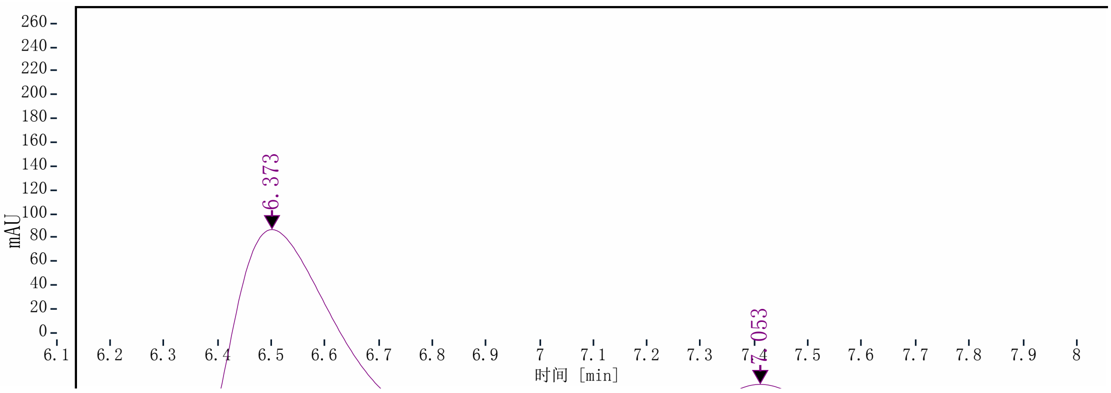

Ret Time[min]	Width [min]	Area	Height	Area %
6.373	0.16	1344.18	132.31	78.51
7.053	0.17	367.88	34.09	21.49
	Totals	1712.06		

ee after recrystallization

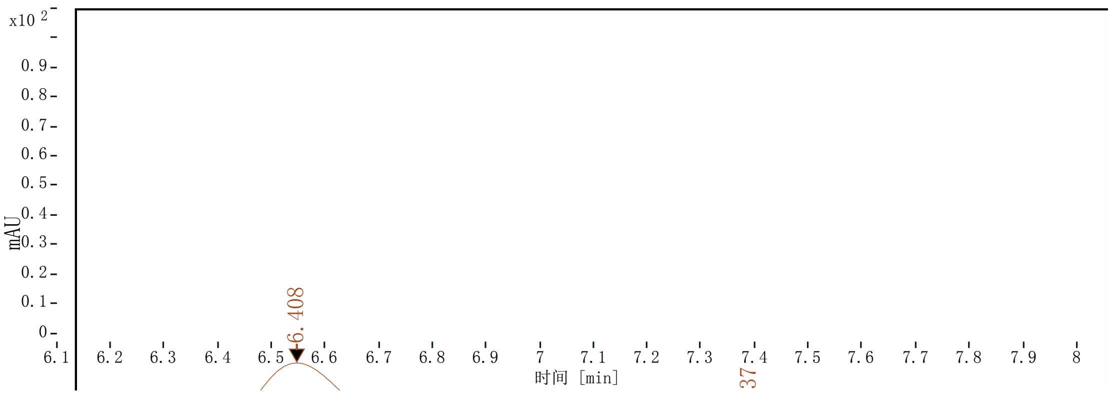

Ret Time[min]	Width [min]	Area	Height	Area %
6.408	0.15	196.47	20.76	97.42
7.037	0.13	5.20	0.52	2.58
	Totals	201.67		

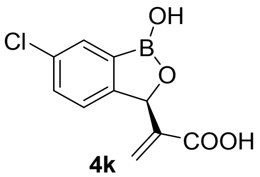

4k

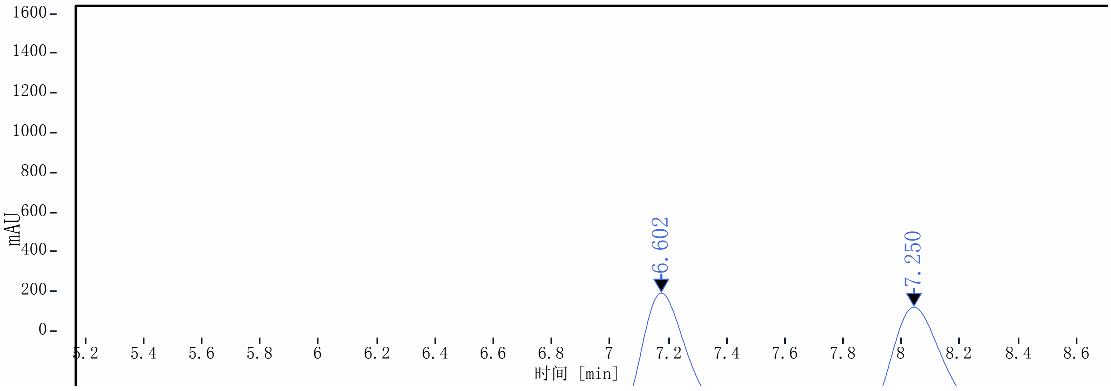

Ret Time[min]	Width [min]	Area	Height	Area %
6.602	0.71	5017.34	527.26	49.43
7.250	1.00	5132.07	469.39	50.57
	Totals	10149.41		

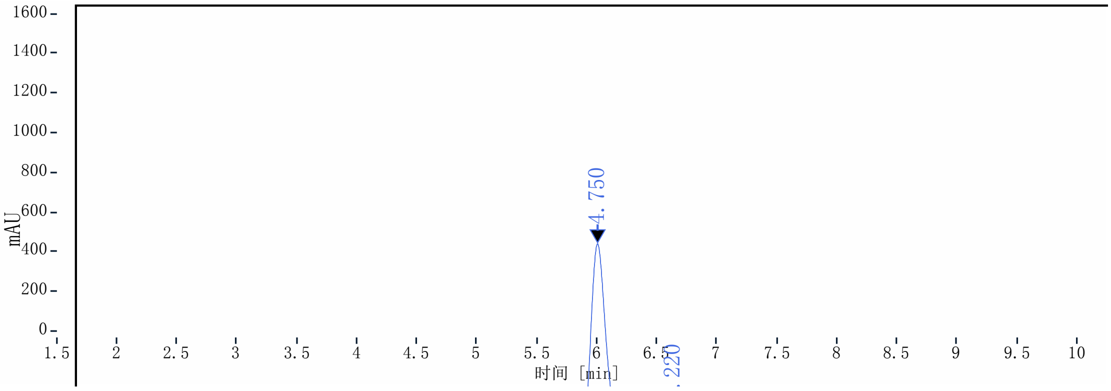

Ret Time[min]	Width [min]	Area	Height	Area %
4.750	0.65	5267.95	750.15	88.91
5.220	0.12	657.42	84.39	11.09
	Totals	5925.37		

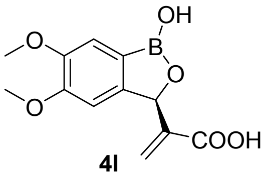

4l

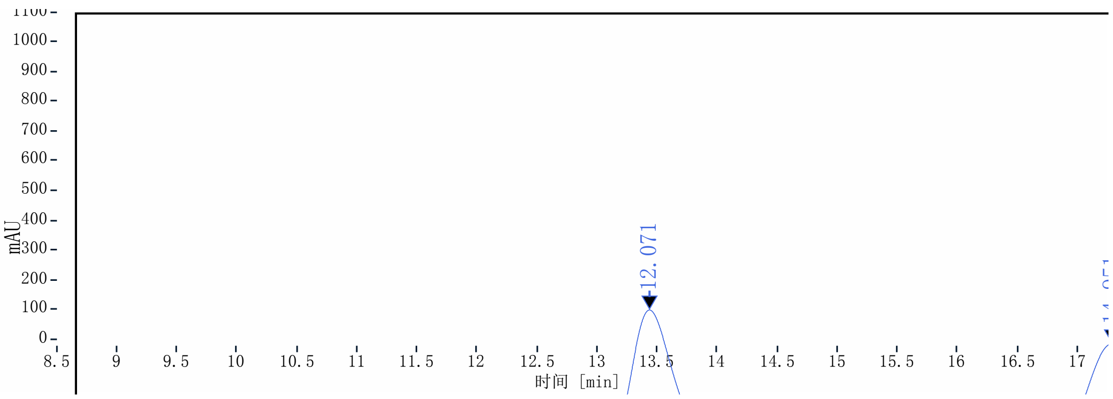

Ret Time[min]	Width [min]	Area	Height	Area %
12.071	1.31	6715.90	356.88	50.26
14.951	1.62	6645.44	270.79	49.74
	Totals	13361.33		

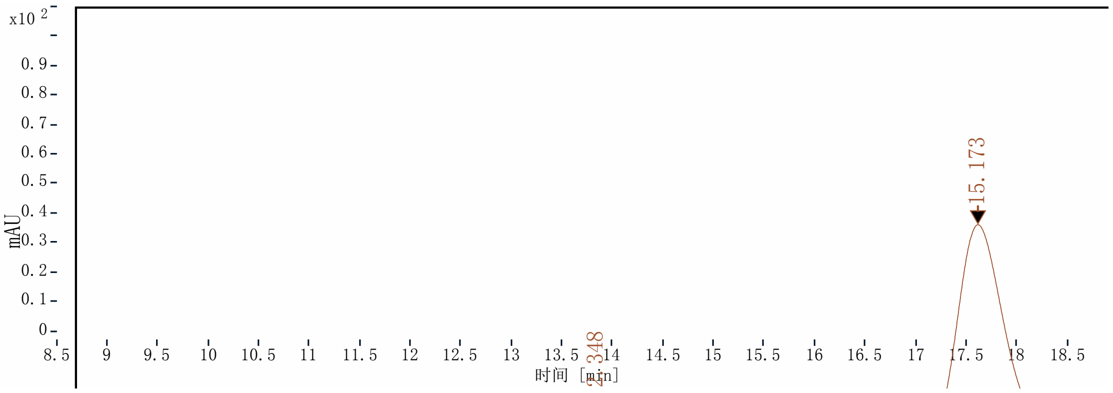

Ret Time[min]	Width [min]	Area	Height	Area %
12.348	0.31	141.51	6.66	9.22
15.173	0.38	1393.76	55.45	90.78
	Totals	1535.27		

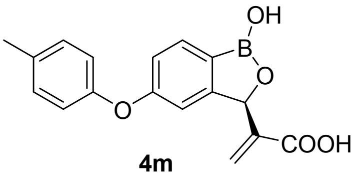

4m

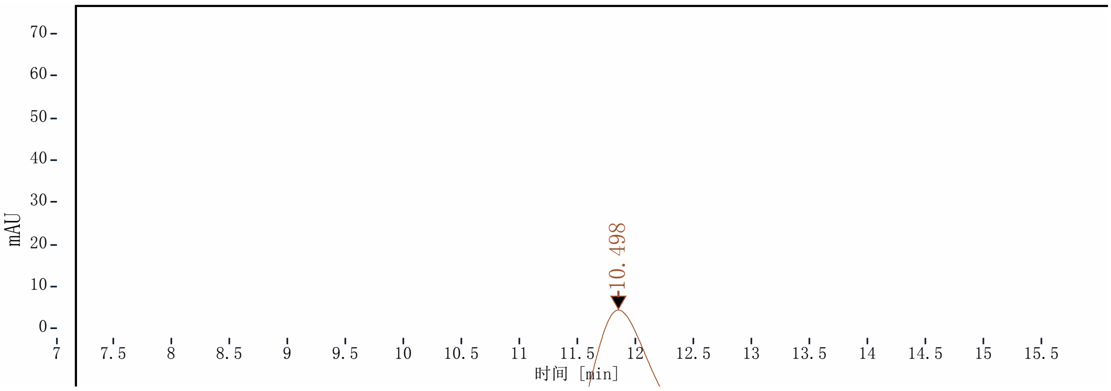

Ret Time[min]	Width [min]	Area	Height	Area %
10.498	0.41	646.64	23.44	50.66
14.160	0.45	629.79	21.08	49.34
	Totals	1276.43		

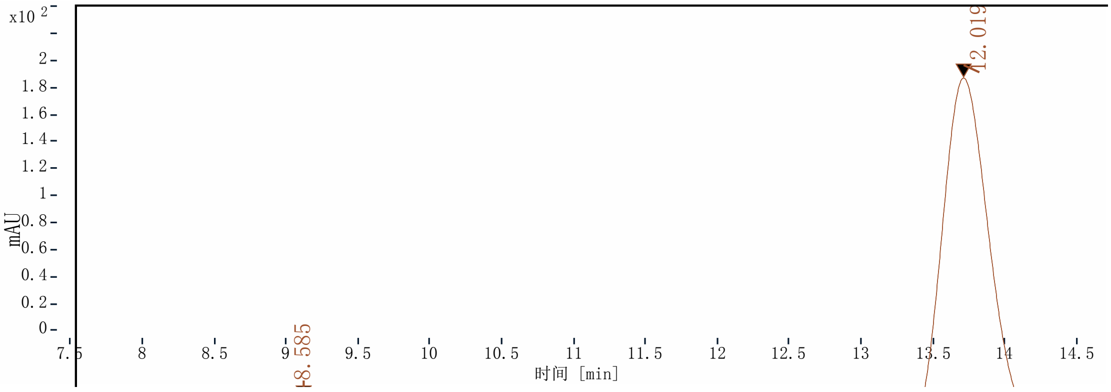

Ret Time[min]	Width [min]	Area	Height	Area %
8.585	0.19	293.61	23.98	7.42
12.019	1.91	3665.35	201.99	92.58
	Totals	3958.97		

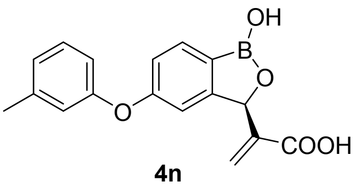

4n

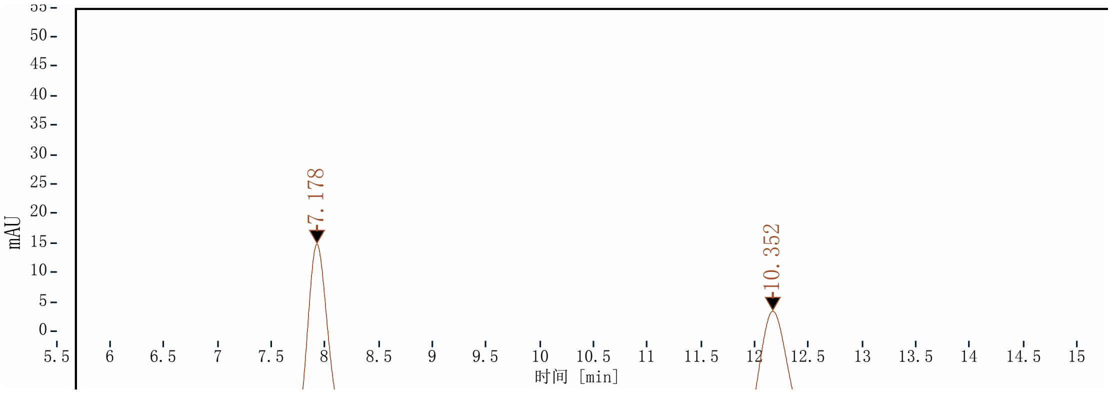

Ret Time[min]	Width [min]	Area	Height	Area %
7.178	0.16	263.85	25.26	50.15
10.352	0.23	262.29	17.40	49.85
	Totals	526.13		

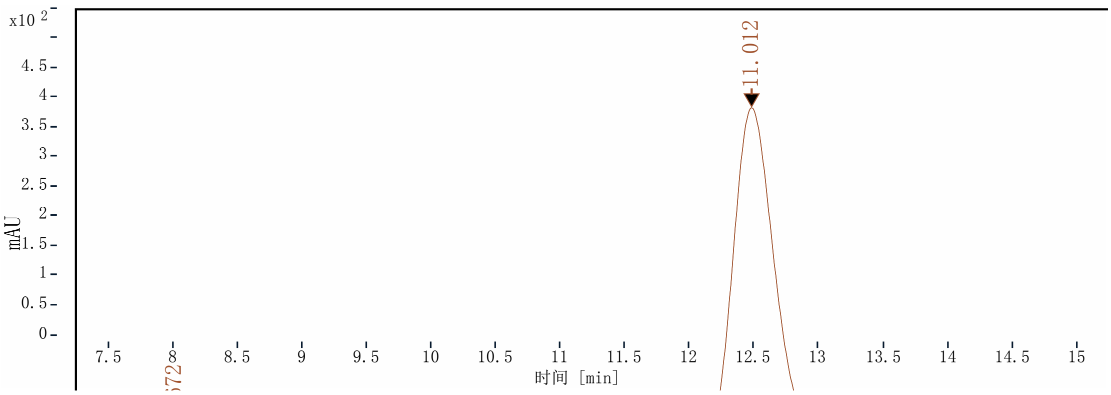

Ret Time[min]	Width [min]	Area	Height	Area %
7.672	0.17	100.52	9.42	1.38
11.012	1.08	7205.84	426.29	98.62
	Totals	7306.36		

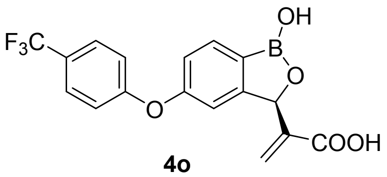

4o

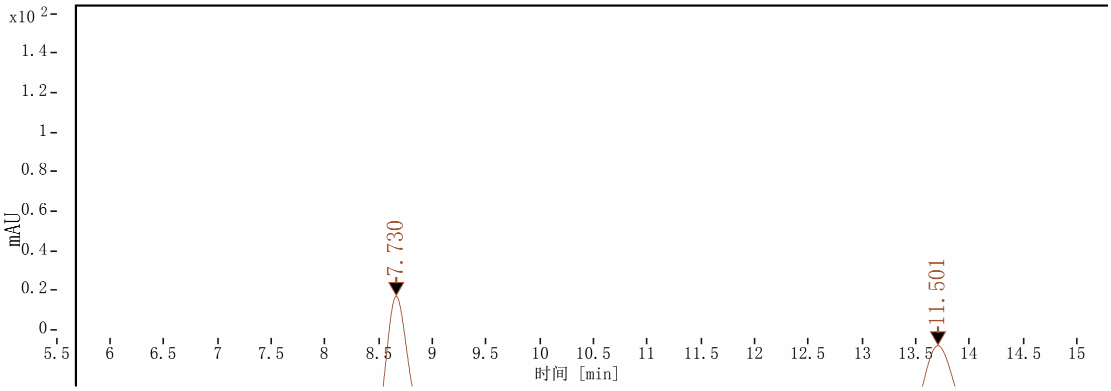

Ret Time[min]	Width [min]	Area	Height	Area %
7.730	0.18	666.58	55.87	51.12
11.501	0.26	637.48	37.19	48.88
	Totals	1304.05		

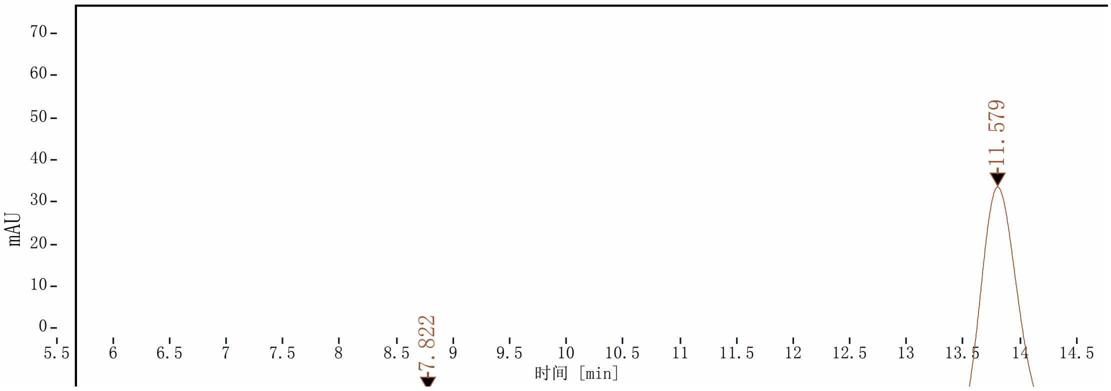

Ret Time[min]	Width [min]	Area	Height	Area %
7.822	0.18	100.48	8.59	11.00
11.579	0.27	812.80	44.73	89.00
	Totals	913.28		

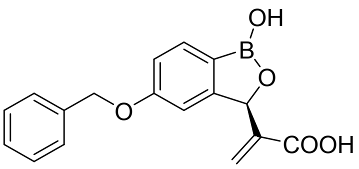

4p

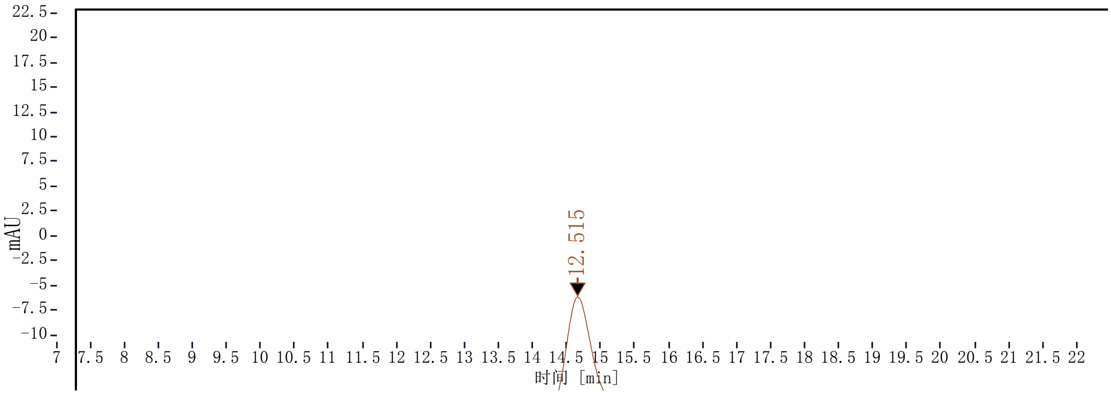

Ret Time[min]	Width [min]	Area	Height	Area %
12.515	0.29	178.68	8.70	50.78
18.974	0.35	173.18	6.02	49.22
	Totals	351.86		

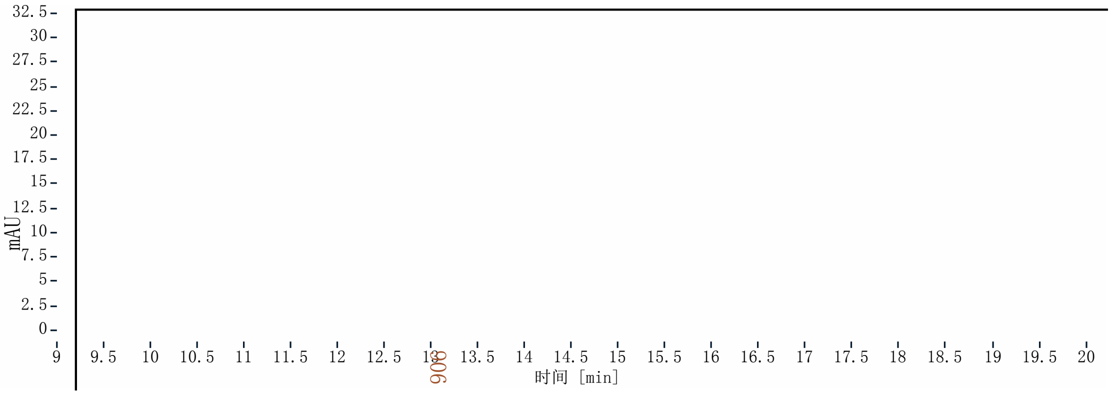

Ret Time[min]	Width [min]	Area	Height	Area %
11.906	0.25	26.02	1.27	6.30
17.899	0.42	386.71	11.75	93.70
	Totals	412.72		

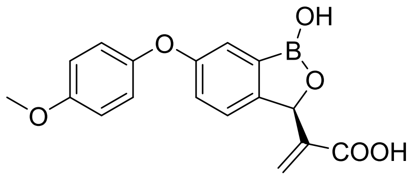

(*R*)-**4q**

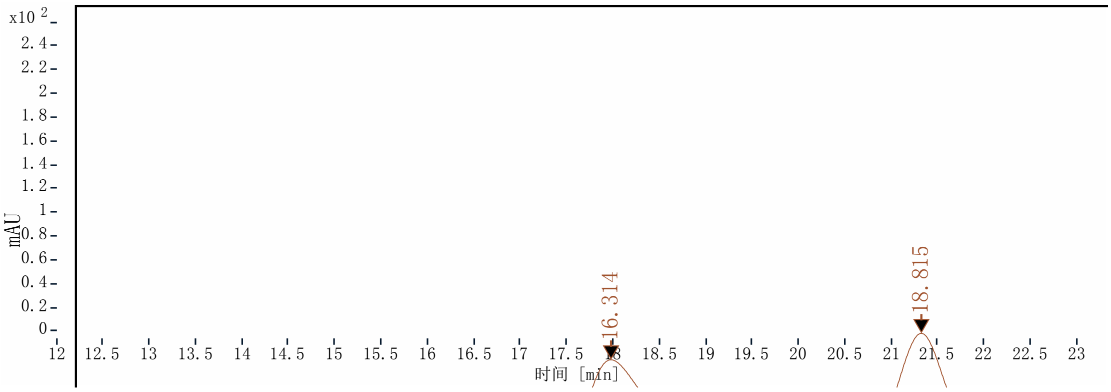

Ret Time[min]	Width [min]	Area	Height	Area %
16.314	0.49	1830.90	53.00	49.87
18.815	0.40	1840.25	69.19	50.13
	Totals	3671.15		

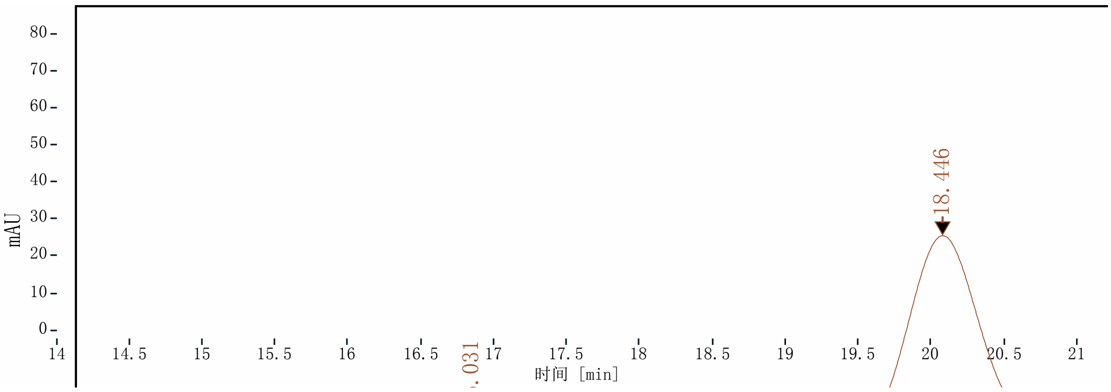

Ret Time[min]	Width [min]	Area	Height	Area %
16.031	0.30	75.35	2.96	6.17
18.446	0.42	1146.86	41.67	93.83
	Totals	1222.22		

ee after recrystallization

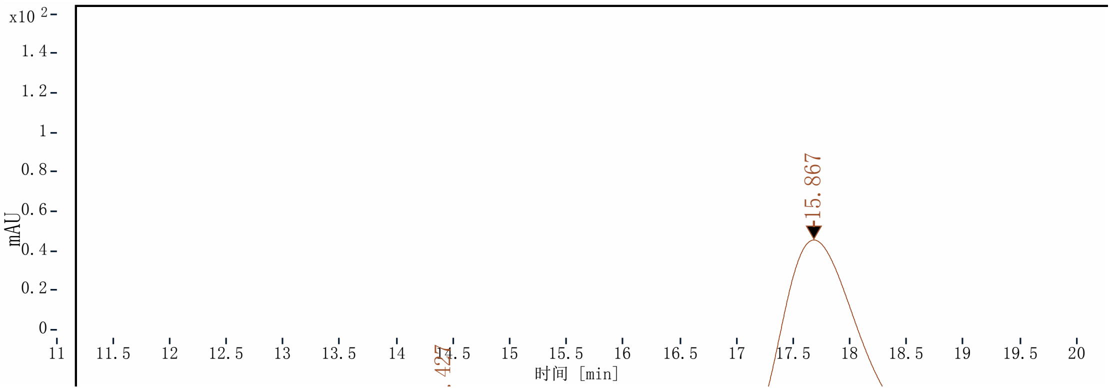

Ret Time[min]	Width [min]	Area	Height	Area %
13.427	0.29	94.38	4.60	3.30
15.867	0.55	2768.36	76.89	96.70
	Totals	2862.74		

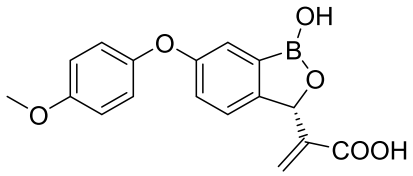

(*S*)-**4q**

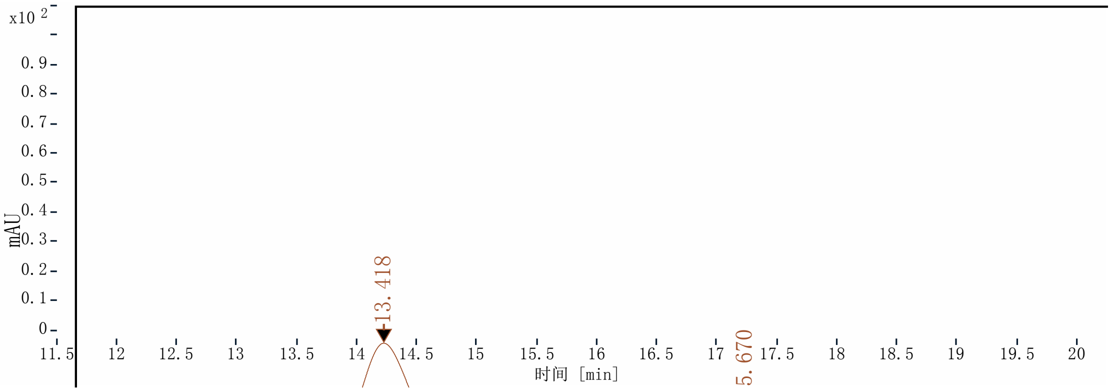

Ret Time[min]	Width [min]	Area	Height	Area %
13.418	0.32	521.22	25.26	75.01
15.670	0.36	173.62	6.62	24.99
	Totals	694.84		

ee after recrystallization

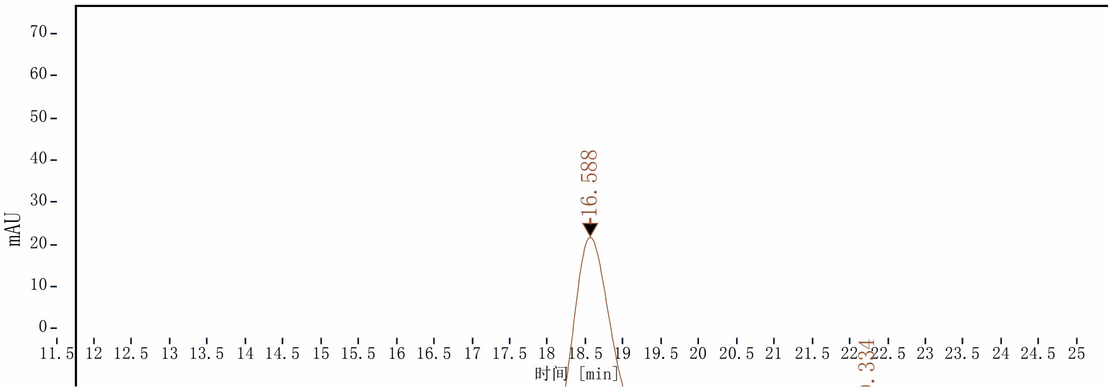

Ret Time[min]	Width [min]	Area	Height	Area %
16.588	0.41	954.22	35.74	94.95
19.334	0.29	50.72	2.06	5.05
	Totals	1004.94		

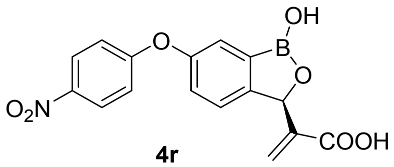

4r

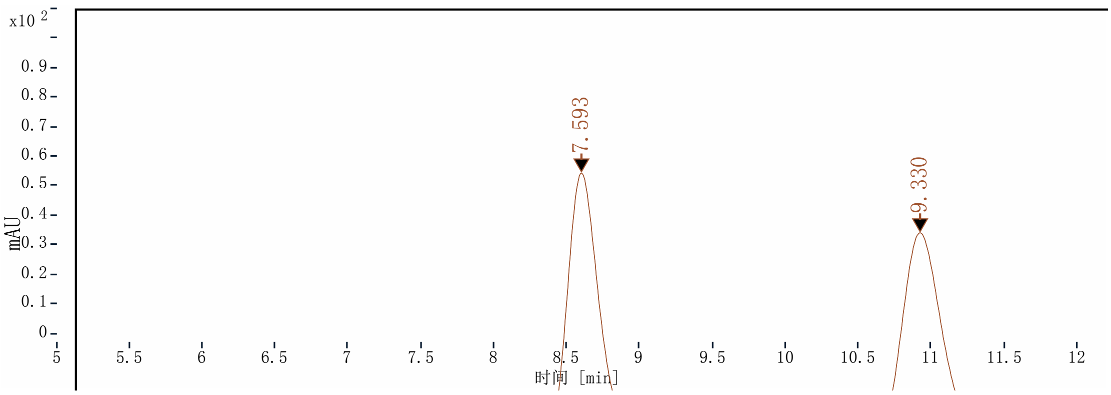

Ret Time[min]	Width [min]	Area	Height	Area %
7.593	0.18	794.80	68.70	49.53
9.330	0.23	809.91	53.92	50.47
	Totals	1604.71		

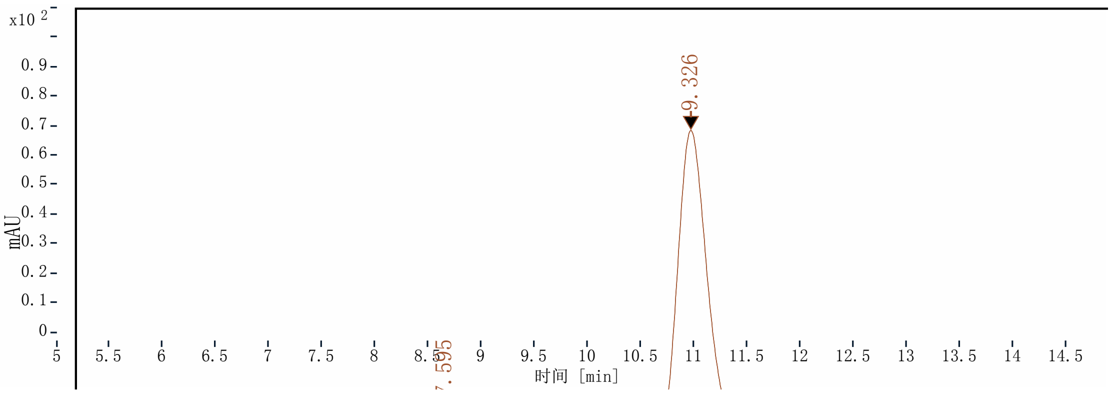

Ret Time[min]	Width [min]	Area	Height	Area %
7.595	0.17	87.86	7.73	6.78
9.326	0.23	1207.10	79.77	93.22
	Totals	1294.96		

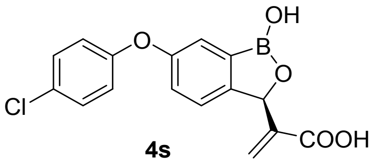

4s

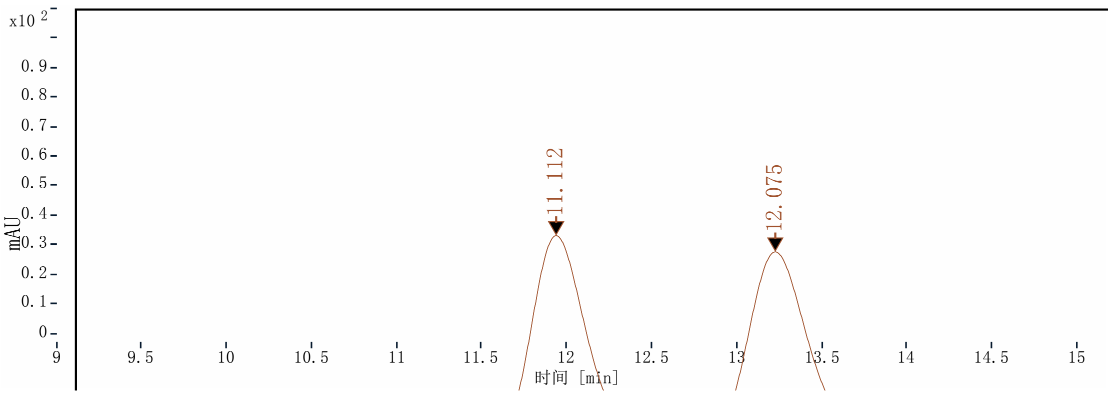

Ret Time[min]	Width [min]	Area	Height	Area %
11.112	0.27	928.64	53.07	49.55
12.075	0.29	945.59	48.90	50.45
	Totals	1874.23		

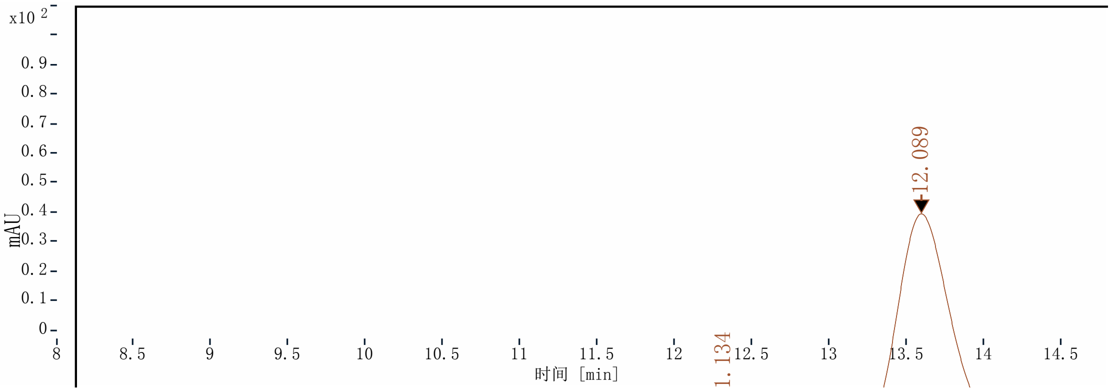

Ret Time[min]	Width [min]	Area	Height	Area %
11.134	0.21	92.33	5.75	7.78
12.089	1.49	1093.80	57.62	92.22
	Totals	1186.13		

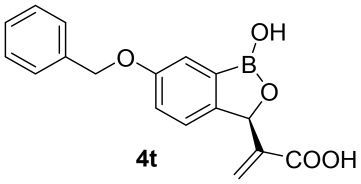

4t

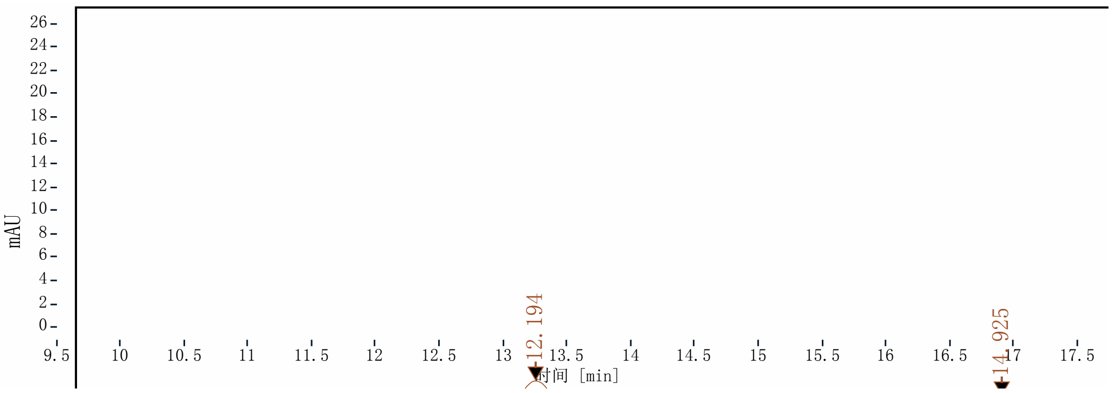

Ret Time[min]	Width [min]	Area	Height	Area %
12.194	0.29	71.96	3.79	50.51
14.925	0.36	70.51	2.97	49.49
	Totals	142.47		

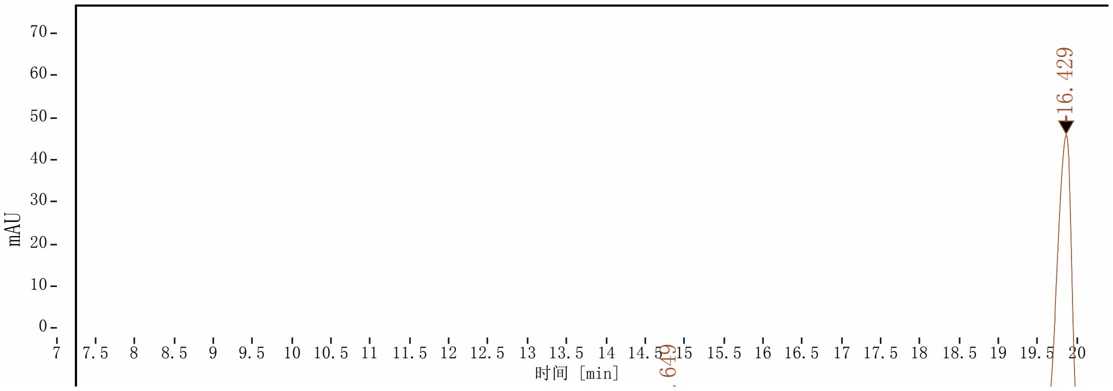

Ret Time[min]	Width [min]	Area	Height	Area %
12.649	0.45	58.10	1.54	10.03
16.429	0.15	521.02	53.93	89.97
	Totals	579.12		

X-ray Crystallographic Data for 4a.

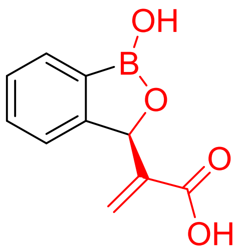

4a

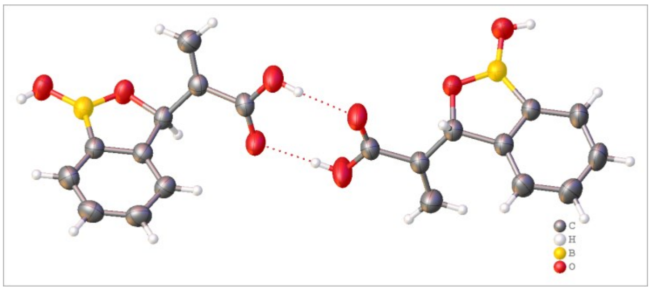

CDCC 2050141 contains supplementary crystallographic data for compound **4a**

Table 1 Crystal data and structure refinement for cu_CFE_CXP_P2_1201_0m.

Identification code	cu_CFE_CXP_P2_1201_0m
Empirical formula	$C_{10}H_9BO_4$
Formula weight	203.98
Temperature/K	295.0
Crystal system	orthorhombic
Space group	$P2_12_12_1$
a/Å	8.1938(4)
b/Å	8.3710(4)
c/Å	28.4985(14)
α/°	90
β/°	90
γ/°	90
Volume/Å^3	1954.72(16)
Z	8
ρ_{calc}g/cm^3	1.386
μ/mm^{-1}	0.885
F(000)	848.0
Crystal size/mm^3	0.3 × 0.2 × 0.2
Radiation	CuKα (λ = 1.54178)
2Θ range for data collection/°	6.202 to 136.636
Index ranges	-9 ≤ h ≤ 9, -9 ≤ k ≤ 10, -33 ≤ l ≤ 34
Reflections collected	19121
Independent reflections	3566 [R_{int} = 0.0275, R_{sigma} = 0.0176]
Data/restraints/parameters	3566/0/275
Goodness-of-fit on F^2	1.063
Final R indexes [I>=2σ (I)]	R_1 = 0.0290, wR_2 = 0.0753
Final R indexes [all data]	R_1 = 0.0313, wR_2 = 0.0770
Largest diff. peak/hole / e Å^{-3}	0.12/-0.14
Flack parameter	0.00(4)

Supplementary Figures

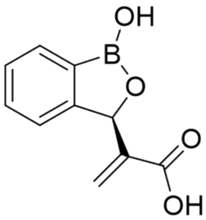

(*R*)-**4a**

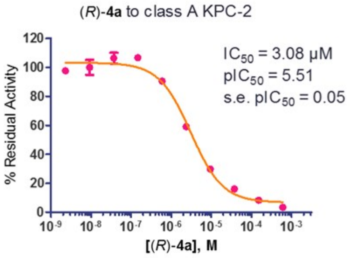

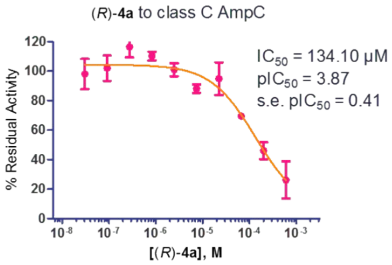

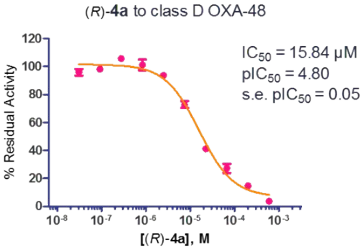

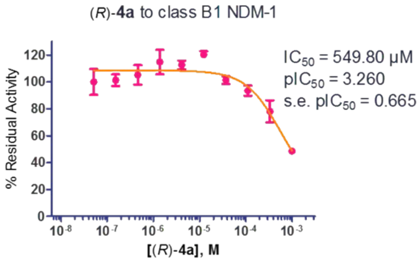

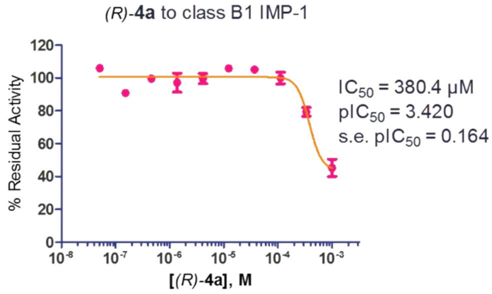

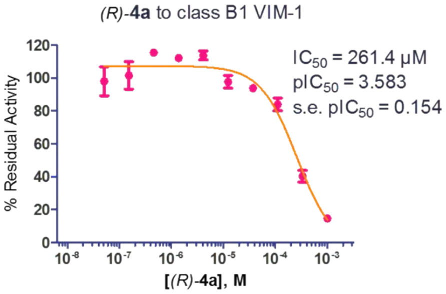

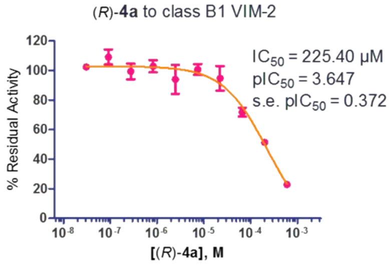

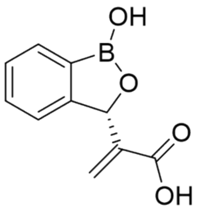

(S)-**4a**

(S)-**4a** to class A KPC

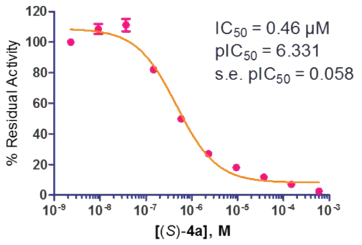

(S)-**4a** to class C Ampc

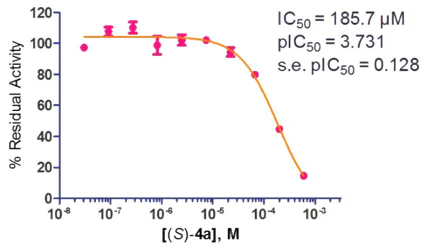

(S)-**4a** to class D OXA-48

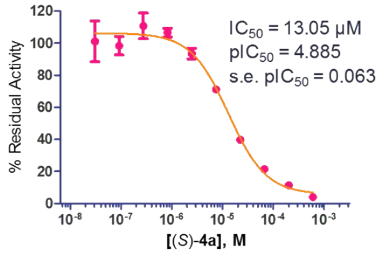

(S)-**4a** to class B1 NDM-1

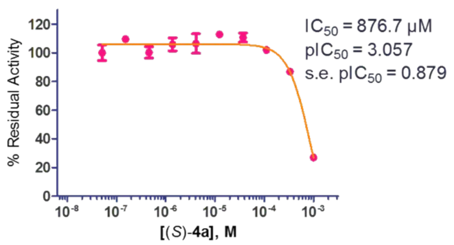

(S)-**4a** to class B1 IMP-1

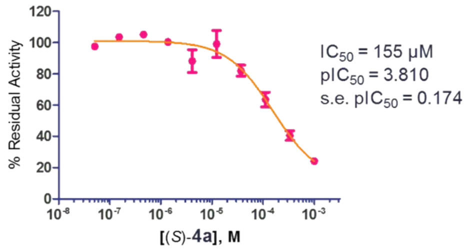

(S)-**4a** to class B1 VIM-1

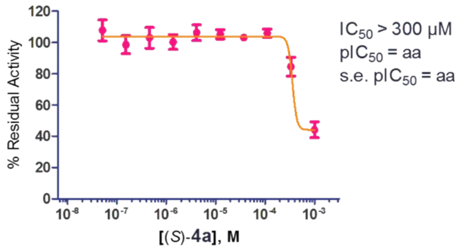

(S)-**4a** to class B1 VIM-2

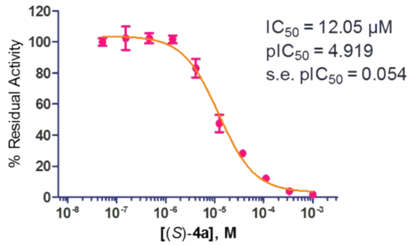

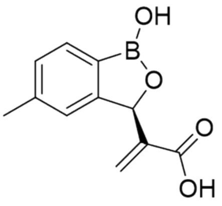

(*R*)-**4b**

(*R*)-**4b** to class A KPC-2

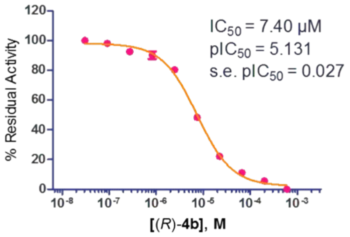

(*R*)-**4b** to class C AmpC

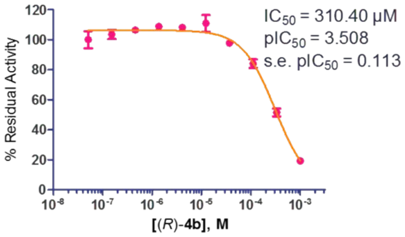

(*R*)-**4b** to class D OXA-48

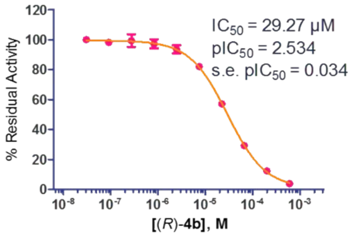

(*R*)-**4b** to class B1 NDM-1

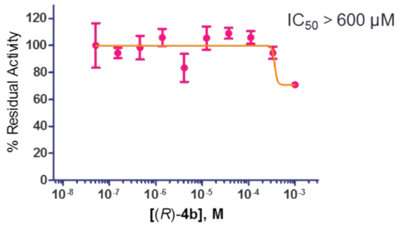

(*R*)-**4b** to class B1 IMP-1

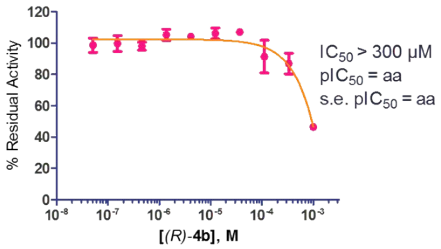

(*R*)-**4b** to class B1 VIM-1

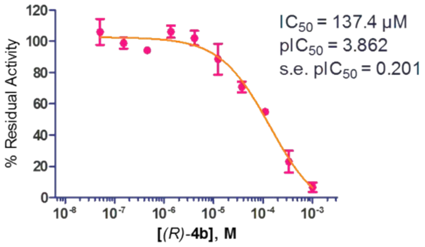

(*R*)-**4b** to class B1 VIM-2

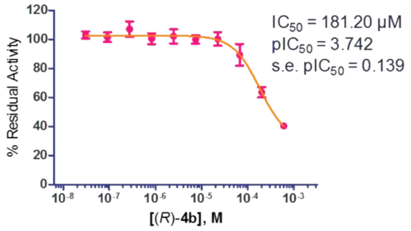

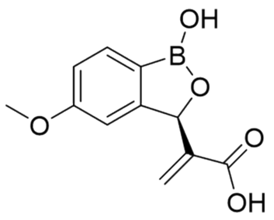

(*R*)-**4c**

(*R*)-**4c** to class A KPC-2

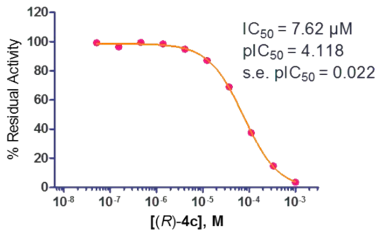

(*R*)-**4c** to class C AmpC

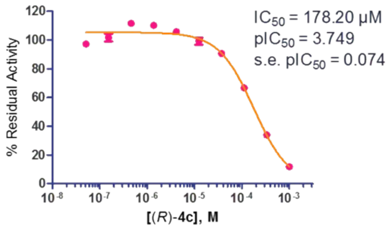

(*R*)-**4c** to class D OXA-48

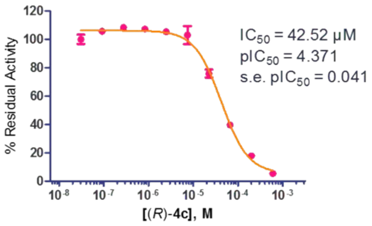

(*R*)-**4c** to class B1 NDM-1

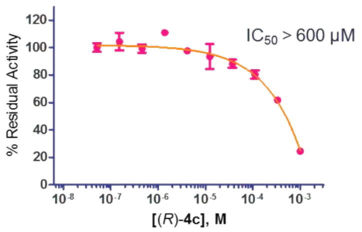

(*R*)-**4c** to class B1 IMP-1

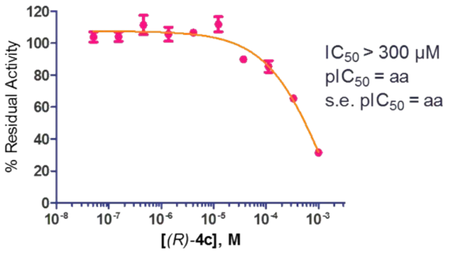

(*R*)-**4c** to class B1 VIM-1

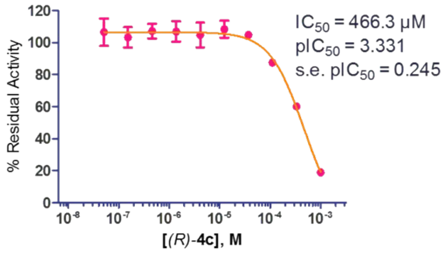

(*R*)-**4c** to class B1 VIM-2

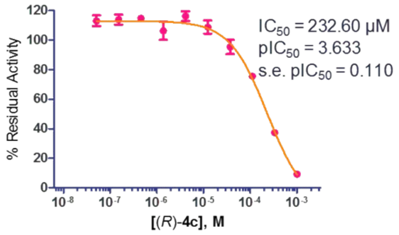

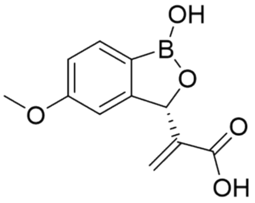

(*S*)-**4c**

(*S*)-**4c** to class A KPC-2

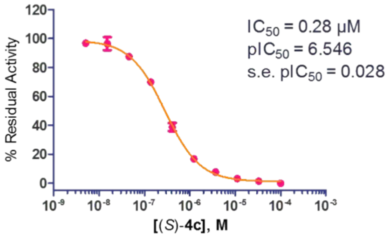

(*S*)-**4c** to class C Ampc

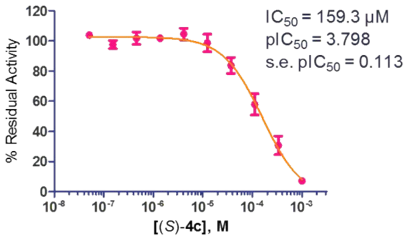

(*S*)-**4c** to class D OXA-48

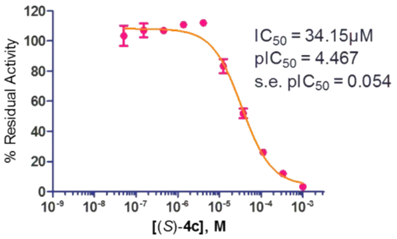

(*S*)-**4c** to class B1 NDM-1

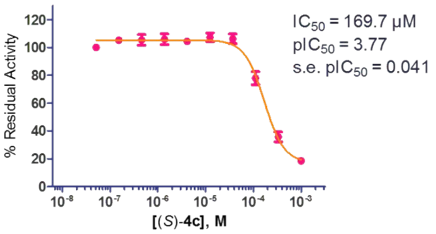

(*S*)-**4c** to class B1 IMP-1

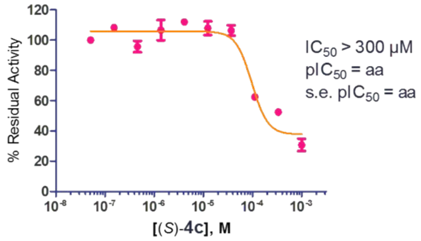

(*S*)-**4c** to class B1 VIM-1

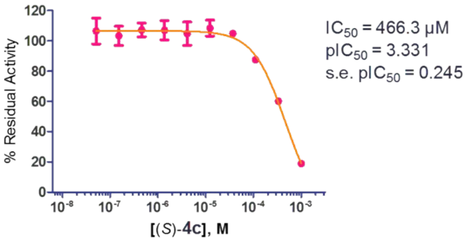

(*S*)-**4c** to class B1 VIM-2

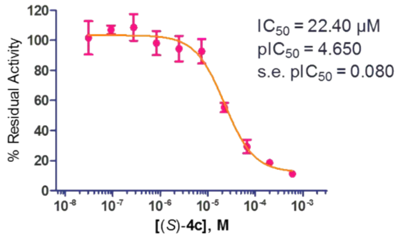

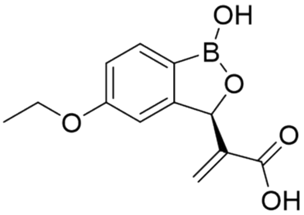

(*R*)-**4d**

(*R*)-**4d** to class A KPC-2

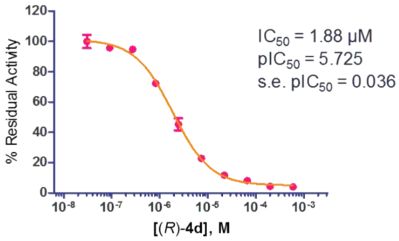

(*R*)-**4d** to class C AmpC

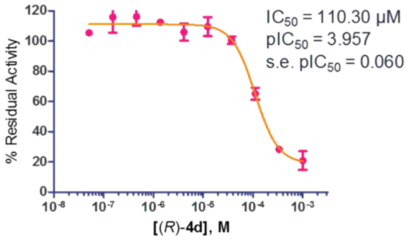

(*R*)-**4d** to class D OXA-48

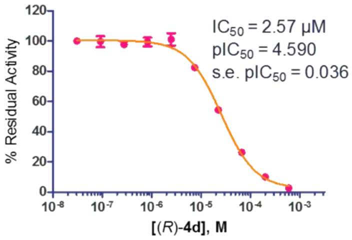

(*R*)-**4d** to class B1 NDM-1

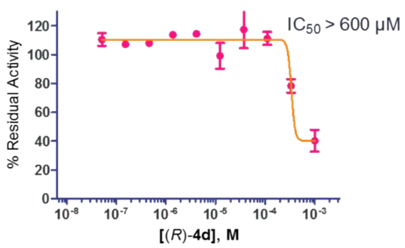

(*R*)-**4d** to class B1 IMP-1

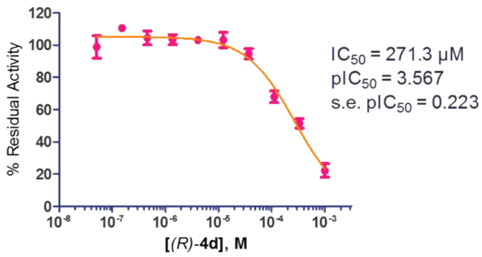

(*R*)-**4d** to class B1 VIM-1

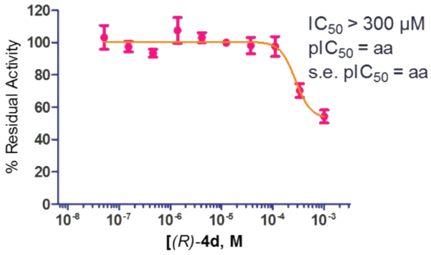

(*R*)-**4d** to class B1 VIM-2

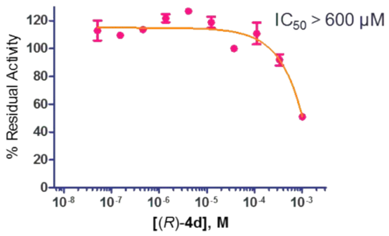

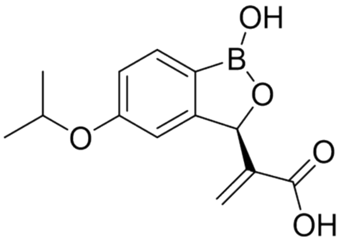

(*R*)-**4e**

(*R*)-**4e** to class A KPC-2

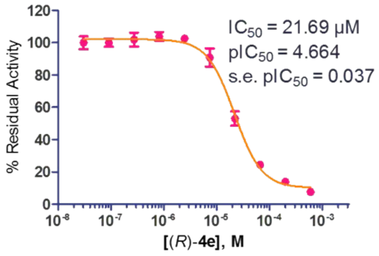

(*R*)-**4e** to class C AmpC

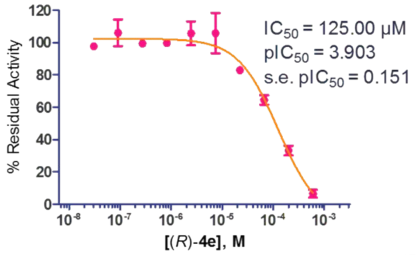

(*R*)-**4e** to class D OXA-48

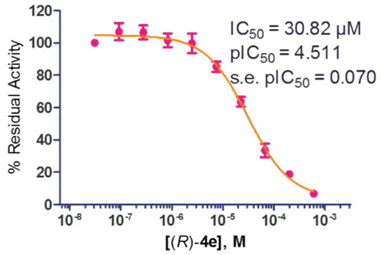

(*R*)-**4e** to class B1 NDM-1

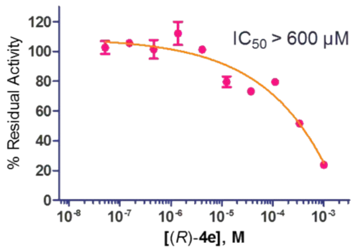

(*R*)-**4e** to class B1 IMP-1

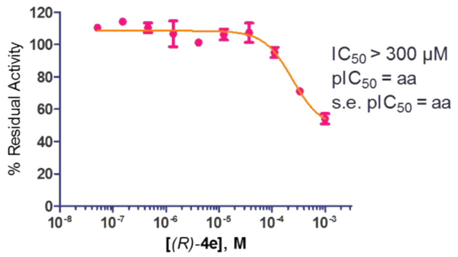

(*R*)-**4e** to class B1 VIM-1

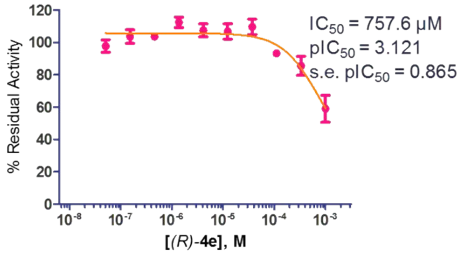

(*R*)-**4e** to class B1 VIM-2

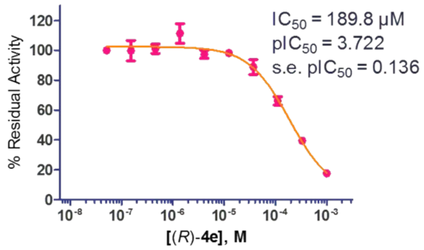

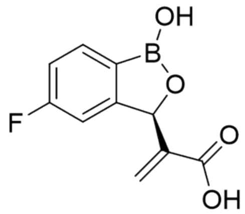

(*R*)-**4f**

(*R*)-**4f** to class A KPC-2

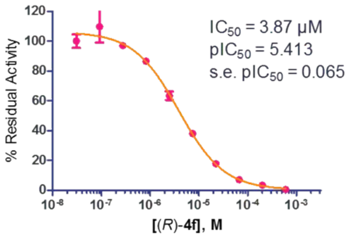

(*R*)-**4f** to class C AmpC

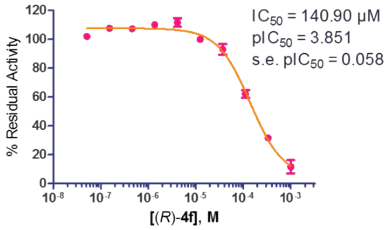

(*R*)-**4f** to class D OXA-48

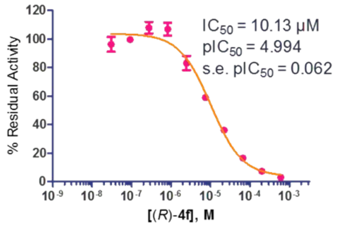

(*R*)-**4f** to class B1 NDM-1

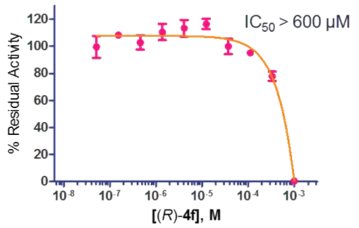

(*R*)-**4f** to class B1 IMP-1

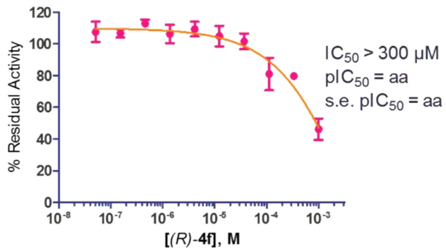

(*R*)-**4f** to class B1 VIM-1

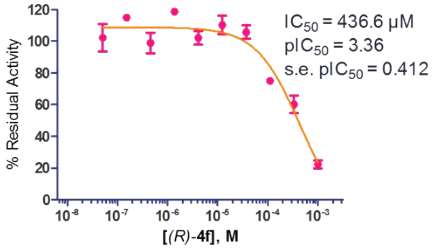

(*R*)-**4f** to class B1 VIM-2

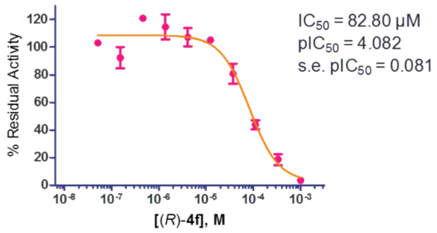

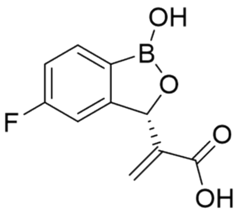

(*S*)-**4f**

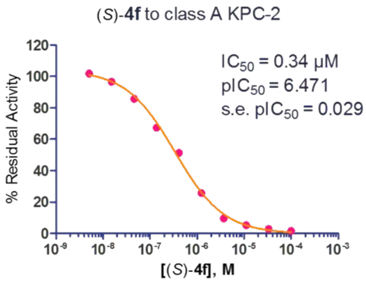
(S)-4f to class A KPC-2
IC50 = 0.34 μM
pIC50 = 6.471
s.e. pIC50 = 0.029
% Residual Activity
[(S)-4f], M

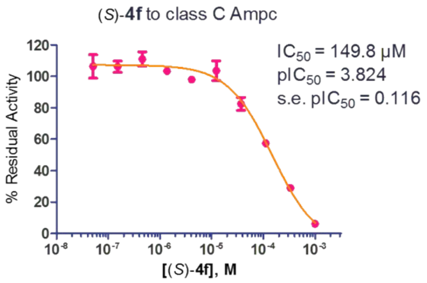
(S)-4f to class C Ampc
IC50 = 149.8 μM
pIC50 = 3.824
s.e. pIC50 = 0.116
% Residual Activity
[(S)-4f], M

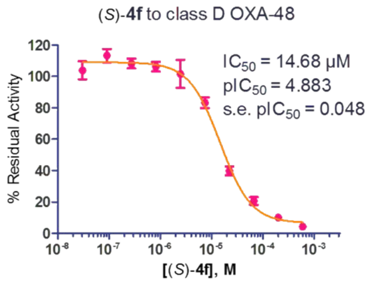
(S)-4f to class D OXA-48
IC50 = 14.68 μM
pIC50 = 4.883
s.e. pIC50 = 0.048
% Residual Activity
[(S)-4f], M

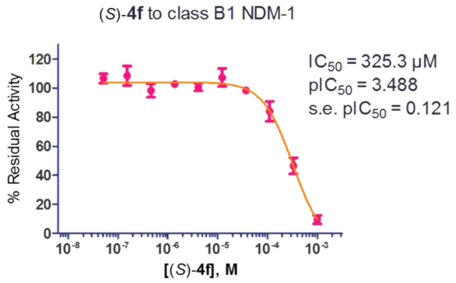
(S)-4f to class B1 NDM-1
IC50 = 325.3 μM
pIC50 = 3.488
s.e. pIC50 = 0.121
% Residual Activity
[(S)-4f], M

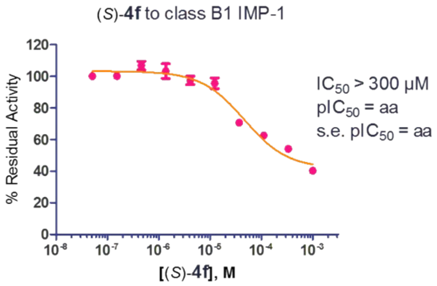
(S)-4f to class B1 IMP-1
IC50 > 300 μM
pIC50 = aa
s.e. pIC50 = aa
% Residual Activity
[(S)-4f], M

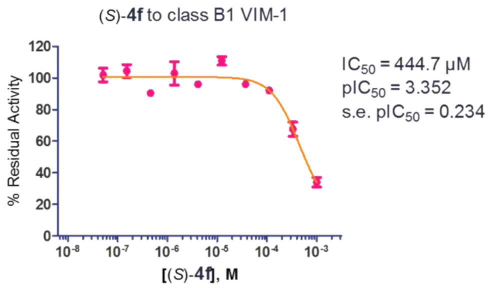
(S)-4f to class B1 VIM-1
IC50 = 444.7 μM
pIC50 = 3.352
s.e. pIC50 = 0.234
% Residual Activity
[(S)-4f], M

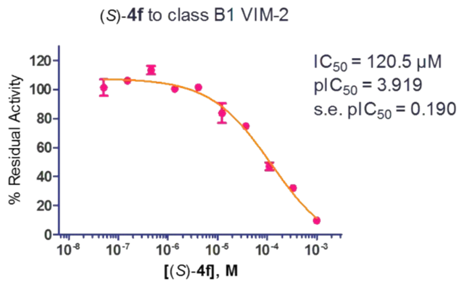
(S)-4f to class B1 VIM-2
IC50 = 120.5 μM
pIC50 = 3.919
s.e. pIC50 = 0.190
% Residual Activity
[(S)-4f], M

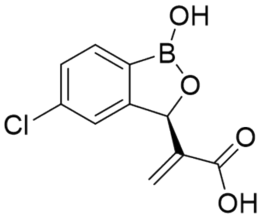

(*R*)-**4g**

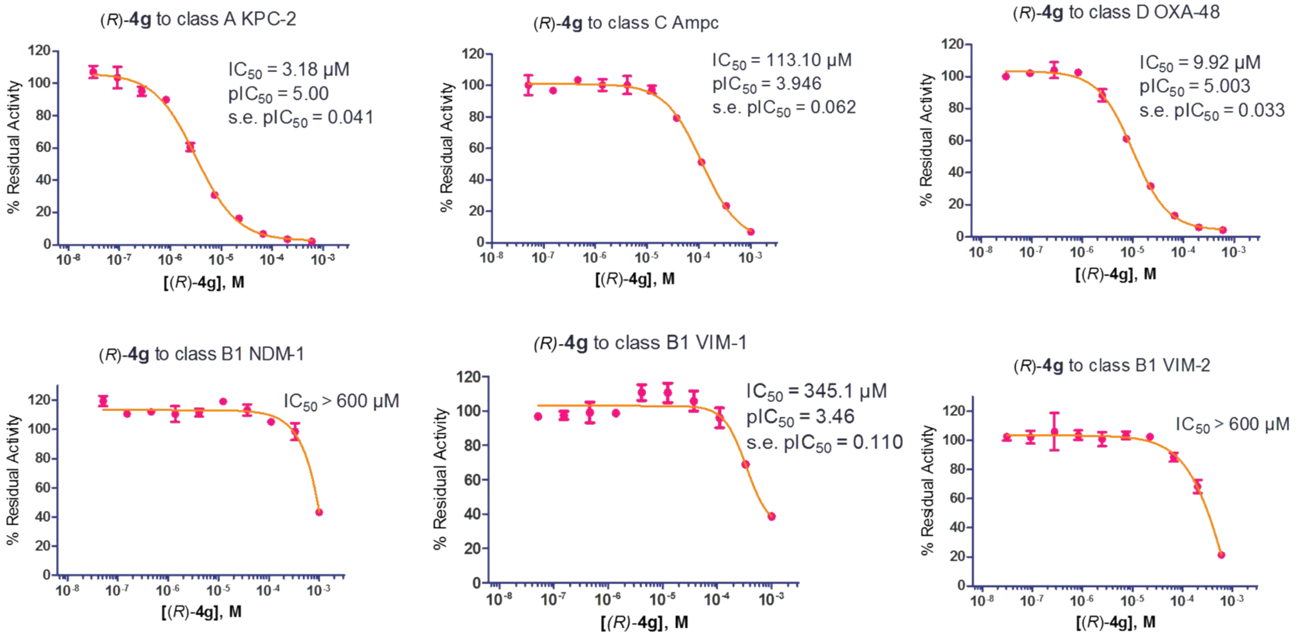
(R)-4g to class A KPC-2
IC50 = 3.18 µM
pIC50 = 5.00
s.e. pIC50 = 0.041
(R)-4g to class C Ampc
IC50 = 113.10 µM
pIC50 = 3.946
s.e. pIC50 = 0.062
(R)-4g to class D OXA-48
IC50 = 9.92 µM
pIC50 = 5.003
s.e. pIC50 = 0.033
(R)-4g to class B1 NDM-1
IC50 > 600 µM
(R)-4g to class B1 VIM-1
IC50 = 345.1 µM
pIC50 = 3.46
s.e. pIC50 = 0.110
(R)-4g to class B1 VIM-2
IC50 > 600 µM
% Residual Activity
[(R)-4g], M

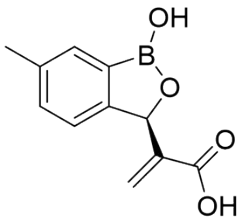

(*R*)-**4h**

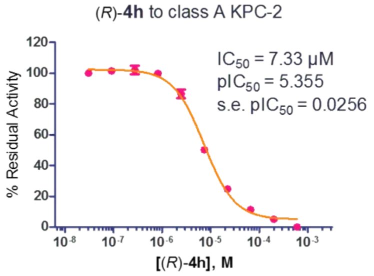
(R)-4h to class A KPC-2
IC50 = 7.33 μM
pIC50 = 5.355
s.e. pIC50 = 0.0256
% Residual Activity
[(R)-4h], M

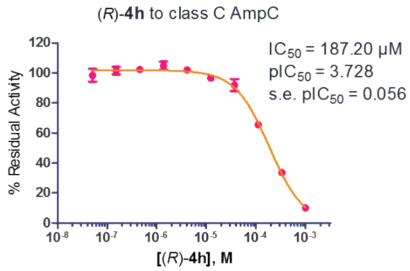
(R)-4h to class C AmpC
IC50 = 187.20 μM
pIC50 = 3.728
s.e. pIC50 = 0.056
% Residual Activity
[(R)-4h], M

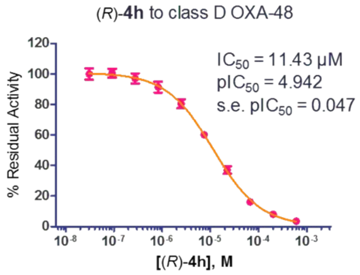
(R)-4h to class D OXA-48
IC50 = 11.43 μM
pIC50 = 4.942
s.e. pIC50 = 0.047
% Residual Activity
[(R)-4h], M

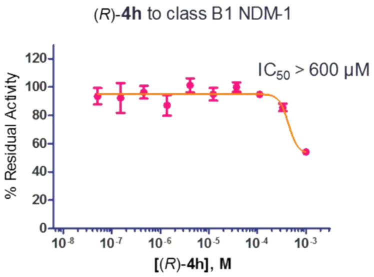
(R)-4h to class B1 NDM-1
IC50 > 600 μM
% Residual Activity
[(R)-4h], M

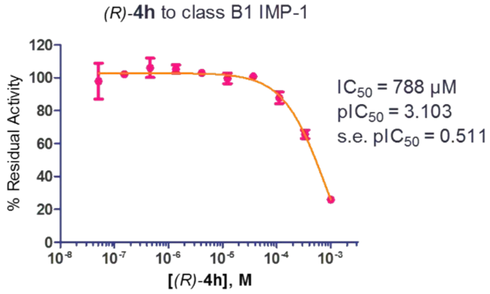
(R)-4h to class B1 IMP-1
IC50 = 788 μM
pIC50 = 3.103
s.e. pIC50 = 0.511
% Residual Activity
[(R)-4h], M

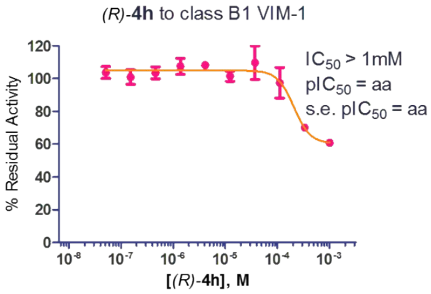
(R)-4h to class B1 VIM-1
IC50 > 1mM
pIC50 = aa
s.e. pIC50 = aa
% Residual Activity
[(R)-4h], M

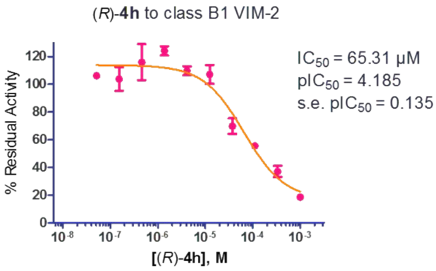
(R)-4h to class B1 VIM-2
IC50 = 65.31 μM
pIC50 = 4.185
s.e. pIC50 = 0.135
% Residual Activity
[(R)-4h], M

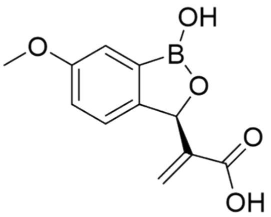

(*R*)-**4i**

(*R*)-**4i** to class A KPC-2

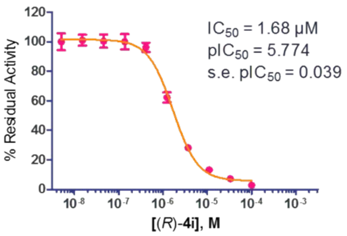

(*R*)-**4i** to class C AmpC

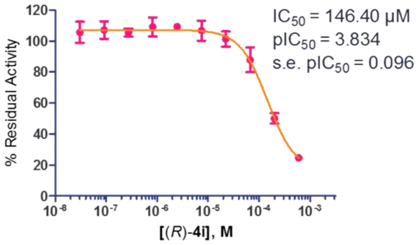

(*R*)-**4i** to class D OXA-48

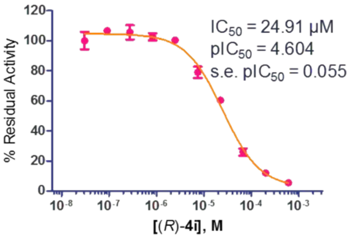

(*R*)-**4i** to class B1 NDM-1

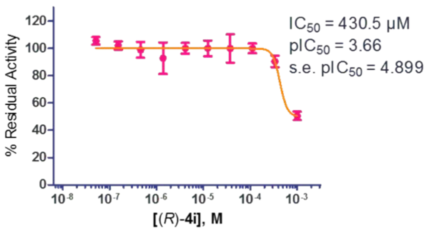

(*R*)-**4i** to class B1 IMP-1

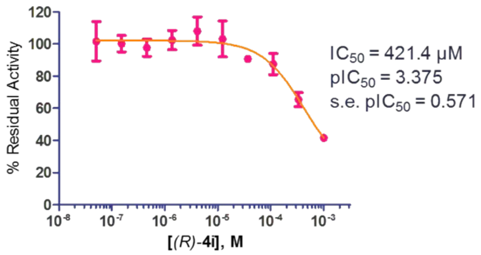

(*R*)-**4i** to class B1 VIM-1

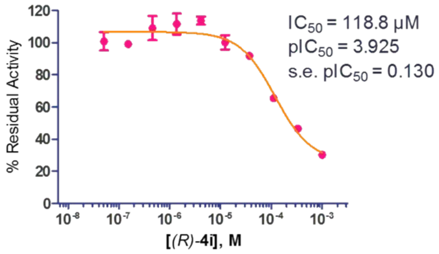

(*R*)-**4i** to class B1 VIM-2

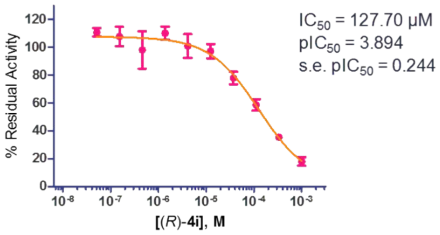

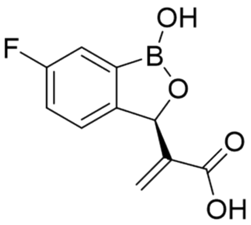

(*R*)-**4j**

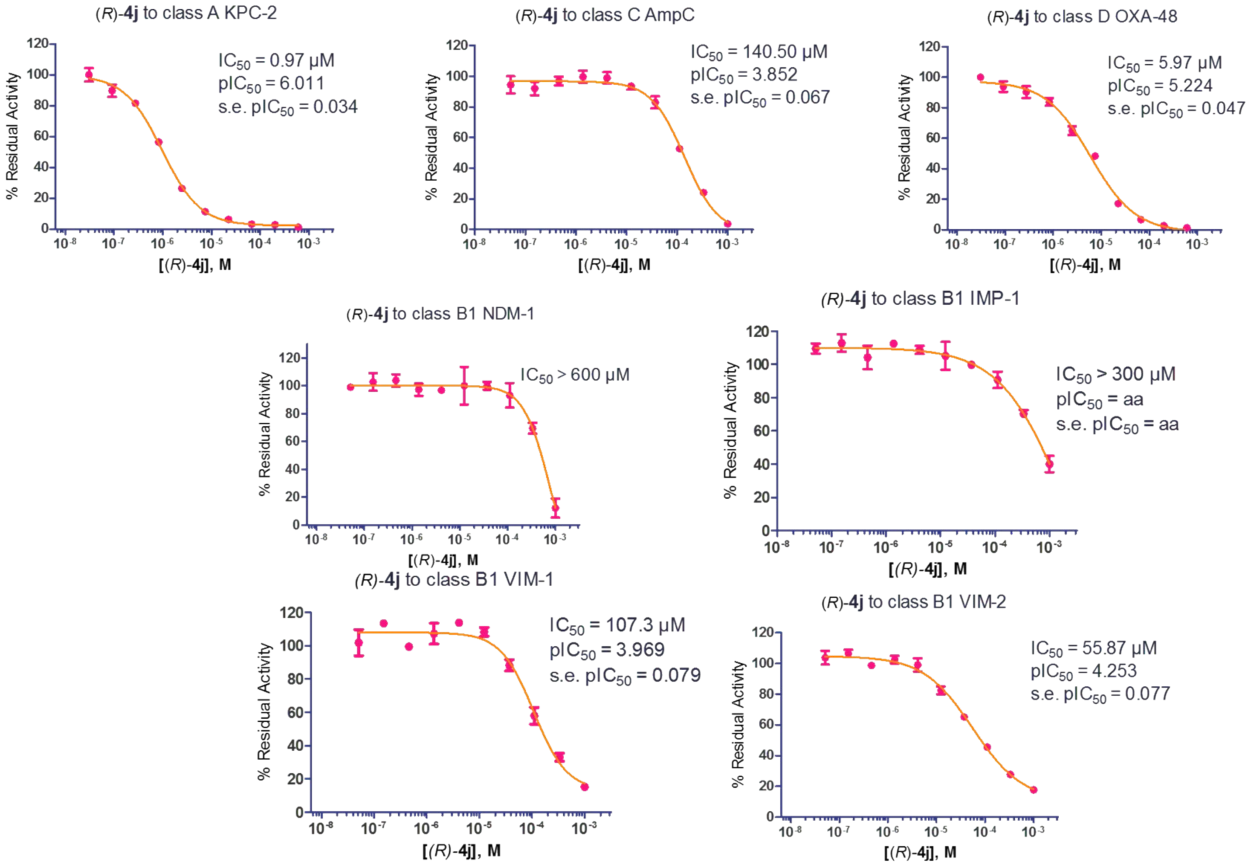
(R)-4j to class A KPC-2
IC50 = 0.97 μM
pIC50 = 6.011
s.e. pIC50 = 0.034
% Residual Activity
[(R)-4j], M
(R)-4j to class C AmpC
IC50 = 140.50 μM
pIC50 = 3.852
s.e. pIC50 = 0.067
% Residual Activity
[(R)-4j], M
(R)-4j to class D OXA-48
IC50 = 5.97 μM
pIC50 = 5.224
s.e. pIC50 = 0.047
% Residual Activity
[(R)-4j], M
(R)-4j to class B1 NDM-1
IC50 > 600 μM
% Residual Activity
[(R)-4j], M
(R)-4j to class B1 IMP-1
IC50 > 300 μM
pIC50 = aa
s.e. pIC50 = aa
% Residual Activity
[(R)-4j], M
(R)-4j to class B1 VIM-1
IC50 = 107.3 μM
pIC50 = 3.969
s.e. pIC50 = 0.079
% Residual Activity
[(R)-4j], M
(R)-4j to class B1 VIM-2
IC50 = 55.87 μM
pIC50 = 4.253
s.e. pIC50 = 0.077
% Residual Activity
[(R)-4j], M

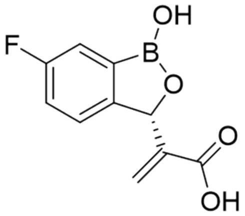

(*S*)-**4j**

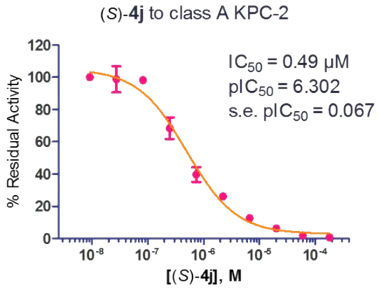
(S)-4j to class A KPC-2
IC50 = 0.49 μM
pIC50 = 6.302
s.e. pIC50 = 0.067
% Residual Activity
[(S)-4j], M

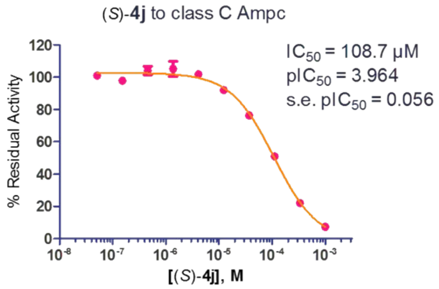
(S)-4j to class C Ampc
IC50 = 108.7 μM
pIC50 = 3.964
s.e. pIC50 = 0.056
% Residual Activity
[(S)-4j], M

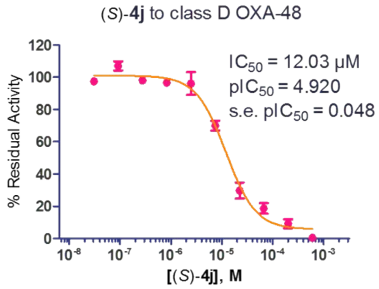
(S)-4j to class D OXA-48
IC50 = 12.03 μM
pIC50 = 4.920
s.e. pIC50 = 0.048
% Residual Activity
[(S)-4j], M

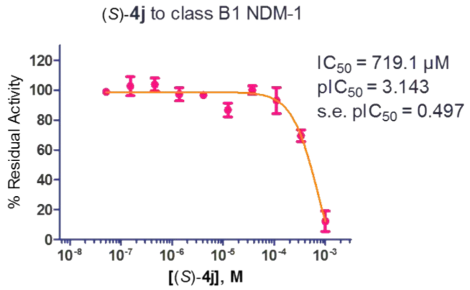
(S)-4j to class B1 NDM-1
IC50 = 719.1 μM
pIC50 = 3.143
s.e. pIC50 = 0.497
% Residual Activity
[(S)-4j], M

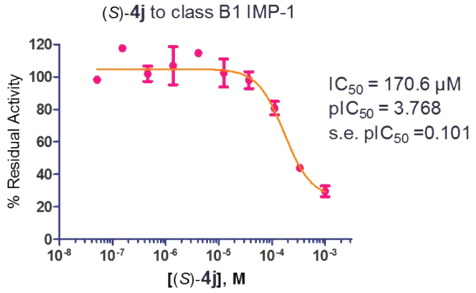
(S)-4j to class B1 IMP-1
IC50 = 170.6 μM
pIC50 = 3.768
s.e. pIC50 =0.101
% Residual Activity
[(S)-4j], M

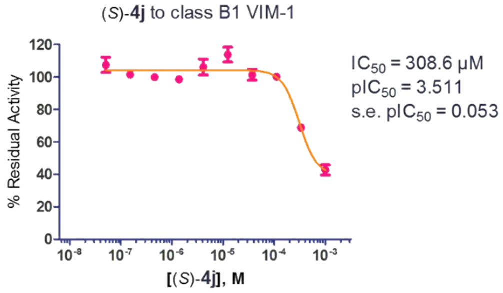
(S)-4j to class B1 VIM-1
IC50 = 308.6 μM
pIC50 = 3.511
s.e. pIC50 = 0.053
% Residual Activity
[(S)-4j], M

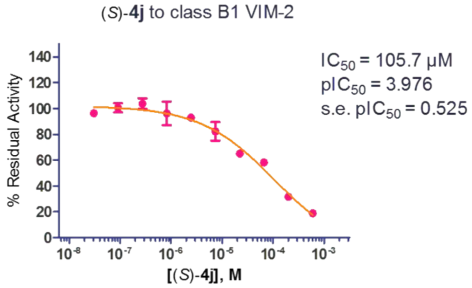
(S)-4j to class B1 VIM-2
IC50 = 105.7 μM
pIC50 = 3.976
s.e. pIC50 = 0.525
% Residual Activity
[(S)-4j], M

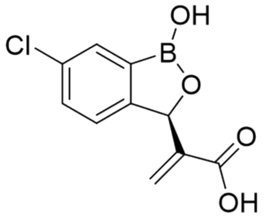

(*R*)-**4k**

(*R*)-**4k** to class A KPC-2

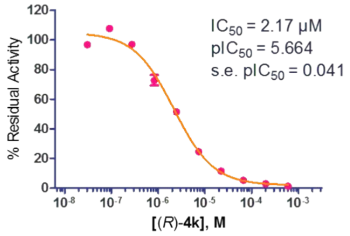

(*R*)-**4k** to class C AmpC

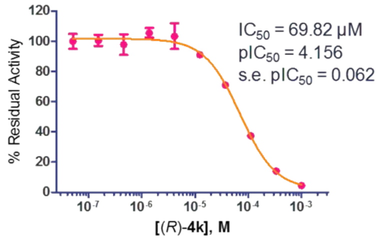

(*R*)-**4k** to class D OXA-48

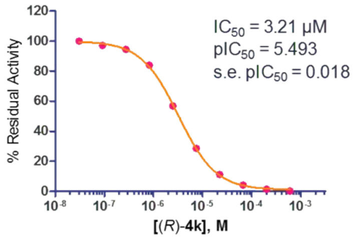

(*R*)-**4k** to class B1 NDM-1

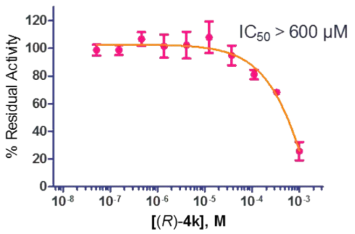

(*R*)-**4k** to class B1 IMP-1

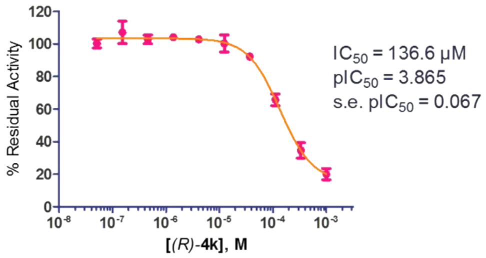

(*R*)-**4k** to class B1 VIM-1

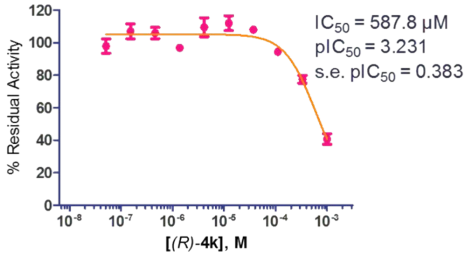

(*R*)-**4k** to class B1 VIM-2

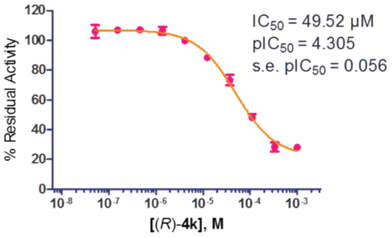

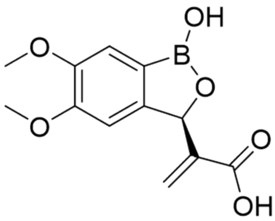

(*R*)-**4l**

(*R*)-**4l** to class A KPC-2

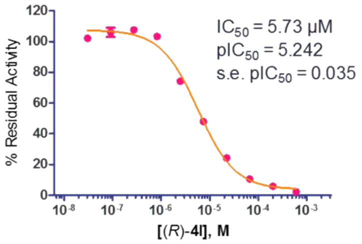

(*R*)-**4l** to class C AmpC

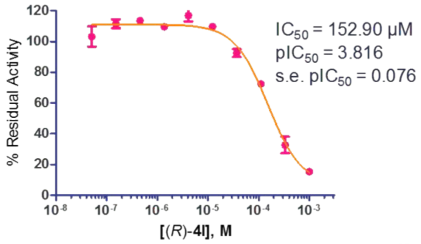

(*R*)-**4l** to class D OXA-48

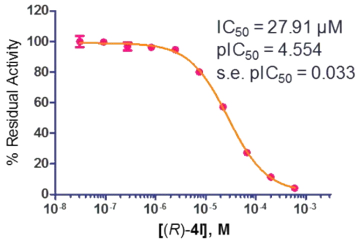

(*R*)-**4l** to class B1 NDM-1

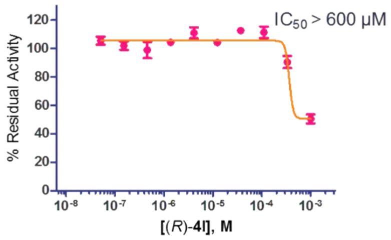

(*R*)-**4l** to class B1 IMP-1

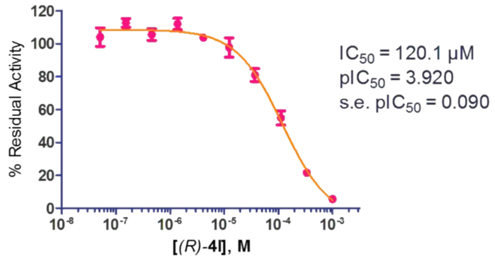

(*R*)-**4l** to class B1 VIM-1

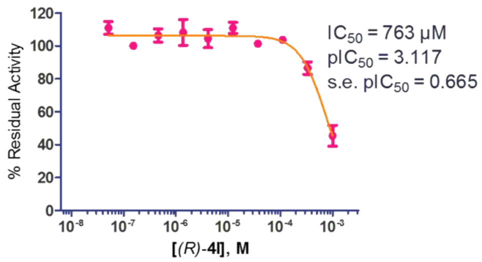

(*R*)-**4l** to class B1 VIM-2

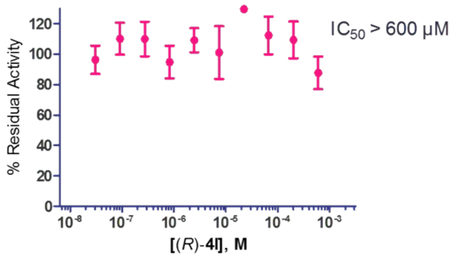

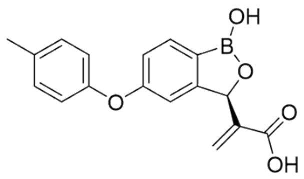

(*R*)-**4m**

(*R*)-**4m** to class A KPC-2

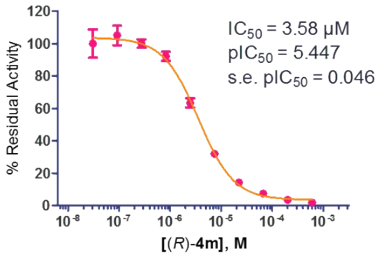

(*R*)-**4m** to class C AmpC

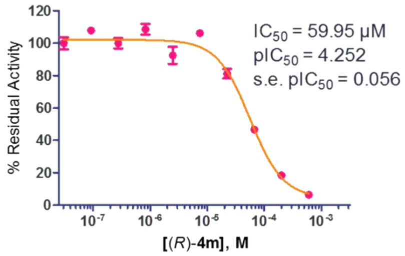

(*R*)-**4m** to class D OXA-48

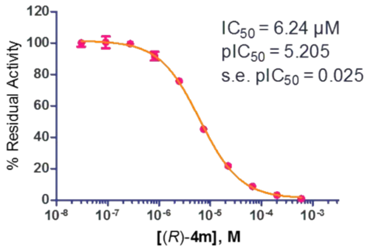

(*R*)-**4m** to class B1 NDM-1

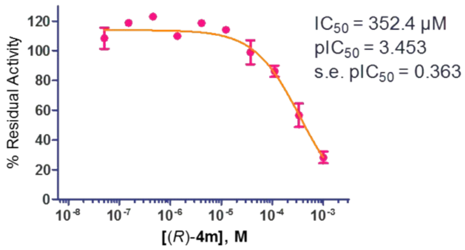

(*R*)-**4m** to class B1 VIM-2

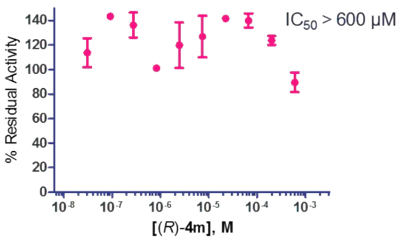

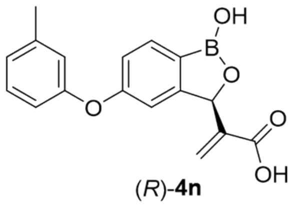

(*R*)-**4n**

(*R*)-**4n** to class A KPC-2

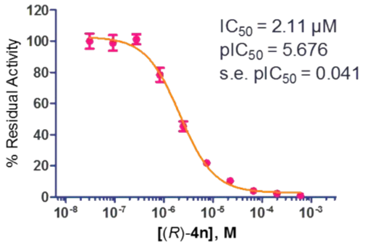

(*R*)-**4n** to class C AmpC

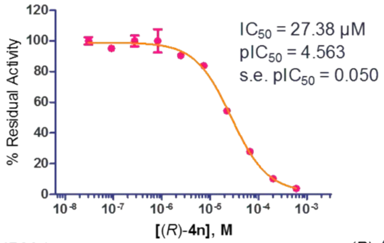

(*R*)-**4n** to class D OXA-48

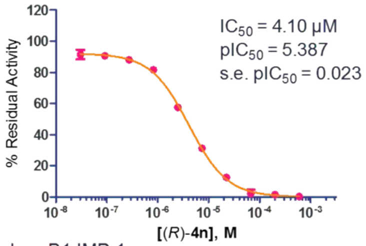

(*R*)-**4n** to class B1 NDM-1

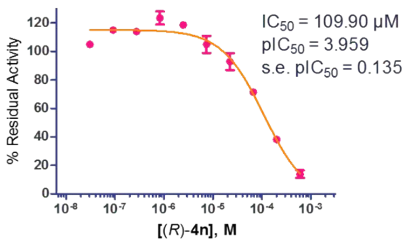

(*R*)-**4n** to class B1 IMP-1

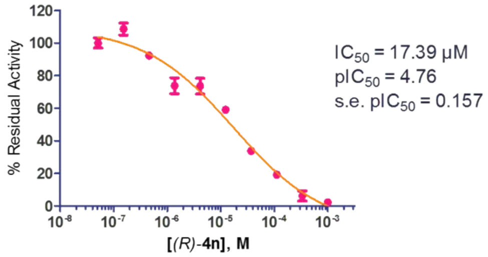

(*R*)-**4n** to class B1 VIM-1

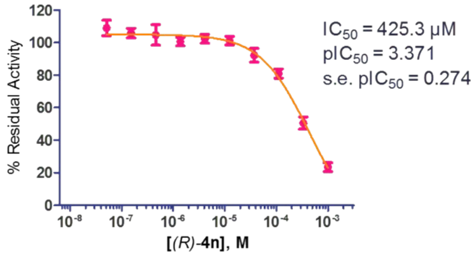

(*R*)-**4n** to class B1 VIM-2

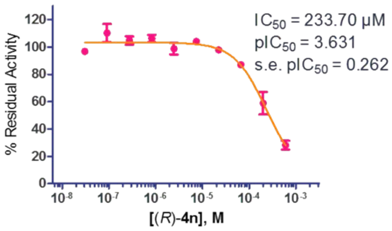

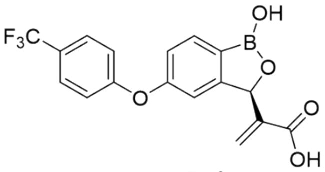

(*R*)-**4o**

(*R*)-**4o** to class A KPC-2

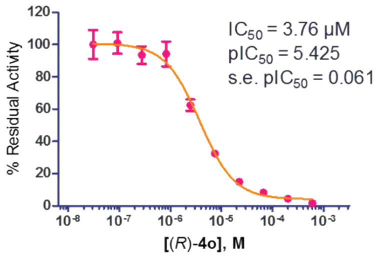

(*R*)-**4o** to class C AmpC

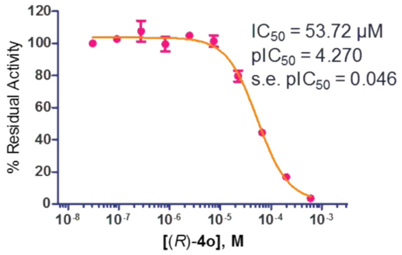

(*R*)-**4o** to class D OXA-48

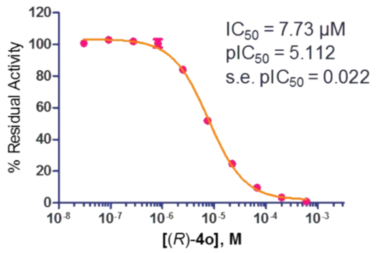

(*R*)-**4o** to class B1 NDM-1

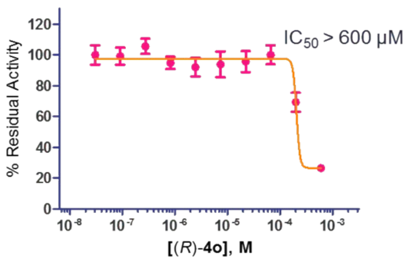

(*R*)-**4o** to class B1 IMP-1

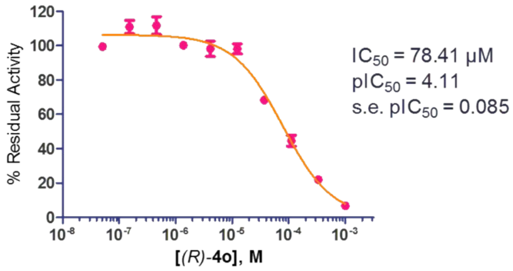

(*R*)-**4o** to class B1 VIM-1

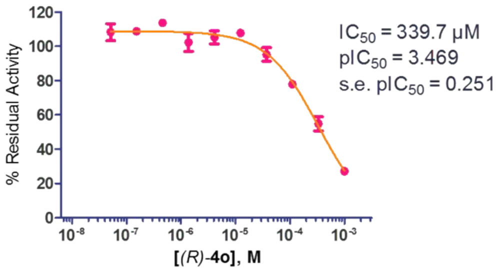

(*R*)-**4o** to class B1 VIM-2

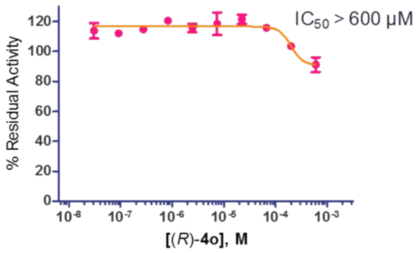

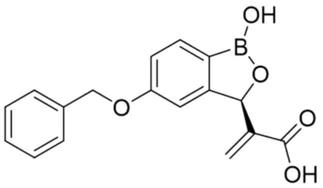

(*R*)-**4p**

(*R*)-**4p** to class A KPC-2

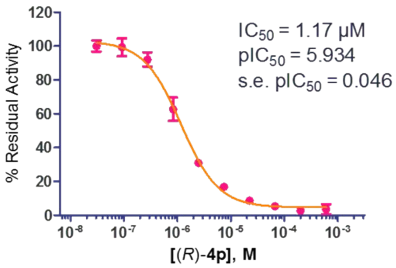

(*R*)-**4p** to class C AmpC

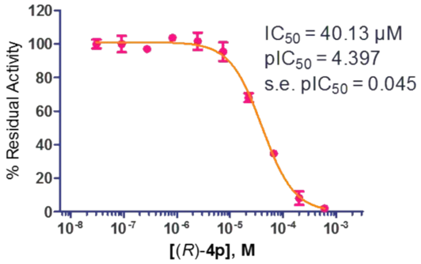

(*R*)-**4p** to class D OXA-48

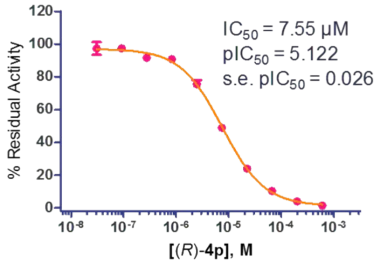

(*R*)-**4p** to class B1 NDM-1

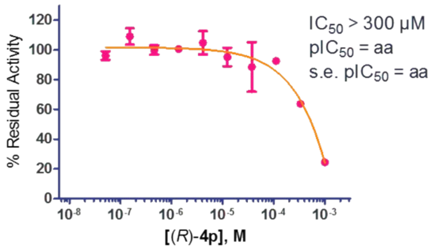

(*R*)-**4p** to class B1 IMP-1

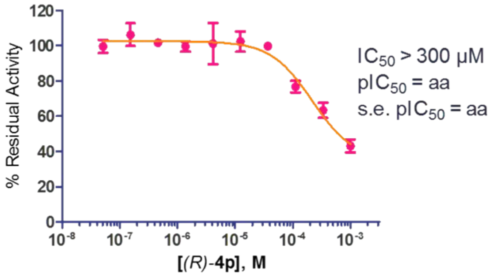

(*R*)-**4p** to class B1 VIM-1

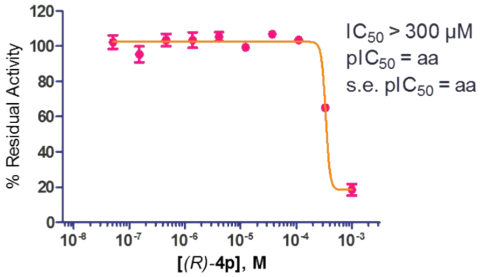

(*R*)-**4p** to class B1 VIM-2

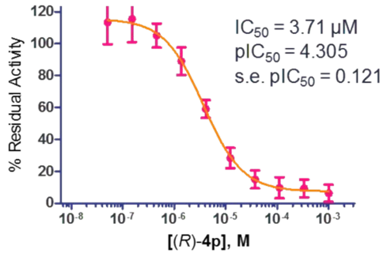

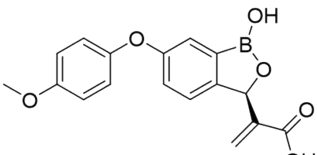

(*R*)-**4q**

(*R*)-**4q** to class A KPC-2

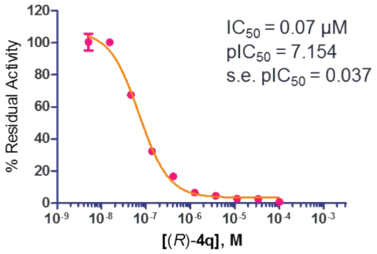

(*R*)-**4q** to class C AmpC

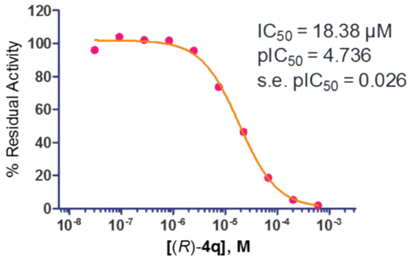

(*R*)-**4q** to class D OXA-48

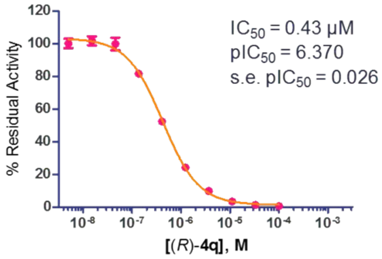

(*R*)-**4q** to class B1 NDM-1

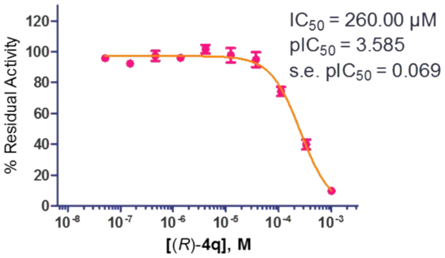

(*R*)-**4q** to class B1 IMP-1

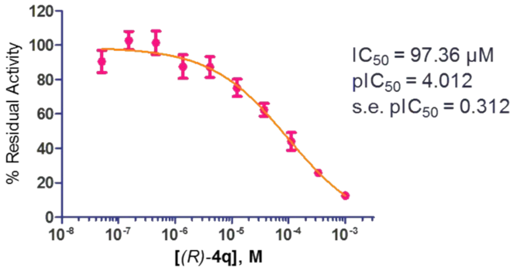

(*R*)-**4q** to class B1 VIM-1

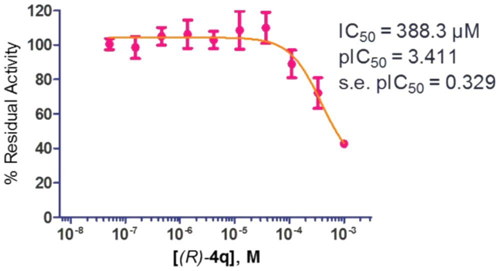

(*R*)-**4q** to class B1 VIM-2

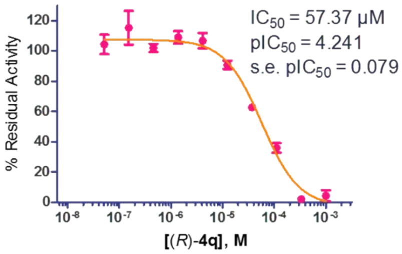

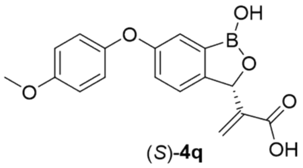

(*S*)-**4q**

(*S*)-**4q** to class A KPC-2

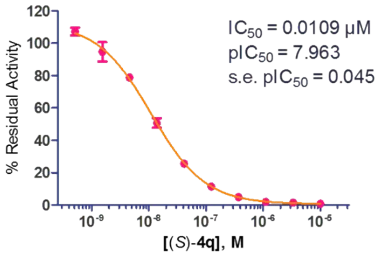

(*S*)-**4q** to class C AmpC

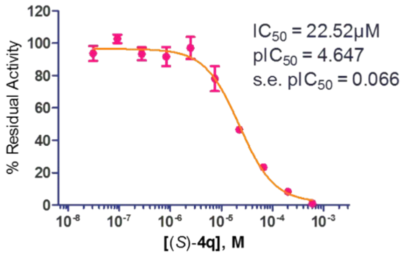

(*S*)-**4q** to class D OXA-48

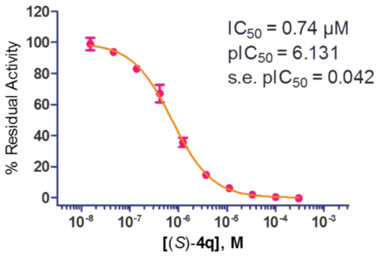

(*S*)-**4q** to class B1 NDM-1

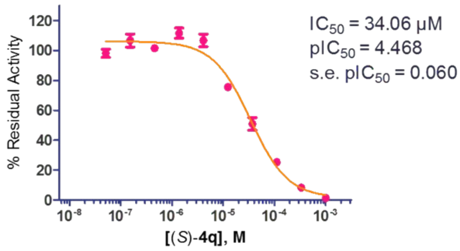

(*S*)-**4q** to class B1 IMP-1

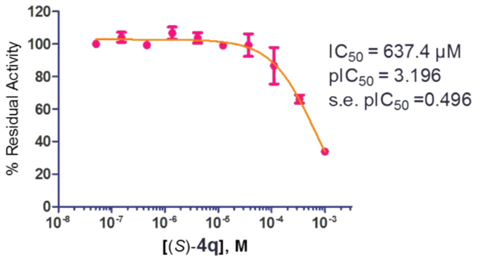

(*S*)-**4q** to class B1 VIM-1

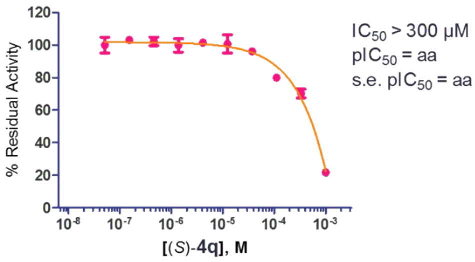

(*S*)-**4q** to class B1 VIM-2

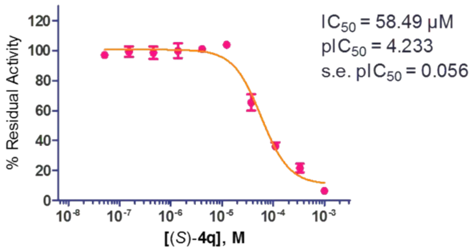

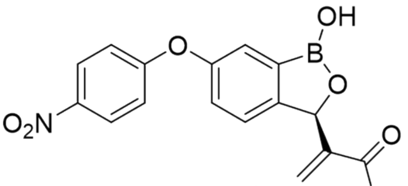

(*R*)-**4r**

(*R*)-**4r** to class A KPC-2

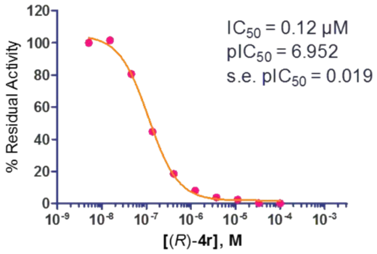

(*R*)-**4r** to class C AmpC

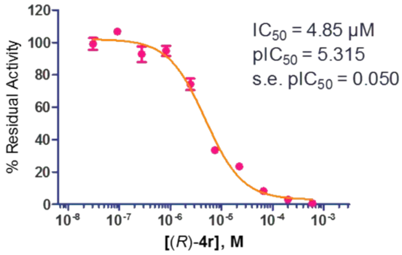

(*R*)-**4r** to class D OXA-48

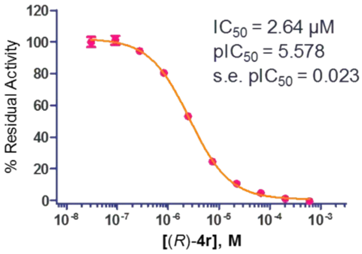

(*R*)-**4r** to class B1 NDM-1

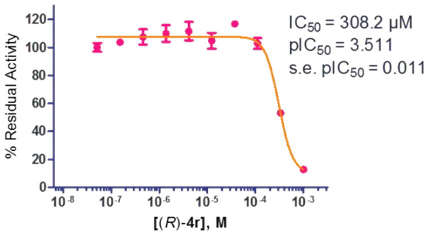

(*R*)-**4r** to class B1 IMP-1

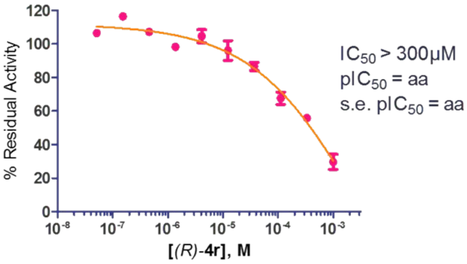

(*R*)-**4r** to class B1 VIM-1

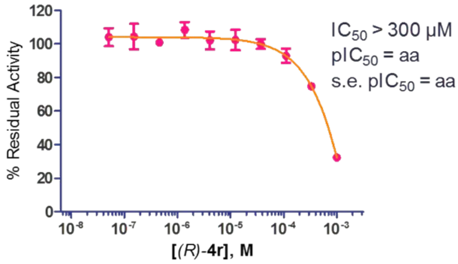

(*R*)-**4r** to class B1 VIM-2

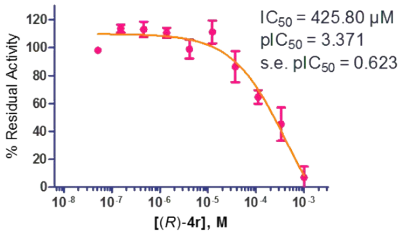

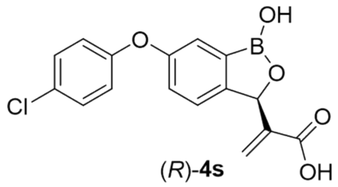
OH
B
O
O
Cl
O
OH
(R)-4s

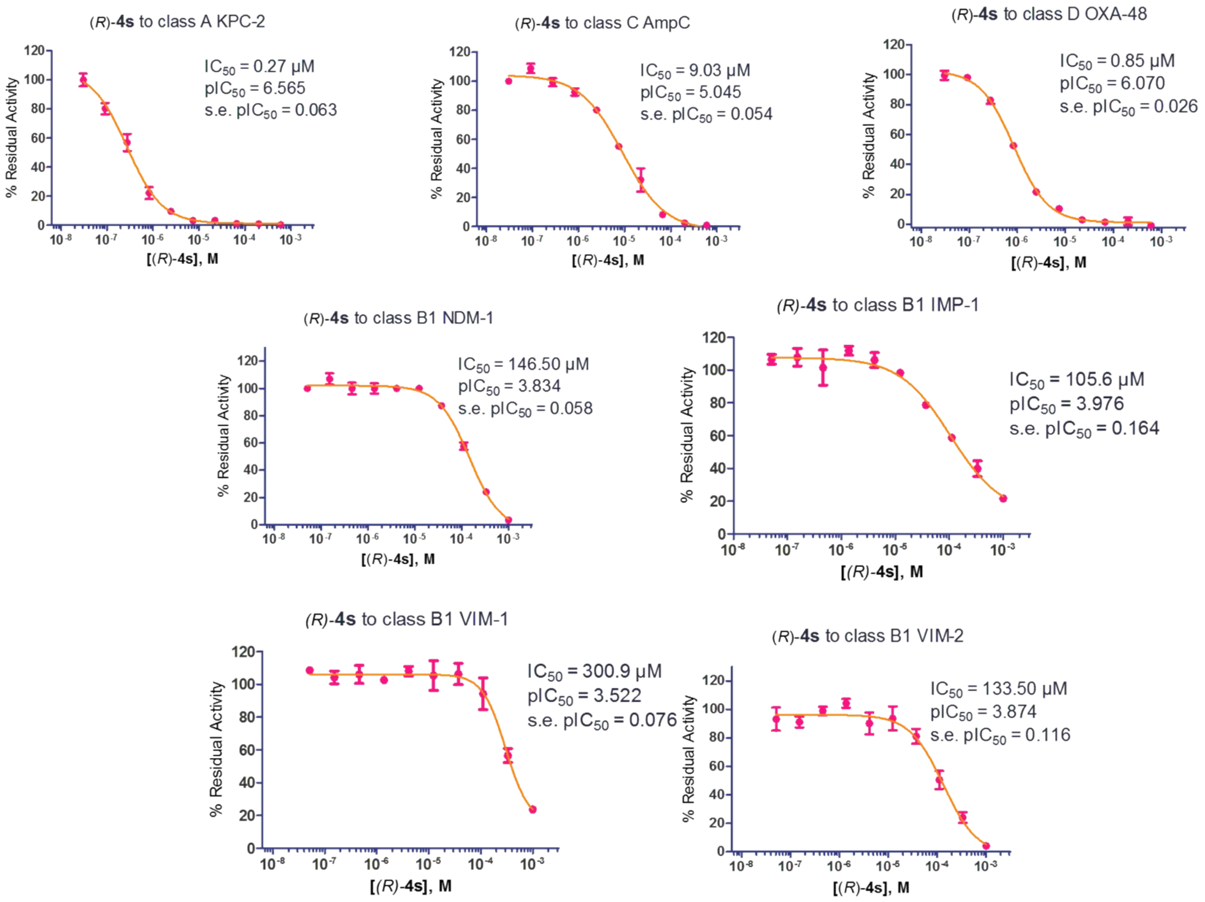
(R)-4s to class A KPC-2
IC50 = 0.27 μM
pIC50 = 6.565
s.e. pIC50 = 0.063
% Residual Activity
[(R)-4s], M
(R)-4s to class C AmpC
IC50 = 9.03 μM
pIC50 = 5.045
s.e. pIC50 = 0.054
(R)-4s to class D OXA-48
IC50 = 0.85 μM
pIC50 = 6.070
s.e. pIC50 = 0.026
(R)-4s to class B1 NDM-1
IC50 = 146.50 μM
pIC50 = 3.834
s.e. pIC50 = 0.058
(R)-4s to class B1 IMP-1
IC50 = 105.6 μM
pIC50 = 3.976
s.e. pIC50 = 0.164
(R)-4s to class B1 VIM-1
IC50 = 300.9 μM
pIC50 = 3.522
s.e. pIC50 = 0.076
(R)-4s to class B1 VIM-2
IC50 = 133.50 μM
pIC50 = 3.874
s.e. pIC50 = 0.116

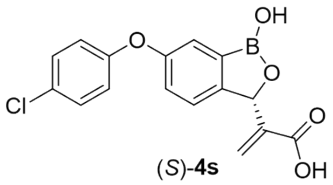
OH
B
O
O
Cl
O
OH

(*S*)-**4s**

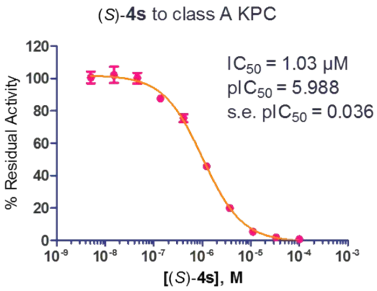
(S)-4s to class A KPC
IC50 = 1.03 µM
pIC50 = 5.988
s.e. pIC50 = 0.036
% Residual Activity
[(S)-4s], M

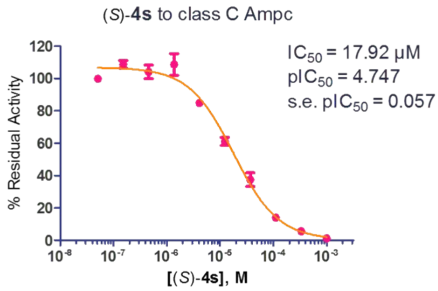
(S)-4s to class C Ampc
IC50 = 17.92 µM
pIC50 = 4.747
s.e. pIC50 = 0.057
% Residual Activity
[(S)-4s], M

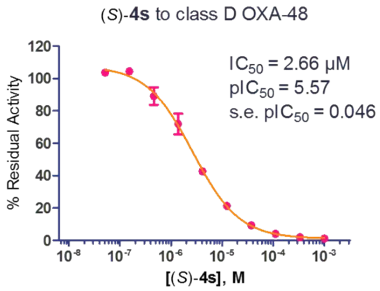
(S)-4s to class D OXA-48
IC50 = 2.66 µM
pIC50 = 5.57
s.e. pIC50 = 0.046
% Residual Activity
[(S)-4s], M

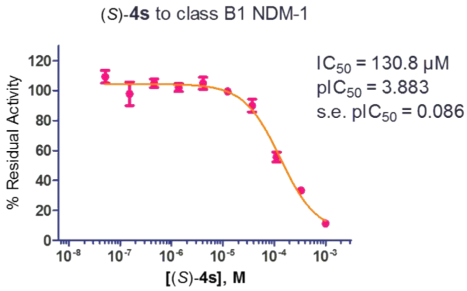
(S)-4s to class B1 NDM-1
IC50 = 130.8 µM
pIC50 = 3.883
s.e. pIC50 = 0.086
% Residual Activity
[(S)-4s], M

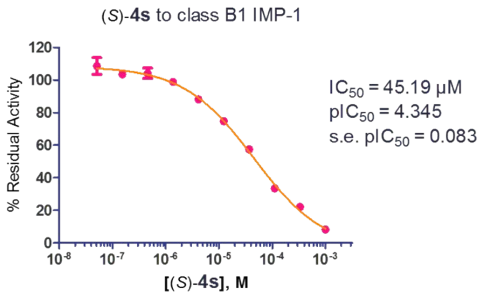
(S)-4s to class B1 IMP-1
IC50 = 45.19 µM
pIC50 = 4.345
s.e. pIC50 = 0.083
% Residual Activity
[(S)-4s], M

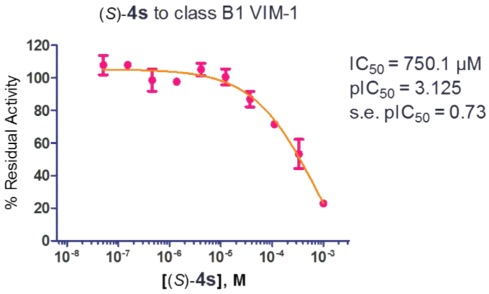
(S)-4s to class B1 VIM-1
IC50 = 750.1 µM
pIC50 = 3.125
s.e. pIC50 = 0.73
% Residual Activity
[(S)-4s], M

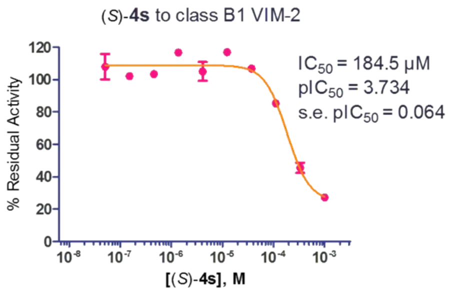
(S)-4s to class B1 VIM-2
IC50 = 184.5 µM
pIC50 = 3.734
s.e. pIC50 = 0.064
% Residual Activity
[(S)-4s], M

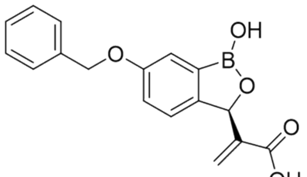

(*R*)-**4t**

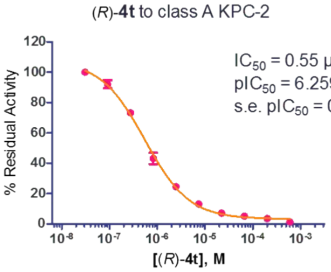
(R)-4t to class A KPC-2
IC50 = 0.55 μM
pIC50 = 6.259
s.e. pIC50 = 0.040
% Residual Activity
[(R)-4t], M

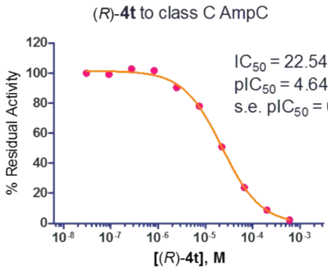
(R)-4t to class C AmpC
IC50 = 22.54 μM
pIC50 = 4.647
s.e. pIC50 = 0.025
% Residual Activity
[(R)-4t], M

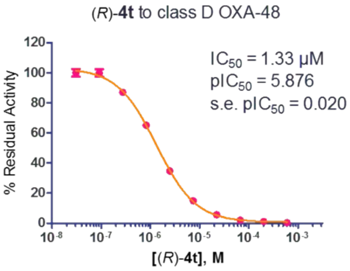
(R)-4t to class D OXA-48
IC50 = 1.33 μM
pIC50 = 5.876
s.e. pIC50 = 0.020
% Residual Activity
[(R)-4t], M

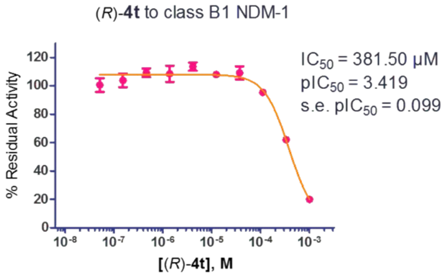
(R)-4t to class B1 NDM-1
IC50 = 381.50 μM
pIC50 = 3.419
s.e. pIC50 = 0.099
% Residual Activity
[(R)-4t], M

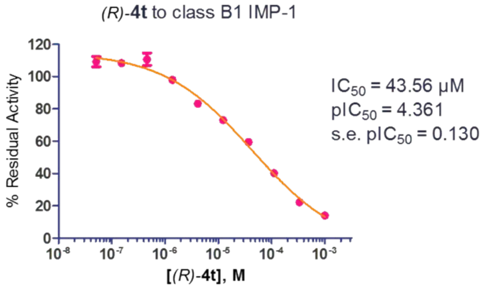
(R)-4t to class B1 IMP-1
IC50 = 43.56 μM
pIC50 = 4.361
s.e. pIC50 = 0.130
% Residual Activity
[(R)-4t], M

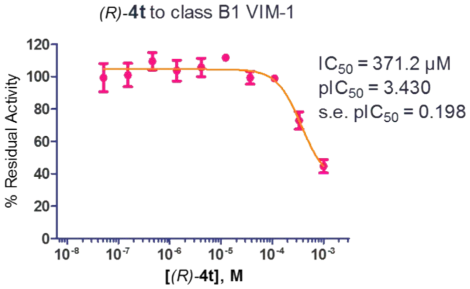
(R)-4t to class B1 VIM-1
IC50 = 371.2 μM
pIC50 = 3.430
s.e. pIC50 = 0.198
% Residual Activity
[(R)-4t], M

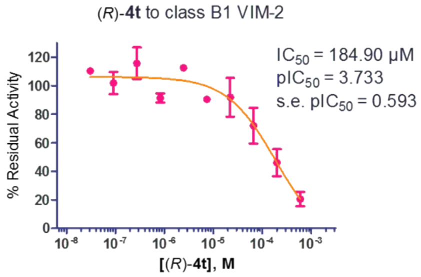
(R)-4t to class B1 VIM-2
IC50 = 184.90 μM
pIC50 = 3.733
s.e. pIC50 = 0.593
% Residual Activity
[(R)-4t], M

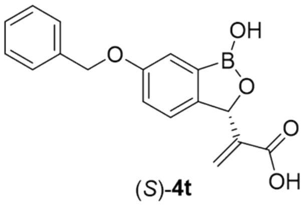

(*S*)-**4t**

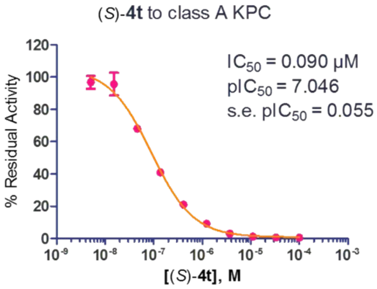

(S)-4t to class A KPC
IC50 = 0.090 μM
pIC50 = 7.046
s.e. pIC50 = 0.055
% Residual Activity
[(S)-4t], M

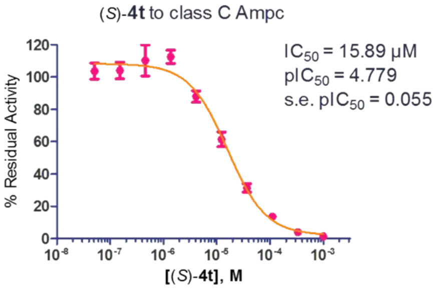

(S)-4t to class C Ampc
IC50 = 15.89 μM
pIC50 = 4.779
s.e. pIC50 = 0.055
% Residual Activity
[(S)-4t], M

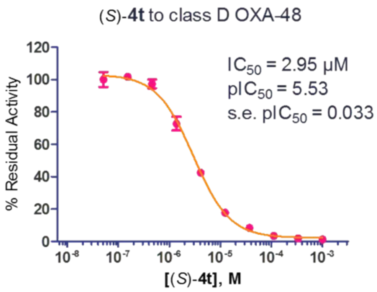

(S)-4t to class D OXA-48
IC50 = 2.95 μM
pIC50 = 5.53
s.e. pIC50 = 0.033
% Residual Activity
[(S)-4t], M

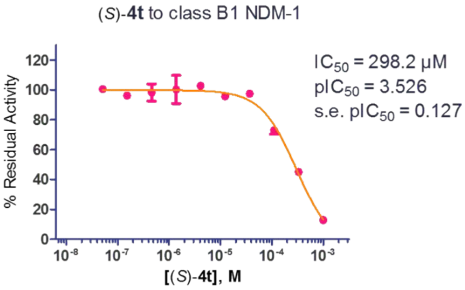

(S)-4t to class B1 NDM-1
IC50 = 298.2 μM
pIC50 = 3.526
s.e. pIC50 = 0.127
% Residual Activity
[(S)-4t], M

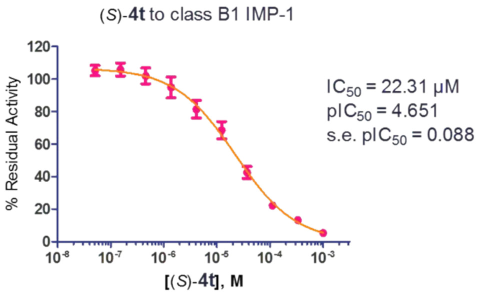

(S)-4t to class B1 IMP-1
IC50 = 22.31 μM
pIC50 = 4.651
s.e. pIC50 = 0.088
% Residual Activity
[(S)-4t], M

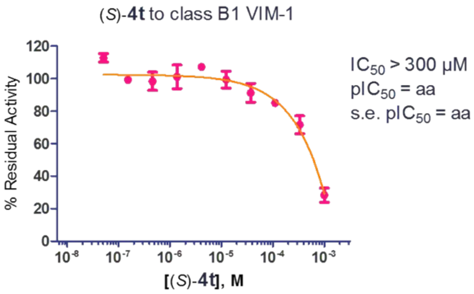

(S)-4t to class B1 VIM-1
IC50 > 300 μM
pIC50 = aa
s.e. pIC50 = aa
% Residual Activity
[(S)-4t], M

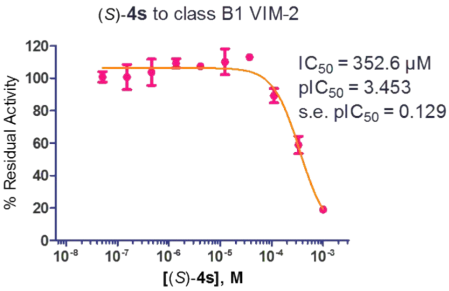

(S)-4s to class B1 VIM-2
IC50 = 352.6 μM
pIC50 = 3.453
s.e. pIC50 = 0.129
% Residual Activity
[(S)-4s], M

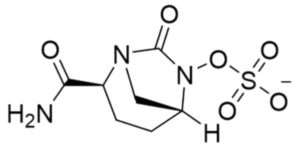

Avibactam

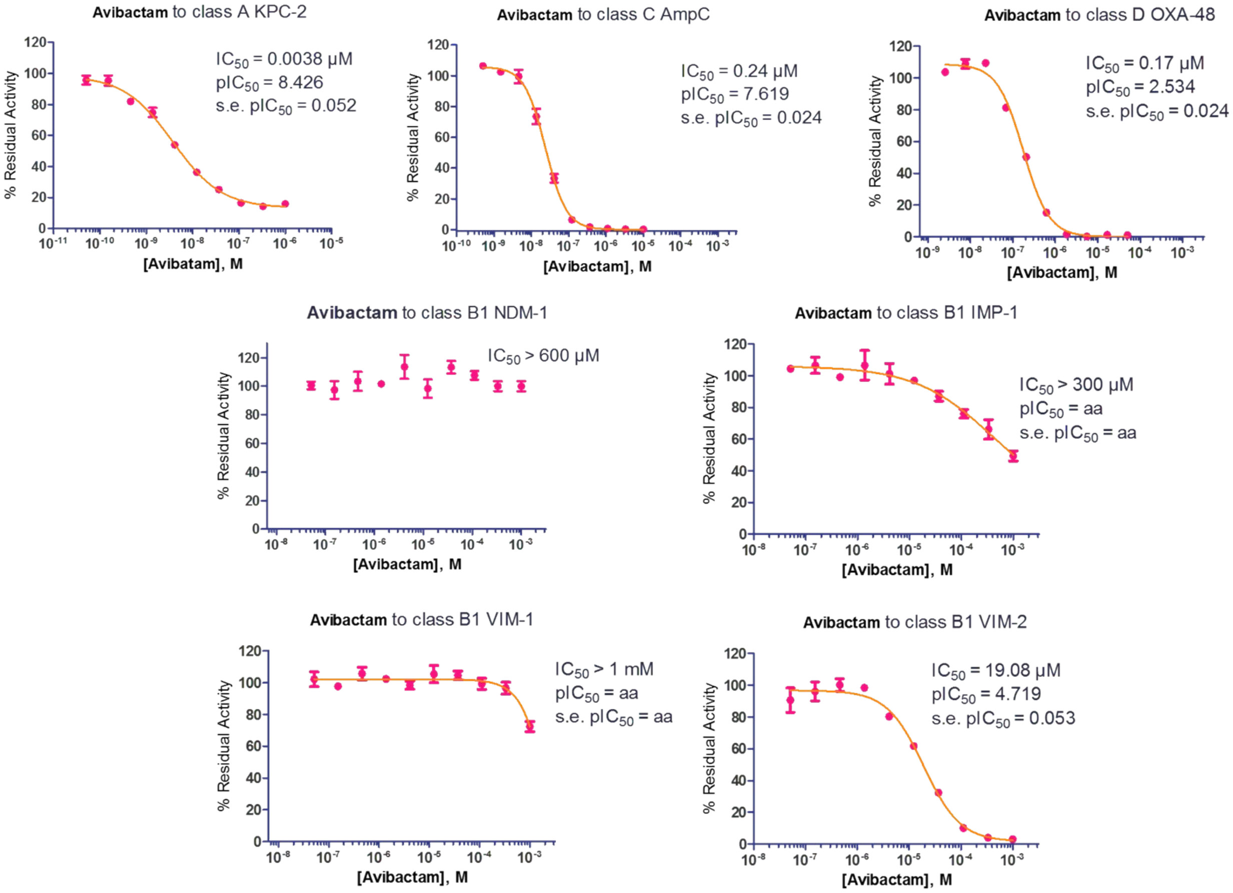

Figure S1. IC_{50} curves of 4a-4t **and avibactam with KPC-2, AmpC, OXA-48, NDM-1, IMP-1, VIM-1, and VIM-2.** The concentrations of enzymes used were: KPC-2, 2.5 nM; AmpC, 0.8 nM; OXA-48, 20 nM; NDM-1, 0.2 nM; IMP-1, 0.8 nM; VIM-1, 0.2 nM; and VIM-2, 0.2 nM; and 5 μM FC-5 substrate were employed for these enzymes. The assay buffer was supplemented with 0.01% (v/v) Triton X-100 for all the tested enzymes.

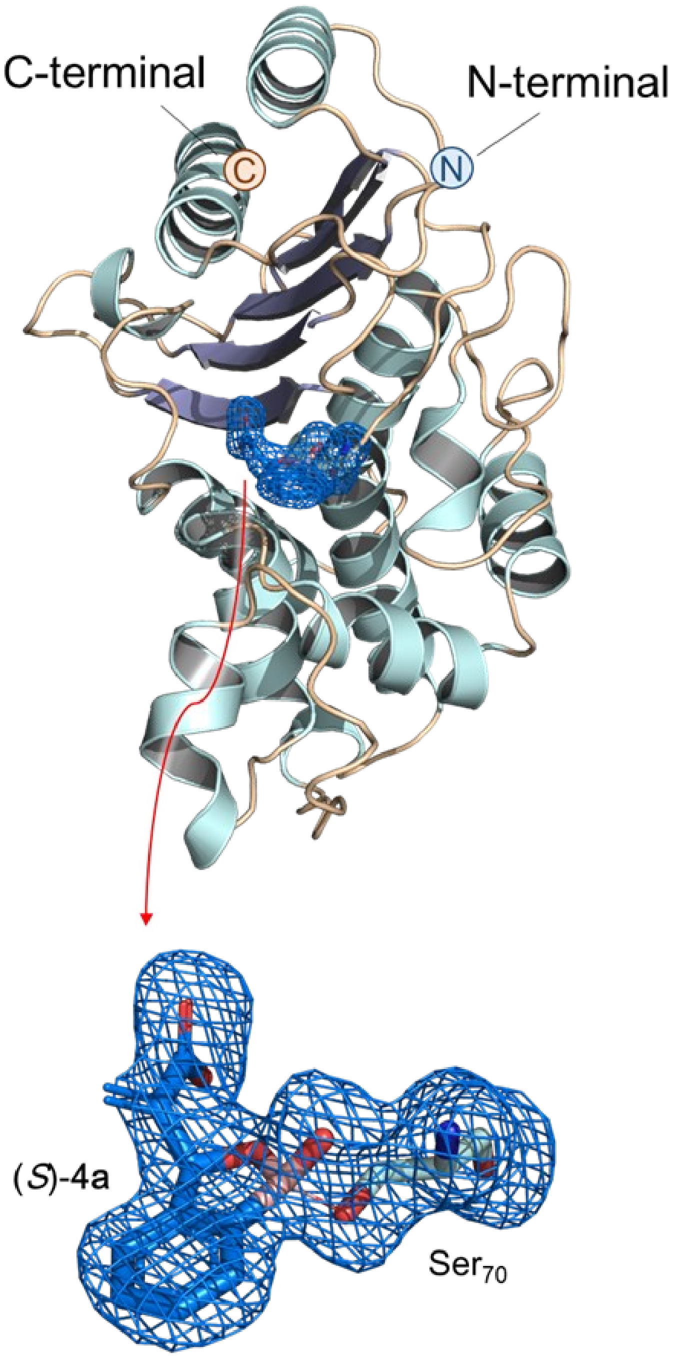

Figure S2. The mode of (*S*)-4a binding to KPC-2 as defined by the electron density map. A view from a structure of the KPC-2:(*S*)-**4a** (PDB ID 7E9A) complex with the mF_o-DF_c electron density (OMIT maps) around (*S*)-**4a** (blue mesh, contoured to 3σ) calculated from the final refined model shown.

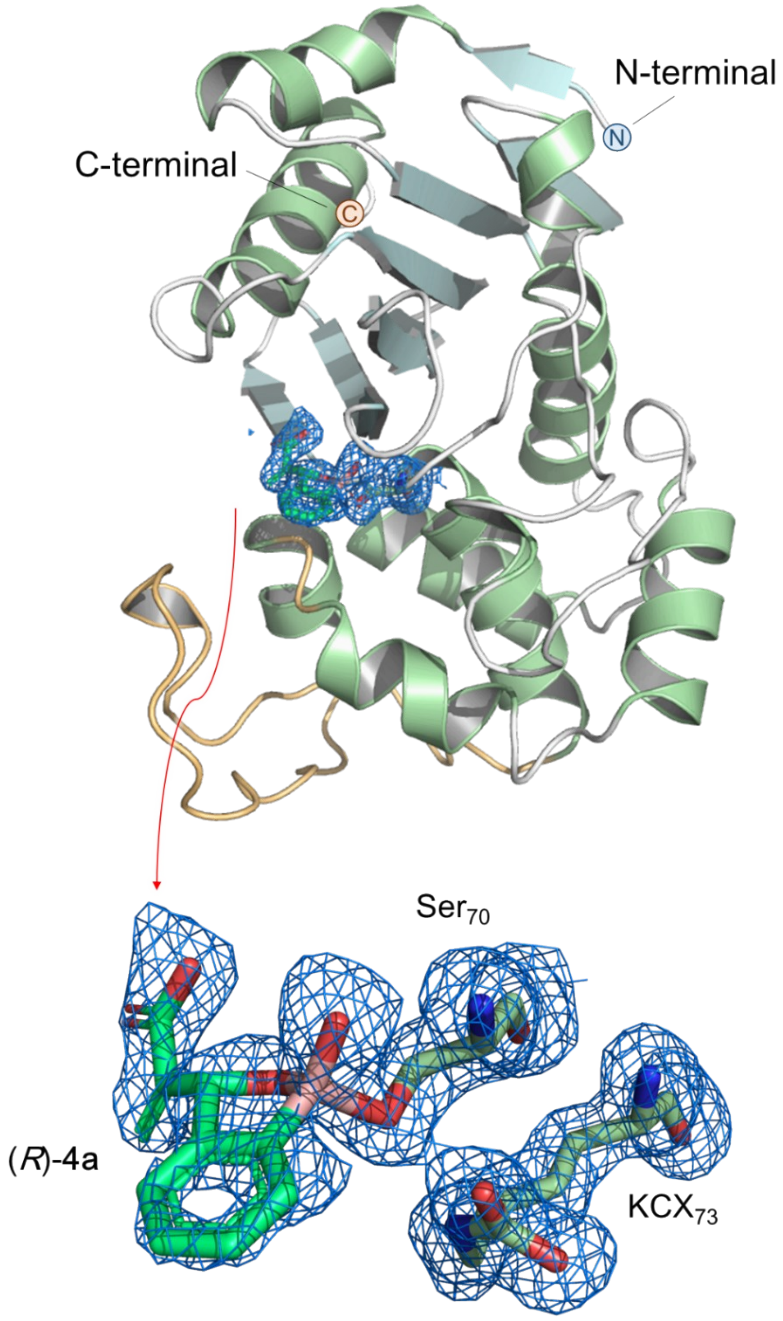

Figure S3. The mode of (*R*)-4a **binding to OXA-48 as defined by the electron density map.** A view from a structure of OXA-48:(*R*)-**4a** (PDB ID 7DML) with the mF_o-DF_c electron density (OMIT map) around (*R*)-**4a** and the catalytically relevant carbamylated Lys73 (KCX73) (blue mesh, contoured to 3σ) calculated from the final refined model shown.

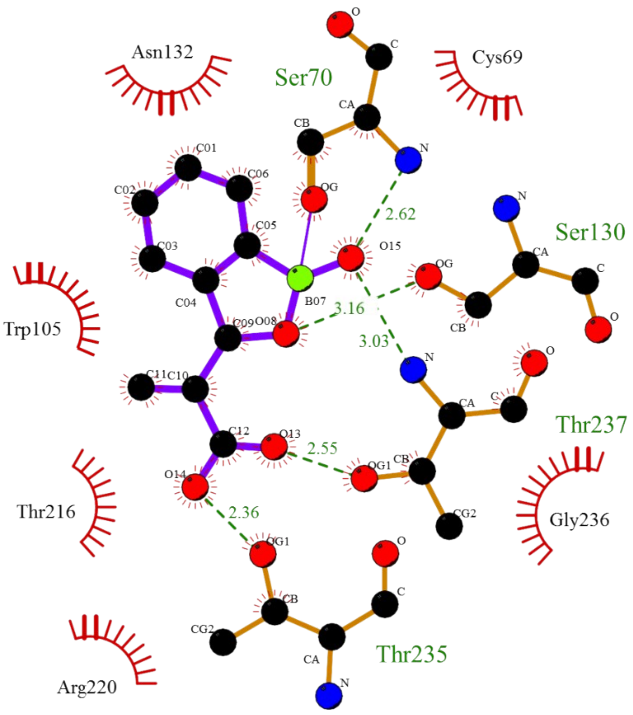

KPC-2:(*S*)-**4a** (PDB code 7E9A)

Figure S4. Protein-ligand interactions between (*S*)-4a and KPC-2 as defined using the LigPlot+ program.[10]

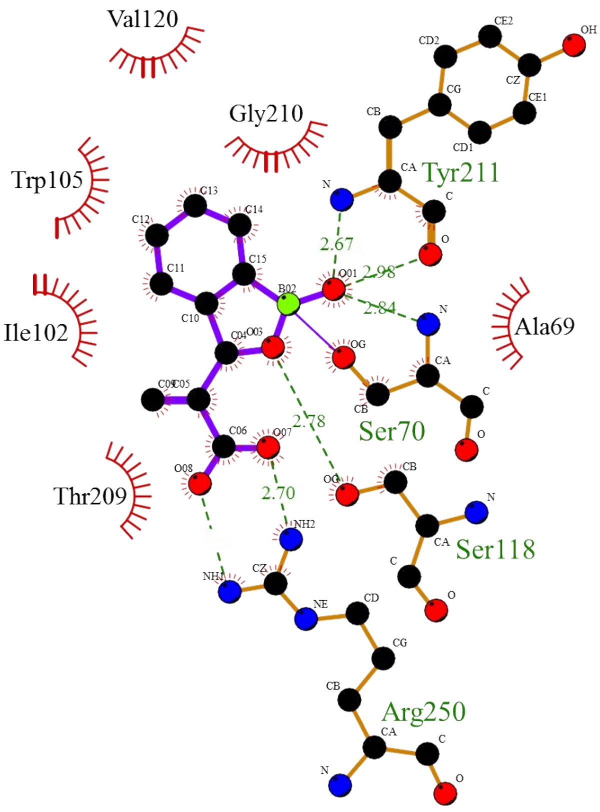

OXA-48:(*R*)-**4a** (PDB code 7DML)

Figure S5. Protein-ligand interactions between (*R*)-4a and OXA-48 as defined using the LigPlot+ program.[9]

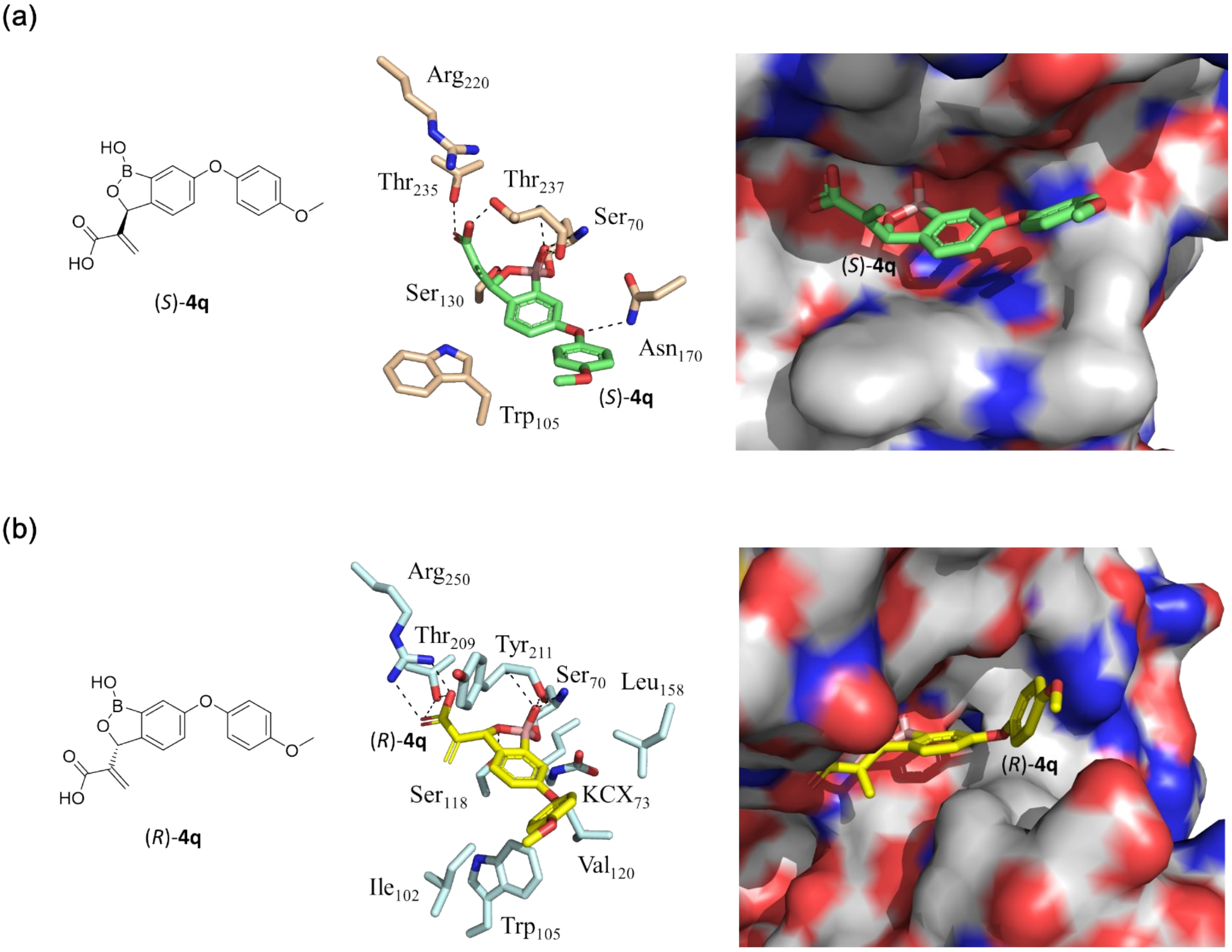

Figure S6. Proposed binding modes of (S)-4q/**(R)-**4q **with KPC-2 and OXA-48.** (a) The C-6 4-methoxyphenoxyl group of (*S*)-**4q** may form an additional hydrogen bond with Asn_{170} of KPC-2. (b) (*S*)-**4q** may occupy the hydrophobic subpocket surrounded by Ile_{102}, Trp_{105}, Val_{120}, and Leu_{158} of OXA-48.

(*S*)-**4a** vs Hydrolysed Faropenem

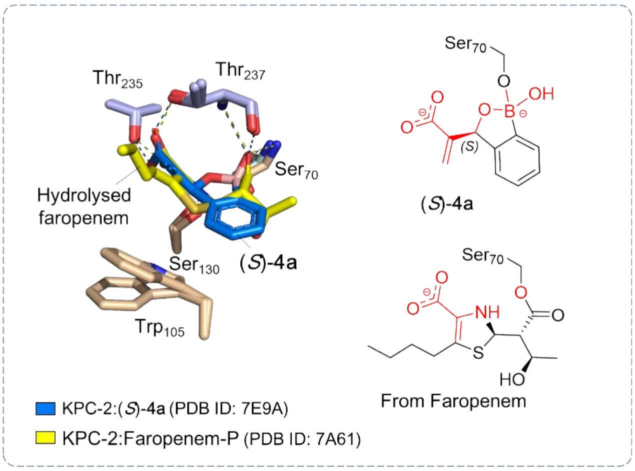

(*S*)-**4a** vs Hydrolyzed Faropenem

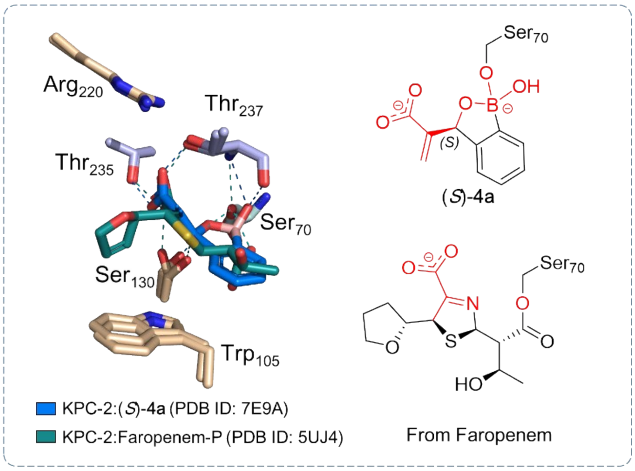

(*S*)-**4a** vs Hydrolyzed Ampicillin

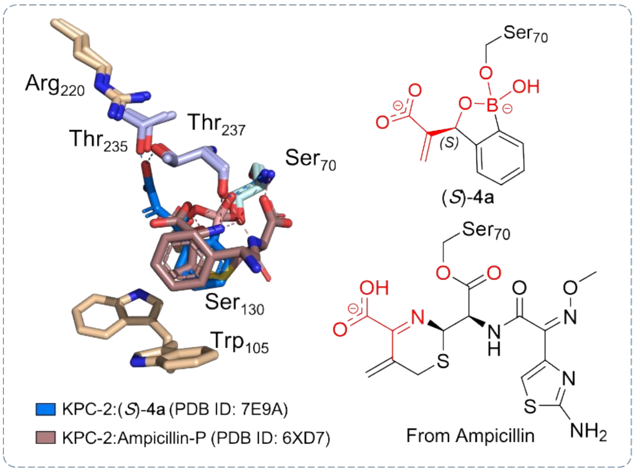

(*S*)-**4a** vs Hydrolyzed Cefotaxime

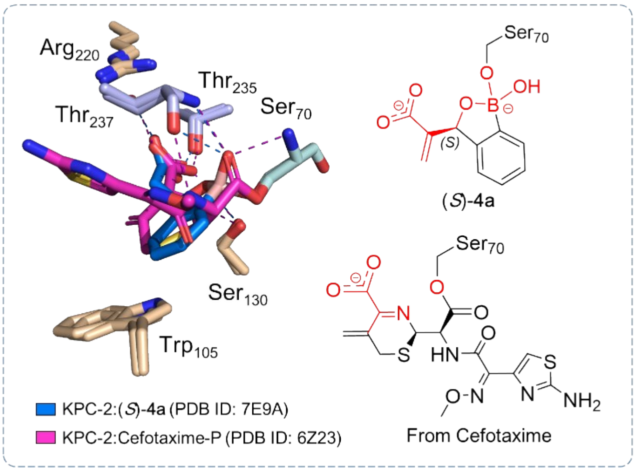

(*R*)-**4a** vs Hydrolyzed Imipenem

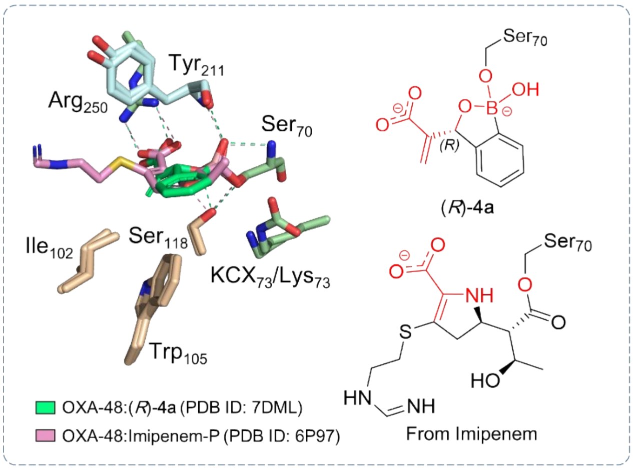

(*R*)-**4a** vs Hydrolyzed Ertapenem

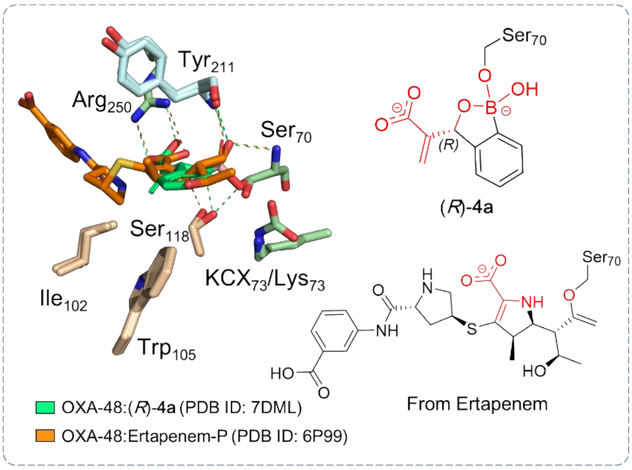

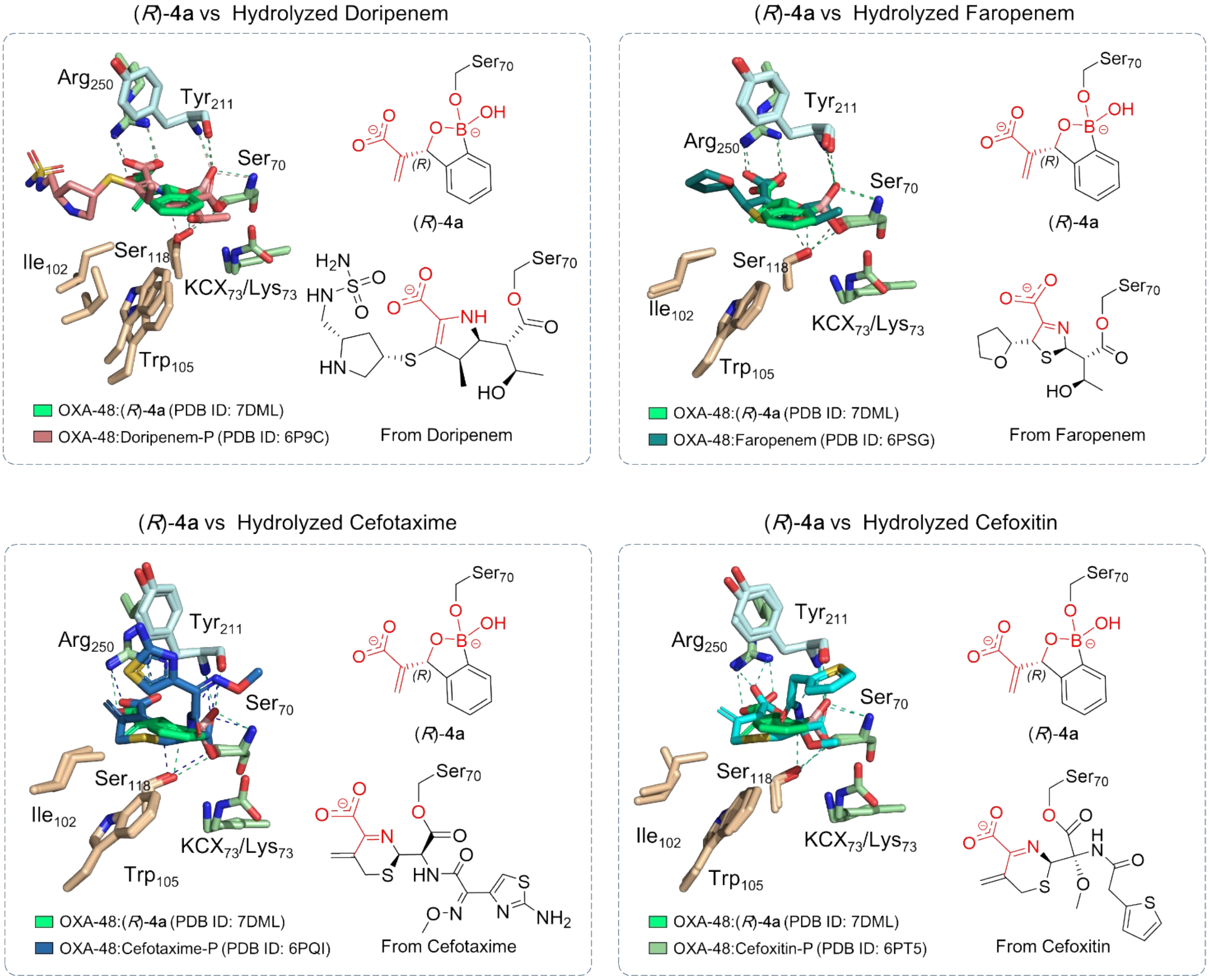

Figure S7. Comparison of KPC-2:(*S*)-4a**/OXA-48:(*R*)-**4a** structures with those of KPC-2/OXA-48 reacted with β-lactam substrates.** β-Lactam substrate reacted (designated by -P) complexes include with faropenem (PDB: 7A61, 5UJ4, and 6PSG), ampicillin (PDB: 6XD7), cefotaxime (PDB: 6Z23), imipenem (PDB: 6P97), ertapenem (PDB: 6P99), doripenem (PDB: 6P9C), cefotaxime (PDB: 6PQI), and cefoxitin (PDB: 6PT5). Note that (*S*)-**4a** and (*R*)-**4a** reaction / binding mimics that of β-lactams, particularly with respect to conserved anchoring features, in particular when comparing reaction of the electrophilic β-lactam carbonyl (observed in the acyl-enzyme complexes) and inhibitor boron, as well as in terms of the substrate/inhibitor carboxylate positions.

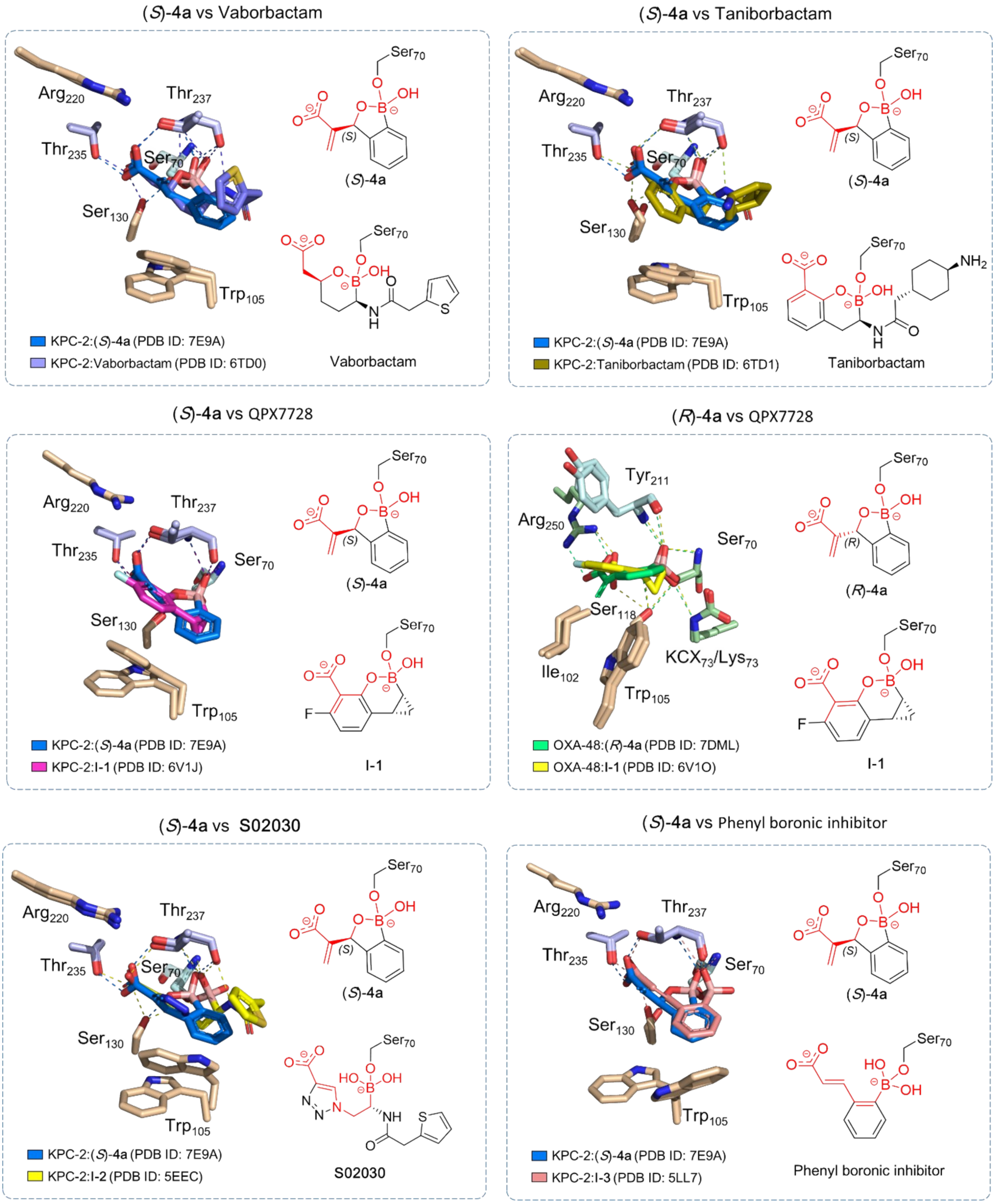

Figure S8. Comparison of KPC-2:(*S*)-4a**/OXA-48:(*R*)-**4a** structures with those of KPC-2/OXA-48 in complex with other boron-containing inhibitors.** The latter comprise vaborbactam (PDB: 6TD0), taniborbactam (PDB: 6TD1), QPX7728 (PDB: 6V1J and 6V1O), S02030 (PDB: 5EEC), and a phenyl boronic inhibitor (PDB: 5LL7); the comparisons reveal related binding modes, highlighting the importance of mimicking anchoring interactions involved in substate / intermediate binding.

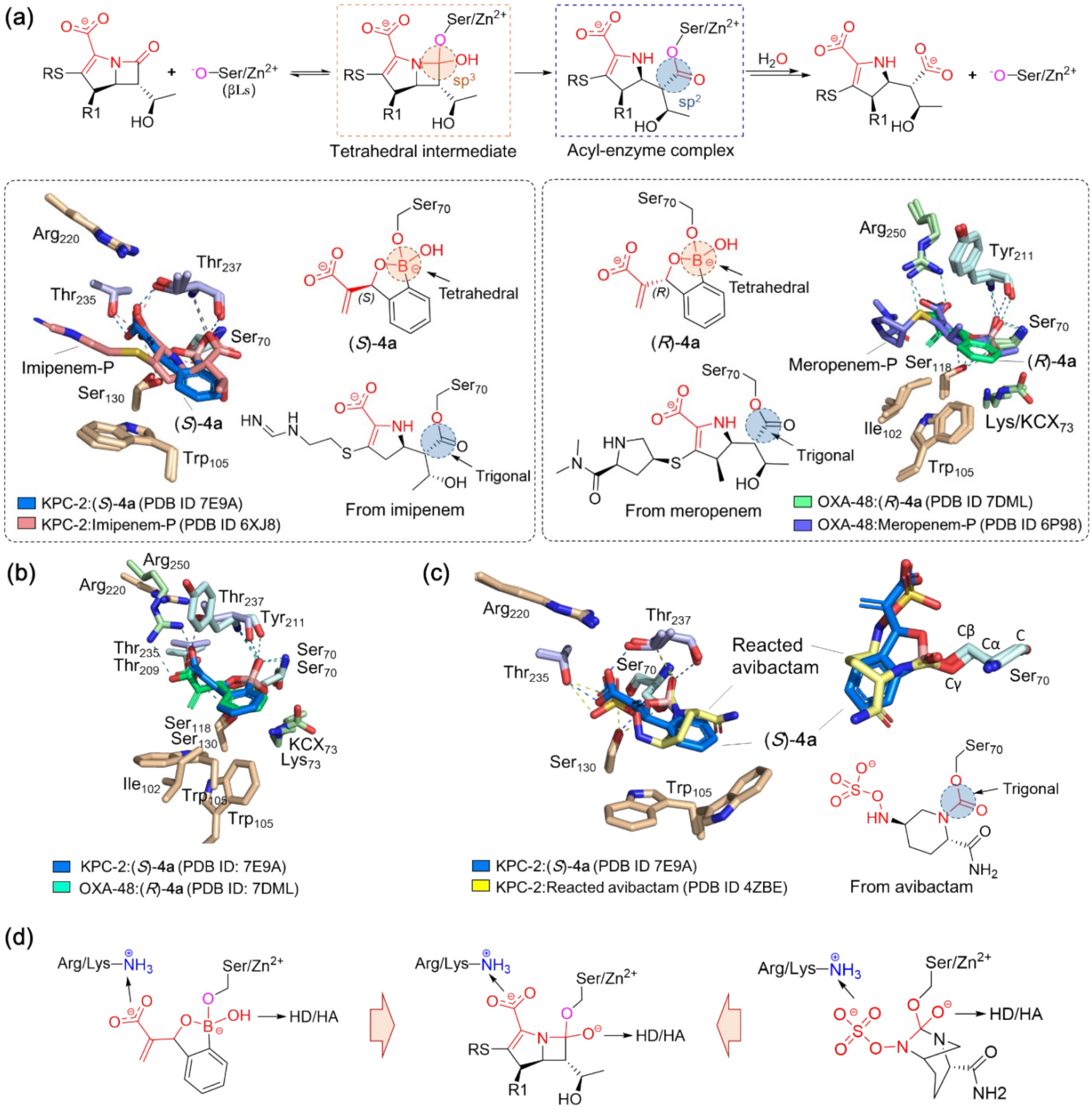

Figure S9. Binding modes of the new C-3 substituted benzoxaboroles mimics those of proposed intermediates in carbapenemase catalysis. (a) Carbapenem hydrolysis catalysed by serine and metallo β-lactamases. Comparisons of structures of KPC-2:(*S*)-**4a** (blue) with KPC-2:reacted imipenem (pink) (PDB: 6XJ8), and OXA-48:(*R*)-**4a** (green) with OXA-48:reacted meropenem (purple) (PDB: 6P98) imply that (*S*)-**4a** and (*R*)-**4a** have similar binding modes compared with those of (reacted) carbapenem (and other β-lactam, Fig. S7) antibiotics, particularly with respect to conserved 'anchoring' features, i.e. of the electrophilic β-lactam substrate carbonyl (observed as the acyl-enzyme)/inhibitor boron and the substrate/inhibitor carboxylate binding modes. (b) Superimposition of KPC-2:(*S*)-**4a** and OXA-48:(*R*)-**4a** structures reveals (*S*)-**4a** and (*R*)-**4a** have similar binding modes to both carbapenemases. (c) Comparison of structures of KPC-2:(*S*)-**4a** (blue) with KPC-2:reacted avibactam (yellow) (PDB: 4ZBE) reveals similar binding modes and evidence for flexibility of

Trp105 in substrate/inhibitor binding. (d) Designing new carbapenemase inhibitors by mimicking the conserved anchoring features, exemplified here for avibactam and benzoxaboroles (for other boron-containing inhibitors, see Fig. S8), is a viable strategy in drug discovery targeting carbapenemases.

Supplementary Tables

Table S1. The inhibitory activities (IC_{50}, μM) of other synthesized benzoxaborole derivatives against representative serine- and metallo-carbapenemases.[a]

Cpd ID	Class A KPC-2	Class C AmpC	Class D OXA-48	Class B1 NDM-1	Class B1 IMP-1	Class B1 VIM-1	Class B1 VIM-2
(R)-**4b**	7.40	310.40	29.27	>600	>300	137.4	181.20
(R)-**4d**	1.88	110.30	2.57	>600	271.3	>300	>600
(R)-**4e**	21.69	125.00	30.82	>600	>300	757.6	189.80
(R)-**4g**	3.18	113.10	9.92	>600	NT	345.1	>600
(R)-**4h**	7.33	187.20	11.43	>600	788	>600	65.31
(R)-**4i**	1.68	146.40	24.91	430.5	421.4	118.8	127.70
(R)-**4k**	2.17	69.82	3.21	>600	136.6	587.8	49.52
(R)-**4l**	5.73	152.90	27.91	>600	120.1	763	>600
(R)-**4m**	3.58	59.95	6.24	352.40	NT	NT	>600
(R)-**4n**	2.11	27.38	4.10	109.90	17.39	425.3	233.70
(R)-**4o**	3.76	53.72	7.73	>600	78.41	339.7	>600
(R)-**4p**	1.17	40.13	7.55	>300	>300	>300	3.71
(R)-**4r**	0.12	4.85	2.64	308.20	>300	>300	425.80
(*R*)-**4s**	0.27	9.03	0.85	146.50	123.6	300.9	133.50
(*R*)-**4t**	0.55	22.54	1.33	381.50	43.56	371.2	184.9

[a] All of the tested compounds were obtained directly from the asymmetric MBH cascade reaction without further recrystallization.

Table S2. Co-crystallization Conditions for the OXA-48:(*R*)-**4a** and KPC-2:(*S*)-**4a** complexes.

Structure	Method	Sample composition	Crystallization condition	Vapor diffusion condition
KPC-2:(*S*)-**4a**	Co-crystallization	KPC-2 in crystallization buffer + inhibitor (*S*)-**4a** (5 mM)	32-35% PEG 8000, 0.1M lithium sulphate, 0.05M sodium acetate (pH 4.5)	Hanging drop, protein-to-well ratio, 1:1, 293K
OXA-48:(*R*)-**4a**	Co-crystallization	OXA-48 in crystallization buffer + inhibitor (*R*)-**4a** (3.5 mM)	0.1 M HEPES pH7.5, 5% 1-butanol, 11~17% (v/v) polyethylene glycol 8000	Hanging drop, protein-to-well ratio, 1:1, 293K

Table S3. Data Collection and Refinement Statistics for KPC-2:(*S*)-**4a** (PDB code 7E9A) and OXA-48:(*R*)-**4a** (PDB code 7DML) complex crystal structures.

Structure	KPC-2**:**(*S*)**-4a**	OXA-48:(*R*)**-4a**
PDB ID	7E9A	7DML
**Processing**		
Radiation Source	SSRF Beamline BL19U	SSRF Beamline BL17U
Space Group	*P* 3 2 1	*P* 1 2_1 1
Unit Cell Dimensions: a, b, c (Å)	165.42, 165.42, 94.86	58.45, 105.86, 94.12
Unit Cell Dimensions: α, β, γ (˚)	90.00, 90.00, 120.00	90.00, 107.81, 90.00
*Mol/ASU	4	4
Resolution Range (outer shell) (Å)	47.43- 2.25 (2.29- 2.25)	19.81-1.94 (2.01-1.94)
Number of Unique Reflections	70988	77650
Completeness (outer shell) (%)	95.5	88.04
$I/\sigma(I)$ (outer shell)	3.06	5.7
R_{merge} (outer shell)	1.701	0.258
Wilson B Factor (Å^2)	30.48	17.17
**Refinement**		
Overall B Factor (Å^2)	34.81	20.78
Protein B Factor (Å^2)	34.77	19.73
Ligand B Factor (Å^2) (occupancy)	30.79 (1.0)	25.72 (1.0)
Water B Factor (Å^2)	34.25	28.98
‡RMSD from Ideal Bond Length (Å)	0.012	0.014
RMSD from Ideal Angles (˚)	1.241	1.23
R_{work} (%)	18.04	13.80
R_{free} (%)	22.10	17.20

*Mol/ASU = molecules per asymmetric unit; ‡RMSD = root mean square deviation.

References

[1] P.-F. Zheng, Q. Ouyang, S.-L Niu, L. Shuai, Y. Yuan, K. Jiang, T.-Y Liu, Y.-C. Chen, *J. Am. Chem. Soc.* **2015**, *137*, 9390.

[2] G. Martelli, M. Orena, S. Rinaldi, *Eur. J. Org. Chem.* **2012**, *2012*, 4140.

[3] Y. Nakamoto, F. Urabe, K. Takahashi, J. Ishihara, S. Hatakeyama, *Chem. Eur. J.* . **2013**, *19*, 12653.

[4] J. L. L. F. Regueira, L. F. Silva, Jr, R. A. Pilli, *Org. Lett.* **2020**, *22*, 6262.

[5] Y. Iwabuchi, M. Nakatani, N. Yokoyama, S. Hatakeyama, *J. Am. Chem. Soc.* **1999**, *121*, 10219.

[6] a) Y.-L. Wang, S. Liu, Z.-J. Yu, Y. Lei, M.-Y. Huang, Y.-H. Yan, Q. Ma, Y. Zheng, H. Deng, Y. Sun, C. Wu, Y. Yu, Q. Chen, Z. Wang, Y. Wu, G.-B. Li, *J. Med. Chem.* **2019**, *62*, 7160-7184; b) S. Liu, L. Jing, Z.-J. Yu, C. Wu, Y. Zheng, E. Zhang, Q. Chen, Y. Yu, L. Guo, Y. Wu, G.-B. Li, *Eur. J. Med. Chem.* **2018**, *145*, 649-660; c) G.-B. Li, M. I. Abboud, J. Brem, H. Someya, C. T. Lohans, S.-Y. Yang, J. Spencer, D. W. Wareham, M. A. McDonough, C. J. Schofield, *Chem. Sci.* **2017**, *8*, 928-937.

[7] S. S. van Berkel, J. Brem, A. M. Rydzik, R. Salimraj, R. Cain, A. Verma, R. J. Owens, C. W. Fishwick, J. Spencer, C. J. Schofield, *J. Med. Chem.* **2013**, *56*, 6945-6953.

[8] P. D. Adams, P. V. Afonine, G. Bunkoczi, V. B. Chen, I. W. Davis, N. Echols, J. J. Headd, L. W. Hung, G. J. Kapral, R. W. Grosse-Kunstleve, A. J. McCoy, N. W. Moriarty, R. Oeffner, R. J. Read, D. C. Richardson, J. S. Richardson, T. C. Terwilliger, P. H. Zwart, *Acta Crystallogr. D Biol. Crystallogr.* **2010**, *66*, 213-221.

[9] P. Emsley, K. Cowtan, *Acta Crystallogr. D Biol. Crystallogr.* **2004**, *60*, 2126-2132.

[10] R. A. Laskowski, M. B. Swindells, *J. Chem. Inf. Model.* **2011**, *51*, 2778-2786.
